# Phenanthrenequinone-Sensitized Photocatalytic Synthesis of Polysubstituted Quinolines from 2-Vinylarylimines

**DOI:** 10.1021/acs.orglett.1c03934

**Published:** 2021-12-20

**Authors:** Juulia Talvitie, Iida Alanko, Evgeny Bulatov, Juho Koivula, Topias Pöllänen, Juho Helaja

**Affiliations:** Department of Chemistry, University of Helsinki, A. I. Virtasen aukio 1, 00014 Helsinki, Finland

## Abstract

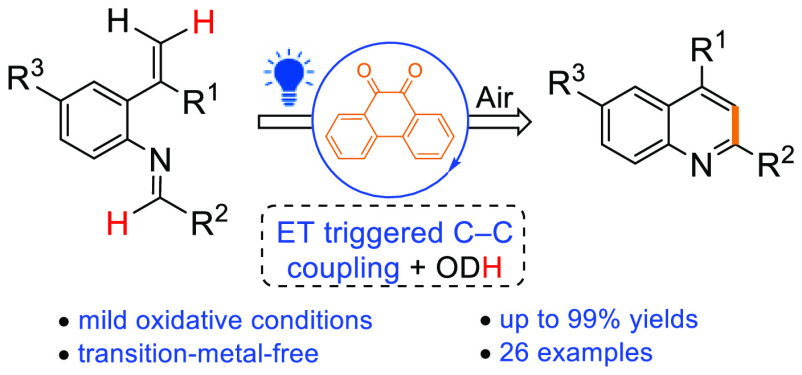

Visible-light-excited 9,10-phenanthrenequinone (PQ*) was used as a photocatalyst for the synthesis of polysubstituted quinolines via the electrocyclization of 2-vinylarylimines. Up to quantitative yields of 2,4-disubstituted quinolines were received after 1 h of excitation with blue LEDs at room temperature when MgCO_3_ was used as an additive in DCM. On the basis of experimental and DFT studies, we propose that PQ* induces one-electron oxidation of the imine substrate that triggers the electrocyclization mechanism.

Quinolines are an important class of N-heterocyclic aromatic compounds as they are ubiquitous structures in natural products and pharmaceuticals.^1a^ The selective functionalization of different positions of the quinoline ring is often hard to achieve and thus substituted quinolines are usually synthesized from precursors carrying functional groups placed at the desired positions.^1^ One classic example of such a reaction is electrocyclization, which is applicable for a wide range of starting materials to give access to polysubstituted quinolines (Scheme 1a). However, the main drawback of this protocol is the need for harsh reaction conditions, for instance, high temperatures or pressures,^2^ strong Lewis acid catalysts,^3^ or short wavelength UV lights.^4^ Yields typically vary from poor to good with long reaction times up to several days, while dihydroquinolines are often received as side products.

**Scheme 1:**
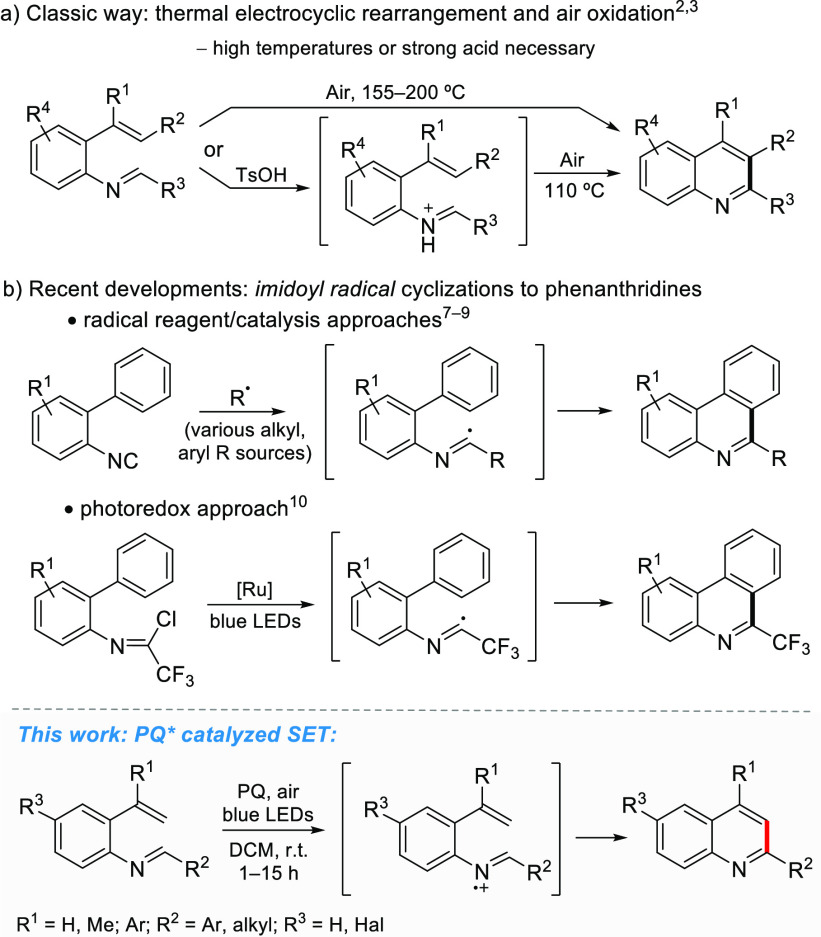
Electrocyclization and Radical Cyclization Synthetic Protocols to Access Quinoline Derivatives

Recent developments in methods to generate organic radicals have led to the rise of novel synthetic methodologies to perform organic transformations under mild reaction conditions.^5^ In particular, visible-light photoredox catalysis has proven to be a useful tool to achieve this goal.^6^ In the development of quinoline derivative synthesis, *in situ* created 2-imidoylbiphenyl radicals have provided access to phenanthridines via homolytic aromatic substitution (Scheme 1b). These radicals have been obtained from the reaction of isocyanides with alkyl or aryl radicals, which can be produced by various synthetic or catalytic protocols using, for example, aryl amines and *t*BuONO,^7^ boronic acids and Mn(acac)_3_,^8^ or the Togni reagent and Bu_4_NI.^9^ On the other hand, the photocatalyzed redox approach provides reductive access to the imidoyl radicals from 2,2,2-trifluoro-*N*-phenylacetimidoyl chlorides.^10^

In this study, our objective was to combine the simplicity of classic electrocyclization methods for quinoline synthesis with the efficiency and mildness of photoredox radical methods. For a range of pericyclic reactions, activation energies for radical cationic pathways remarkably lower than those for the corresponding neutral reactions have been reported.^11^

We envisioned that a strong oxidant could transform the imine to an iminium radical cation or a neutral radical that would spontaneously cyclize to dihydroquinoline and subsequently aromatize to quinoline. This scenario was further supported by our previous DFT computational results, which predicted low energy barriers for the radical-cation-mediated cyclization route of 2-vinylarylimines to dihydroquinolines.^12^

Quinones at their ground states have been frequently used as mediators in various oxidative organic transformations.^13^ This is in contrast with the relatively low number of studies reported where quinones were studied as photocatalysts, even though their excited states are known to exhibit high oxidation potentials.^14^ An exception to this is anthraquinone as a photo-oxidant. Besides being reported as a competent electron and hydrogen atom acceptor, it also often acts as a catalyst to produce reactive oxygen species.^15^ For the current study, the latter feature would be unsuitable, as our interest was to discover catalysts able to oxidize the imine substrates via either single electron transfer (SET) or hydrogen atom transfer (HAT) and be regenerated by atmospheric oxygen afterward.^6b,14^ In this frame, visible-light-excited 9,10-phenanthrenequinone (PQ) raised our attention as a potential photocatalyst.

In 2000, Fukuzumi and co-workers reported the pioneering catalytic study on visible-light-activated PQ as a photo-oxidant, where benzylic alcohols were oxidized to the corresponding aldehydes utilizing molecular oxygen as the terminal oxidant.^16^ In this case, it was concluded that the oxidation step proceeds via a SET mechanism. Since this study, PQ has been reported as a photocatalyst only in a few other studies. Kumar’s team developed a PQ-catalyzed cascade trifluoromethylation and oxidation of 1,6-enynes in which excited-state PQ (PQ*) performs the SET activation of Langlois’ reagent (CF_3_SO_2_Na).^17^ Vila and co-workers observed that PQ* could work as a SET photoredox catalyst in the oxidation of benzoxazinones to iminium cations to couple with indoles in a Friedel–Crafts reaction.^18^ Wang and co-workers applied PQ* for HAT catalysis to convert aldehydes to acyl radicals, which then reacted with thiosulfonates to access thioesters.^19^ Recently, Xia and co-workers used a similar procedure to oxidize aldehydes to acyl radicals, which were further utilized in a Pd catalytic cycle.^20^

We commenced the study of the PQ*-catalyzed cyclization of imine **1a** using a 20 mol % PQ loading and blue LED irradiation in acetonitrile, receiving a promising yield of quinoline **2a** (43%). Solvent screening exposed DCM as being superior affording fast reactivity, while other solvents (Table 1 and Table S1) resulted in poorer yields even after prolonged reaction times. Trace amounts of water seemed to disturb the reaction significantly, causing imine **1a** to hydrolyze to the corresponding aniline and aldehyde. First, we tried to suppress the imine hydrolysis using drying additives. The tested molecular sieves and sulfates, being slightly acidic or neutral in nature, caused only a slight improvement in the yield of **2a** and the hydrolysis of **1a** still took place (Table S3). Our next attempt was to inhibit the imine hydrolysis with carbonate additives as slightly basic drying agents. The yields improved significantly with all the carbonates tested, and only a trace amount of benzaldehyde was detected. Notably, the highest yield was achieved with MgCO_3_*n*-hydrate, indicating that its role was to act as a base rather than as a drying agent. Regardless, all the additives were only sparingly soluble in DCM. We measured the UV–vis spectra of PQ, **1a**, and their mixture both with and without MgCO_3_ (Figure S4) and observed that the additive caused no shifts in the absorbance. This indicates that the carbonate does not have a strong interaction with either the substrate or the catalyst. Hence, we presume that its key effect is to stabilize imines as a non-nucleophilic base.^21^ The compatibility of various carbonates with quinone photocatalysts has been shown in previous studies.^14a,19^ Additionally, other tested photocatalysts (anthraquinone, eosin Y, and Acr^+^–Mes ClO_4_^–^) did not provide substantial reactivity (Table S5).

With the optimized reaction conditions in hand, we moved on to study the cyclization scope by varying the substitution on the R^1^–R^3^ positions, probing electronic effects (R^1^–R^3^) and the aromatic or aliphatic substituent effect (R^1^ and R^2^). Both 4-methylquinolines **2a**–**2h** and 4-phenylquinolines **2t**–**2z** were obtained in excellent yields in 1–3 h (Scheme 2). All quinolines bearing 4- and 3-substituted aryl groups as R^2^ substituents were achieved in very good yields, but when the imine substrate had a 2-aryl substituent on the same position the yields of quinolines **2i** and **2j** were notably lower even though the reaction time was increased to 15 h. A similar substrate **1ad** with a 2-nitroaryl substituent was not able to undergo cyclization and instead resulted in a complicated mixture of unknown side products.

**Scheme 2:**
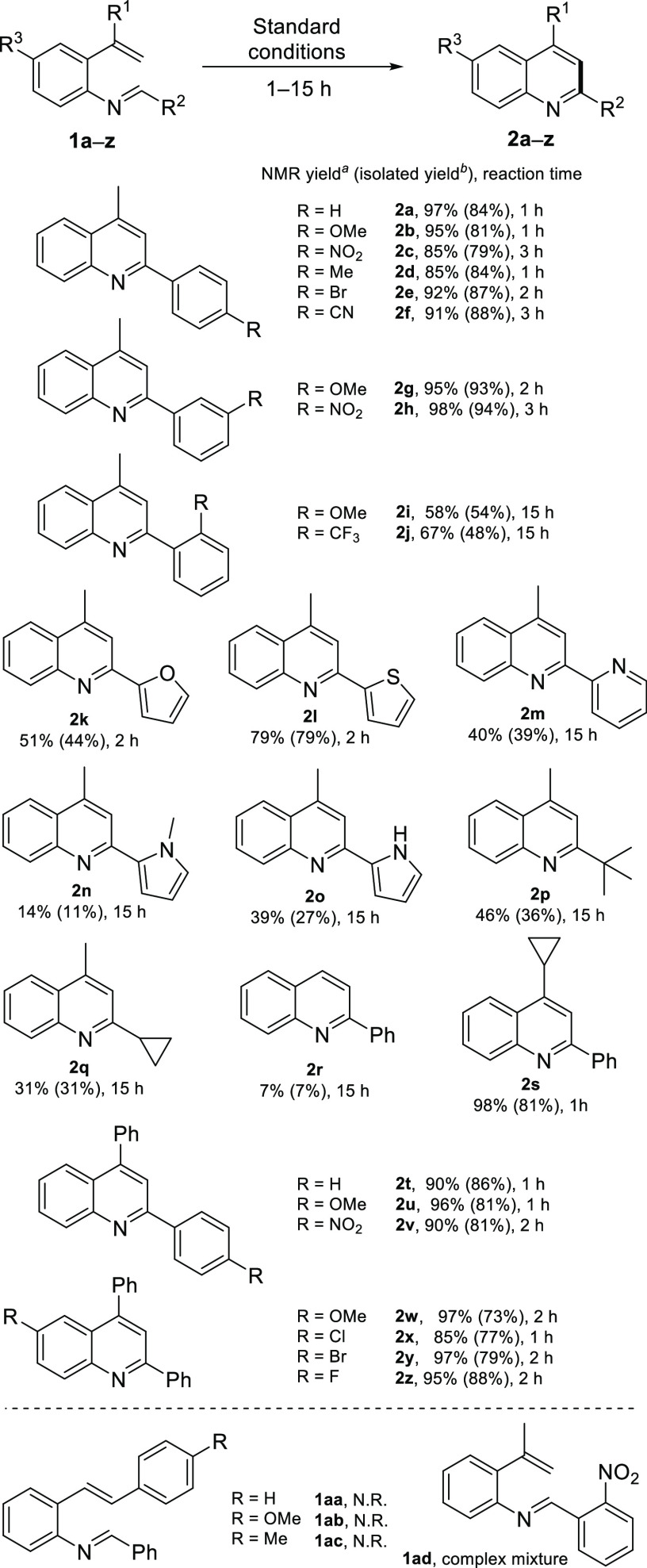
Reaction Scope Study 1,3,5-Trimethoxybenzene was used as an internal standard After SiO_2_ chromatography.

The synthesis of 4-methylquinolines **2k**–**2o** with 2-heteroaryl substituents gave varying results. The reaction with imines **1k** and **1l** was stopped after 2 h, as the appearance of polymer-like side products was detected during the ^1^H NMR follow-up. Besides, the reactions with *N*-heteroaryl substituents did not proceed cleanly, as only 11–39% yields of quinolines **2m**–**2o** were isolated after 15 h. The received lower yields were attributed to the slower conversion of the imines to quinolines as the imine hydrolysis started to compete with the desired reaction. Similarly, hydrolysis was also a problem with imines **1p** and **1q** with aliphatic substituents on the R^2^ position, resulting in 31–36% isolated yields of the corresponding quinolines. The instability of these imines was probably due to the lack of the stabilizing conjugation over the imine bond.

Our attempt to further expand the reaction scope to 2,3-diarylquinolines was unsuccessful, as styryl-derivatized imines **1aa**–**1ac** were completely unreactive even when the reaction time was extended to 15 h. In similar fashion, substrate **1r** lacking an R^1^ substituent cyclized rather sluggishly into 2-phenylquinoline **2r**, giving only a poor yield of 7% after 15 h. Other unsuitable substrates are listed in the SI. To explain this behavior, we started to investigate the reaction mechanism.

At first, we carried out the reaction with imine **1a** under the standard conditions in the presence of varying amount of TEMPO as a radical scavenger. With 2 equiv of TEMPO, the isolated yield of **2a** decreased (from 84% to 35%), and a TEMPO-trapped product **3** was identified and isolated with a 16% yield (Scheme 3). Additionally, a trace amount of amide **4** was obtained. Increasing the loading of TEMPO to 5 equiv lowered the quinoline yield to 13%. Moreover, only 4% of adduct **3** could be detected, while the amount of amide **4** increased to 10%, indicating that its formation was associated with the TEMPO additive. The decreased yield suggested a radical character for the reaction, whereas the formation of the TEMPO adduct indicated the presence of a stabilized radical on the 4-position of the quinoline. The presence of long-living radical intermediates was further evaluated by employing cyclopropyl radical clocks with substrates **1q** and **1s**, which did not deliver any ring opening products.

**Scheme 3:**
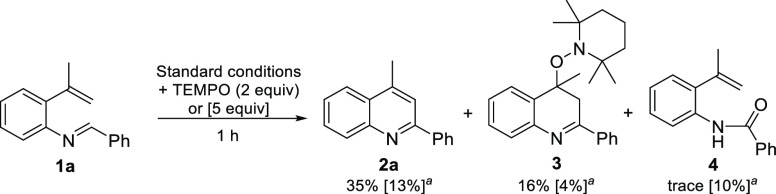
Reaction with TEMPO Radical Scavenger NMR yields with 5 equiv of TEMPO.

Next, we studied the kinetic isotopic effect with imine **1a** and its deuterated derivative **1ah** (Figure S3). The isotopic effect did not change the reaction rates, suggesting that the imine proton or hydrogen transfer was not connected to the rate limiting step in the reaction pathway.

To gain theoretical insight into the possible mechanistic routes, we performed computational studies of the initial steps of both HAT and electron transfer–proton transfer (ET-PT) routes of selected imine substrates at the DFT level.^22^ Interestingly, the HAT mechanism involving imidoyl radicals resulted in very low cyclization barriers (1.5–8.2 kcal/mol) and highly exergonic intermediate energies (−29.6 to −39.3 kcal/mol) for a diverse set of substrates **1a**, **1b**, **1f**, **1r**, and **1aa** (Figure 1), which was a mismatch with the experimental observation of the reactivities of **1r** and **1aa**. Instead, the computed radical cation cyclization barriers were 10.5, 11.4, and 12.2 kcal/mol for the smoothly reactive substrates **1f**, **1a**, and **1b**, respectively. Besides, the higher cyclization barriers and endergonic energies of the cyclized intermediates of **1r** and **1aa** explain well their poor reactivities considering that these steps are reversible.

**Figure 1:**
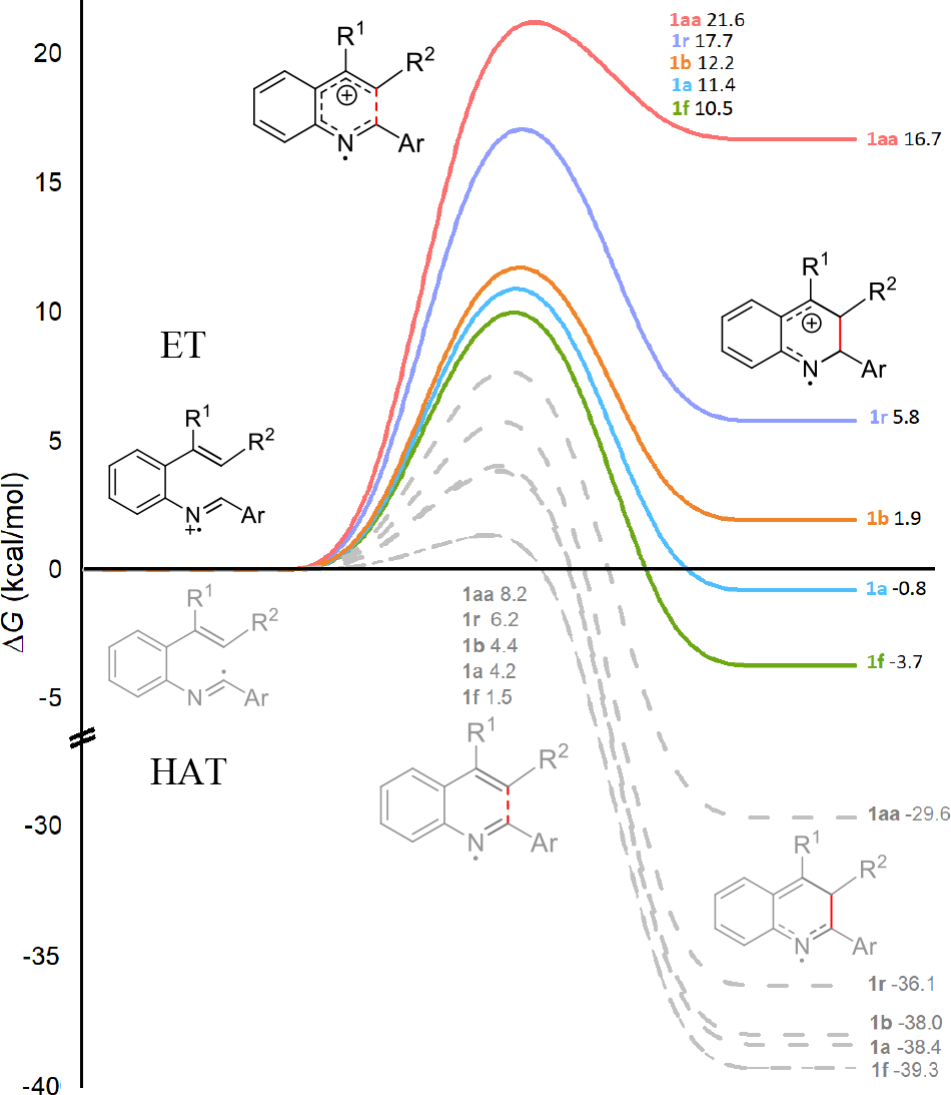
Computed initial steps in HAT and ET-PT pathways.

Computed p*K*_a_s of the iminium radical cation **INT1** and the cyclized intermediate **INT2** (Scheme 4) resulted in values of 45–51 and 17–20 in DCM, respectively (Table S10). This indicates the dramatically increased acidity for the proton laying on the 2-position of **INT2**, favoring the C–C coupling step before the deprotonation event in the mechanistic route.^23^

**Scheme 4:**
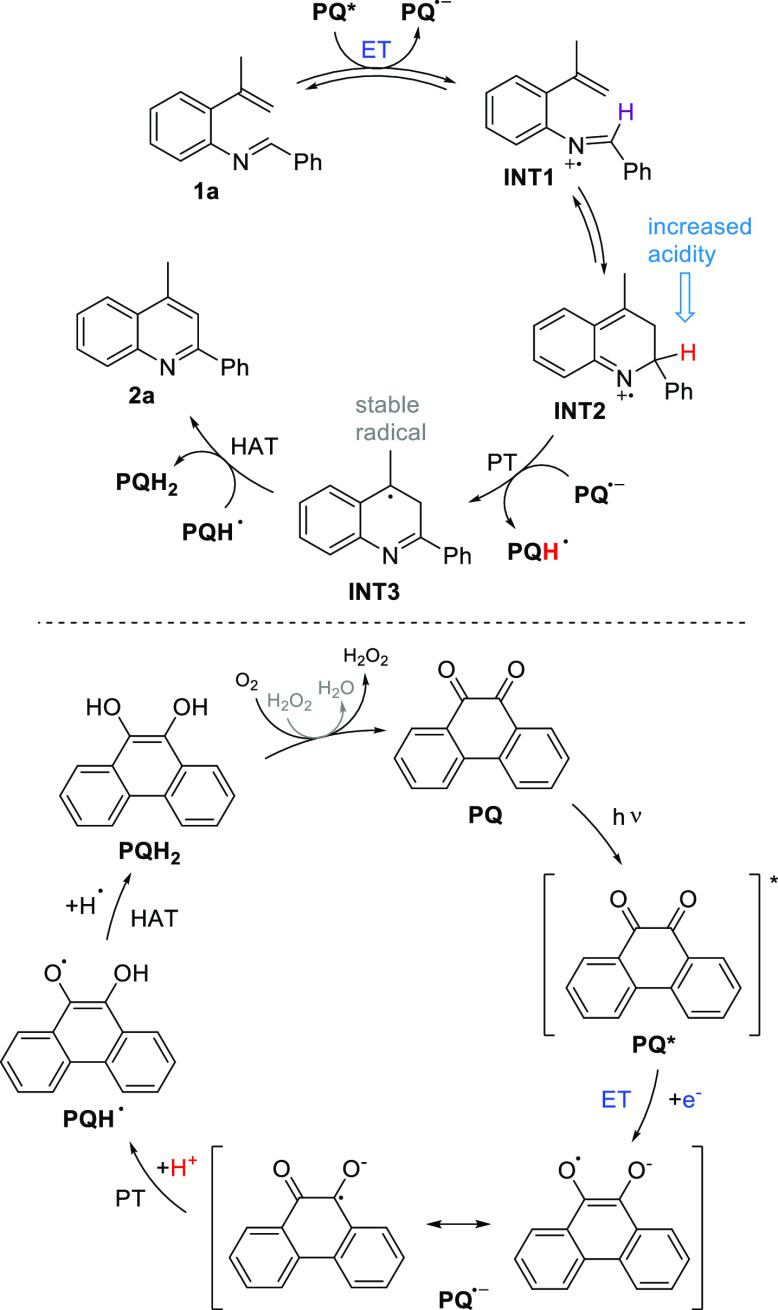
Proposed Reaction Mechanism

The computed oxidation potentials (*E*_1/2_^ox^) for the studied imine substrates are in the range of 1.1–1.5 V (versus SCE), being lower than the reported excited state reduction potential *E*_1/2_^red^* of PQ (^3^PQ*/**PQ̇**^**–**^ = 1.6 V vs SCE).^16,17^ This suggests that PQ* is a strong enough oxidant for the imines to operate with the SET mechanism (SI).

Based on the experimental reactivity, TEMPO-trapped intermediates, and the computational studies, we propose a reaction mechanism for the electrocyclization (Scheme 4). After the visible-light excitation of PQ to its excited triplet state (PQ*),^16^ it induces SET from **1a**, generating a radical on the nitrogen atom. The radical cation intermediate **INT1** next cyclizes to the dihydroquinoline cation radical **INT2** carrying an acidic proton, which is removed by the radical anion **PQ**^**•–**^ resulting in two neutral radical species, **PQH**^**•**^ and **INT3**. The formation of **INT3** is supported by the TEMPO-trapped product **3** (Scheme 3). In the final stage, HAT yields phenanthrene-9,10-diol **PQH**_**2**_ and quinoline **2a**. The reduced **PQH**_**2**_ is then readily oxidized back to PQ by molecular oxygen. The key role of oxygen was proven by conducting the standard cyclization reaction under an argon atmosphere (Table 1, entry 6), where only a stoichiometric amount of **2a** was obtained.

**Table 1:**
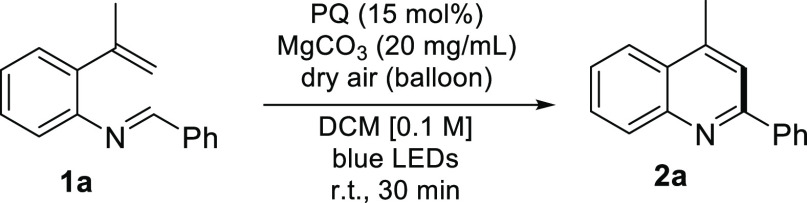
Effect of Deviation from the Standard Reaction Conditions^a^

entry	deviation from the standard conditions	yield (%)^b^
1	none	97
2	1 h reaction time	97 (84)^c^
3	no light	0
4	no photocatalyst	0
5	no MgCO_3_	45
6	Ar atmosphere	15
7	O_2_ atmosphere	84
8	MeCN as solvent	44
9	toluene as solvent	51
10	EtOAc as solvent	48
11	0.12 M concentration	90
12	0.08 M concentration	80
13	MgSO_4_ as additive^d^^,^^e^	54
14	K_2_CO_3_ as additive^d^^,^^e^	83
15	10 mg/mL MgCO_3_^d^^,^^e^	94
16	40 mg/mL MgCO_3_^d^^,^^e^	96
17	10 mol % PQ^d^	90
18	20 mol % PQ^d^	97
19	1 mmol scale, 21 h reaction time	78^c^

a Full reaction optimization in Tables S1–S7.

b NMR yield using 1,3,5-trimethoxybenzene as an internal standard.

c Isolated yield after SiO_2_ chromatography.

d 1 h reaction time, O_2_ atmosphere.

e 20 mol % PQ.

In summary, we have developed a mild and efficient method to selectively synthesize polysubstituted quinolines using visible-light-excited PQ as a photocatalyst. The performed preliminary mechanistic studies suggest that excited-state PQ oxidizes imines to iminium cation radicals that trigger the cyclization step, leading to the subsequent deprotonation and dehydrogenation steps and the final formation of quinolines. Time-resolved photophysical studies are underway in our laboratory to acquire more detailed information on the reaction mechanism.

## Supporting Information Available

Experimental procedures, full reaction optimization, ^1^H NMR reaction monitoring, mechanistic studies, computational procedures and details, and ^1^H and ^13^C NMR spectra for all compounds (PDF)
