# Supplementary material for: Phenanthrenequinone-Sensitized Photocatalytic Synthesis of Polysubstituted Quinolines from 2-Vinylarylimines
Source: Org Lett. 2021 Dec 20;24(1):274–8. doi: 10.1021/acs.orglett.1c03934 (PMC8762703; doi:10.1021/acs.orglett.1c03934)

Supporting information

Phenanthrenequinone-Sensitized Photocatalytic Synthesis of Polysubstituted Quinolines from 2-Vinylarylimines

Juulia Talvitie, Iida Alanko, Evgeny Bulatov, Juho Koivula, Topias Pöllänen, and Juho Helaja*

Department of Chemistry, University of Helsinki, A. I. Virtasen aukio 1, 00014 Helsinki, Finland

*Corresponding author

- E-mail: juho.helaja@helsinki.fi

Contents

General Information 3

Synthesis and characterization of the starting materials 5

General procedure A 5

General procedure B 5

Unsuitable substrates 15

Optimization of reaction conditions 18

Synthesis and characterization of quinolines 2a–2z 20

General procedure C 20

Mechanistic studies 29

Kinetics experiment 29

UV–vis spectroscopic measurements 30

Attempted detection of H_2O_2 30

Radical trapping by TEMPO 31

Computational studies 32

Bibliography 40

Copies of NMR spectra 43

General Information

All reagents and solvents were purchased from commercial sources (Acros Organics, Alfa Aesar, Fluorochem, J.T.Baker, Merck, Sigma-Aldrich, TCI and VWR) and used without further purification. Magnesium carbonate used was *n*-hydrate. Its most common formula is $(MgCO_3)_4{\cdot}Mg(OH)_2{\cdot}4{-}5H_2O$[1] so its molecular weight was approximated to be 95.50 g/mol. Dichloromethane, tetrahydrofuran and toluene were dried over 4Å molecular sieves prior to use. Glassware was stored in ambient conditions before use. NMR spectra were recorded at 25 °C on Bruker Avance Neo 400 MHz [400.15 MHz] spectrometer. Acetone-d_6 and TMS-containing $CDCl_3$ were used as deuterated solvents with solvent signals (2.05, 29.84 ppm and 0.00 (from TMS), 77.16 ppm, respectively) as references to chemical shifts. In ^{19}F NMR measurements, $CFCl_3$ was used as a reference. NMR yields were calculated with 1,3,5-trimethoxybenzene as an internal standard. UV–vis spectra were measured with Varian Cary 50 UV–Visible spectrophotometer using standard 10 mm glass cuvettes. Thin-layer chromatography (TLC) was conducted using silica gel GF254 with fluorescence indicator (254 nm). Chromatographic separations were performed with VWR silica gel (230-300 mesh). High-resolution mass spectra (HRMS) were obtained on a Jeol MStation JMS-700 (EI) instrument with a quadrupole mass analyzer. The photoreaction setup is shown in Figure S1. The light source was 3 x 3W ProLight Opto royal blue (455–460 nm) LEDs which were positioned on the bottom of the reaction vial at a 4 mm distance. The UV–vis emission spectrum of the LEDs is shown in Figure S2. No filters were used. The measured light power of 3 LEDs was 1.03 W. The reaction stand was a custom-made aluminum block with build-in water cooling.

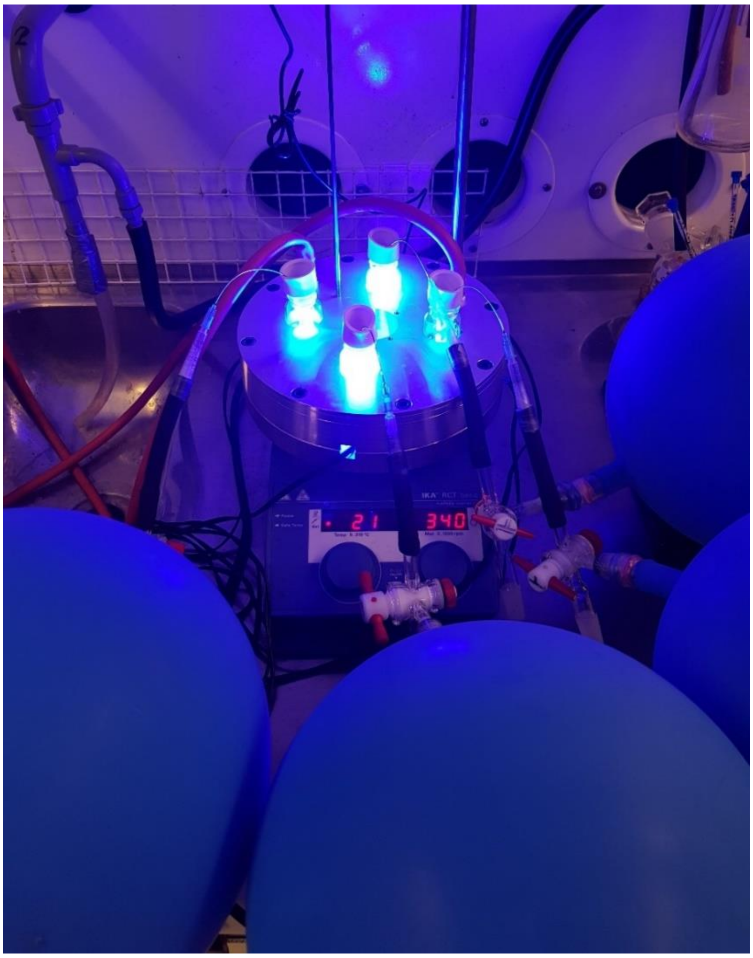

Figure S1. The experimental setup of the photoreactions.

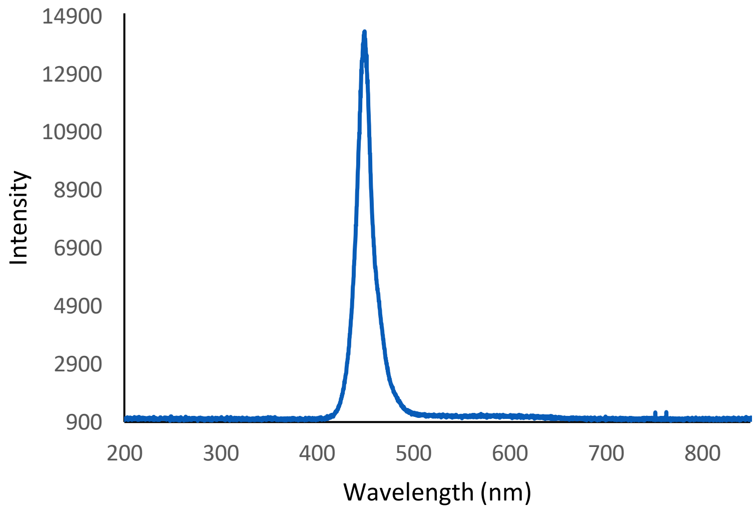

Figure S2. UV–vis emission spectrum of the used LEDs.

Synthesis and characterization of the starting materials

General procedure A

The synthesis of imines was performed following the procedure modified from literature.[2] A two-neck flask equipped with a stirrer bar and a condenser was loaded with drying agent (excess amount). The flask was evacuated and backfilled with argon three times. Anhydrous toluene (to get the concentration of aniline to 0.2 M), respective aniline (1 equiv) and respective aldehyde (1 equiv) were added to the suspension via septum and the mixture was bubbled with argon for 30 min. The reaction mixture was refluxed on an oil bath overnight while stirring. After cooling down to r.t., the mixture was filtered through Celite and washed with EtOAc. The solvents were evaporated from the filtrate on rotary evaporator and the product was purified with flash chromatography on silica gel.

General procedure B

The synthesis of imines was performed following the procedure modified from literature.[3] A two-neck flask equipped with a stirrer bar was evacuated and backfilled with argon three times. Respective aniline (1 equiv) and respective aldehyde (1 equiv) were added to the flask via septum and the reaction mixture was stirred at the specified temperature for the time needed. The mixture was diluted with DCM and Na_2SO_4 was added to remove water. The mixture was filtered and solvents were evaporated on rotary evaporator. The crude product was dried under vacuum and used without further purification.

1-Phenyl-*N*-(2-(prop-1-en-2-yl)phenyl)methanimine (1a)

The product was prepared from 2-(prop-1-en-2-yl)aniline (0.62 mL, 4.6 mmol) and benzaldehyde (0.46 mL, 4.5 mmol) according to the general procedure A. Na_2SO_4 (7.0 g, 49 mmol) was used as drying agent. The product was isolated with flash chromatography (silica gel, *n*-Hex:Et_3N 100:3) as a yellow oil (910.6 mg, 4.1 mmol, 91%).

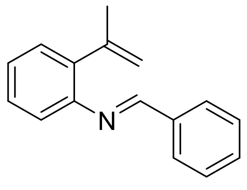

^{1}H NMR (400 MHz, $CDCl_3$) δ 8.37 (s, 1H), 7.92 – 7.87 (m, 2H), 7.50 – 7.45 (m, 3H), 7.33 – 7.26 (m, 2H), 7.18 (td, *J* = 7.5, 1.3 Hz, 1H), 6.95 (ddd, *J* = 7.6, 1.3, 0.5 Hz, 1H), 5.17 (dq, *J* = 2.2, 1.5 Hz, 1H), 5.02 (dq, *J* = 1.8, 0.9 Hz, 1H), 2.15 (dd, *J* = 1.5, 0.9 Hz, 3H).

^{13}C NMR (101 MHz, $CDCl_3$) δ 160.0, 150.0, 144.9, 137.2, 136.6, 131.4, 128.97, 128.95, 128.9, 128.2, 125.6, 118.8, 116.4, 23.9.

The characterization data was in agreement with the previous literature.[2]

1-(4-Methoxyphenyl)-*N*-(2-(prop-1-en-2-yl)phenyl)methanimine (1b)

The product was prepared from 2-(prop-1-en-2-yl)aniline (0.50 mL, 3.7 mmol) and *p*-anisaldehyde (0.44 mL, 3.6 mmol) according to the general procedure A. Na_2SO_4 (5.2 g, 37 mmol) was used as a drying agent. The product was isolated with flash chromatography (silica gel, *n*-Hex:Et_3N 100:3) as a yellow oil (762.2 mg, 3.0 mmol, 84%).

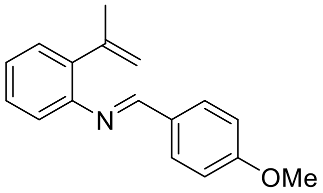

^{1}H NMR (400 MHz, $CDCl_3$) δ 8.29 (s, 1H), 7.86 – 7.81 (m, 2H), 7.31 – 7.25 (m, 2H), 7.15 (td, *J* = 7.5, 1.3 Hz, 1H), 7.01 – 6.96 (m, 2H), 6.93 (ddd, *J* = 7.6, 1.4, 0.5 Hz, 1H), 5.15 (dt, *J* = 3.0, 1.5 Hz, 1H), 5.02 (dd, *J* = 2.1, 0.9 Hz, 1H), 3.87 (s, 3H), 2.14 (dd, *J* = 1.5, 0.8 Hz, 3H).

^{13}C NMR (101 MHz, $CDCl_3$) δ 162.3, 159.2, 150.3, 145.0, 137.1, 130.6, 129.7, 128.9, 128.2, 125.3, 118.9, 116.2, 114.3, 55.6, 23.9.

The characterization data was in agreement with the previous literature.[4]

1-(4-Nitrophenyl)-*N*-(2-(prop-1-en-2-yl)phenyl)methanimine (1c)

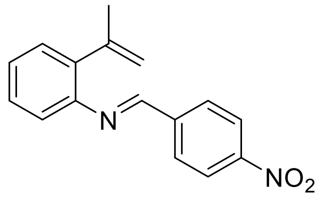

The synthesis was performed following the procedure modified from literature.[5] A two-neck flask equipped with a stirrer bar was loaded with Na_2SO_4 (2.5 g, 18 mmol), $MgSO_4$ (2.5 g, 21 mmol), 4-nitrobenzaldehyde (556.0 mg, 3.7 mmol), anhydrous THF (15 mL) and 2-(prop-1-en-2-yl)aniline (0.50 mL, 3.7 mmol). The suspension was bubbled with argon for 30 min and then stirred at r.t. overnight. The mixture was filtered through Celite and washed with EtOAc. The solvents were evaporated from the filtrate on rotary evaporator and the crude was stored in a fridge until it crystallized. The recrystallization from EtOH afforded the product as a yellow solid (426.1 mg, 1.6 mmol, 43%).

^{1}H NMR (400 MHz, $CDCl_3$) δ 8.47 (s, 1H), 8.36 – 8.30 (m, 2H), 8.09 – 8.03 (m, 2H), 7.36 – 7.28 (m, 2H), 7.27 – 7.21 (m, 2H), 6.99 (ddd, *J* = 7.6, 1.3, 0.5 Hz, 1H), 5.20 (dt, *J* = 3.0, 1.5 Hz, 1H), 4.99 (dd, *J* = 2.0, 0.9 Hz, 1H), 2.15 (dd, *J* = 1.5, 0.9 Hz, 3H).

^{13}C NMR (101 MHz, $CDCl_3$) δ 157.2, 149.4, 148.9, 144.4, 141.9, 137.8, 129.5, 129.2, 128.3, 126.8, 124.2, 118.3, 116.8, 23.9.

The characterization data was in agreement with the previous literature.[4]

***N*-(2-(Prop-1-en-2-yl)phenyl)-1-(*p*-tolyl)methanimine (1d)**

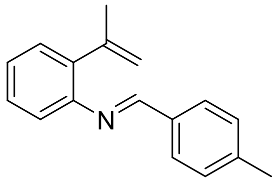

The product was prepared from 2-(prop-1-en-2-yl)aniline (0.39 mL, 2.8 mmol) and *p*-tolualdehyde (0.33 mL, 2.8 mmol) according to the general procedure A. Na_2SO_4 (2.5 g, 18 mmol) and $MgSO_4$ (2.5 g, 21 mmol) were used as drying agents. The product was isolated with flash chromatography (silica gel, *n*-Hex:Et_3N 100:2) as a pale yellow oil (296.4 mg, 1.3 mmol, 44%).

^{1}H NMR (400 MHz, $CDCl_3$) δ 8.32 (s, 1H), 7.78 (d, *J* = 8.1 Hz, 2H), 7.32 – 7.24 (m, 4H), 7.16 (td, *J* = 7.5, 1.3 Hz, 1H), 6.96 – 6.91 (m, 1H), 5.15 (dt, *J* = 3.0, 1.5 Hz, 1H), 5.02 (dq, *J* = 1.8, 0.9 Hz, 1H), 2.42 (s, 3H), 2.14 (dd, *J* = 1.5, 0.9 Hz, 3H).

^{13}C NMR (101 MHz, $CDCl_3$) δ 159.9 (CH), 150.2 (C), 144.9 (C), 141.9 (C), 137.1 (C), 134.1 (C), 129.6 (CH), 128.9 (CH), 128.2 (CH), 125.4 (CH), 118.9 (CH), 116.3 (CH_2), 23.9 (CH_3), 21.8 (CH_3).

HRMS (EI) *m/z*: [M^+] calcd for $C_{17}H_{17}N$ 235.1361, found: 235.1363.

1-(4-Bromophenyl)-*N*-(2-(prop-1-en-2-yl)phenyl)methanimine (1e)

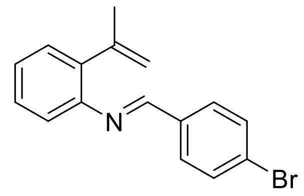

The product was prepared from 2-(prop-1-en-2-yl)aniline (0.39 mL, 2.8 mmol) and 4-bromobenzaldehyde (520.4 mg, 2.8 mmol) according to the general procedure A. The solid aldehyde was added to the flask together with drying agent, which was Na_2SO_4 (4.0 g, 28 mmol). According to ^{1}H NMR of the reaction mixture, the reaction had not proceeded enough in one night, so $MgSO_4$ (4.0 g, 33 mmol) was added and refluxing was continued for another night. The product was isolated with flash chromatography (silica gel, *n*-Hex:Et_3N 100:3) as a yellow oil (622.6 mg, 2.1 mmol, 74%).

^{1}H NMR (400 MHz, $CDCl_3$) δ 8.32 (s, 1H), 7.78 – 7.73 (m, 2H), 7.63 – 7.58 (m, 2H), 7.32 – 7.26 (m, 2H), 7.19 (td, *J* = 7.5, 1.3 Hz, 1H), 6.96 – 6.92 (m, 1H), 5.17 (dt, *J* = 3.0, 1.5 Hz, 1H), 5.00 (dd, *J* = 2.1, 0.9 Hz, 1H), 2.13 (dd, *J* = 1.5, 0.8 Hz, 3H).

^{13}C NMR (101 MHz, $CDCl_3$) δ 158.6 (CH), 149.6 (C), 144.7 (C), 137.3 (C), 135.5 (C), 132.2 (CH), 130.3 (CH), 129.0 (CH), 128.3 (CH), 125.94 (C), 125.92 (CH), 118.6 (CH), 116.5 (CH_2), 23.9 (CH_3).

HRMS (EI) *m/z*: [M^+] calcd for $C_{16}H_{14}BrN$ 299.0310, found: 299.0312.

1-(4-Cyanophenyl)-*N*-(2-(prop-1-en-2-yl)phenyl)methanimine (1f)

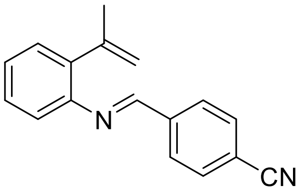

The product was prepared from 2-(prop-1-en-2-yl)aniline (0.28 mL, 2.1 mmol) and 4-cyanobenzaldehyde (272.0 mg, 2.1 mmol) according to the general procedure A. Na_2SO_4 (2.0 g, 14 mmol) and $MgSO_4$ (2.0 g, 17 mmol) were used as drying agents. The reaction was stirred at r.t. for 4 h and then at 90 °C overnight. The product was isolated with flash chromatography (silica gel, *n*-Hex:Et_3N 100:3) as a yellow waxy solid (333.0 mg, 1.4 mmol, 66%).

^{1}H NMR (400 MHz, $CDCl_3$) δ 8.41 (s, 1H), 8.02 – 7.96 (m, 2H), 7.78 – 7.68 (m, 2H), 7.34 – 7.27 (m, 2H), 7.23 (td, *J* = 7.4, 1.3 Hz, 1H), 6.96 (dd, *J* = 7.8, 1.3 Hz, 1H), 5.19 (p, *J* = 1.5 Hz, 1H), 4.98 (dq, *J* = 1.9, 0.9 Hz, 1H), 2.14 (dd, *J* = 1.5, 0.9 Hz, 3H).

^{13}C NMR (101 MHz, $CDCl_3$) δ 157.6 (CH), 148.9 (C), 144.5 (C), 140.3 (C), 137.7 (C), 132.7 (CH), 129.23 (CH), 129.16 (CH), 128.3 (CH), 126.6 (CH), 118.6 (C), 118.3 (CH), 116.7 (CH_2), 114.5 (C), 23.9 (CH_3).

HRMS (EI) *m/z*: [M^+] calcd for $C_{17}H_{14}N_2$ 246.1157, found: 246.1150.

1-(3-Methoxyphenyl)-*N*-(2-(prop-1-en-2-yl)phenyl)methanimine (1g)

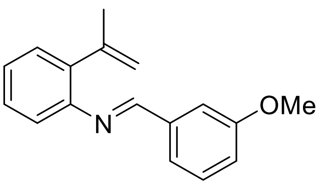

The product was prepared from 2-(prop-1-en-2-yl)aniline (0.14 mL, 1.0 mmol) and *m*-anisaldehyde (0.12 mL, 1.0 mmol) according to the general procedure A. Na_2SO_4 (0.63 g, 4.4 mmol) and $MgSO_4$ (0.63 g, 5.2 mmol) were used as drying agents. The product was isolated with flash chromatography (silica gel, *n*-Hex:Et_3N 100:2) as a yellow oil (142.9 mg, 0.57 mmol, 57%).

^{1}H NMR (400 MHz, $CDCl_3$) δ 8.33 (s, 1H), 7.50 (dd, *J* = 2.7, 1.3 Hz, 1H), 7.43 – 7.35 (m, 2H), 7.33 – 7.26 (m, 2H), 7.18 (td, *J* = 7.5, 1.3 Hz, 1H), 7.04 (ddd, *J* = 7.7, 2.7, 1.5 Hz, 1H), 6.94 (ddd, *J* = 7.7, 1.3, 0.5 Hz, 1H), 5.16 (dt, *J* = 3.0, 1.5 Hz, 1H), 5.02 (dd, *J* = 2.1, 0.9 Hz, 1H), 3.88 (s, 3H), 2.15 (dd, *J* = 1.5, 0.8 Hz, 3H).

^{13}C NMR (101 MHz, $CDCl_3$) δ 160.1 (C), 159.9 (CH), 149.9 (C), 144.8 (C), 138.0 (C), 137.1 (C), 129.9 (CH), 129.0 (CH), 128.2 (CH), 125.7 (CH), 122.3 (CH), 118.8 (CH), 118.0 (CH), 116.5 (CH_2), 112.4 (CH), 55.6 (CH_3), 23.8 (CH_3).

HRMS (EI) *m/z*: [M$^+$] calcd for $C_{17}H_{17}NO$ 251.1310, found: 251.1316.

1-(3-Nitrophenyl)-*N*-(2-(prop-1-en-2-yl)phenyl)methanimine (1h)

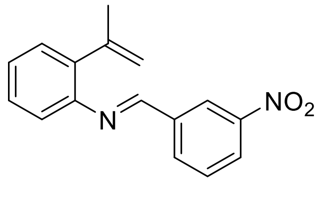

The product was prepared from 2-(prop-1-en-2-yl)aniline (0.54 mL, 4.0 mmol) and 3-nitrobenzaldehyde (604.1 mg, 4.0 mmol) according to the general procedure A. The solid aldehyde was added to the flask together with drying agents, which were Na_2SO_4 (2.5 g, 18 mmol) and $MgSO_4$ (2.5 g, 21 mmol). The product was isolated with flash chromatography (silica gel, *n*-Hex:Et_3N 100:2) as a yellow oil (366.6 mg, 1.4 mmol, 34%).

^{1}H NMR (400 MHz, $CDCl_3$) δ 8.69 (t, *J* = 2.0 Hz, 1H), 8.46 (s, 1H), 8.33 (ddd, *J* = 8.2, 2.3, 1.1 Hz, 1H), 8.26 (dt, *J* = 7.7, 1.4 Hz, 1H), 7.70 – 7.63 (m, 1H), 7.36 – 7.28 (m, 2H), 7.23 (td, *J* = 7.5, 1.3 Hz, 1H), 6.98 (ddd, *J* = 7.5, 1.3, 0.6 Hz, 1H), 5.19 (dt, *J* = 3.0, 1.5 Hz, 1H), 5.00 (dd, *J* = 2.0, 0.9 Hz, 1H), 2.15 (dd, *J* = 1.5, 0.9 Hz, 3H).

^{13}C NMR (101 MHz, $CDCl_3$) δ 157.1 (CH), 148.9 (C), 148.8 (C), 144.5 (C), 138.2 (C), 137.6 (C), 134.1 (CH), 130.0 (CH), 129.2 (CH), 128.3 (CH), 126.5 (CH), 125.7 (CH), 123.7 (CH), 118.5 (CH), 116.8 (CH_2), 23.9 (CH_3).

HRMS (EI) *m/z*: [M$^+$] calcd for $C_{16}H_{14}N_2O_2$ 266.1055, found: 266.1061.

1-(2-Methylphenyl)-*N*-(2-(prop-1-en-2-yl)phenyl)methanimine (1i)

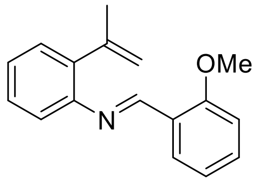

The product was prepared from 2-(prop-1-en-2-yl)aniline (0.54 mL, 4.0 mmol) and *o*-anisaldehyde (547.2 mg, 4.0 mmol) according to the general procedure A. The solid aldehyde was added to the flask together with drying agents, which were Na_2SO_4 (2.5 g, 18 mmol) and $MgSO_4$ (2.5 g, 21 mmol). The product was isolated with flash chromatography (silica gel, *n*-Hex:Et_3N 100:2) as a yellow oil (534.2 mg, 2.1 mmol, 53%). The purity of the product could not be improved despite several subsequent purification attempts.

^{1}H NMR (400 MHz, $CDCl_3$) δ 8.82 (t, *J* = 0.5 Hz, 1H), 8.15 (dd, *J* = 7.7, 1.8 Hz, 1H), 7.43 (ddd, *J* = 8.3, 7.3, 1.8 Hz, 1H), 7.31 – 7.26 (m, 2H), 7.16 (td, *J* = 7.5, 1.3 Hz, 1H), 7.04 (tt, *J* = 7.5, 0.9 Hz, 1H), 6.98 – 6.93 (m, 2H), 5.16 (dt, *J* = 3.0, 1.5 Hz, 1H), 5.01 (dq, *J* = 1.8, 0.9 Hz, 1H), 3.89 (s, 3H), 2.15 (dd, *J* = 1.5, 0.9 Hz, 3H).

^{13}C NMR (101 MHz, $CDCl_3$) δ 159.6, 155.9, 150.8, 145.0, 137.3, 132.7, 128.8, 128.2, 127.8, 125.4, 125.1, 121.0, 119.1, 116.3, 111.3, 55.7, 24.0.

The characterization data was in agreement with the previous literature.[4]

***N*-(2-(Prop-1-en-2-yl)phenyl)-1-(2-(trifluoromethyl)phenyl)methanimine (1j)**

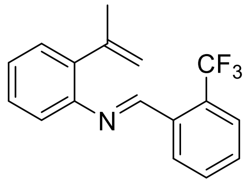

The product was prepared from 2-(prop-1-en-2-yl)aniline (0.14 mL, 1.0 mmol) and 2-(trifluoromethyl)benzaldehyde (0.13 mL, 1.0 mmol) according to the general procedure A. Na_2SO_4 (0.63 g, 4.4 mmol) and $MgSO_4$ (0.63 g, 5.2 mmol) were used as drying agents. The product was isolated with flash chromatography (silica gel, *n*-Hex:Et_3N 100:2) as a yellow oil (196.7 mg, 0.68 mmol, 68%).

^{1}H NMR (400 MHz, $CDCl_3$) δ 8.74 (q, *J* = 2.4 Hz, 1H), 8.42 (d, *J* = 7.8 Hz, 1H), 7.73 (d, *J* = 7.8 Hz, 1H), 7.66 (t, *J* = 7.6 Hz, 1H), 7.56 (t, *J* = 7.4 Hz, 1H), 7.34 – 7.28 (m, 2H), 7.24 – 7.19 (m, 1H), 7.00 – 6.93 (m, 1H), 5.20 (dq, *J* = 3.1, 1.5 Hz, 1H), 4.99 (dd, *J* = 2.0, 0.9 Hz, 1H), 2.14 (dd, *J* = 1.5, 0.9 Hz, 3H).

^{13}C NMR (101 MHz, $CDCl_3$) δ 156.2 (d, *J* = 2.2 Hz, CH), 149.5 (C), 144.4 (C), 137.4 (C), 134.4 (d, *J* = 1.2 Hz, C), 132.3 (CH), 130.7 (CH), 129.8 (q, *J* = 31.0 Hz, C), 129.0 (CH), 128.7 (CH), 128.3 (CH), 126.3 (CH), 125.9 (q, *J* = 5.7 Hz, CH), 124.3 (q, *J* = 274.3 Hz, CF_3), 118.9 (CH), 116.7 (CH_2), 24.0 (CH_3).

^{19}F NMR (377 MHz, $CDCl_3$) δ -57.5.

HRMS (EI) *m/z*: [M^+] calcd for $C_{17}H_{14}F_3N$ 289.1078, found: 289.1076.

1-(Furyl)-*N*-(2-(prop-1-en-2-yl)phenyl)methanimine (1k)

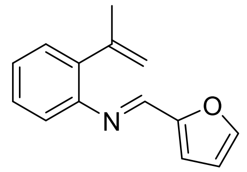

The product was prepared from 2-(prop-1-en-2-yl)aniline (0.49 mL, 3.6 mmol) and furfural (0.30 mL, 3.6 mmol) according to the general procedure A. Na_2SO_4 (2.5 g, 18 mmol) and $MgSO_4$ (2.5 g, 21 mmol) were used as drying agents. The product was isolated with flash chromatography (silica gel, *n*-Hex:Et_3N 100:3) as a dark orange oil (506.2 mg, 2.4 mmol, 66%).

^1H NMR (400 MHz, $CDCl_3$) δ 8.18 (s, 1H), 7.61 (dt, *J* = 1.8, 0.5 Hz, 1H), 7.31 – 7.24 (m, 2H), 7.17 (td, *J* = 7.5, 1.3 Hz, 1H), 6.95 (dd, *J* = 3.4, 0.8 Hz, 1H), 6.93 (dd, *J* = 7.7, 1.0 Hz, 1H), 6.55 (dd, *J* = 3.5, 1.8 Hz, 1H), 5.19 (dt, *J* = 3.0, 1.5 Hz, 1H), 5.02 (dd, *J* = 2.0, 0.9 Hz, 1H), 2.13 (dd, *J* = 1.5, 0.9 Hz, 3H).

^{13}C NMR (101 MHz, $CDCl_3$) δ 152.6 (C), 149.8 (C), 148.3 (CH), 145.7 (CH), 144.4 (C), 137.2 (C), 129.0 (CH), 128.2 (CH), 125.7 (CH), 119.1 (CH), 116.8 (CH_2), 115.5 (CH), 112.2 (CH), 23.9 (CH_3).

HRMS (EI) *m/z*: [M^+] calcd for $C_{14}H_{13}NO$ 211.0997, found: 211.0993.

***N*-(2-(Prop-1-en-2-yl)phenyl)-1-(thiophen-2-yl)methanimine (1l)**

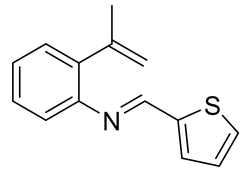

The product was prepared from 2-(prop-1-en-2-yl)aniline (0.49 mL, 3.6 mmol) and thiophene-2-carboxaldehyde (0.34 mL, 3.6 mmol) according to the general procedure A. Na_2SO_4 (2.5 g, 18 mmol) and $MgSO_4$ (2.5 g, 21 mmol) were used as drying agents. The product was isolated with flash chromatography (silica gel, *n*-Hex:Et_3N 100:3) as a yellow oil (633.2 mg, 2.8 mmol, 77%).

^1H NMR (400 MHz, $CDCl_3$) δ 8.46 (d, *J* = 1.2 Hz, 1H), 7.50 (dt, *J* = 5.0, 1.1 Hz, 1H), 7.46 (dd, *J* = 3.7, 0.9 Hz, 1H), 7.31 – 7.25 (m, 2H), 7.17 (td, *J* = 7.5, 1.3 Hz, 1H), 7.13 (dd, *J* = 5.0, 3.6 Hz, 1H), 6.96 (dd, *J* = 7.8, 1.0 Hz, 1H), 5.17 (dq, *J* = 3.0, 1.5 Hz, 1H), 5.03 (dd, *J* = 2.1, 0.9 Hz, 1H), 2.15 (dd, *J* = 1.5, 0.9 Hz, 3H).

^{13}C NMR (101 MHz, $CDCl_3$) δ 152.8 (CH), 149.4 (C), 144.9 (C), 143.4 (C), 137.3 (C), 131.8 (CH), 130.5 (CH), 129.1 (CH), 128.2 (CH), 127.9 (CH), 125.7 (CH), 118.9 (CH), 116.5 (CH_2), 23.8 (CH_3).

HRMS (EI) *m/z*: [M^+] calcd for $C_{14}H_{13}NS$ 227.0769, found: 227.0772.

***N*-(2-(Prop-1-en-2-yl)phenyl)-1-(pyridin-2-yl)methanimine (1m)**

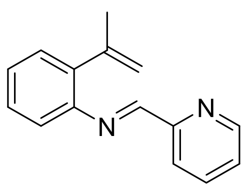

The product was prepared from 2-(prop-1-en-2-yl)aniline (0.14 mL, 1.0 mmol) and 2-pyridinecarboxaldehyde (0.10 mL, 1.1 mmol) according to the general procedure A. Na_2SO_4 (2.1 g, 15 mmol) and $MgSO_4$ (2.1 g, 17 mmol) were used as drying agents. The product was isolated with flash chromatography (silica gel, *n*-Hex:Et_3N 100:2) as a yellow oil (167.2 mg, 0.75 mmol, 73%).

^{1}H NMR (400 MHz, CDCl$_3$) δ 8.70 (ddd, *J* = 4.9, 1.8, 1.0 Hz, 1H), 8.52 (d, *J* = 0.7 Hz, 1H), 8.22 (dt, *J* = 7.9, 1.1 Hz, 1H), 7.84 – 7.78 (m, 1H), 7.37 (ddd, *J* = 7.5, 4.9, 1.2 Hz, 1H), 7.34 – 7.28 (m, 2H), 7.24 – 7.19 (m, 1H), 7.05 – 7.01 (m, 1H), 5.20 (dt, *J* = 3.0, 1.5 Hz, 1H), 5.01 (dd, *J* = 2.1, 1.0 Hz, 1H), 2.16 (dd, *J* = 1.5, 0.9 Hz, 3H).

^{13}C NMR (101 MHz, CDCl$_3$) δ 160.5 (CH), 155.0 (C), 149.8 (CH), 148.9 (C), 144.6 (C), 137.7 (C), 136.8 (CH), 129.0 (CH), 128.3 (CH), 126.4 (CH), 125.2 (CH), 121.8 (CH), 118.6 (CH), 116.6 (CH$_2$), 24.0 (CH$_3$).

HRMS (EI) *m/z*: [M$^+$] calcd for $C_{15}H_{14}N_2$ 222.1157, found: 222.1148.

1-(1-Methyl-1*H*-pyrrol-2-yl)-*N*-(2-(prop-1-en-2-yl)phenyl)methanimine (1n)

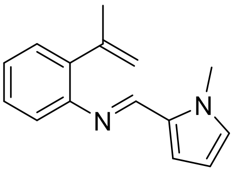

The synthesis was performed following the procedure modified from literature.[6] 2-(Prop-1-en-2-yl)aniline (0.20 mL, 1.5 mmol) and *N*-methyl-2-pyrrolecarboxaldehyde (0.16 mL, 1.5 mmol) were added to the flask via septum and the reaction mixture was stirred at 60 °C overnight. The product was isolated with flash chromatography (silica gel, *n*-Hex: Et$_3$N 100:2) as a yellow oil (220.6 mg, 0.98 mmol, 66%).

^{1}H NMR (400 MHz, CDCl$_3$) δ 8.20 (s, 1H), 7.29 – 7.22 (m, 2H), 7.16 – 7.09 (m, 1H), 6.91 (ddd, *J* = 7.7, 1.3, 0.5 Hz, 1H), 6.79 (t, *J* = 2.2 Hz, 1H), 6.64 (dd, *J* = 3.8, 1.8 Hz, 1H), 6.20 (dd, *J* = 3.8, 2.6 Hz, 1H), 5.12 (dq, *J* = 2.3, 1.5 Hz, 1H), 4.98 (dd, *J* = 2.2, 0.9 Hz, 1H), 4.04 (s, 3H), 2.12 (dd, *J* = 1.5, 0.9 Hz, 3H).

^{13}C NMR (101 MHz, CDCl$_3$) δ 150.8 (C), 150.7 (CH), 145.4 (C), 137.8 (C), 130.7 (C), 129.2 (CH), 128.9 (CH), 128.3 (CH), 124.9 (CH), 118.8 (CH), 118.5 (CH), 115.5 (CH$_2$), 108.7 (CH), 37.1 (CH$_3$), 24.0 (CH$_3$).

HRMS (EI) *m/z*: [M$^+$] calcd for $C_{15}H_{16}N_2$ 224.1313, found: 224.1307.

N-(2-(Prop-1-en-2-yl)phenyl)-1-(1*H*-pyrrol-2-yl)methanimine (1o)

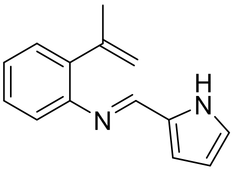

The synthesis was performed following the procedure modified from literature.[7] A two-neck flask equipped with a stirrer bar was loaded with 2-pyrrolecarboxaldehyde (380.4 mg, 4.0 mmol), anhydrous EtOH (10 mL) and 2-(prop-1-en-2-yl)aniline (0.55 mL, 4.0 mmol). The solution was bubbled with argon for 30 min and then stirred at r.t. for 73 h. The solvents were evaporated from the reaction mixture on rotary evaporator. The crude was recrystallized from *n*-Hex. The resulting crystals contained black insoluble particles, so they were dissolved in acetone and filtered. The solvent was evaporated on rotary evaporator and the second recrystallization from *n*-Hex afforded the product as flesh-colored crystals (557.0 mg, 2.6 mmol, 66%).

^{1}H NMR (400 MHz, acetone-*d*$_6$) 10.79 (s, 1H), 8.19 (s, 1H), 7.32 – 7.20 (m, 2H), 7.12 (td, *J* = 7.5, 1.3 Hz, 1H), 7.07 – 7.04 (m, 1H), 6.95 (dd, *J* = 7.8, 1.2 Hz, 1H), 6.69 (dt, *J* = 3.5, 1.7 Hz, 1H), 6.27 – 6.23 (m, 1H), 5.08 (dt, *J* = 3.0, 1.5 Hz, 1H), 4.95 (dq, *J* = 1.9, 1.0 Hz, 1H), 2.10 (dd, *J* = 1.5, 0.9 Hz, 3H).

^{13}C NMR (101 MHz, acetone-*d*$_6$) 151.4 (C), 150.6 (CH), 146.5 (C), 138.0 (C), 132.1 (C), 129.5 (CH), 129.1 (CH), 125.5 (CH), 124.2 (CH), 119.6 (CH), 116.8 (CH), 115.8 (CH$_2$), 110.6 (CH), 24.1 (CH$_3$).

HRMS (EI) *m/z*: [M$^+$] calcd for $C_{14}H_{14}N_2$ 210.1157, found: 210.1159.

2,2-Dimethyl-*N*-(2-(prop-1-en-2-yl)phenyl)propan-1-imine (1p)

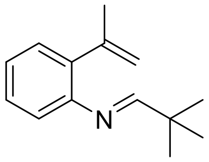

The product was prepared from 2-(prop-1-en-2-yl)aniline (0.14 mL, 1.0 mmol) and pivaldehyde (0.11 mL, 1.0 mmol) at r.t. according to the general procedure B. The reaction time was 45 min. Imine was too labile to sustain flash chromatography on silica gel. The crude was a clear liquid containing 5 wt% unreacted aniline and 95 wt% imine (167.7 mg, 0.84 mmol, 84%).

^{1}H NMR (400 MHz, $CDCl_3$) δ 7.58 (s, 1H), 7.23 – 7.18 (m, 2H), 7.11 – 7.06 (m, 1H), 6.73 (dd, *J* = 8.1, 1.3 Hz, 1H), 5.12 (dq, *J* = 2.2, 1.5 Hz, 1H), 4.96 (dd, *J* = 2.2, 0.9 Hz, 1H), 2.06 (dd, *J* = 1.5, 0.9 Hz, 3H), 1.17 (s, 9H).

^{13}C NMR (101 MHz, $CDCl_3$) δ 172.8 (CH), 150.3 (C), 144.7 (C), 136.1 (C), 128.7 (CH), 128.0 (CH), 124.7 (CH), 119.4 (CH), 116.1 (CH_2), 37.0 (C), 26.6 (CH_3), 23.6 (CH_3).

HRMS (EI) *m/z*: [M^+] calcd for $C_{14}H_{19}N$ 201.1517, found: 201.1526.

1-Cyclopropyl-*N*-(2-(prop-1-en-2-yl)phenyl)methanimine (1q)

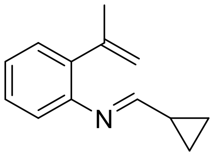

The product was prepared from 2-(prop-1-en-2-yl)aniline (0.27 mL, 2.0 mmol) and cyclopropanecarboxaldehyde (0.15 mL, 2.0 mmol) at r.t. according to the general procedure B. The reaction time was 2 h. Imine was too labile to sustain flash chromatography on silica gel. The crude was a pale red liquid containing 71 wt% imine (154.4 mg, 0.83 mmol, 42%). The rest was unreacted starting materials.

^{1}H NMR (400 MHz, acetone-d_6) δ 7.28 (d, *J* = 7.1 Hz, 1H), 7.23 – 7.15 (m, 2H), 7.07 (td, *J* = 7.5, 1.3 Hz, 1H), 6.77 – 6.74 (m, 1H), 5.09 (dq, *J* = 3.0, 1.5 Hz, 1H), 4.90 – 4.88 (m, 1H), 2.07 (dd, *J* = 1.5, 0.9 Hz, 3H), 1.88 – 1.78 (m, 1H), 1.01 – 0.95 (m, 2H), 0.95 – 0.85 (m, 2H).

^{13}C NMR (101 MHz, acetone-d_6) δ 168.4 (CH), 151.2 (C), 146.2 (C), 137.3 (C), 129.3 (CH), 128.9 (CH), 125.4 (CH), 119.9 (CH), 115.8 (CH_2), 23.8 (CH_3), 17.7 (CH), 7.2 (CH).

HRMS (EI) *m/z*: [M^+] calcd for $C_{13}H_{15}N$ 185.1204, found: 185.1201.

1-phenyl-*N*-(2-vinylphenyl)methanimine (1r)

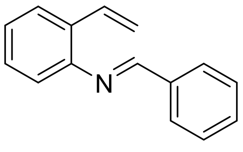

The product was prepared from 2-vinylaniline (0.15 mL, 1.3 mmol) and benzaldehyde (0.13 mL, 1.3 mmol) according to the general procedure A. Na_2SO_4 (800 mg, 5.6 mmol) and $MgSO_4$ (800 mg, 6.6 mmol) were used as drying agents. The product was isolated with flash chromatography (silica gel, *n*-Hex:Et_3N 100:2) as a pale yellow oil (202.5 mg, 0.98 mmol, 78%).

^{1}H NMR (400 MHz, acetone-d_6) δ 8.51 (s, 1H), 8.03 – 7.98 (m, 2H), 7.65 (dt, *J* = 7.7, 1.0 Hz, 1H), 7.56 – 7.51 (m, 3H), 7.33 (td, *J* = 7.6, 1.5 Hz, 1H), 7.30 – 7.20 (m, 2H), 7.08 (dd, *J* = 7.7, 1.3 Hz, 1H), 5.79 (dd, *J* = 17.8, 1.5 Hz, 1H), 5.26 (dd, *J* = 11.2, 1.5 Hz, 1H).

^{13}C NMR (101 MHz, acetone-d_6) δ 161.1, 150.7, 137.5, 134.2, 132.3, 132.2, 129.8, 129.7, 129.7, 126.8, 126.3, 119.2, 114.8.

The characterization data was in agreement with the previous literature.[8]

N-(2-(1-cyclopropylvinyl)phenyl)-1-phenylmethanimine (1s)

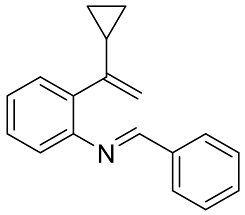

The product was prepared from 2-(1-cyclopropylvinyl)aniline[9] (300 mg, 1.9 mmol) and benzaldehyde (0.19 mL, 1.9 mmol) according to the general procedure A. The reaction temperature was 80 °C. Na_2SO_4 (1.2 g, 8.3 mmol) and $MgSO_4$ (1.2 g, 9.8 mmol) were used as drying agents. The product was isolated with flash chromatography (silica gel, *n*-Hex:Et_3N 100:2) as a yellow oil (276.5 mg, 1.1 mmol, 60%).

^{1}H NMR (400 MHz, acetone-d_6) δ 8.48 (s, 1H), 7.97 – 7.93 (m, 2H), 7.54 – 7.49 (m, 3H), 7.32 (ddd, *J* = 7.8, 7.3, 1.6 Hz, 1H), 7.28 (dd, *J* = 7.6, 1.5 Hz, 1H), 7.19 (td, *J* = 7.4, 1.3 Hz, 1H), 7.04 (dd, *J* = 7.8, 1.2 Hz, 1H), 5.04 (dd, *J* = 1.7, 1.1 Hz, 1H), 4.85 (d, *J* = 1.7 Hz, 1H), 1.77 (ttd, *J* = 8.3, 5.3, 1.0 Hz, 1H), 0.70 – 0.64 (m, 2H), 0.53 – 0.47 (m, 2H).

^{13}C NMR (101 MHz, acetone-d_6) δ 160.9 (CH), 151.2 (C), 151.1 (C), 137.7 (C), 137.3 (C), 132.1 (CH), 130.1 (CH), 129.6 (CH), 129.0 (CH), 126.1 (CH), 119.4 (CH), 112.3 (CH_2), 17.7 (CH), 7.4 (CH).

HRMS (EI) *m/z*: [M^+] calcd for $C_{18}H_{17}N$ 247.1361, found: 247.1357.

1-Phenyl-N-(2-(1-phenylvinyl)phenyl)methanimine (1t)

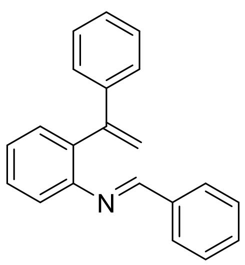

The product was prepared from 2-(1-phenylvinyl)aniline[9] (750.0 mg, 3.8 mmol) and benzaldehyde (0.39 mL, 3.8 mmol) according to the general procedure A. Na_2SO_4 (2.7 g, 19 mmol) and $MgSO_4$ (2.7 g, 22 mmol) were used as drying agents. According to ^{1}H NMR of the reaction mixture, the reaction had not proceeded well overnight, so it was further bubbled with argon for 30 min and then refluxed for another night. The product was isolated with flash chromatography (silica gel, *n*-Hex:Et_3N 100:2 and *n*-Hex:Et_3N 100:5) as a pale yellow liquid (548.0 mg, 1.9 mmol, 50%).

^{1}H NMR (400 MHz, $CDCl_3$) δ 8.18 (s, 1H), 7.53 – 7.48 (m, 2H), 7.42 – 7.12 (m, 11H), 6.98 (d, *J* = 7.7 Hz, 1H), 5.65 (d, *J* = 1.4 Hz, 1H), 5.31 (d, *J* = 1.4 Hz, 1H).

^{13}C NMR (101 MHz, $CDCl_3$) δ 160.1, 150.6, 148.6, 142.0, 136.4, 135.4, 131.2, 130.8, 128.9, 128.7, 128.5, 128.1, 127.3, 127.1, 125.5, 118.8, 116.7.

The characterization data was in agreement with the previous literature.[10]

1-(4-methoxyphenyl)-N-(2-(1-phenylvinyl)phenyl)methanimine (1u)

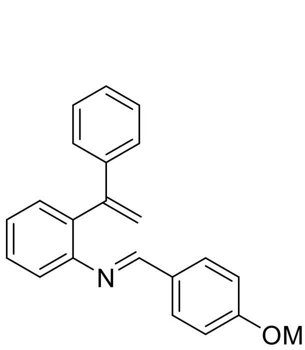

The product was prepared from 2-(1-phenylvinyl)aniline[9] (400 mg, 2.1 mmol) and *p*-anisaldehyde (0.25 mL, 2.1 mmol) according to the general procedure A. The reaction temperature was 80 °C. Na_2SO_4 (1.3 g, 9.0 mmol) and $MgSO_4$ (1.3 g, 11 mmol) were used as drying agents. Upon adding the flash chromatography eluent (*n*-Hex:Et_3N 100:2), the crude precipitated so the product was recrystallized from *n*-Hex as a yellow solid (370.1 mg, 1.2 mmol, 58%).

^{1}H NMR (400 MHz, acetone-d_6) δ 8.23 (s, 1H), 7.57 – 7.52 (m, 2H), 7.42 – 7.36 (m, 1H), 7.34 (dd, *J* = 7.5, 1.5 Hz, 1H), 7.28 – 7.14 (m, 6H), 7.04 (dd, *J* = 7.8, 1.2 Hz, 1H), 6.93 – 6.88 (m, 2H), 5.65 (d, *J* = 1.5 Hz, 1H), 5.23 (d, *J* = 1.5 Hz, 1H), 3.83 (s, 3H).

^{13}C NMR (101 MHz, acetone-d_6) δ ^{13}C NMR (101 MHz, Acetone) δ 163.1 (C), 159.7 (CH), 151.7 (C), 150.0 (C), 142.8 (C), 136.5 (C), 131.2 (CH), 131.1 (CH), 130.5 (C), 129.7 (CH), 128.8 (CH), 127.9 (CH), 127.6 (CH), 125.9 (CH), 119.3 (CH), 116.2 (CH_2), 114.7 (CH), 55.7 (CH_3).

HRMS (EI) *m/z*: [M^+] calcd for $C_{22}H_{19}NO$ 313.1467, found: 313.1470.

1-(4-Nitrophenyl)-*N*-(2-(1-phenylvinyl)phenyl)methanimine (1v)

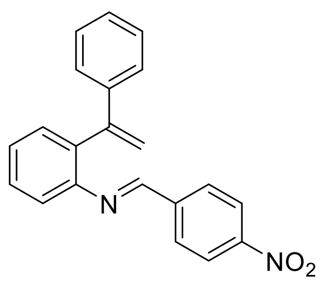

The product was prepared from 2-(1-phenylvinyl)aniline[9] (750.0 mg, 3.8 mmol) and 4-nitrobenzaldehyde (0.39 mL, 3.8 mmol) according to the general procedure A. The solid aldehyde was added to the flask together with drying agents, which were Na_2SO_4 (2.7 g, 19 mmol) and $MgSO_4$ (2.7 g, 22 mmol). According to ^1H NMR of the reaction mixture, the reaction had not proceeded well overnight, so it was further bubbled with argon for 30 min and then refluxed for another night. The product was isolated with several subsequent recrystallizations from EtOH as a yellow solid (170.0 mg, 0.52 mmol, 13%). The overall yield was not measured, as most of the product crystallized together with quinoline **2v**, which had formed as a side product.

^1H NMR (400 MHz, $CDCl_3$) δ 8.26 (s, 1H), 8.19 – 8.14 (m, 2H), 7.63 – 7.59 (m, 2H), 7.46 (dd, *J* = 7.5, 1.5 Hz, 1H), 7.41 (td, *J* = 7.6, 1.6 Hz, 1H), 7.31 (td, *J* = 7.5, 1.3 Hz, 1H), 7.25 – 7.13 (m, 5H), 7.01 (dd, *J* = 7.9, 1.2 Hz, 1H), 5.67 (d, *J* = 1.4 Hz, 1H), 5.33 (d, *J* = 1.4 Hz, 1H).

^{13}C NMR (101 MHz, $CDCl_3$) δ 157.0 (CH), 149.3 (C), 149.2 (C), 148.6 (C), 141.8 (C), 141.7 (C), 135.9 (C), 131.1 (CH), 129.2 (CH), 129.1 (CH), 128.2 (CH), 127.4 (CH), 127.0 (CH), 126.7 (CH), 123.8 (CH), 118.2 (CH), 116.8 (CH_2).

HRMS (EI) *m/z*: [M^+] calcd for $C_{21}H_{16}N_2O_2$ 328.1212, found: 328.1213.

N-(4-methoxy-2-(1-phenylvinyl)phenyl)-1-phenylmethanimine (1w)

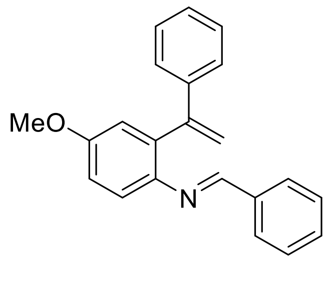

The product was prepared from 4-methoxy-2-(1-phenylvinyl)aniline[9] (738.9 mg, 3.3 mmol) and benzaldehyde (0.33 mL, 3.3 mmol) according to the general procedure A. Na_2SO_4 (2.0 g, 14 mmol) and $MgSO_4$ (2.0 g, 17 mmol) were used as drying agents. The product was isolated with flash chromatography (silica gel, *n*-Hex:Et_3N 100:2) as a yellow oil (697.6 mg, 2.2 mmol, 68%).

^1H NMR (400 MHz, $CDCl_3$) δ 8.23 (s, 1H), 7.50 – 7.47 (m, 2H), 7.38 – 7.33 (m, 1H), 7.33 – 7.27 (m, 4H), 7.25 – 7.20 (m, 2H), 7.19 – 7.14 (m, 1H), 7.01 (d, *J* = 8.5 Hz, 1H), 6.96 – 6.90 (m, 2H), 5.68 (d, *J* = 1.5 Hz, 1H), 5.32 (d, *J* = 1.4 Hz, 1H), 3.85 (s, 3H).

^{13}C NMR (101 MHz, $CDCl_3$) δ 158.4 (CH), 157.8 (C), 148.7 (C), 143.6 (C), 141.9 (C), 137.4 (C), 136.7 (C), 130.9 (CH), 128.6 (CH), 128.5 (CH), 128.1 (CH), 127.3 (CH), 127.1 (CH), 119.4 (CH), 116.6 (CH_2), 116.2 (CH), 114.0 (CH), 55.7 (CH_3).

HRMS (EI) *m/z*: [M^+] calcd for $C_{22}H_{19}NO$ 313.1467, found: 313.1464.

N-(4-Chloro-2-(1-phenylvinyl)phenyl)-1-phenylmethanimine (1x)

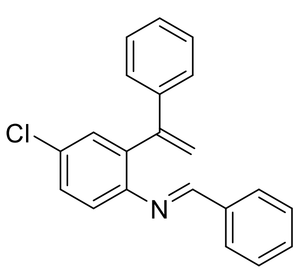

The product was prepared from 4-chloro-2-(1-phenylvinyl)aniline[9] (360.8 mg, 1.6 mmol) and benzaldehyde (0.16 mL, 1.6 mmol) according to the general procedure A. Na_2SO_4 (1.0 g, 6.9 mmol) and $MgSO_4$ (1.0 g, 8.2 mmol) were used as drying agents. The product was isolated with flash chromatography (silica gel, *n*-Hex:Et_3N 100:2) as a yellow oil (324.2 mg, 1.0 mmol, 65%).

^{1}H NMR (400 MHz, $CDCl_3$) δ 8.16 (s, 1H), 7.50 – 7.46 (m, 2H), 7.42 – 7.28 (m, 5H), 7.27 – 7.15 (m, 5H), 6.92 (d, *J* = 8.3 Hz, 1H), 5.67 (d, *J* = 1.3 Hz, 1H), 5.31 (d, *J* = 1.3 Hz, 1H).

^{13}C NMR (101 MHz, acetone-*d*$_6$) δ 161.3 (CH), 150.2 (C), 148.6 (C), 142.0 (C), 138.4 (C), 137.3 (C), 132.1 (CH), 130.8 (CH), 129.5 (CH), 129.3 (CH), 128.9 (CH), 128.2 (C), 127.6 (CH), 121.0 (CH), 117.3 (CH_2).

HRMS (EI) *m/z*: [M^+] calcd for $C_{21}H_{16}ClN$ 317.0971, found: 317.0971.

N-(4-Bromo-2-(1-phenylvinyl)phenyl)-1-phenylmethanimine (1y)

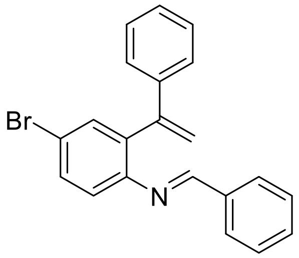

The product was prepared from 4-bromo-2-(1-phenylvinyl)aniline[9] (226.1 mg, 2.1 mmol) and benzaldehyde (0.22 mL, 2.1 mmol) according to the general procedure A. Na_2SO_4 (1.3 g, 9.4 mmol) and $MgSO_4$ (1.3 g, 11 mmol) were used as drying agents. The product was isolated with flash chromatography (silica gel, *n*-Hex:Et_3N 100:2) as a yellow oil (434.4 mg, 1.2 mmol, 56%).

^{1}H NMR (400 MHz, $CDCl_3$) δ 8.15 (s, 1H), 7.53 – 7.45 (m, 4H), 7.42 – 7.36 (m, 1H), 7.35 – 7.29 (m, 2H), 7.25 – 7.16 (m, 5H), 6.86 (d, *J* = 8.4 Hz, 1H), 5.66 (d, *J* = 1.2 Hz, 1H), 5.31 (d, *J* = 1.2 Hz, 1H).

^{13}C NMR (101 MHz, $CDCl_3$) δ 160.4 (CH), 149.7 (C), 147.5 (C), 141.3 (C), 137.4 (C), 136.1 (C), 133.4 (CH), 131.7 (CH), 131.4 (CH), 128.83 (CH), 128.80 (CH), 128.6 (CH), 128.2 (CH), 127.5 (CH), 127.0 (CH), 126.7 (CH), 120.4 (CH), 118.6 (C), 117.4 (CH_2).

HRMS (EI) *m/z*: [M^+] calcd for $C_{21}H_{16}BrN$ 361.0466, found: 361.0471.

N-(4-Fluoro-2-(1-phenylvinyl)phenyl)-1-phenylmethanimine (1z)

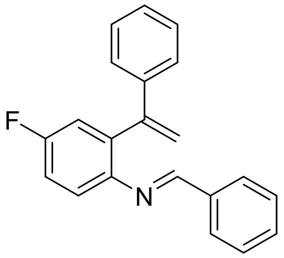

The product was prepared from 4-fluoro-2-(1-phenylvinyl)aniline[9] (178.5 mg, 1.7 mmol) and benzaldehyde (0.17 mL, 1.7 mmol) according to the general procedure A. Na_2SO_4 (1.1 g, 7.4 mmol) and $MgSO_4$ (1.1 g, 8.8 mmol) were used as drying agents. The product was isolated with flash chromatography (silica gel, *n*-Hex:Et_3N 100:2) as a yellow oil (269.5 mg, 0.89 mmol, 53%).

^{1}H NMR (400 MHz, $CDCl_3$) δ 8.18 (s, 1H), 7.51 – 7.48 (m, 2H), 7.41 – 7.36 (m, 1H), 7.35 – 7.30 (m, 2H), 7.28 – 7.15 (m, 5H), 7.11 (dd, *J* = 9.2, 2.8 Hz, 1H), 7.06 (td, *J* = 8.3, 2.9 Hz, 1H), 6.96 (dd, *J* = 8.6, 5.2 Hz, 1H), 5.68 (d, *J* = 1.3 Hz, 1H), 5.32 (d, *J* = 1.3 Hz, 1H).

^{13}C NMR (101 MHz, acetone-*d*$_6$) δ 161.5 (d, *J* = 242.7 Hz, CF), 160.7 (d, *J* = 1.3 Hz, CH), 148.8 (d, *J* = 1.3 Hz, C), 147.6 (d, *J* = 2.4 Hz, C), 142.1 (C), 138.7 (d, *J* = 7.9 Hz, C), 137.4 (C), 132.0 (CH), 129.4 (CH), 129.3 (CH), 128.9 (CH), 128.2 (CH), 127.6 (CH), 120.7 (d, *J* = 8.2 Hz, CH), 117.7 (d, *J* = 22.6 Hz, CH), 117.2 (CH_2), 116.1 (d, *J* = 22.4 Hz, CH).

^{19}F NMR (377 MHz, acetone-d_6) δ -118.8.

HRMS (EI) *m/z*: [M$^+$] calcd for $C_{21}H_{16}FN$ 301.1267, found: 301.1263.

Unsuitable substrates

The following imines **1aa**–**1ag** were also synthesized and tested in the cyclization reaction under the optimal reaction conditions. However, they were completely unreactive (imines **1aa**–**1ac**) or formed a complicated mixture of products with very little or untraceable amount of the target compound (imines **1ad**–**1ag**).

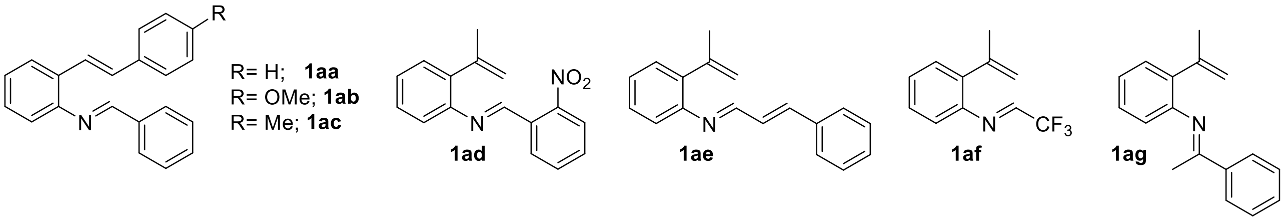

(1-Phenyl-*N*-(2-styryl)phenyl)methanimine (1aa)

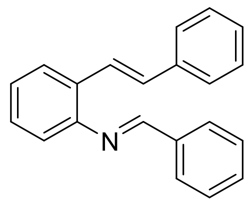

The product was prepared from 2-styrylaniline (781.1 mg, 4.0 mmol)[9] and benzaldehyde (0.41 mL, 4.0 mmol) according to the general procedure A. Na_2SO_4 (2.5 g, 18 mmol) and $MgSO_4$ (2.5 g, 21 mmol) were used as drying agents. The product was recrystallized from EtOH as pale yellow crystals (833.8 mg, 2.9 mmol, 74%).

^1H NMR (400 MHz, $CDCl_3$) δ 8.41 (s, 1H), 7.98 – 7.94 (m, 2H), 7.70 (dd, *J* = 7.6, 1.7 Hz, 1H), 7.63 (d, *J* = 16.5 Hz, 1H), 7.53 – 7.48 (m, 5H), 7.36 – 7.19 (m, 5H), 7.12 (d, *J* = 16.4 Hz, 1H), 6.98 (dd, *J* = 7.6, 1.5 Hz, 1H).

^{13}C NMR (101 MHz, $CDCl_3$) δ 160.3, 150.2, 138.0, 136.5, 131.6, 131.3, 129.8, 129.03, 128.96, 128.7, 128.6, 127.6, 126.8, 126.2, 125.9, 125.4, 118.7.

The characterization data was in agreement with the previous literature.[11]

***N*-(2-(4-Methoxystyryl)phenyl)-1-phenylmethanimine (1ab)**

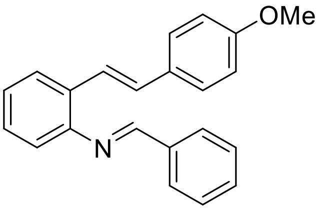

The product was prepared from 2-(4-methoxystyryl)aniline (201.4 mg, 0.89 mmol)[9] and benzaldehyde (0.09 mL, 0.89 mmol) according to the general procedure A. Na_2SO_4 (0.56 g, 3.9 mmol) and $MgSO_4$ (0.56 g, 4.7 mmol) were used as drying agents. The product was recrystallized from EtOH as an orange solid (141.4 mg, 0.45 mmol, 50%).

^1H NMR (400 MHz, $CDCl_3$) δ 8.41 (s, 1H), 7.98 – 7.94 (m, 2H), 7.68 (dd, *J* = 7.4, 1.8 Hz, 1H), 7.53 – 7.42 (m, 6H), 7.30 – 7.20 (m, 2H), 7.08 (d, *J* = 16.4 Hz, 1H), 6.99 – 6.96 (m, 1H), 6.90 – 6.85 (m, 2H), 3.81 (s, 3H).

^{13}C NMR (101 MHz, $CDCl_3$) δ 160.2 (CH), 159.4 (C), 150.0 (C), 136.6 (C), 131.6 (C), 131.5 (CH), 130.9 (C), 129.3 (CH), 129.02 (CH), 128.96 (CH), 128.2 (CH), 128.0 (CH), 126.1 (CH), 125.7 (CH), 123.3 (CH), 118.7 (CH), 114.2 (CH), 55.5 (CH_3).

HRMS (EI) *m/z*: [M$^+$] calcd for $C_{22}H_{19}NO$ 313.1467, found: 313.1470.

N-(2-(4-Methylstyryl)phenyl)-1-phenylmethanimine (1ac)

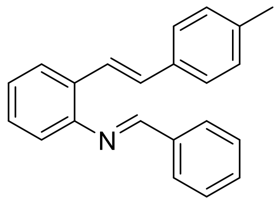

The product was prepared from 2-(4-methylstyryl)aniline (418.6 mg, 2.0 mmol)[9] and benzaldehyde (0.20 mL, 2.0 mmol) according to the general procedure A. Na_2SO_4 (1.3 g, 8.8 mmol) and $MgSO_4$ (1.3 g, 10 mmol) were used as drying agents. The product was recrystallized from EtOH as a pale yellow solid (375.2 mg, 1.3 mmol, 63%).

^{1}H NMR (400 MHz, $CDCl_3$) δ 8.40 (s, 1H), 7.98 – 7.93 (m, 2H), 7.68 (dd, J = 7.5, 1.7 Hz, 1H), 7.57 (d, J = 16.4 Hz, 1H), 7.52 – 7.47 (m, 3H), 7.40 (d, J = 8.2 Hz, 2H), 7.27 (td, J = 7.5, 1.8 Hz, 1H), 7.22 (td, J = 7.5, 1.7 Hz, 1H), 7.13 (d, J = 8.1 Hz, 2H), 7.09 (d, J = 16.5 Hz, 1H), 6.97 (dd, J = 7.5, 1.6 Hz, 1H), 2.33 (s, 3H).

^{13}C NMR (101 MHz, $CDCl_3$) δ 160.2 (CH), 150.1 (C), 137.5 (C), 136.5 (C), 135.2 (C), 131.52 (CH), 131.47 (C), 129.7 (CH), 129.5 (CH), 129.0 (CH), 128.9 (CH), 128.4 (CH), 126.7 (CH), 126.1 (CH), 125.8 (CH), 124.4 (CH), 118.7 (CH), 21.4 (CH_3).

HRMS (EI) m/z: [M^+] calcd for $C_{22}H_{19}N$ 297.1517, found: 297.1518.

1-(2-Nitrophenyl)-N-(2-(prop-1-en-2-yl)phenyl)methanimine (1ad)

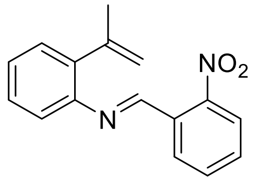

The synthesis was performed following the procedure modified from literature.[5] A two-neck flask equipped with a stirrer bar was loaded with Na_2SO_4 (2.1 g, 15 mmol), $MgSO_4$ (2.0 g, 17 mmol) and anhydrous THF (15 mL). The suspension was bubbled with argon for 15 min. 2-Nitrobenzaldehyde (349.0 mg, 2.3 mmol) and 2-(prop-1-en-2-yl)aniline (0.31 mL, 2.3 mmol) were added and the mixture was stirred at r.t. overnight and the refluxed for 4 h. The mixture was filtered through Celite and washed with EtOAc. The solvents were evaporated from the filtrate on rotary evaporator. The recrystallization from EtOH afforded the product as a yellow solid (406.6 mg, 1.5 mmol, 66%).

^{1}H NMR (400 MHz, $CDCl_3$) δ 8.85 (s, 1H), 8.29 (dd, J = 7.8, 1.5 Hz, 1H), 8.07 (dd, J = 8.2, 1.3 Hz, 1H), 7.74 (t, J = 7.5 Hz, 1H), 7.65 – 7.59 (m, 1H), 7.37 – 7.28 (m, 2H), 7.28 – 7.19 (m, 1H), 7.09 – 7.01 (m, 1H), 5.23 (p, J = 1.6 Hz, 1H), 4.99 (dd, J = 2.1, 1.0 Hz, 1H), 2.16 (dd, J = 1.5, 0.9 Hz, 3H).

^{13}C NMR (101 MHz, $CDCl_3$) δ 155.6, 149.5, 149.0, 144.3, 137.7, 133.8, 131.4, 131.2, 129.9, 129.0, 128.4, 126.6, 124.7, 119.0, 116.8, 24.1.

The characterization data was in agreement with the previous literature.[4]

3-Phenyl-N-(2-(prop-1-en-2-yl)phenyl)prop-2-en-1-imine (1ae)

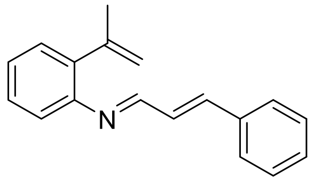

The product was prepared from 2-(prop-1-en-2-yl)aniline (0.54 mL, 4.0 mmol) and cinnamaldehyde (0.51 mL, 4.0 mmol) according to the general procedure A. Na_2SO_4 (2.5 g, 18 mmol) and $MgSO_4$ (2.5 g, 21 mmol) were used as drying agents. The product was isolated with flash chromatography (silica gel, n-Hex:Et_3N 100:3) as a yellow oil (782.9 mg, 3.2 mmol, 79%).

^{1}H NMR (400 MHz, $CDCl_3$) δ 8.17 (dd, J = 6.6, 1.7 Hz, 1H), 7.56 – 7.51 (m, 2H), 7.42 – 7.34 (m, 3H), 7.30 – 7.24 (m, 2H), 7.19 – 7.11 (m, 3H), 6.90 (dd, J = 8.1, 1.3 Hz, 1H), 5.21 (dt, J = 3.0, 1.5 Hz, 1H), 4.99 (dd, J = 2.1, 1.0 Hz, 1H), 2.11 (dd, J = 1.6, 0.9 Hz, 3H).

^{13}C NMR (101 MHz, $CDCl_3$) δ 161.9 (CH), 150.0 (C), 144.5 (C), 143.8 (CH), 137.4 (C), 135.8 (C), 129.7 (CH), 129.1 (CH), 128.9 (CH), 128.2 (CH), 127.6 (CH), 125.7 (CH), 119.0 (CH), 116.6 (CH_2), 24.1 (CH_3).

HRMS (EI) *m/z*: [M^+] calcd for $C_{18}H_{17}N$ 247.1361, found: 247.1357.

2,2,2-Trifluoro-*N*-(2-(prop-1-en-2-yl)phenyl)ethan-1-imine (1af)

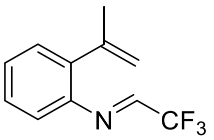

The product was prepared from 2-(prop-1-en-2-yl)aniline (0.27 mL, 2.0 mmol) and trifluoroacetaldehyde ethyl hemiacetal (0.24 mL, 2.0 mmol) at r.t. according to the general procedure B. The reaction time was 22 h. Imine was too labile to sustain flash chromatography on silica gel. The crude was a clear liquid containing 70 wt% imine (219.4 mg, 1.0 mmol, 51%). The rest was unreacted starting materials.

^1H NMR (400 MHz, $CDCl_3$) δ 7.22 – 7.16 (m, 1H), 7.11 – 7.08 (m, 1H), 6.89 (m, 2H), 5.35 (p, *J* = 1.6 Hz, 1H), 5.32 – 5.25 (m, 1H), 5.02 (dd, *J* = 2.0, 1.0 Hz, 1H), 2.05 (dd, *J* = 1.5, 1.0 Hz, 3H).

^{13}C NMR (101 MHz, $CDCl_3$) δ 142.9 (C), 139.8 (C), 131.5 (C), 128.6 (CH), 128.4 (CH), 123.0 (q, *J* = 282.6 Hz, CF_3), 120.4 (CH), 116.7 (CH_2), 112.9 (CH), 77.4 (CH), 24.3 (CH_3).

^{19}F NMR (377 MHz, $CDCl_3$) δ -82.5.

HRMS (EI) *m/z*: [M^+] calcd for $C_{11}H_{10}F_3N$ 213.0765, found: 213.0765.

1-Phenyl-*N*-(2-(prop-1-en-2-yl)phenyl)ethan-1-imine (1ag)

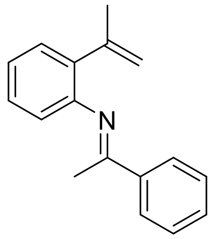

The product was prepared from 2-(prop-1-en-2-yl)aniline (0.54 mL, 4.0 mmol) and acetophenone (0.47 mL, 4.0 mmol) according to the general procedure A. Na_2SO_4 (2.5 g, 18 mmol) and $MgSO_4$ (2.5 g, 21 mmol) were used as drying agents. The product was isolated with flash chromatography (silica gel, *n*-Hex:Et_3N 100:2) as a yellow oil (181.2 mg, 0.77 mmol, 19%).

^1H NMR (400 MHz, acetone-d_6) δ 8.06 – 8.00 (m, 2H), 7.51 – 7.44 (m, 3H), 7.30 – 7.24 (m, 2H), 7.06 (td, *J* = 7.5, 1.3 Hz, 1H), 6.68 (dd, *J* = 8.2, 1.3 Hz, 1H), 5.02 (dt, *J* = 3.0, 1.5 Hz, 1H), 4.93 (dd, *J* = 2.2, 1.0 Hz, 1H), 2.15 (s, 3H), 1.99 (dd, *J* = 1.5, 0.9 Hz, 3H).

^{13}C NMR (101 MHz, $CDCl_3$) δ 165.1 (C), 149.1 (C), 145.1 (C), 139.5 (C), 133.8 (C), 130.5 (CH), 129.0 (CH), 128.5 (CH), 127.9 (CH), 127.3 (CH), 123.4 (CH), 119.2 (CH), 115.5 (CH_2), 22.6 (CH_3), 17.8 (CH_3).

HRMS (EI) *m/z*: [M^+] calcd for $C_{17}H_{17}N$ 235.1361, found: 235.1369.

Optimization of reaction conditions

General screening procedure

Imine **1a**, photocatalyst (varying equivalent), additive (varying equivalent) and solvent were added in a vial, which was sealed with a septum. The atmosphere in the vial was exchanged with respective gas (balloon) for 15 min. The balloon was left attached and the reaction mixture was stirred and irradiated with blue LEDs (455 nm) at r.t. for 30 min or 1 h. A stock solution of 1,3,5-trimethoxybenzene (TMB) in anisole (0.50 M) or in DMSO (0.48 M) was prepared in a volumetric flask and used as an internal standard for the reaction condition screening.

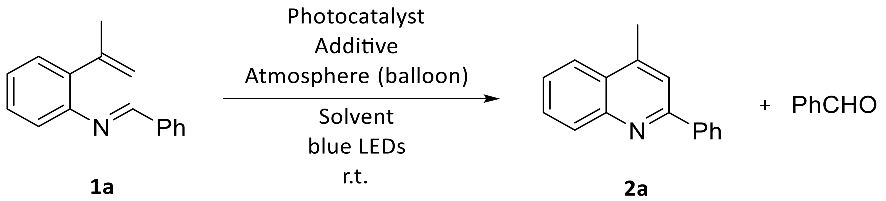

Table S1. Optimization of solvent. Conditions: Imine **1a** (22.1 mg, 0.1 mmol), PQ (3.1 mg, 15 mol%), $MgCO_3$ (20 mg), dry air (balloon), blue LEDs (455 nm), concentration 0.1 M in respective solvent (1 mL). Reaction time 30 min.

Entry	Variation: Solvent	Unreacted **1a** (%)[a]	Yield of **2a** (%)[a]	Formed PhCHO (%)[a]
**S1**	**DCM**	**6**	**90**	**3**
S2	MeCN	43	44	3
S3	toluene	35	51	4
S4	EtOAc	41	48	2
S5	acetone	53	37	2
S6	EtOH	6	65	10
S7	DMSO	71	20	7
S8	THF	90	0	10

[a]NMR yield; TMB as internal standard.

Table S2. Optimization of concentration. Conditions: Imine **1a** (22.1 mg, 0.1 mmol), PQ (3.1 mg, 20 mol%), $MgCO_3$ (20 mg), dry air (balloon), blue LEDs (455 nm), varying concentration in DCM. Reaction time 30 min.

Entry	Variation: Concentration	Unreacted **1a** (%)[a]	Yield of **2a** (%)[a]	Formed PhCHO (%)[a]
S1	0.12 M (0.83 mL)	2	90	2
**S2**	**0.1 M (1 mL)**	**0**	**97**	**1**
S3	0.08 M (1.25 mL)	8	80	4
S4	0.06 M (1.67 mL)	3	90	2
S5	0.04 M (2.5 mL)	5	86	3

[a]NMR yield; TMB as internal standard.

Table S3. Optimization of additive. Conditions: Imine **1a** (22.1 mg, 0.1 mmol), PQ (4.2 mg, 20 mol%), respective additive, O_2 (balloon), blue LEDs (455 nm), concentration 0.1 M in DCM (1 mL). Reaction time 1 h.

Entry	Variation: Additive	Unreacted **1a** (%)[a]	Yield of **2a** (%)[a]	Formed PhCHO (%)[a]
S1	4Å molecular sieves	0	68	1
S2	Na_2SO_4 (5 equiv)	7	54	22
S3	$MgSO_4$ (5 equiv)	1	54	21
S4	K_2CO_3 (5 equiv)	0	83	3
**S5**	**$MgCO_3$ *n*-hydrate (60 mg/mL)**	**0**	**91**	**0**
S6	Cs_2CO_3 (5 equiv)	0	81	0

[a]NMR yield; TMB as internal standard.

Table S4. Optimization of $MgCO_3$ loading. Conditions: Imine **1a** (22.1 mg, 0.1 mmol), PQ (4.2 mg, 20 mol%), $MgCO_3$ (varying amount), O_2 (balloon), blue LEDs (455 nm), concentration 0.1 M in DCM (1 mL). Reaction time 1 h.

Entry	Variation: $MgCO_3$ loading	Unreacted **1a** (%)[a]	Yield of **2a** (%)[a]	Formed PhCHO (%)[a]
S1	5 mg/mL	1	76	7
S2	10 mg/mL	0	94	1
**S3**	**20 mg/mL**	**0**	**97**	**0**
S4	40 mg/mL	0	96	0
S5	60 mg/mL	0	91	0
S6	80 mg/mL	0	88	0

[a]NMR yield; TMB as internal standard.

Table S5. Optimization of photocatalyst. Conditions: Imine **1a** (22.1 mg, 0.1 mmol), varying photocatalyst, $MgCO_3$ (20 mg), dry air (balloon), blue LEDs (455 nm), concentration 0.1 M in DCM (1 mL). Reaction time 30 min.

Entry	Variation: Photocatalyst & light	Unreacted **1a** (%)[a]	Yield of **2a** (%)[a]	Formed PhCHO (%)[a]
**S1**	**PQ (10 mol%)**	**16**	**71**	**7**
S2	Acr^+-Mes ClO_4^- (5 mol%)	76	2	8
S3	Eosin Y (5 mol%)[b]	96	2	2
S4	Anthraquinone (10 mol%)	97	0	3

[a]NMR yield; TMB as internal standard. [b]Green LEDs (520 nm) were used as the light source.

Table S6. Optimization of PQ loading. Conditions: Imine **1a** (22.1 mg, 0.1 mmol), PQ (varying loading), $MgCO_3$ (20 mg), O_2 (balloon), blue LEDs (455 nm), concentration 0.1 M in DCM (1 mL). Reaction time 1 h.

Entry	Variation: PQ loading	Unreacted **1a** (%)[a]	Yield of **2a** (%)[a]	Formed PhCHO (%)[a]
S1	25 mol%	0	95	0
S2	20 mol%	0	97	0
**S3**	**15 mol%**	**0**	**99**	**0**
S4	10 mol%	0	90	1

[a]NMR yield; TMB as internal standard.

Table S7. Optimization of other conditions: Imine **1a** (22.1 mg, 0.1 mmol), PQ (3.1 mg, 15 mol%), $MgCO_3$ (20 mg), dry air (balloon), blue LEDs (455 nm), concentration 0.1 M in DCM (1 mL). Reaction time 30 min.

Entry	Variation: Miscellaneous	Unreacted **1a** (%)[a]	Yield of **2a** (%)[a]	Formed PhCHO (%)[a]
S1	no light	97	0	2
S2	no PQ	100	0	0
S3	no $MgCO_3$	36	45	16
S4	Ar atmosphere	81	15	4
S5	O_2 atmosphere	3	84	2
S6	CFL bulb as light source[b]	90	7	3

[a]NMR yield; TMB as internal standard. [b]16 W common household lamp, 1.0 cm distance from the reaction vial.

Synthesis and characterization of quinolines 2a–2z

General procedure C

Imine **1a**–**1z** (1 equiv)**,** PQ (15 mol%), $MgCO_3$ *n*-hydrate (20 mg/mL, 2.1 equiv) and DCM (1 or 2 mL, so that the concentration of imine was 0.1 M) were added in a 20 mL vial, which was sealed with a septum. The atmosphere in the vial was exchanged with dry air (balloon) for 15 min. The balloon was left attached and the reaction mixture was stirred and irradiated with blue LEDs (455 nm) at r.t. for the time needed. The reaction mixture was filtered through Celite and washed with DCM. The solvent was evaporated from the filtrate on rotary evaporator and the crude was purified with flash chromatography on silica gel.

4-Methyl-2-phenylquinoline (2a)

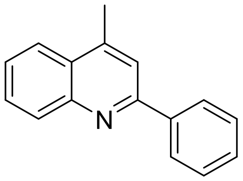

The product was prepared from **1a** according to the general procedure C in 0.21 mmol scale with the reaction time of 1 h and isolated with flash chromatography (silica gel, *n*-Hex:EtOAc 40:1 → 20:1) as a yellow oil (38.8 mg, 0.18 mmol, 84%).

^{1}H NMR (400 MHz, acetone-d_6) δ 8.37 – 8.25 (m, 2H), 8.11 (ddd, J = 8.4, 1.2, 0.6 Hz, 1H), 8.07 (dd, J = 8.2, 1.1 Hz, 1H), 7.95 (d, J = 0.9 Hz, 1H), 7.75 (ddd, J = 8.4, 6.9, 1.5 Hz, 1H), 7.60 – 7.45 (m, 4H), 2.77 (d, J = 1.0 Hz, 3H).

^{13}C NMR (101 MHz, acetone-d_6) δ 157.1, 149.0, 145.9, 140.3, 131.0, 130.14, 130.11, 129.5, 128.1, 128.1, 126.9, 124.7, 119.9, 18.9.

The characterization data was in agreement with the previous literature.[12]

2-(4-Methoxyphenyl)-4-methyl-quinoline (2b)

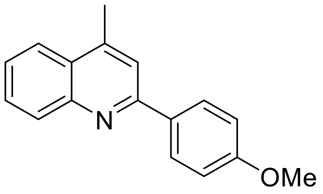

The product was prepared from **1b** according to the general procedure C in 0.21 mmol scale with the reaction time of 1 h and isolated with flash chromatography (silica gel, *n*-Hex:EtOAc [2% Et_3N] 80:1 → 40:1) as a clear oil (42.4 mg, 0.17 mmol, 81%).

^{1}H NMR (400 MHz, acetone-d_6) δ 8.32 – 8.23 (m, 2H), 8.05 (dddd, J = 9.5, 8.3, 1.4, 0.6 Hz, 1H), 7.89 (q, J = 1.0 Hz, 1H), 7.72 (ddd, J = 8.4, 6.8, 1.5 Hz, 1H), 7.54 (ddd, J = 8.2, 6.8, 1.3 Hz, 1H), 7.12 – 7.03 (m, 2H), 3.87 (s, 3H), 2.75 (d, J = 1.0 Hz, 3H).

^{13}C NMR (101 MHz, acetone-d_6) δ 161.9, 156.7, 149.0, 145.6, 132.8, 130.8, 130.0, 129.5, 127.9, 126.5, 124.7, 119.5, 114.8, 55.7, 18.9.

The characterization data was in agreement with the previous literature.[12]

4-Methyl-2-(4-nitrophenyl)quinoline (2c)

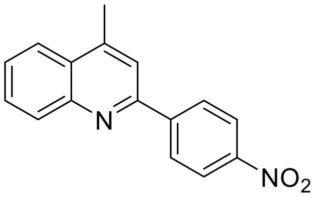

The product was prepared from **1c** according to the general procedure C in 0.20 mmol scale with the reaction time of 3 h and isolated with flash chromatography (silica gel, *n*-Hex:EtOAc 20:1) as a white solid (41.6 mg, 0.16 mmol, 79%).

^{1}H NMR (400 MHz, $CDCl_3$) δ 8.37 – 8.24 (m, 4H), 8.17 (dt, *J* = 8.3, 1.1 Hz, 1H), 8.00 (dt, *J* = 8.4, 0.9 Hz, 1H), 7.75 (ddd, *J* = 8.4, 6.8, 1.4 Hz, 1H), 7.71 (d, *J* = 1.0 Hz, 1H), 7.59 (ddd, *J* = 8.2, 6.8, 1.3 Hz, 1H), 2.78 (d, *J* = 1.0 Hz, 3H).

^{13}C NMR (101 MHz, $CDCl_3$) δ 154.3, 148.3, 148.2, 145.7, 145.7, 130.6, 130.0, 128.3, 127.7, 127.1, 124.0, 123.8, 119.6, 19.2.

The characterization data was in agreement with the previous literature.[12]

2-(4-methylphenyl)-4-methylquinoline (2d)

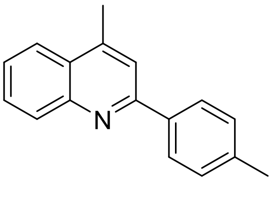

The product was prepared from **1d** according to the general procedure C in 0.20 mmol scale with the reaction time of 1 h and isolated with flash chromatography (silica gel, *n*-Hex:EtOAc 80:1 → 40:1) as a yellow solid (39.3 mg, 0.17 mmol, 84%).

^{1}H NMR (400 MHz, $CDCl_3$) δ 8.18 – 8.11 (m, 1H), 8.09 – 8.01 (m, 2H), 7.95 (dd, *J* = 8.3, 1.4 Hz, 1H), 7.73 – 7.62 (m, 2H), 7.50 (ddd, *J* = 8.3, 6.9, 1.3 Hz, 1H), 7.31 (d, *J* = 7.9 Hz, 2H), 2.72 (d, *J* = 1.0 Hz, 3H), 2.41 (s, 3H).

^{13}C NMR (101 MHz, $CDCl_3$) δ 157.1, 148.2, 144.7, 139.3, 137.1, 130.3, 129.6, 129.3, 127.5, 127.3, 125.9, 123.7, 119.7, 21.4, 19.1.

The characterization data was in agreement with the previous literature.[12]

2-(4-Bromophenyl)-4-methylquinoline (2e)

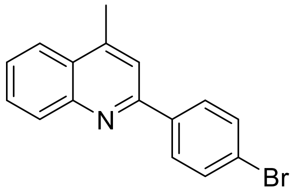

The product was prepared from **1e** according to the general procedure C in 0.20 mmol scale with the reaction time of 2 h and isolated with flash chromatography (silica gel, *n*-Hex:EtOAc 40:1) as a white solid (53.0 mg, 0.18 mmol, 87%).

^{1}H NMR (400 MHz, $CDCl_3$) δ 8.14 (d, *J* = 8.4 Hz, 1H), 8.05 – 7.97 (m, 2H), 7.95 (dd, *J* = 8.4, 1.4 Hz, 1H), 7.70 (ddd, *J* = 8.4, 6.8, 1.4 Hz, 1H), 7.65 – 7.57 (m, 3H), 7.52 (ddd, *J* = 8.2, 6.9, 1.3 Hz, 1H), 2.72 (d, *J* = 1.0 Hz, 3H).

^{13}C NMR (101 MHz, $CDCl_3$) δ 155.8, 148.2, 145.2, 138.7, 132.0, 130.4, 129.6, 129.2, 127.4, 126.4, 123.9, 123.8, 119.3, 19.1.

The characterization data was in agreement with the previous literature.[12]

2-(4-Cyanophenyl)-4-methylquinoline (2f)

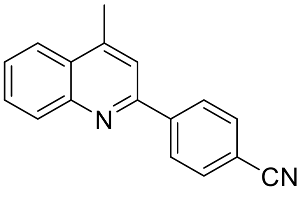

The product was prepared from **1f** according to the general procedure C in 0.20 mmol scale with the reaction time of 3 h and isolated with flash chromatography (silica gel, *n*-Hex:EtOAc 40:1 → 10:1) as a yellow solid (41.8 mg, 0.17 mmol, 88%).

^{1}H NMR (400 MHz, $CDCl_3$) δ 8.24 (d, *J* = 8.5 Hz, 2H), 8.15 (d, *J* = 8.2 Hz, 1H), 7.99 (dd, *J* = 8.4, 1.4 Hz, 1H), 7.80 – 7.71 (m, 3H), 7.67 (s, 1H), 7.58 (ddd, *J* = 8.3, 6.9, 1.3 Hz, 1H), 2.76 (d, *J* = 1.0 Hz, 3H).

^{13}C NMR (101 MHz, $CDCl_3$) δ 154.6, 148.1, 145.6, 143.9, 132.6, 130.5, 129.9, 128.1, 127.6, 127.0, 123.8, 119.4, 119.0, 112.6, 19.2.

The characterization data was in agreement with the previous literature.[13]

2-(3-Methoxyphenyl)-4-methylquinoline (2g)

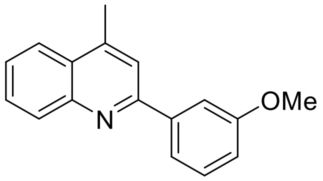

The product was prepared from **1g** according to the general procedure C in 0.19 mmol scale with the reaction time of 2 h and isolated with flash chromatography (silica gel, *n*-Hex:EtOAc [2% Et_3N] 80:1 → 40:1) as a clear oil (45.0 mg, 0.18 mmol, 93%).

^1H NMR (400 MHz, acetone-d_6) δ 8.09 (td, *J* = 8.2, 0.8 Hz, 2H), 7.96 (d, *J* = 1.0 Hz, 1H), 7.91 (dd, *J* = 2.6, 1.6 Hz, 1H), 7.86 (ddd, *J* = 7.7, 1.7, 1.0 Hz, 1H), 7.75 (ddd, *J* = 8.4, 6.9, 1.5 Hz, 1H), 7.58 (ddd, *J* = 8.2, 6.9, 1.3 Hz, 1H), 7.44 (t, *J* = 8.0 Hz, 1H), 7.05 (ddd, *J* = 8.2, 2.6, 0.9 Hz, 1H), 3.91 (s, 3H), 2.78 (d, *J* = 1.0 Hz, 3H).

^{13}C NMR (101 MHz, acetone-d_6) δ 161.2, 156.9, 149.0, 145.9, 141.8, 131.0, 130.5, 130.2, 128.2, 127.0, 124.8, 120.5, 120.1, 115.8, 113.5, 55.6, 18.8.

The characterization data was in agreement with the previous literature.[15]

2-(3-Nitrophenyl)-4-methylquinoline (2h)

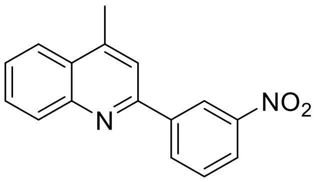

The product was prepared from **1h** according to the general procedure C in 0.20 mmol scale with the reaction time of 3 h and isolated with flash chromatography (silica gel, *n*-Hex:EtOAc [2% Et_3N] 80:1 → 40:1) as a white solid (49.9 mg, 0.19 mmol, 94%).

^1H NMR (400 MHz, $CDCl_3$) δ 8.98 (t, *J* = 2.0 Hz, 1H), 8.51 (dt, *J* = 7.8, 1.4 Hz, 1H), 8.26 (ddd, *J* = 8.2, 2.4, 1.1 Hz, 1H), 8.18 – 8.13 (m, 1H), 7.99 (dd, *J* = 8.4, 1.4 Hz, 1H), 7.78 – 7.69 (m, 2H), 7.65 (t, *J* = 8.0 Hz, 1H), 7.57 (ddd, *J* = 8.2, 6.8, 1.3 Hz, 1H), 2.77 (d, *J* = 1.0 Hz, 3H).

^{13}C NMR (101 MHz, $CDCl_3$) δ 154.1 (C), 148.9 (C), 148.1 (C), 145.8 (C), 141.5 (C), 133.4 (CH), 130.5 (CH), 129.9 (CH), 129.8 (CH), 127.7 (C), 126.9 (CH), 123.8 (CH), 123.8 (CH), 122.4 (CH), 119.1 (CH), 19.1 (CH_3).

HRMS (EI) *m/z*: [M^+] calcd for $C_{16}H_{12}N_2O_2$ 264.0899, found: 264.0899.

2-(2-Methoxyphenyl)-4-methylquinoline (2i)

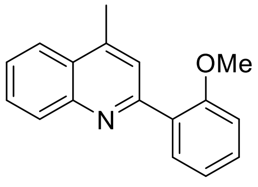

The product was prepared from **1i** according to the general procedure C in 0.21 mmol scale with the reaction time of 15 h and isolated with flash chromatography (silica gel, *n*-Hex:EtOAc [20:1 → 10:1) as a yellow solid (28.7 mg, 0.12 mmol, 54%).

^1H NMR (400 MHz, acetone-d_6) δ 8.11 – 8.03 (m, 2H), 7.90 (dd, *J* = 7.6, 1.8 Hz, 1H), 7.85 (d, *J* = 1.0 Hz, 1H), 7.73 (ddd, *J* = 8.4, 6.8, 1.4 Hz, 1H), 7.58 (ddd, *J* = 8.2, 6.9, 1.3 Hz, 1H), 7.44 (ddd, *J* = 8.3, 7.3, 1.8 Hz, 1H), 7.16 (dd, *J* = 8.4, 1.1 Hz, 1H), 7.10 (td, *J* = 7.5, 1.1 Hz, 1H), 3.90 (s, 3H), 2.74 (d, *J* = 1.0 Hz, 3H).

^{13}C NMR (101 MHz, acetone-d_6) δ 158.4, 157.4, 149.1, 143.7, 132.3, 131.1, 130.9, 130.5, 129.7, 127.87, 126.8, 124.7, 124.6, 121.5, 112.5, 56.0, 18.8.

The characterization data was in agreement with the previous literature.[14]

2-(2-Trifluoromethylphenyl)-4-methylquinoline (2j)

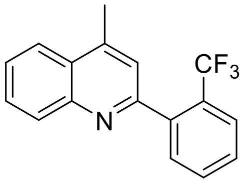

The product was prepared from **1j** according to the general procedure C in 0.18 mmol scale with the reaction time of 15 h and isolated with flash chromatography (silica gel, *n*-Hex:EtOAc [2% Et_3N] 80:1 → 40:1) as a clear oil (24.1 mg, 0.08 mmol, 48%).

^1H NMR (400 MHz, acetone-d_6) δ 8.16 (dd, *J* = 8.4, 0.9 Hz, 1H), 8.06 (ddd, *J* = 8.4, 1.3, 0.6 Hz, 1H), 7.91 – 7.86 (m, 1H), 7.82 – 7.75 (m, 2H), 7.73 – 7.63 (m, 3H), 7.50 (s, 1H), 2.79 (d, *J* = 1.0 Hz, 3H).

^{13}C NMR (101 MHz, acetone-d_6) δ 158.7 (C), 148.5 (C), 145.3 (C), 141.7 (d, *J* = 2.1 Hz, C), 132.8 (CH), 132.5 (CH), 131.0 (CH), 130.3 (CH), 129.5 (CH), 128.7 (q, *J* = 30.5 Hz, C), 127.9 (C), 127.5 (CH), 127.2 (q, *J* = 5.2 Hz, CH), 125.37 (q, *J* = 273.2 Hz, CF_3), 124.8 (CH), 123.1 (d, *J* = 2.2 Hz, CH), 18.7 (CH_3).

^{19}F NMR (377 MHz, acetone-d_6) δ -56.0 (s).

HRMS (EI) *m/z*: [M^+] calcd for $C_{17}H_{12}F_3N$ 287.0922, found: 287.0918.

2-(Furan-2-yl)-4-methylquinoline (2k)

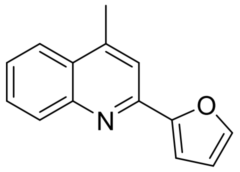

The product was prepared from **1k** according to the general procedure C in 0.20 mmol scale with the reaction time of 2 h and isolated with flash chromatography (silica gel, *n*-Hex:EtOAc 40:1) as a yellow oil (18.8 mg, 0.09 mmol, 44%).

^1H NMR (400 MHz, $CDCl_3$) δ 8.13 (d, *J* = 8.4 Hz, 1H), 7.94 (d, *J* = 8.3 Hz, 1H), 7.72 – 7.59 (m, 3H), 7.50 (ddd, *J* = 8.2, 6.8, 1.3 Hz, 1H), 7.19 (dd, *J* = 3.5, 0.8 Hz, 1H), 6.57 (dd, *J* = 3.5, 1.8 Hz, 1H), 2.72 (d, *J* = 1.0 Hz, 3H).

^{13}C NMR (101 MHz, $CDCl_3$) δ 153.9, 148.8, 148.1, 144.9, 144.1, 130.0, 129.6, 127.4, 126.1, 123.8, 118.1, 112.3, 110.0, 19.0.

The characterization data was in agreement with the previous literature.[12]

4-Methyl-2-(thiophen-2-yl)quinoline (2l)

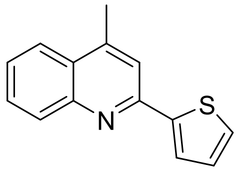

The product was prepared from **1l** according to the general procedure C in 0.20 mmol scale with the reaction time of 2 h and isolated with flash chromatography (silica gel, *n*-Hex:EtOAc 40:1) as a yellow oil (35.4 mg, 0.16 mmol, 79%).

^1H NMR (400 MHz, $CDCl_3$) δ 8.10 – 8.06 (m, 1H), 7.91 (dd, *J* = 8.5, 1.4 Hz, 1H), 7.70 (dd, *J* = 3.7, 1.1 Hz, 1H), 7.66 (ddd, *J* = 8.4, 6.9, 1.4 Hz, 1H), 7.61 (d, *J* = 1.0 Hz, 1H), 7.48 (ddd, *J* = 8.3, 6.9, 1.3 Hz, 1H), 7.44 (dd, *J* = 5.0, 1.1 Hz, 1H), 7.14 (dd, *J* = 5.0, 3.7 Hz, 1H), 2.70 (d, *J* = 0.9 Hz, 3H).

^{13}C NMR (101 MHz, $CDCl_3$) δ 152.1, 148.1, 145.6, 144.8, 129.9, 129.6, 128.4, 128.1, 127.4, 126.0, 125.8, 123.7, 118.4, 19.0.

The characterization data was in agreement with the previous literature.[13]

4-Methyl-2-(pyridin-2-yl)quinoline (2m)

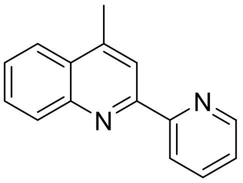

The product was prepared from **1m** according to the general procedure C in 0.21 mmol scale with the reaction time of 15 h and isolated with flash chromatography (silica gel, *n*-Hex:EtOAc 40:1 → 10:1) as a clear oil (18.2 mg, 0.08 mmol, 39%).

^{1}H NMR (400 MHz, acetone-d_6) δ 8.73 (ddd, *J* = 4.8, 1.8, 1.0 Hz, 1H), 8.70 (dt, *J* = 8.0, 1.1 Hz, 1H), 8.51 (d, *J* = 0.9 Hz, 1H), 8.15 – 8.10 (m, 2H), 7.96 (ddd, *J* = 8.0, 7.5, 1.8 Hz, 1H), 7.77 (ddd, *J* = 8.3, 6.8, 1.4 Hz, 1H), 7.62 (ddd, *J* = 8.3, 6.8, 1.3 Hz, 1H), 7.46 (ddd, *J* = 7.4, 4.8, 1.2 Hz, 1H), 2.81 (d, *J* = 1.0 Hz, 3H).

^{13}C NMR (101 MHz, acetone-d_6) δ 157.0, 156.4, 150.0, 148.7, 145.8, 137.7, 131.1, 130.2, 129.1, 127.5, 125.1, 124.9, 122.0, 119.9, 18.9.

The characterization data was in agreement with the previous literature.[15]

4-Methyl-2-(1-methyl-1H-pyrrol-2-yl)quinoline (2n)

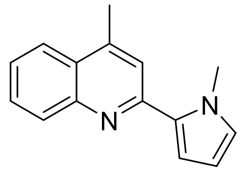

The product was prepared from **1n** according to the general procedure C in 0.21 mmol scale with the reaction time of 15 h and isolated with flash chromatography (silica gel, *n*-Hex:EtOAc [2% Et_3N] 80:1 → 40:1) as a clear oil (4.8 mg, 0.022 mmol, 11%).

^{1}H NMR (400 MHz, $CDCl_3$) δ 8.01 (ddd, *J* = 8.4, 1.3, 0.6 Hz, 1H), 7.93 (dd, *J* = 8.3, 1.1 Hz, 1H), 7.65 (ddd, *J* = 8.4, 6.9, 1.5 Hz, 1H), 7.54 (d, *J* = 1.0 Hz, 1H), 7.47 (ddd, *J* = 8.2, 6.8, 1.3 Hz, 1H), 6.80 – 6.77 (m, 1H), 6.75 (dd, *J* = 3.8, 1.8 Hz, 1H), 6.21 (dd, *J* = 3.9, 2.6 Hz, 1H), 4.18 (s, 3H), 2.69 (d, *J* = 1.0 Hz, 3H).

^{13}C NMR (101 MHz, $CDCl_3$) δ 152.2 (C), 147.7 (C), 143.8 (C), 132.5 (C), 129.8 (CH), 129.2 (CH), 127.5 (CH), 126.5 (C), 125.4 (CH), 123.7 (CH), 120.8 (CH), 112.2 (CH), 107.9 (CH), 37.7 (CH_3), 19.0 (CH_3).

HRMS (EI) *m/z*: [M^+] calcd for $C_{15}H_{14}N_2$ 222.1157, found: 222.1158.

4-Methyl-2-(1H-pyrrol-2-yl)quinoline (2o)

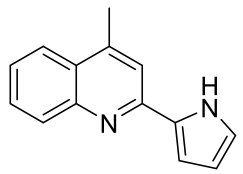

The product was prepared from **1o** according to the general procedure C in 0.20 mmol scale with the reaction time of 15 h and isolated with flash chromatography (silica gel, *n*-Hex:EtOAc [2% Et_3N] 80:1 → 40:1) as a pale yellow solid (11.5 mg, 0.06 mmol, 27%).

^{1}H NMR (400 MHz, acetone-d_6) δ 10.86 (s, 1H), 7.99 (dd, *J* = 8.3, 0.9 Hz, 1H), 7.90 (ddd, *J* = 8.4, 1.4, 0.6 Hz, 1H), 7.72 (d, *J* = 1.1 Hz, 1H), 7.65 (ddd, *J* = 8.4, 6.9, 1.5 Hz, 1H), 7.47 (ddd, *J* = 8.2, 6.9, 1.3 Hz, 1H), 7.03 (td, *J* = 2.6, 1.4 Hz, 1H), 6.94 (ddd, *J* = 3.7, 2.5, 1.5 Hz, 1H), 6.25 (dt, *J* = 3.6, 2.5 Hz, 1H), 2.70 (d, *J* = 1.0 Hz, 3H).

^{13}C NMR (101 MHz, acetone-d_6) δ 151.3 (C), 148.8 (C), 145.1 (C), 132.7 (C), 130.0 (CH), 129.8 (CH), 127.7 (C), 125.8 (CH), 124.8 (CH), 122.1 (CH), 118.8 (CH), 110.5 (CH), 110.0 (CH), 18.7 (CH_3).

HRMS (EI) *m/z*: [M^+] calcd for $C_{14}H_{12}N_2$ 208.1000, found: 208.1008.

2-(*Tert*-butyl)-4-methylquinoline (2p)

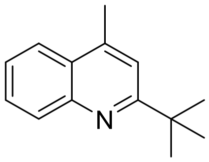

The product was prepared from **1p** according to the general procedure C in 0.28 mmol scale with the reaction time of 15 h and isolated with flash chromatography (silica gel, *n*-Hex:EtOAc [2% Et_3N] 80:1 → 40:1) as a clear oil (20.4 mg, 0.10 mmol, 36%).

^{1}H NMR (400 MHz, acetone-d_6) δ 8.02 (dd, J = 8.3, 1.0 Hz, 1H), 7.98 (ddd, J = 8.4, 1.2, 0.6 Hz, 1H), 7.68 (ddd, J = 8.4, 6.9, 1.4 Hz, 1H), 7.56 – 7.50 (m, 2H), 2.70 (d, J = 1.0 Hz, 3H), 1.44 (s, 9H).

^{13}C NMR (101 MHz, acetone-d_6) δ 169.4, 148.2, 144.7, 130.6, 129.5, 127.4, 126.3, 124.5, 119.6, 38.5, 18.8.

The characterization data was in agreement with the previous literature.[16]

2-Cyclopropyl-4-methylquinoline (2q)

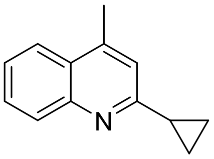

The product was prepared from **1q** according to the general procedure C in 0.22 mmol scale with the reaction time of 15 h and isolated with flash chromatography (silica gel, *n*-Hex:EtOAc [2% Et_3N] 80:1 → 40:1) as a clear oil (12.5 mg, 0.07 mmol, 31%).

^{1}H NMR (400 MHz, acetone-d_6) δ 7.99 (dd, J = 8.3, 0.9 Hz, 1H), 7.85 (ddd, J = 8.4, 1.3, 0.6 Hz, 1H), 7.64 (ddd, J = 8.4, 6.8, 1.5 Hz, 1H), 7.47 (ddd, J = 8.2, 6.8, 1.3 Hz, 1H), 7.25 (d, J = 1.0 Hz, 1H), 2.66 (d, J = 1.0 Hz, 3H), 2.19 (tt, J = 8.1, 4.8 Hz, 1H), 1.16 – 1.11 (m, 2H), 1.03 – 0.98 (m, 2H).

^{13}C NMR (101 MHz, acetone-d_6) δ 163.7, 149.0, 144.4, 130.0, 129.7, 127.7, 125.7, 124.7, 121.9, 18.5, 18.0, 10.5.

The characterization data was in agreement with the previous literature.[16]

2-Phenylquinoline (2r)

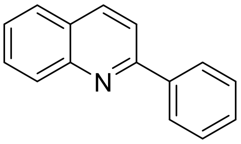

The product was prepared from **1r** according to the general procedure C in 0.21 mmol scale with the reaction time of 15 h and isolated with flash chromatography (silica gel, *n*-Hex:EtOAc [2% Et_3N] 80:1 → 40:1) as a white solid (2.9 mg, 0.01 mmol, 7%).

^{1}H NMR (400 MHz, acetone-d_6) δ 8.43 – 8.39 (m, 1H), 8.34 – 8.30 (m, 2H), 8.11 (d, J = 8.7 Hz, 2H), 7.97 (dd, J = 8.2, 1.4 Hz, 1H), 7.78 (ddd, J = 8.5, 6.9, 1.5 Hz, 1H), 7.61 – 7.46 (m, 4H).

^{13}C NMR (101 MHz, acetone-d_6) δ 157.5, 149.2, 140.3, 137.8, 130.5, 130.4, 130.3, 129.6, 128.6, 128.3, 128.2, 127.2, 119.4.

The characterization data was in agreement with the previous literature.[17]

2-Phenyl-4-cyclopropylquinoline (2s)

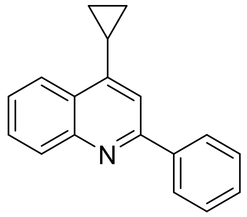

The product was prepared from **1s** according to the general procedure C in 0.21 mmol scale with the reaction time of 1 h and isolated with flash chromatography (silica gel, *n*-Hex:EtOAc [2% Et_3N] 80:1 → 40:1) as a pale yellow oil (41.4 mg, 0.17 mmol, 81%).

^{1}H NMR (400 MHz, acetone-d_6) δ 8.39 (dd, *J* = 8.4, 0.9 Hz, 1H), 8.34 – 8.27 (m, 2H), 8.12 (ddd, *J* = 8.4, 1.3, 0.6 Hz, 1H), 7.75 (ddd, *J* = 8.4, 6.8, 1.4 Hz, 1H), 7.71 (d, *J* = 0.6 Hz, 1H), 7.59 (ddd, *J* = 8.3, 6.8, 1.3 Hz, 1H), 7.57 – 7.42 (m, 3H), 2.56 (ttd, *J* = 8.4, 5.4, 0.8 Hz, 1H), 1.25 – 1.15 (m, 2H), 1.03 – 0.98 (m, 2H).

^{13}C NMR (101 MHz, acetone-d_6) δ 157.3, 151.2, 149.1, 140.5, 131.0, 130.10, 130.05, 129.4, 128.6, 128.2, 126.9, 124.8, 115.0, 12.8, 8.4.

The characterization data was in agreement with the previous literature.[18]

2,4-Diphenylquinoline (2t)

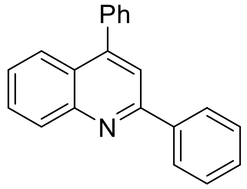

The product was prepared from **1t** according to the general procedure C in 0.20 mmol scale with the reaction time of 1 h and isolated with flash chromatography (silica gel, *n*-Hex:EtOAc [2% Et_3N] 80:1 → 40:1) as a yellow oil (48.7 mg, 0.17 mmol, 86%).

^{1}H NMR (400 MHz, $CDCl_3$) δ 8.28 – 8.21 (m, 1H), 8.21 – 8.16 (m, 2H), 7.89 (dd, *J* = 8.4, 1.3 Hz, 1H), 7.80 (s, 1H), 7.70 (ddd, *J* = 8.4, 6.8, 1.5 Hz, 1H), 7.57 – 7.41 (m, 9H).

^{13}C NMR (101 MHz, $CDCl_3$) δ 157.0, 149.2, 148.9, 139.8, 138.5, 130.2, 129.7, 129.6, 129.4, 128.9, 128.7, 128.5, 127.7, 126.4, 125.9, 125.7, 119.4.

The characterization data was in agreement with the previous literature.[19]

2-(4-Methoxyphenyl)-4-phenylquinoline (2u)

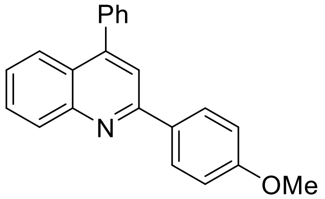

The product was prepared from **1u** according to the general procedure C in 0.20 mmol scale with the reaction time of 1 h and isolated with flash chromatography (silica gel, *n*-Hex:EtOAc [2% Et_3N] 80:1 → 40:1) as a pale yellow oil (50.5 mg, 0.16 mmol, 81%).

^{1}H NMR (400 MHz, acetone-d_6) δ 8.35 – 8.30 (m, 2H), 8.15 (ddd, *J* = 8.4, 1.3, 0.7 Hz, 1H), 7.91 (s, 1H), 7.85 (dd, *J* = 8.4, 0.9 Hz, 1H), 7.73 (ddd, *J* = 8.4, 6.8, 1.4 Hz, 1H), 7.62 – 7.51 (m, 5H), 7.47 (ddd, *J* = 8.3, 6.8, 1.3 Hz, 1H), 7.10 – 7.04 (m, 2H), 3.86 (s, 3H).

^{13}C NMR (101 MHz, acetone-d_6) δ 162.0, 156.7, 149.71, 149.68, 139.2, 132.5, 130.7, 130.4, 130.3, 129.6, 129.5, 129.2, 126.9, 126.23, 126.18, 119.1, 114.9, 55.7.

The characterization data was in agreement with the previous literature.[19]

2-(4-Nitrophenyl)-4-phenylquinoline (2v)

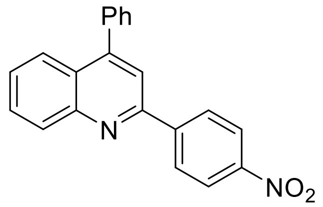

The product was prepared from **1v** according to the general procedure C in 0.07 mmol scale with the reaction time of 2 h and isolated with flash chromatography (silica gel, *n*-Hex:EtOAc [2% Et_3N] 80:1 → 40:1) as a white solid (17.9 mg, 0.05 mmol, 81%).

^1H NMR (400 MHz, $CDCl_3$) δ 8.43 – 8.32 (m, 1H), 8.26 (d, *J* = 8.3 Hz, 0H), 7.94 (dd, *J* = 8.4, 1.4 Hz, 0H), 7.86 (s, 0H), 7.78 (ddd, *J* = 8.4, 6.9, 1.5 Hz, 0H), 7.61 – 7.50 (m, 2H).

^{13}C NMR (101 MHz, $CDCl_3$) δ 154.2, 150.0, 149.0, 148.5, 145.6, 138.1, 130.5, 130.2, 129.7, 128.87, 128.86, 128.5, 127.5, 126.3, 125.9, 124.2, 119.2.

The characterization data was in agreement with the previous literature.[19]

6-Methoxy-2,4-diphenylquinoline (2w)

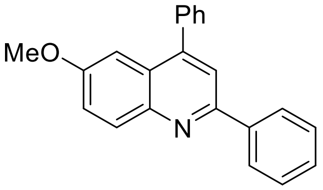

The product was prepared from **1w** according to the general procedure C in 0.11 mmol scale with the reaction time of 2 h and isolated with flash chromatography (silica gel, *n*-Hex:EtOAc [2% Et_3N] 80:1 → 40:1) as a white solid (26.0 mg, 0.08 mmol, 73%).

^1H NMR (400 MHz, $CDCl_3$) δ 8.18 – 8.12 (m, 3H), 7.77 (s, 1H), 7.59 – 7.47 (m, 7H), 7.45 – 7.36 (m, 2H), 7.19 (d, *J* = 2.8 Hz, 1H), 3.79 (s, 3H).

^{13}C NMR (101 MHz, $CDCl_3$) δ 157.9, 154.8, 147.9, 145.0, 139.9, 138.9, 131.8, 129.5, 129.1, 128.9, 128.8, 128.5, 127.4, 126.8, 121.9, 119.8, 103.8, 55.6.

The characterization data was in agreement with the previous literature.[19]

6-Chloro-2,4-diphenylquinoline (2x)

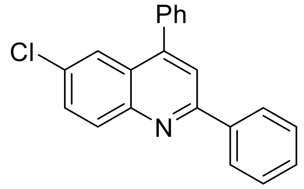

The product was prepared from **1x** according to the general procedure C in 0.16 mmol scale with the reaction time of 1 h and isolated with flash chromatography (silica gel, *n*-Hex:EtOAc [2% Et_3N] 80:1 → 40:1) as a white solid (37.8 mg, 0.12 mmol, 77%).

^1H NMR (400 MHz, $CDCl_3$) δ 8.20 – 8.14 (m, 3H), 7.86 (d, *J* = 2.3 Hz, 1H), 7.83 (s, 1H), 7.65 (dd, *J* = 9.0, 2.4 Hz, 1H), 7.59 – 7.43 (m, 8H).

^{13}C NMR (101 MHz, $CDCl_3$) δ 157.2, 148.6, 147.4, 139.3, 137.9, 132.3, 131.9, 130.6, 129.7, 129.6, 129.03, 128.95, 128.8, 127.7, 126.6, 124.6, 120.2.

The characterization data was in agreement with the previous literature.[19]

6-Bromo-2,4-diphenylquinoline (2y)

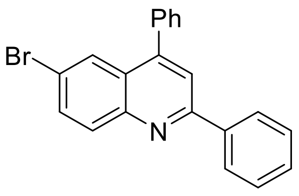

The product was prepared from **1y** according to the general procedure C in 0.12 mmol scale with the reaction time of 2 h and isolated with flash chromatography (silica gel, *n*-Hex:EtOAc [2% Et_3N] 80:1 → 40:1) as a white solid (35.3 mg, 0.10 mmol, 79%).

^{1}H NMR (400 MHz, $CDCl_3$) δ 8.20 – 8.15 (m, 2H), 8.09 (d, *J* = 8.9 Hz, 1H), 8.02 (d, *J* = 2.2 Hz, 1H), 7.82 (s, 1H), 7.78 (dd, *J* = 8.9, 2.2 Hz, 1H), 7.59 – 7.43 (m, 8H).

^{13}C NMR (101 MHz, $CDCl_3$) δ 157.3, 148.5, 147.6, 139.3, 137.8, 133.1, 132.0, 129.8, 129.6, 129.03, 128.96, 128.9, 127.9, 127.7, 127.1, 120.6, 120.1.

The characterization data was in agreement with the previous literature.[19]

6-Fluoro-2,4-diphenylquinoline (2z)

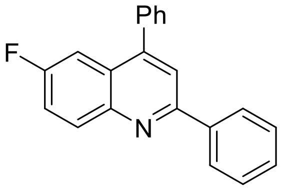

The product was prepared from **1z** according to the general procedure C in 0.09 mmol scale with the reaction time of 2 h and isolated with flash chromatography (silica gel, *n*-Hex:EtOAc [2% Et_3N] 80:1 → 40:1) as a white solid (23.7 mg, 0.08 mmol, 88%).

^{1}H NMR (400 MHz, acetone-d_6) δ 8.38 – 8.32 (m, 2H), 8.23 (dd, *J* = 9.2, 5.7 Hz, 1H), 8.03 (d, *J* = 0.8 Hz, 1H), 7.67 – 7.46 (m, 10H).

^{13}C NMR (101 MHz, acetone-d_6) δ 161.40 (d, *J* = 245.6 Hz), 156.7 (d, *J* = 2.8 Hz), 149.6 (d, *J* = 5.7 Hz), 146.8, 139.9, 138.7, 133.7 (d, *J* = 9.1 Hz), 130.4, 130.3, 129.7, 129.6, 129.6, 128.2, 127.2 (d, *J* = 9.5 Hz), 120.5, 120.3, 109.5 (d, *J* = 23.3 Hz).

^{19}F NMR (377 MHz, acetone-d_6) δ -113.4 (s).

The characterization data was in agreement with the previous literature.[19]

1 mmol scale synthesis of 2a

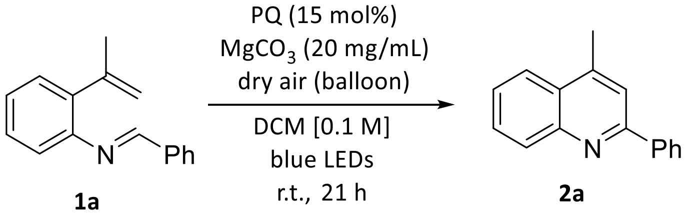

The product was prepared from **1a** according to the general procedure C in 1.0 mmol scale. After 5 h of irradiation, the reaction mixture was bubbled with dry air while the irradiation was continued overnight. The conversion was complete after 21 h, and **2a** was isolated with flash chromatography (silica gel, *n*-Hex:EtOAc 40:1 → 20:1) as a pale yellow oil (172.1 mg, 0.78 mmol, 79%). The decrease in the yield was caused solely by the increased hydrolysis of imine **1a** due to the longer reaction time.

Mechanistic studies

1-Phenyl-*N*-(2-(prop-1-en-2-yl)phenyl)methanimine-*d* (1ah)

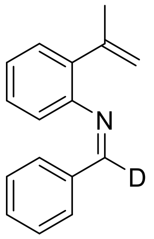

Deuterated imine **1ah** was prepared from 2-(prop-1-en-2-yl)aniline (0.27 mL, 2.0 mmol) and benzaldehyde-d_1 (0.20 mL, 2.0 mmol) according to the general procedure A. Na_2SO_4 (3.0 g, 21 mmol) was used as drying agent. The product was isolated with flash chromatography (silica gel, *n*-Hex:Et_3N 100:3) as a pale yellow oil (354.5 mg, 1.6 mmol, 80%).

^1H NMR (400 MHz, $CDCl_3$) δ 7.92 – 7.86 (m, 2H), 7.49 – 7.45 (m, 3H), 7.32 – 7.26 (m, 2H), 7.18 (td, *J* = 7.5, 1.3 Hz, 1H), 6.96 – 6.93 (m, 1H), 5.16 (dt, *J* = 2.9, 1.5 Hz, 1H), 5.02 (dd, *J* = 2.1, 0.9 Hz, 1H), 2.15 (dd, *J* = 1.5, 0.8 Hz, 3H).

^{13}C NMR (101 MHz, $CDCl_3$) δ 159.7 (t, *J* = 24.3 Hz, CD), 150.0 (C), 144.9 (C), 137.2 (C), 136.5 (C), 131.4 (CH), 129.0 (CH), 128.93 (CH), 128.90 (CH), 128.2 (CH), 125.6 (CH), 118.8 (CH), 116.4 (CH_2), 23.9 (CH_3).

HRMS (EI) *m/z*: [M^+] calcd for $C_{16}H_{14}DN$ 222.1267, found: 222.1275.

Kinetics experiment

The reactions were done according to the general procedure C in 0.10 mmol scale using imines **1a** and **1ah** as starting materials. A separate reaction was carried out for each time point with both imines. A stock solution of 1,3,5-trimethoxybenzene (TMB) in DMSO (0.48 M) was prepared in a volumetric flask and used as an internal standard for the NMR yield measurements. The difference in the final yields between the two experiments was caused solely by the hydrolysis of imine **1ah**.

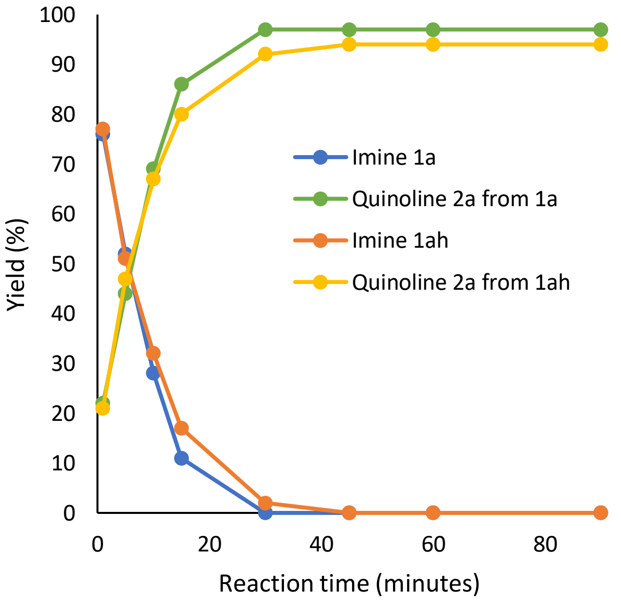

Figure S3. Kinetic measurements with imines **1a** and **1ah**.

UV–vis spectroscopic measurements

A 10 × 10 mm light path glass cuvette was loaded with a solution of PQ in DCM (0.1 mM). The UV–vis spectra of PQ were measured with imine **1a** (0.1 mM), $MgCO_3$ (5.0 mg) and the combination of the two. The UV–vis spectra of imine **1a** (0.1 mM) and imine **1a** with $MgCO_3$ (5.0 mg) were also measured. The visible light absorption maximum of PQ was found at 415 nm in all samples. $MgCO_3$ did not have any effect on the absorption maxima or the absorption intensity of PQ or imine **1a**.

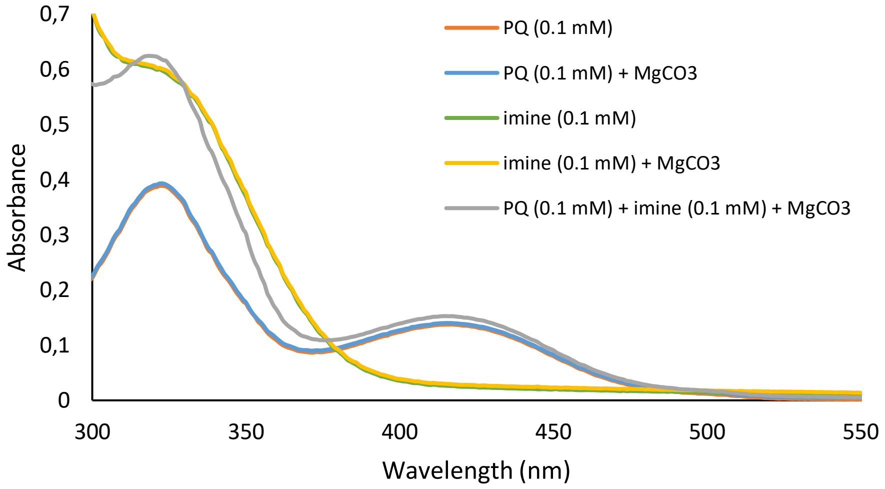

Figure S4. The UV–vis spectra of PQ with varying components.

Attempted detection of H_2O_2

The detection of H_2O_2 possibly forming in the reaction was done by using aqueous $Ti(O)SO_4$ as has been previously described in literature[20]. A reaction was performed according to the general procedure C in 0.10 mmol scale using imine **1a** as starting material. After 30 min irradiation, the reaction mixture was diluted to 10.0 mL with DCM and 6.0 mL H_2O was added. A 2.5 mL aliquot of the aqueous layer was added to a 10 × 10 mm light path glass cuvette and the UV–vis spectrum was measured. $Ti(O)SO_4$ (0.10 mL, 15 wt% in dilute H_2SO_4) was added to this solution and the UV–vis spectrum was measured. Additionally, the spectrum was measured of a sample with 2.5 mL H_2O and 0.10 mL of $Ti(O)SO_4$ solution. No change was obtained between the spectra of the reaction mixture with and without $Ti(O)SO_4$ solution indicating that the reaction does not produce H_2O_2.

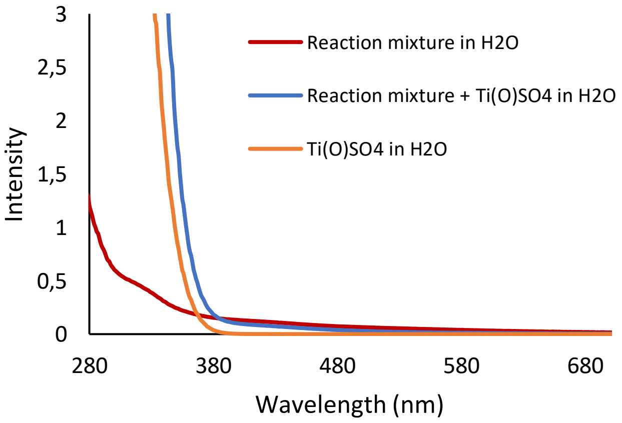

Figure S5. UV–vis spectra of H_2O_2 detection samples.

Radical trapping by TEMPO

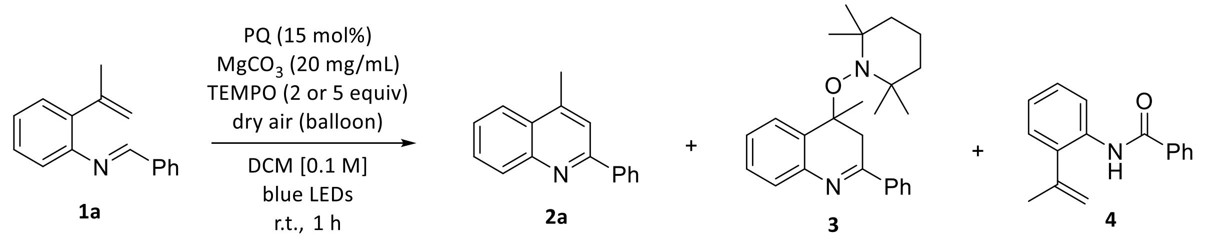

2a: 2 equiv TEMPO: 35%; 5 equiv TEMPO: 13%
3: 2 equiv TEMPO: 16%; 5 equiv TEMPO: 4%
4: 2 equiv TEMPO: trace; 5 equiv TEMPO: 10%

The reaction was performed according to the general procedure C in 0.20 mmol scale with the reaction time of 1 h. (2,2,6,6-Tetramethylpiperidin-1-yl)oxyl (TEMPO, 63.3 mg, 0.41 mmol, 2 equiv) was added in the reaction mixture. The crude was purified with flash chromatography (silica gel, *n*-Hex:EtOAc [2% Et_3N] 80:1 → 40:1 and then *n*-Hex:EtOAc 20:1 → 10:1). The yield of **2a** was 35% (15.5 mg, 0.071 mmol). Additionally, TEMPO-adduct **3** was isolated (11.8 mg, 0.031 mmol, 16%). A trace amount of amide **4** was also identified.

Another reaction was performed in the same way in 0.23 mmol scale using 5 equiv of TEMPO (176.2 mg, 1.1 mmol). The NMR yields of **2a**, **3**, and **4** were 13%, 4% and 10%, respectively.

4-Methyl-2-phenyl-4-((2,2,6,6-tetramethylpiperidin-1-yl)oxy)-3,4-dihydroquinoline (3)

^{1}H NMR (400 MHz, $CDCl_3$) δ 8.16 (dd, *J* = 6.6, 3.0 Hz, 2H), 7.69 (d, *J* = 7.6 Hz, 1H), 7.49 (d, *J* = 7.2 Hz, 1H), 7.47 – 7.44 (m, 3H), 7.37 (td, *J* = 7.6, 1.2 Hz, 1H), 7.25 (td, *J* = 7.8, 1.3 Hz, 1H), 4.24 (d, *J* = 8.4 Hz, 1H), 3.99 (d, *J* = 8.4 Hz, 1H), 1.72 (s, 3H), 1.53 – 1.17 (m, 6H), 1.01 (s, 3H), 0.92 (s, 3H), 0.82 (s, 3H), 0.74 (s, 3H).

^{13}C NMR (101 MHz, $CDCl_3$) δ 180.2 (C), 153.6 (C), 144.1 (C), 133.6 (C), 130.1 (CH), 128.1 (CH), 128.0 (CH), 127.6 (CH), 125.0 (CH), 122.2 (CH), 120.3 (CH), 79.3 (CH_2), 59.8 (C), 59.6 (C), 58.1 (C), 39.4 (CH_2), 32.4 (CH_3), 32.3 (CH_3), 19.9 (CH_3), 19.7 (CH_3), 19.6 (CH_3), 16.5 (CH_2).

HRMS (EI) *m/z*: [M^+] calcd for $C_{25}H_{32}N_2O$ 376.2515, found: 376.2504.

***N*-(2-(Prop-1-en-2-yl)phenyl)benzamide (4)**

^{1}H NMR (400 MHz, $CDCl_3$) δ 8.50 (d, *J* = 8.3 Hz, 1H), 8.45 (br s, 1H), 7.86 – 7.80 (m, 2H), 7.57 – 7.52 (m, 1H), 7.52 – 7.47 (m, 2H), 7.35 – 7.31 (m, 1H), 7.19 (dd, *J* = 7.6, 1.7 Hz, 1H), 7.12 (td, *J* = 7.5, 1.1 Hz, 1H), 5.48 (p, *J* = 1.6 Hz, 1H), 5.12 (dd, *J* = 2.0, 1.0 Hz, 1H), 2.13 – 2.12 (m, 3H).

^{13}C NMR (101 MHz, $CDCl_3$) δ 164.7, 142.9, 134.8, 133.7, 133.1, 131.4, 128.5, 127.7, 127.3, 126.6, 123.5, 120.3, 116.5, 24.3.

The characterization data was in agreement with the previous literature.[21]

Computational studies

DFT computational studies were performed with Gaussian16 package.[22] Geometry optimizations were performed with pbe0,[23] def2-svp basis set including Grimme empirical dispersion = gd3bj[23] and the CPCM solvation model for dichloromethane (298.15 K and 1 atm pressure). All the optimized stationary points related to the transition states showed a single imaginary frequency. These geometries were confirmed to connect the reactants and the products by intrinsic reaction coordinate (IRC) calculations. Single point electronic energy and frequency calculations were performed with pw6b95d3[25] def2-tzvp[26] basis set with the implicit solvation model CPCM[27] to access refined thermal energies. The 3D images were created using CYLview.[28] We used the same methodology in our previous publication for the computation of thermal electrocyclization and quinoidic dehydrogenation pathways of similar imine substrates.[9] In the previous study, the electrocyclization step was identified as the rate-limiting step, with energy barriers above 25 kcal/mol, whereas the barriers for thermal quinoidic dehydrogenations of dihydroquinolines were below 13 kcal/mol.

The HAT and radical cation catalyzed reaction profiles for the cyclizations of imines **1a**, **1b**, **1f**, **1r** and **1aa** are compared in Figure S6 (optimized geometry in Figure S7; single point energies in Figure 1 and Table S9). Very low cyclization barriers (1.1–7.7 kcal/mol) were obtained for all neutral radicals. However, they do not match with the experimental observations. Particularly, they fail to explain the poor reactivity of **1r** and no reactivity of **1aa**. Instead, the radical cation mediated routes are in agreement with the performed experiments. The cyclization barriers for the experimentally reactive substrates **1a**, **1b** and **1f** are still rather low being in the range of 9.2–11.5 kcal/mol. For **1r** and **1aa**, these barriers are 17.2 and 19.8 kcal/mol, respectively. Notably, the cyclization step is endergonic for these substrates. Hence, the radical cation mediated energy profiles offer a good explanation for the experimental observations considering the reversible nature of the cyclization step.

The oxidation potentials ($E^{ox}_{½}$) of the studied imines (Table S8) are clearly below the excited state reduction potential ($E^{red}_{½}$) of PQ* (ca. 1.6 V vs. SCE). The imine oxidation potentials were calculated by applying the methodology developed by Nicewicz.[29]

Table S8. Computed oxidation potentials $E^{ox}_{½}$) with (B3LYP 6-31+G(d,p)) CPCM MeCN for selected imines.

	**1a**	**1b**	**1f**	**1h**	**1c**	**1z**	**1w**	**1t**	**1aa**	**1p**	**1m**	**1k**	**1r**
**Radical cation (a.u.)**	-673.086	-803.645	-765.334	-877.596	-877.595	-964.036	-979.299	-864.787	-864.807	-599.255	-689.132	-670.89	-633.795
**Neutral (a.u.)**	-673.298	-803.856	-765.55	-877.812	-877.813	-964.25	-979.501	-865.000	-865.010	-599.465	-689.347	-671.100	-634.006
ΔG (kcal/mol)	132.95	132.42	135.95	135.68	137.12	134.29	126.72	133.43	126.99	132.20	135.19	131.99	132.25
**ΔG/*F*** (V)	5.77	5.74	5.90	5.88	5.95	5.82	5.49	5.79	5.51	5.73	5.86	5.72	5.73
$E^{o}_{½}$ (V vs. SHE)	1.48	1.46	1.61	1.60	1.66	1.54	1.21	1.50	1.23	1.45	1.58	1.44	1.45
**$E^{o}_{½}$ (V vs. SCE)**	**1.34**	**1.32**	**1.47**	**1.46**	**1.52**	**1.40**	**1.07**	**1.36**	**1.08**	**1.31**	**1.44**	**1.30**	**1.31**
**$E^{o}_{½}$= (**V vs. Fc)	0.84	0.82	0.97	0.96	1.02	0.90	0.57	0.86	0.59	0.81	0.94	0.80	0.81

F = 23,061 kcal/(mol*V)

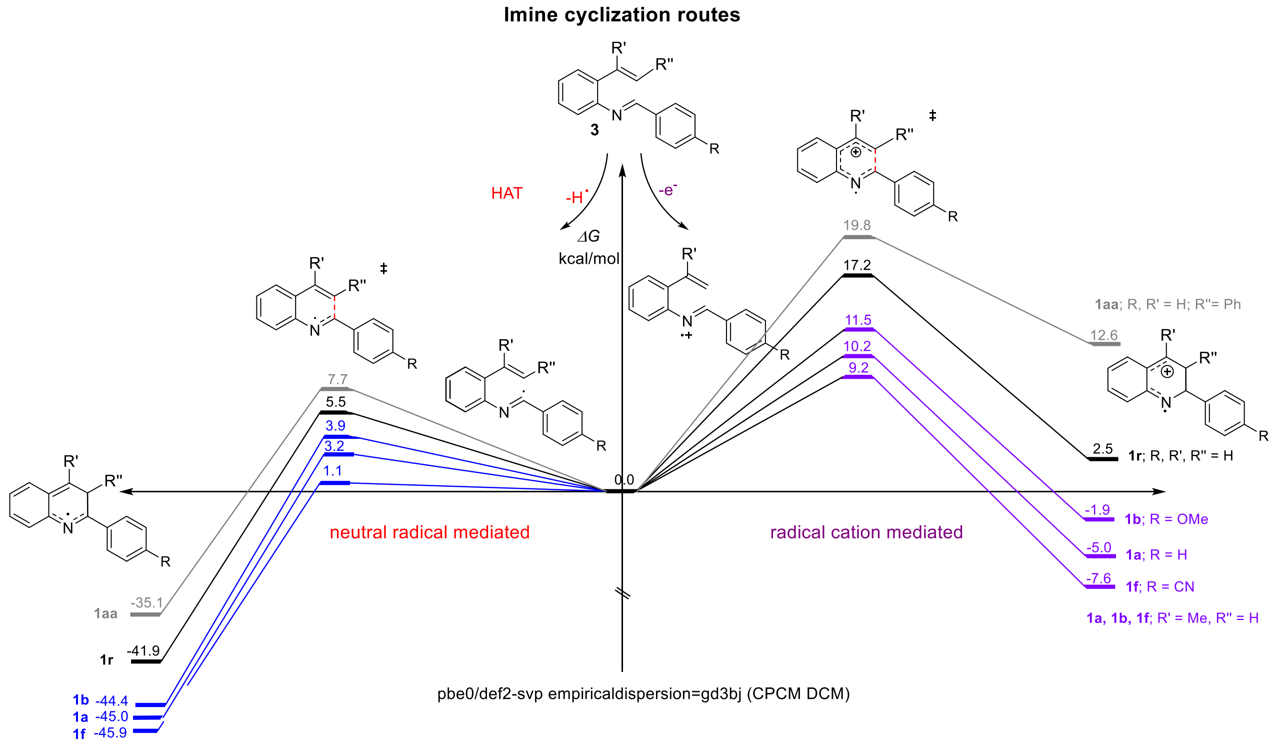

Figure S6. The electrocyclization step via HAT catalysis (left) and hole catalysis (right); cyclization free energy (*ΔG*) barriers in kcal/mol starting from iminium neutral radical and iminium radical cation, respectively.

Table S9. Computed single point energies for **1a**, **1b**, **1f**, **1r** and **1aa** with method pw6b95d3/def2-tzvp//pbe0-D3bj/def2-svp (CPCM DCM)) related to Figure 1.

	**1a**						
	neutral radical			radical cation			
	nR-Int	TS-nR	SM-nR	INT2	INT1-**TS**-INT2	INT1	SM
free energy (a.u.)	-673.79223	-673.724453	-673.731	-674.167172	-674.147715	-674.16584	-674.383
ΔG (kcal/mol)	-38.37	4.16	88.20 (BDE)*	-0.84	11.37	136.02	0
	**1b**						
	neutral radical			radical cation			
	nR-Int	TS-nR	SM-nR	INT2	INT1-**TS**-INT2	INT1	SM
free energy (a.u.)	-788.47	-788.4	-788.409	-788.844	-788.828	-788.84715	-789.06
ΔG (kcal/mol)	-37.97	4.43	87.92 (BDE)*	1.90	12.23	133.83	0
	**1f**						
	neutral radical			radical cation			
	nR-Int	TS-nR	SM-nR	INT2	INT1-**TS**-INT2	INT1	SM
free energy (a.u.)	-766.197	-766.132	-766.134	-766.57	-766.547	-766.563915	-766.786
ΔG (kcal/mol)	-39.31	1.51	88.48 (BDE)*	-3.66	10.56	139.47	0
	**1r**						
	neutral radical			radical cation			
	nR-Int	TS-nR	SM-nR	INT2	INT1-**TS**-INT2	INT1	SM
free energy (a.u.)	-634.434584	-634.367097	-634.377	-634.801693	-634.782685	-634.810876	-635.028908
ΔG (kcal/mol)	-36.13	6.21	88.45 (BDE)*	5.76	17.70	136.82	0
	**1aa**						
	neutral radical			radical cation			
	nR-Int	TS-nR	SM-nR	INT2	INT1-**TS**-INT2	INT1	SM
free energy (a.u.)	-865.807133	-865.747023	-865.760041	-866.175783	-866.168001	-866.202347	-866.411915
ΔG (kcal/mol)	-29.55	8.17	88.43 (BDE)*	16.70	21.55	131.51	0

* Bond dissociation energy of imine N=C–HR (BDE) connected to HAT mechanism (ΔG = (SM-nR + H•) – SM); H• = -0.51096 kcal/mol

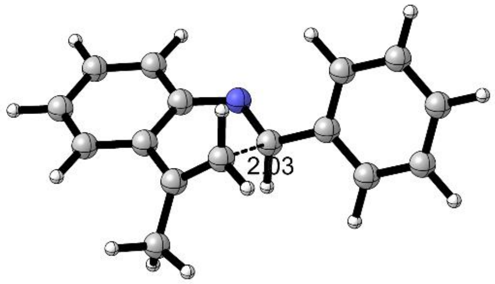

rc-1a-TS

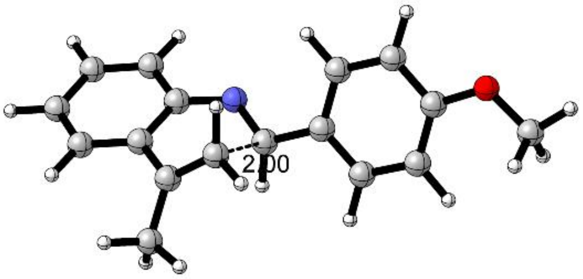

rc-1b-TS

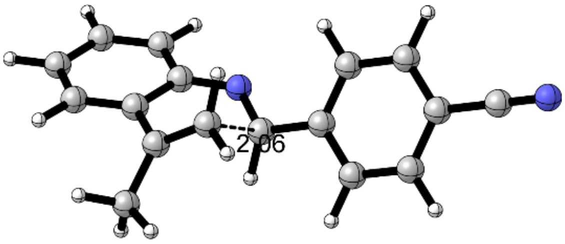

rc-1f-TS

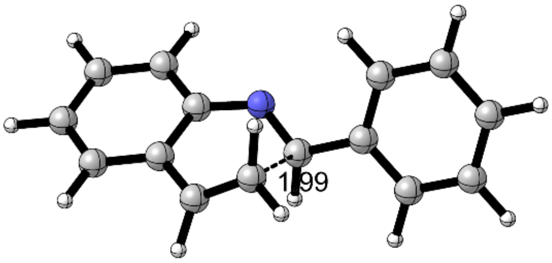

rc-1r-TS

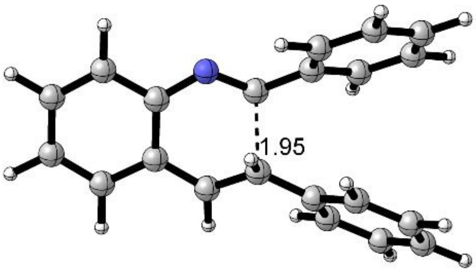

rc-1aa-TS

Figure S7. TS geometries of the radical cation cyclization related to ∆G illustrated in Figures 1 and S6.

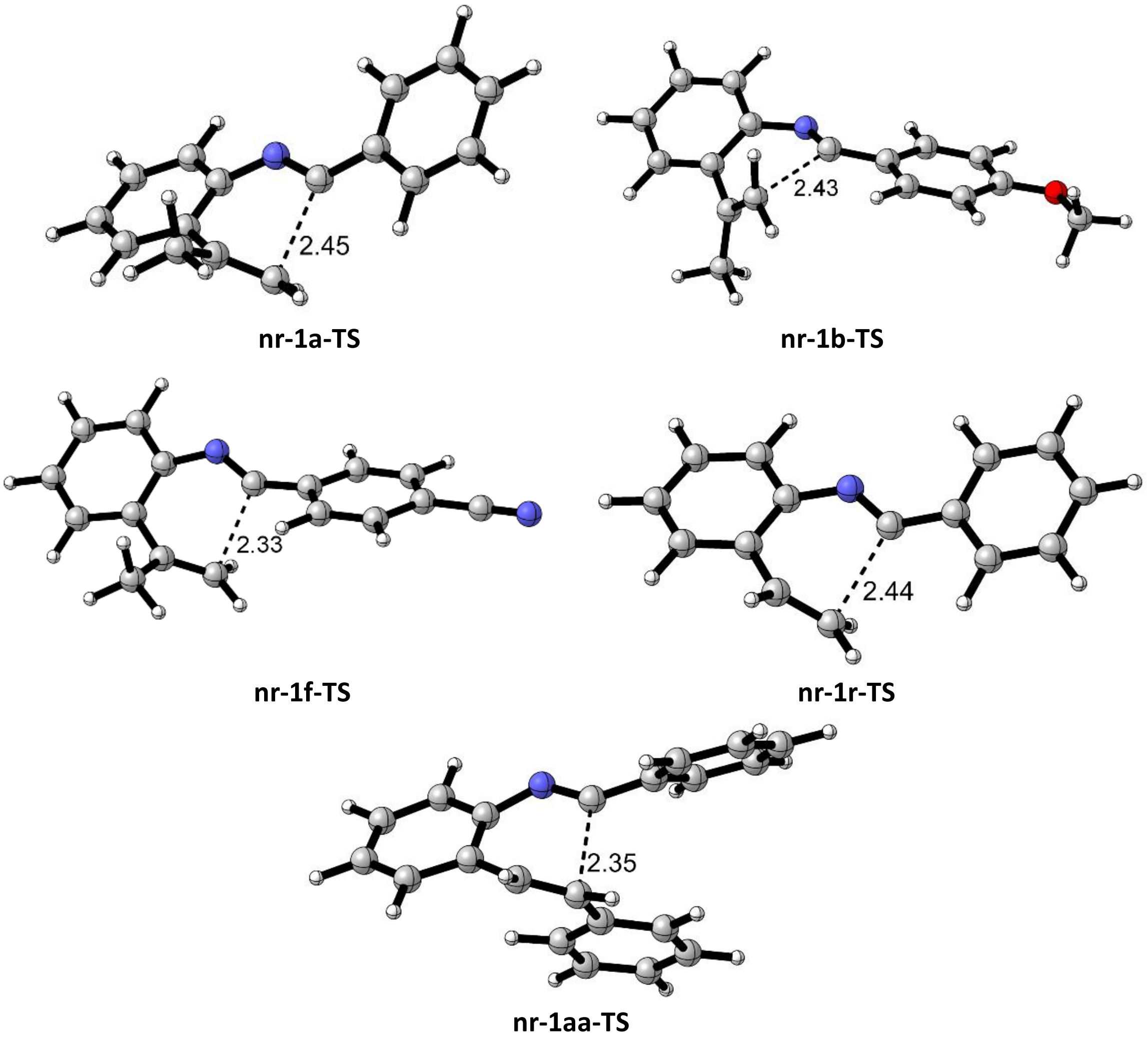

Figure S8. TS geometries of the neutral radical cyclization related to ΔG illustrated in Figures 1 and S6.

pK_a calculations were done according to the following equation:

$$\mathrm{p}K\mathrm{a} \; = \; \frac{\Delta G}{RT \ln 10} \approx \frac{\Delta G}{1{,}364}$$

ΔG is the acid dissociation free energy: HA → H^+ + A^-; *R* is the gas constant and *T* is the temperature in Kelvins.

(free energy of neutral radical – free energy of radical cation) + H^+ (solvation in DCE = -207.7)[30] kcal/mol

The calculation was benchmarked to the experimental pK_a value of TfOH in dichloroethane reported by Leito and co-workers.[30]

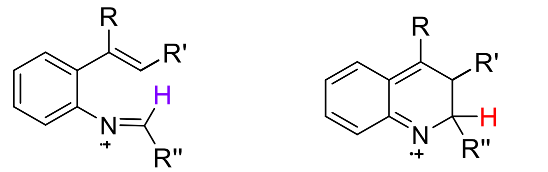

pK_a = INT1 (DCM)   pK_a = INT2 (DCM)

Table S10. Computed pK_as in DCM (pw6b95d3/def2-tzvp//pbe0/def2-svp empirical dispersion=gd3bj (CPCM DCM)).

	**1a**	**1b**	**1f**	**1r**	**1aa**	**PQ$^{\bullet-}$** (semiquinone radical anion)	**TfOH** (benchmark)
pK_a INT1	48	49	45	47	51		
pK_a INT2	20	20	19	17	17		
pK_{aH}						61	
pK_a							36 (33.7)*

* Experimental DCE[30]

XYZ-parameters and energies for TS structures

E opt/au = pbe0/def2-svp empirical dispersion=gd3bj (CPCM DCM): The SCF energy of the molecule at the optimization level of theory (in hartrees)
E/au = single point (sp) frequency calculation with pw6b95d3/def2-tzvp (CPCM DCM) (in hartrees)
ZPE/au= Zero-point correction (sp in hartrees)
H/au = sum of electronic and thermal enthalpies (sp in hartrees)
DG/au = sum of electronic and thermal free (sp in hartrees)
i-freq = The imaginary frequency for a transition state in cm^{-1}

**rc-1a-TS**			
E opt = -671.99263612			
i-freq =-461.5			
E/au = -674.37042492			
ZPE/au = -674.108198			
H/au = -674.094326			
DG/au = -674.147715			
32			
C	2.10882	0.44614	0.20840
C	3.48912	0.58628	0.34802
C	4.33957	-0.50131	0.15075
H	5.41418	-0.37771	0.29621
C	1.57133	-0.83971	-0.20515
C	2.46485	-1.92535	-0.40291
H	3.90423	1.54574	0.66216
C	3.82265	-1.75348	-0.22435
H	2.04345	-2.88099	-0.71714
H	4.50096	-2.59430	-0.38145
N	0.27531	-1.00250	-0.45131
C	1.17621	1.54120	0.38550
C	-0.06867	1.25467	0.93710
C	-0.58098	0.02946	-0.59771
H	-0.81966	2.04531	1.00379
H	-0.16833	0.43012	1.64685
C	-2.00741	-0.14029	-0.29486
C	-2.44651	-1.19537	0.52001
C	-2.93469	0.76814	-0.82699
C	-3.80035	-1.33721	0.79456
H	-1.71833	-1.90632	0.91652
C	-4.28866	0.62232	-0.54588
H	-2.59016	1.58794	-1.46264
C	-4.72117	-0.42797	0.26537
H	-4.14503	-2.16267	1.42075
H	-5.01074	1.32676	-0.96342
H	-5.78518	-0.54307	0.48426
C	1.52476	2.89295	-0.11394
H	1.93698	2.85250	-1.13297
H	0.66979	3.57812	-0.07083
H	2.32247	3.30593	0.52971
H	-0.37506	0.68735	-1.46012

**rc-1b-TS**			
E opt = -786.31687059			
i-freq =-494.6			
E/au = -788.785431			
ZPE/au = -788.785431			
H/au = -788.769265			
DG/au = -788.827668			
36			
C	-2.90920	0.44115	-0.21702
C	-4.29295	0.57343	-0.34924
C	-5.13524	-0.51848	-0.15085
H	-6.21133	-0.40212	-0.29137
C	-2.36056	-0.83944	0.19776
C	-3.24822	-1.93185	0.39188
H	-4.71456	1.53081	-0.66115
C	-4.60732	-1.76900	0.21735
H	-2.81980	-2.88531	0.70345
H	-5.27921	-2.61531	0.37318
N	-1.06697	-0.99901	0.46019
C	-1.98600	1.53704	-0.40777
C	-0.72284	1.23827	-0.91726
C	-0.21650	0.05098	0.60618
H	0.02280	2.03353	-0.98857
H	-0.61436	0.41463	-1.62694
C	1.20780	-0.11591	0.34452
C	1.68149	-1.15567	-0.48047
C	2.12880	0.78289	0.90275
C	3.03061	-1.29060	-0.72696
H	0.97149	-1.86703	-0.90815
C	3.48880	0.65918	0.65921
H	1.77330	1.59466	1.54275

C	3.95146	-0.38228	-0.16312
H	3.41675	-2.09752	-1.35205
H	4.18176	1.36744	1.11215
C	-2.35218	2.90270	0.03929
H	-2.78093	2.89467	1.05238
H	-1.50042	3.59135	-0.01160
H	-3.14045	3.29052	-0.63092
O	5.23242	-0.59126	-0.45869
C	6.22023	0.26952	0.06785
H	7.18148	-0.09653	-0.31016
H	6.06855	1.30680	-0.27103
H	6.22924	0.24134	1.16919
H	-0.44401	0.70963	1.46253

**rc-1f-TS**			
E opt = -764.06121914			
i-freq = -427.6			
E/au = -766.76637711			
ZPE/au = -766.505387			
H/au = -766.489831			
DG/au = -766.547085			
33			
C	-2.73925	0.39711	-0.25500
C	-4.11451	0.47893	-0.46337
C	-4.93118	-0.63752	-0.27940
H	-6.00152	-0.55907	-0.47799
C	-2.17468	-0.85907	0.21148
C	-3.03277	-1.97441	0.39497
H	-4.55154	1.41362	-0.81949
C	-4.38636	-1.85957	0.15031
H	-2.59028	-2.90558	0.75078
H	-5.03919	-2.72213	0.29670
N	-0.88327	-0.96310	0.50902
C	-1.84212	1.52733	-0.41094
C	-0.57213	1.29142	-0.92471
C	-0.07830	0.09704	0.67840
H	0.14803	2.11155	-0.97572
H	-0.40875	0.45443	-1.60753
C	1.36610	-0.02083	0.42232
C	1.87063	-1.09134	-0.33009
C	2.23665	0.94879	0.93883
C	3.23202	-1.19165	-0.56734
H	1.18639	-1.85090	-0.71288
C	3.60061	0.85684	0.70368
H	1.84345	1.78065	1.52766
C	4.10034	-0.21412	-0.05286
H	3.63191	-2.02487	-1.14667
H	4.28269	1.60719	1.10521
C	-2.26075	2.86513	0.07134
H	-2.67809	2.81180	1.08803
H	-1.44081	3.59167	0.02882
H	-3.07514	3.23131	-0.57927
H	-0.34289	0.76927	1.51276
C	5.50767	-0.31441	-0.29836
N	6.64669	-0.39503	-0.49844

**rc-1r-TS**			
E opt = -632.73870620			
i-freq =-584.5			
E/au = -634.97876421			
ZPE/au = -634.744557			
H/au = -634.731994			
DG/au = -634.782685			
29			
C	2.19992	0.77812	0.18738
C	3.58119	1.01227	0.23167
C	4.47819	-0.03319	0.05079
H	5.55082	0.15434	0.12329
C	1.71568	-0.55718	-0.14352
C	2.66151	-1.60006	-0.32228
H	3.94413	2.01489	0.46608
C	4.01220	-1.33597	-0.21943
H	2.28849	-2.59390	-0.57229
H	4.73170	-2.14420	-0.36539
N	0.42835	-0.78460	-0.36703
C	1.23213	1.80312	0.37766
C	-0.04687	1.51504	0.84608
C	-0.47790	0.19752	-0.58317
H	-0.80124	2.30489	0.85597
H	-0.16950	0.73213	1.59993
H	-0.29154	0.79986	-1.49028
C	-1.89918	-0.02534	-0.29313
C	-2.85787	0.80197	-0.89793
C	-2.30373	-1.04668	0.58129
C	-4.20864	0.60891	-0.63089
H	-2.53886	1.59588	-1.57811
C	-3.65418	-1.23611	0.84103
H	-1.55141	-1.69599	1.03441
C	-4.60655	-0.40748	0.23895
H	-4.95478	1.24984	-1.10454
H	-3.97225	-2.03661	1.51205
H	-5.66797	-0.56006	0.44716
H	1.47847	2.82150	0.05393

**rc-1aa-TS**			
E opt = -863.38495591			
i-freq =-579.1			
E/au = -866.43977449			
ZPE/au = -866.124772			
H/au = -866.107883			
DG/au = -866.168001			
39			
C	2.68496	-0.78428	-0.03403
C	3.91178	-1.48734	-0.01407
C	5.10755	-0.79939	-0.04827
H	6.05214	-1.34533	-0.06675
C	2.69672	0.67576	0.00790
C	3.94819	1.34548	-0.04868
H	3.89567	-2.57895	-0.03324
C	5.11721	0.61708	-0.07485
H	3.95060	2.43575	-0.02725
H	6.07489	1.14156	-0.10141
N	1.58234	1.36497	0.18438
C	1.44916	-1.43991	-0.07087
C	0.25940	-0.78559	-0.49581
C	0.43552	0.78198	0.64350
H	0.38797	-0.15431	-1.38488
C	-0.85076	1.45952	0.44776
C	-1.03388	2.36962	-0.60435

C	-1.92358	1.14514	1.29453
C	-2.27816	2.95329	-0.80455
H	-0.18995	2.61971	-1.25116
C	-3.16759	1.73183	1.08972
H	-1.78001	0.43268	2.11032
C	-3.34673	2.63151	0.03830
H	-2.42013	3.66691	-1.61882
H	-4.00122	1.48573	1.75036
H	-4.32450	3.09056	-0.12467
H	0.54029	0.30284	1.63373
H	1.40553	-2.48536	0.25464
C	-1.05829	-1.41047	-0.37856
C	-2.06456	-1.05096	-1.29008
C	-1.36425	-2.30553	0.66000
C	-3.34289	-1.58486	-1.17570
H	-1.83686	-0.34198	-2.08989
C	-2.64366	-2.83812	0.77173
H	-0.60494	-2.57566	1.39843
C	-3.63518	-2.47859	-0.14409
H	-4.11726	-1.29989	-1.89092
H	-2.87324	-3.53228	1.58286
H	-4.64107	-2.89380	-0.04933

**nr-1a-TS**			
E opt = -671.55519583			
i-freq =-213.2			
E/au = -673.93464454			
ZPE/au = -673.684689			
H/au = -673.670723			
DG/au = -673.724453			
31			
C	2.23121	0.45326	-0.04865
C	3.63029	0.45864	0.05863
C	4.37074	-0.71815	0.02842
H	5.46092	-0.67837	0.08408
C	1.56747	-0.80432	-0.10891
C	2.32641	-1.98693	-0.11677
H	4.14719	1.41899	0.13217
C	3.71298	-1.94794	-0.07252
H	1.79100	-2.93832	-0.14765
H	4.28449	-2.87871	-0.09639
N	0.18886	-0.92771	-0.02200
C	1.45212	1.70330	-0.03720
C	0.50716	1.90123	-0.99070
C	-0.70419	-0.13570	-0.38407
H	0.49009	1.30483	-1.90385
H	-0.13521	2.78667	-0.96802
C	-2.13781	-0.19332	-0.15429
C	-2.73701	-1.37807	0.31395
C	-2.94249	0.92387	-0.42314
C	-4.11234	-1.43643	0.50468
H	-2.10140	-2.24214	0.51959
C	-4.31835	0.86363	-0.21759
H	-2.47502	1.84012	-0.78972
C	-4.90541	-0.31555	0.24260
H	-4.57284	-2.35987	0.86427
H	-4.93751	1.73997	-0.42232
H	-5.98604	-0.36383	0.39663
C	1.65322	2.64523	1.11034

H	1.42986	2.15170	2.07126
H	2.70198	2.98055	1.16912
H	1.01646	3.53586	1.01619

**nr-1b-TS**			
E opt = -785.87691853			
i-freq =-224.6			
E/au = -788.64245717			
ZPE/au = -788.359711			
H/au = -788.343411			
DG/au = -788.402298			
35			
C	-3.03006	0.48611	-0.01118
C	-4.42329	0.52743	0.14767
C	-5.20182	-0.62332	0.07984
H	-6.28750	-0.55488	0.17900
C	-2.40697	-0.78464	-0.16667
C	-3.20435	-1.94112	-0.21536
H	-4.90611	1.49730	0.29415
C	-4.58666	-1.86377	-0.11569
H	-2.70122	-2.90478	-0.32117
H	-5.18764	-2.77454	-0.17082
N	-1.03149	-0.95198	-0.13725
C	-2.20852	1.70800	0.03909
C	-1.29222	1.92520	-0.93845
C	-0.12619	-0.16138	-0.47918
H	-0.61158	2.78052	-0.89037
H	-1.33695	1.38492	-1.88501
C	1.30140	-0.24080	-0.24890
C	1.86630	-1.33215	0.44562
C	2.15482	0.76488	-0.71927
C	3.22985	-1.40734	0.65189
H	1.20409	-2.11834	0.81508
C	3.52964	0.70326	-0.51227
H	1.72808	1.61501	-1.25622
C	4.07716	-0.38864	0.17603
H	3.67889	-2.24749	1.18574
H	4.16463	1.50482	-0.88918
C	-2.32639	2.58474	1.24820
H	-3.36014	2.94624	1.37691
H	-2.07502	2.02814	2.16702
H	-1.66635	3.46053	1.17674
O	5.38460	-0.54505	0.42635
C	6.29076	0.43543	-0.02086
H	7.28822	0.10354	0.29090
H	6.27114	0.53390	-1.11891
H	6.08030	1.41806	0.43301

**nr-1f-TS**			
E opt = -763.63129069			
i-freq =-342.6			
E/au = -766.33813891			
ZPE/au = -766.089883			
H/au = -766.074158			
DG/au =-766.131820			
32			
C	-2.81643	0.46052	0.17636
C	-4.20693	0.64575	0.21810
C	-5.08631	-0.41492	0.03366

H	-6.16320	-0.24699	0.10454
C	-2.32598	-0.83416	-0.14900
C	-3.22162	-1.89268	-0.35916
H	-4.59927	1.64107	0.44056
C	-4.59015	-1.69323	-0.24286
H	-2.81422	-2.86997	-0.62561
H	-5.27602	-2.53049	-0.38943
N	-0.98015	-1.05636	-0.42248
C	-1.86933	1.56094	0.39200
C	-0.79995	1.34085	1.20442
C	0.01858	-0.44621	-0.04353
H	-0.80393	0.51980	1.92523
H	-0.02239	2.09921	1.33204
C	1.41878	-0.36065	-0.09772
C	2.21393	-1.40085	0.45690
C	2.06490	0.79510	-0.61303
C	3.58958	-1.29246	0.47613
H	1.72813	-2.28976	0.86261
C	3.44028	0.89341	-0.59416
H	1.46339	1.60500	-1.02920
C	4.22437	-0.14647	-0.04825
H	4.19284	-2.10035	0.89443
H	3.92858	1.77969	-1.00327
C	-2.04503	2.82091	-0.39603
H	-2.02793	2.61772	-1.47961
H	-3.02196	3.28469	-0.18091
H	-1.26156	3.55401	-0.16041
C	5.64412	-0.03866	-0.02597
N	6.80311	0.04969	-0.00869

**nr-1r-TS**			
E opt = -632.30907854			
i-freq = -232.6			
E/au = -634.55070168			
ZPE/au = -634.328715			
H/au = -634.316057			
DG/au = -634.367097			
28			
C	-2.33574	0.75021	-0.15454
C	-3.72893	0.78269	-0.33984
C	-4.51186	-0.35514	-0.19179
H	-5.59548	-0.29192	-0.31440
C	-1.72226	-0.51119	0.09234
C	-2.52560	-1.65672	0.22375
H	-4.19945	1.74158	-0.57362
C	-3.90640	-1.58007	0.11160
H	-2.02729	-2.61269	0.39887
H	-4.51334	-2.48029	0.23224
N	-0.34867	-0.70001	0.05105
C	-1.53407	1.96209	-0.27668
C	-0.51292	2.25936	0.56385
C	0.58504	0.09893	0.24869
H	-0.41493	1.77523	1.53689
H	0.11345	3.13969	0.39405
C	2.01593	-0.06326	0.07816
C	2.57553	-1.35117	-0.02773
C	2.85948	1.05781	0.05078
C	3.95058	-1.50525	-0.15644
H	1.91057	-2.21724	-0.00447
C	4.23481	0.89670	-0.09300

H	2.42254	2.05486	0.13684
C	4.78280	-0.38278	-0.19258
H	4.38013	-2.50701	-0.23383
H	4.88438	1.77442	-0.12197
H	5.86317	-0.50774	-0.29690
H	-1.74128	2.61776	-1.13056

**nr-1aa-TS**			
E opt = -862.94997612			
i-freq = -312.4			
E/au = -866.00606826			
ZPE/au = -865.703503			
H/au = -865.686546			
DG/au = -865.747023			
38			
C	2.37146	-0.72459	0.75257
C	3.75289	-0.71041	1.01856
C	4.68756	-0.80130	-0.00434
H	5.75407	-0.75361	0.22726
C	1.95475	-0.95347	-0.58884
C	2.90762	-1.05761	-1.61215
H	4.08348	-0.58146	2.05257
C	4.26259	-0.95249	-1.33064
H	2.55401	-1.23905	-2.62939
H	4.99399	-1.01707	-2.13924
N	0.62983	-1.23076	-0.90443
C	1.37953	-0.59630	1.80451
C	0.21906	0.12772	1.69853
C	-0.40408	-0.97891	-0.28222
H	-0.53163	-0.05121	2.47692
C	-1.81139	-1.16942	-0.30451
C	-2.59770	-0.45690	-1.23783
C	-2.45211	-1.96581	0.67052
C	-3.98284	-0.56181	-1.20214
H	-2.10642	0.17515	-1.97993
C	-3.83696	-2.06076	0.68927
H	-1.84904	-2.50183	1.40634
C	-4.61064	-1.36060	-0.24279
H	-4.58157	-0.01058	-1.93134
H	-4.32251	-2.68732	1.44129
H	-5.69999	-1.43483	-0.21776
H	1.51182	-1.22255	2.69387
C	-0.04651	1.33851	0.89909
C	-1.35531	1.85547	0.88057
C	0.95111	2.04133	0.19916
C	-1.66695	3.00215	0.15734
H	-2.13948	1.33441	1.43607
C	0.63704	3.18832	-0.52401
H	1.98388	1.69229	0.23054
C	-0.67256	3.67127	-0.55753
H	-2.69344	3.37684	0.15283
H	1.42816	3.71796	-1.06073
H	-0.91345	4.57195	-1.12709

Bibliography

(1) Budavari, S.; O'Neil, M. J.; Smith, A.; Heckelman, P. E.; Kinneary, J. F.; *The Merck Index: 12th edition*; MERCK & CO., Inc., 1996.

(2) Kametani, T.; Takeda, H.; Suzuki, Y.; Kasai, H.; Honda, T. Application of the Lewis Acid Catalyzed [4+2]Cycloaddition Reaction to Synthesis of Natural Quinoline Alkaloids. *Heterocycles* **1986**, *24*, 3385–3395. https://doi.org/10.3987/R-1986-12-3385.

(3) Pelagalli, A.; Pellacani, L.; Scandozza, E.; Fioravanti, S. Aza-Henry Reactions on C-Alkyl Substituted Aldimines. *Molecules* **2016**, *21*, 723. https://doi.org/10.3390/molecules21060723.

(4) Qiang, L. G.; Baine, N. H. A Convenient Synthesis of Substituted Quinolines by Thermal Electrocyclic Rearrangement of O-Vinyl Anils under Nonacidic Conditions. *J. Org. Chem.* **1988**, *53*, 4218–4222. https://doi.org/10.1021/jo00253a011.

(5) Kwon, S. H.; Seo, H.-A.; Cheon, C.-H. Total Synthesis of Luotonin A and Rutaecarpine from an Aldimine via the Designed Cyclization. *Org. Lett.* **2016**, *18*, 5280–5283. https://doi.org/DOI: 10.1021/acs.orglett.6b02597.

(6) Imhof, W. C,H Bond Activation of Imino Substituted Heterocycles: Synthesis and Crystal Structure of [M2-H3-(R) N-CH2-C=C-C(H)=C(H)-X]Fe2(CO)6 and the Isomeric Clusters [M2-H3-(R)N-CH2-C=C-X-C(R')=C(R")]Fe2(CO)6. *J. Organomet. Chem.* **1997**, *533*, 31–43. https://doi.org/10.1016/S0022-328X(96)06824-6.

(7) Yoshida, Y.; Saito, J.; Mitani, M.; Takagi, Y.; Matsui, S.; Ishii, S.; Nakano, T.; Kashiwa, N.; Fujita, T. Living Ethylene/Norbornene Copolymerisation Catalyzed by Titanium Complexes Having Two Pyrrolide-Imine Chelate Ligands. *Chem. Commun.* **2002**, *2*, 1298–1299. https://doi.org/10.1039/b202391a.

(8) Shrestha, B.; Basnet, P.; Dhungana, R. K.; KC, S.; Thapa, S.; Sears, J. M.; Giri, R. Ni-Catalyzed Regioselective 1,2-Dicarbofunctionalization of Olefins by Intercepting Heck Intermediates as Imine-Stabilized Transient Metallacycles. *J. Am. Chem. Soc.* **2017**, *139*, 10653–10656. https://doi.org/10.1021/jacs.7b06340.

(9) Mäkelä, M. K.; Bulatov, E.; Malinen, K.; Talvitie, J.; Nieger, M.; Melchionna, M.; Lenarda, A.; Hu, T.; Wirtanen, T.; Helaja, J. Carbocatalytic Cascade Synthesis of Polysubstituted Quinolines from Aldehydes and 2-Vinyl Anilines. *Adv. Synth. Catal.* **2021**, *363*, 3775–3782. https://doi.org/10.1002/adsc.202100711.

(10) Ghorai, J.; Reddy, A. C. S.; Anbarasan, P. Divergent Functionalization of *N*-Alkyl-2-Alkenylanilines: Efficient Synthesis of Substituted Indoles and Quinolines. *Chem. - An Asian J.* **2018**, *13*, 2499–2504. https://doi.org/10.1002/asia.201800441.

(11) San Jang, S.; Kim, Y. H.; Youn, S. W. Divergent Syntheses of Indoles and Quinolines Involving N1–C2–C3 Bond Formation through Two Distinct Pd Catalyses. *Org. Lett.* **2020**, *22*, 9151–9157. https://doi.org/10.1021/acs.orglett.0c02898.

(12) Xiao, F.; Chen, W.; Liao, Y.; Deng, G.-J. Cu(II)-Promoted Three-Component Coupling Sequence for the Efficient Synthesis of Substituted Quinolines. *Org. Biomol. Chem.* **2012**, *10*, 8593–8596. https://doi.org/10.1039/c2ob26484f.

(13) Zhong, M.; Sun, S.; Cheng, J.; Shao, Y. Iron-Catalyzed Cyclization of Nitrones with Geminal-

Substituted Vinyl Acetates: A Direct [4 + 2] Assembly Strategy Leading to 2,4-Disubstituted Quinolines. *J. Org. Chem.* **2016**, *81*, 10825–10831. https://doi.org/10.1021/acs.joc.6b01910.

(14) Yuan, J.-W.; Liu, S.-N.; Qu, L.-B. $Cu(OAc)_2$-Catalyzed Direct Radical C2 Arylation of Quinoline *N*-Oxide with Arylamines. *Tetrahedron* **2017**, *73*, 2267–2275. https://doi.org/10.1016/j.tet.2017.03.009.

(15) Kumar, P.; Garg, V.; Kumar, M.; Verma, A. K. Rh(III)-Catalyzed Alkynylation: Synthesis of Functionalized Quinolines from Aminohydrazones. *Chem. Commun.* **2019**, *55*, 12168–12171. https://doi.org/10.1039/c9cc06205j.

(16) Genovino, J.; Lian, Y.; Zhang, Y.; Hope, T. O.; Juneau, A.; Gagné, Y.; Ingle, G.; Frenette, M. Metal-Free-Visible Light C-H Alkylation of Heteroaromatics via Hypervalent Iodine-Promoted Decarboxylation. *Org. Lett.* **2018**, *20*, 3229–3232. https://doi.org/10.1021/acs.orglett.8b01085.

(17) Lan, X.-B.; Ye, Z.; Huang, M.; Liu, J.; Liu, Y.; Ke, Z. Nonbifunctional Outer-Sphere Strategy Achieved Highly Active α-Alkylation of Ketones with Alcohols by *N*-Heterocyclic Carbene Manganese (NHC-Mn). *Org. Lett.* **2019**, *21*, 8065–8070. https://doi.org/10.1021/acs.orglett.9b03030.

(18) Zheng, W.; Yang, W.; Luo, D.; Min, L.; Wang, X.; Hu, Y. A Tf_2O-Promoted Synthesis of Functionalized Quinolines from Ketoximes and Alkynes. *Adv. Synth. Catal.* **2019**, *361*, 1995–1999. https://doi.org/10.1002/adsc.201801724.

(19) Xiao, S.-T.; Ma, C.-T.; Di, J.-Q.; Zhang, Z.-H. MOF-5 as a Highly Efficient and Recyclable Catalyst for One Pot Synthesis of 2,4-Disubstituted Quinoline Derivatives. *New J. Chem.* **2020**, *44*, 8614–8620. https://doi.org/10.1039/d0nj01301c.

(20) Anson, C. W.; Stahl, S. S. *J. Am. Chem. Soc.* **2017**, *139*, 18472–18475. https://doi.org/10.1021/jacs.7b11362.

(21) Theodorou, A.; Triandafillidi, I.; Kokotos, C. G. Organocatalytic Synthesis of Oxazolines and Dihydrooxazines from Allyl-Amides: Bypassing the Inherent Regioselectivity of the Cyclization. *Adv. Synth. Catal.* **2018**, *360*, 951–957. https://doi.org/10.1002/ADSC.201701386.

(22) Frisch, M. J.; Trucks, G. W.; Schlegel, H. B.; Scuseria, G. E.; Robb, M. A.; Cheeseman, J. R.; Scalmani, G.; Barone, V.; Petersson, G. A.; Nakatsuji, H.; Li, X.; Caricato, M.; Marenich, A. V.; Bloino, J.; Janesko, B. G.; Gomperts, R.; Mennucci, B.; Hratch, D. J. Gaussian 16. Gaussian, Inc., Wallingford CT 2016.

(23) Adamo, C.; Barone, V. Toward Reliable Density Functional Methods without Adjustable Parameters: The PBE0 Model. *J. Chem. Phys.* **1999**, *110*, 6158. https://doi.org/10.1063/1.478522.

(24) Grimme, S.; Ehrlich, S.; Goerigk, L. Effect of the Damping Function in Dispersion Corrected Density Functional Theory. *J. Comput. Chem.* **2011**, *32*, 1456–1465. https://doi.org/10.1002/JCC.21759.

(25) Zhao, Y.; Truhlar, D. G. Design of Density Functionals That Are Broadly Accurate for Thermochemistry, Thermochemical Kinetics, and Nonbonded Interactions. *J. Phys. Chem. A* **2005**, *109*, 5656–5667. https://doi.org/10.1021/jp050536c.

(26) Weigend, F.; Ahlrichs, R. Balanced Basis Sets of Split Valence, Triple Zeta Valence and Quadruple Zeta Valence Quality for H to Rn: Design and Assessment of Accuracy. *Phys. Chem. Chem. Phys.* **2005**, *7*, 3297–3305. https://doi.org/10.1039/B508541A.

(27) Barone, V.; Cossi, M.; Tomasi, J. Geometry Optimization of Molecular Structures in Solution by the Polarizable Continuum Model. *J. Comput. Chem.* **1998**, *19*, 404–417. https://doi.org/10.1002/(SICI)1096-987X(199803)19:4<404::AID-JCC3>3.0.CO;2-W.

(28) Legault, C. Y. CYLview, 1.0b; Université de Sherbrooke, 2009 (http://www.cylview.org).

(29) Roth, H. G.; Romero, N. A.; Nicewicz, D. A. Experimental and Calculated Electrochemical Potentials of Common Organic Molecules for Applications to Single-Electron Redox Chemistry. *Synlett* **2016**, *27*, 714–723. https://doi.org/10.1055/s-0035-1561297.

(30) Paenurk, E.; Kaupmees, K.; Himmel, D.; Kütt, A.; Kaljurand, I.; Koppel, I. A.; Krossing, I.; Leito, I. A Unified View to Brønsted Acidity Scales: Do We Need Solvated Protons? *Chem. Sci.* **2017**, *8*, 6964–6973. https://doi.org/10.1039/c7sc01424d.

Copies of NMR spectra

^{1}H NMR (400 MHz, $CDCl_3$) of **1d**

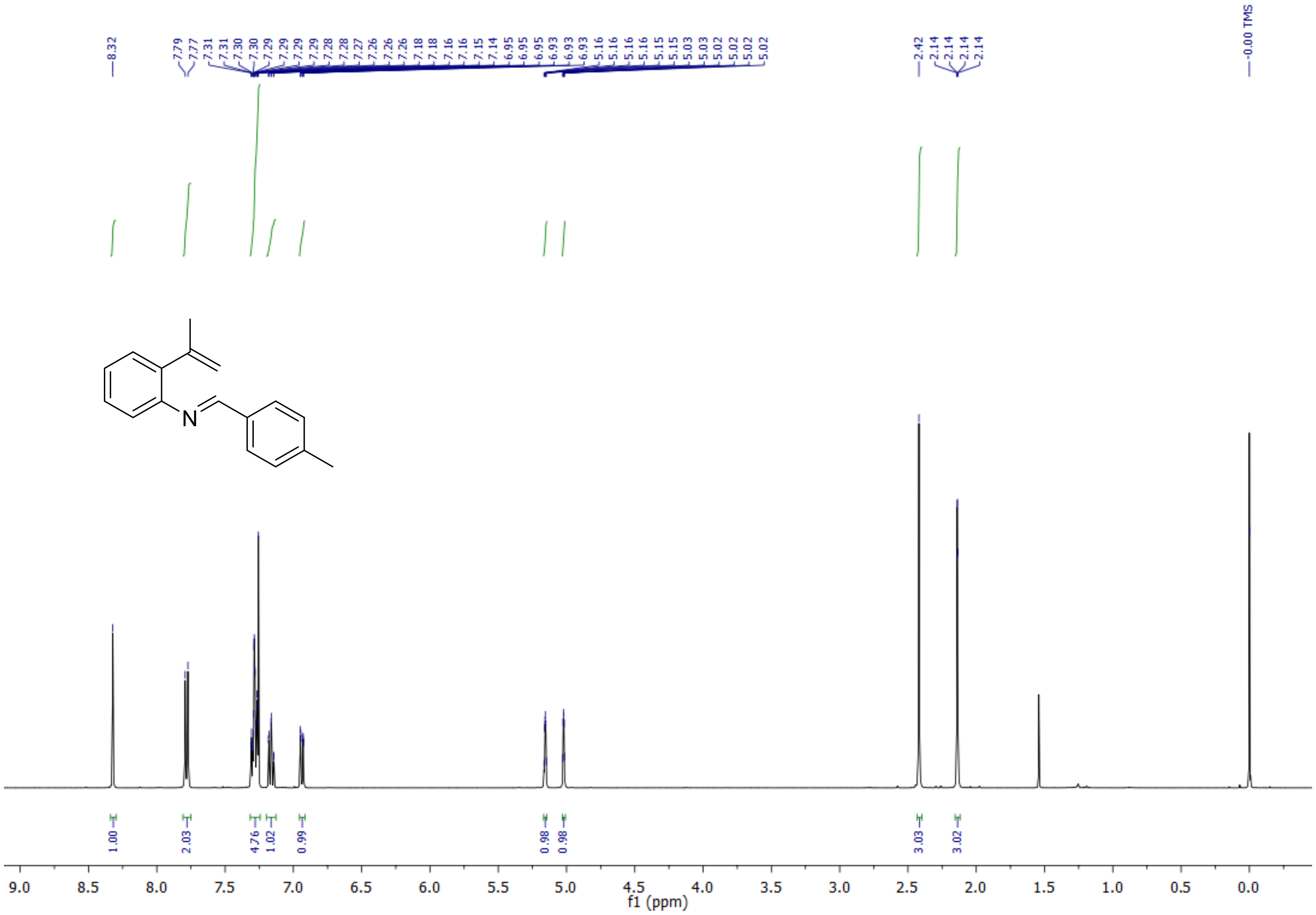

^{13}C NMR (101 MHz, $CDCl_3$) of **1d**

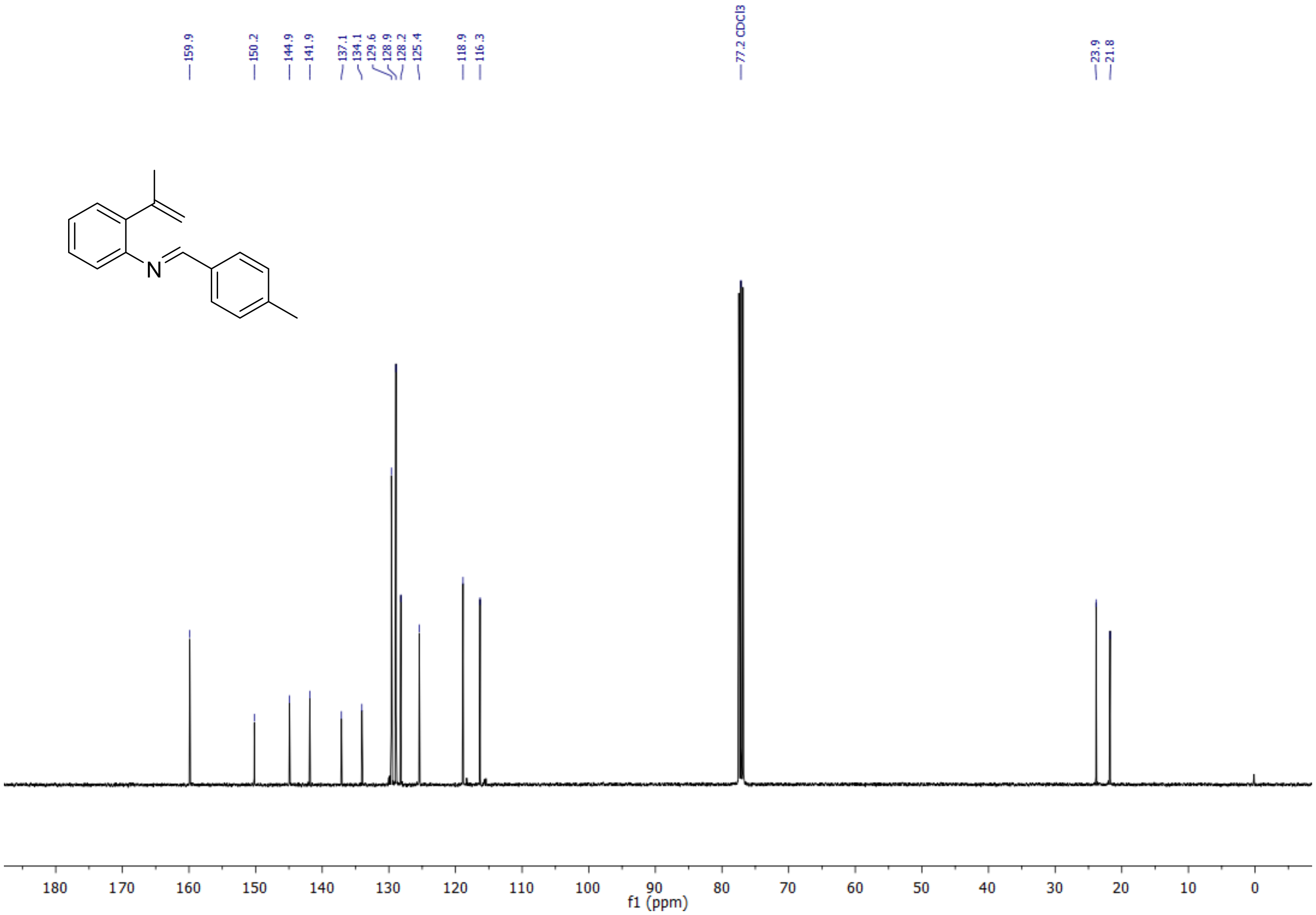

^{1}H–^{13}C HSQC-DEPT NMR (400 MHz, $CDCl_3$) of **1d**

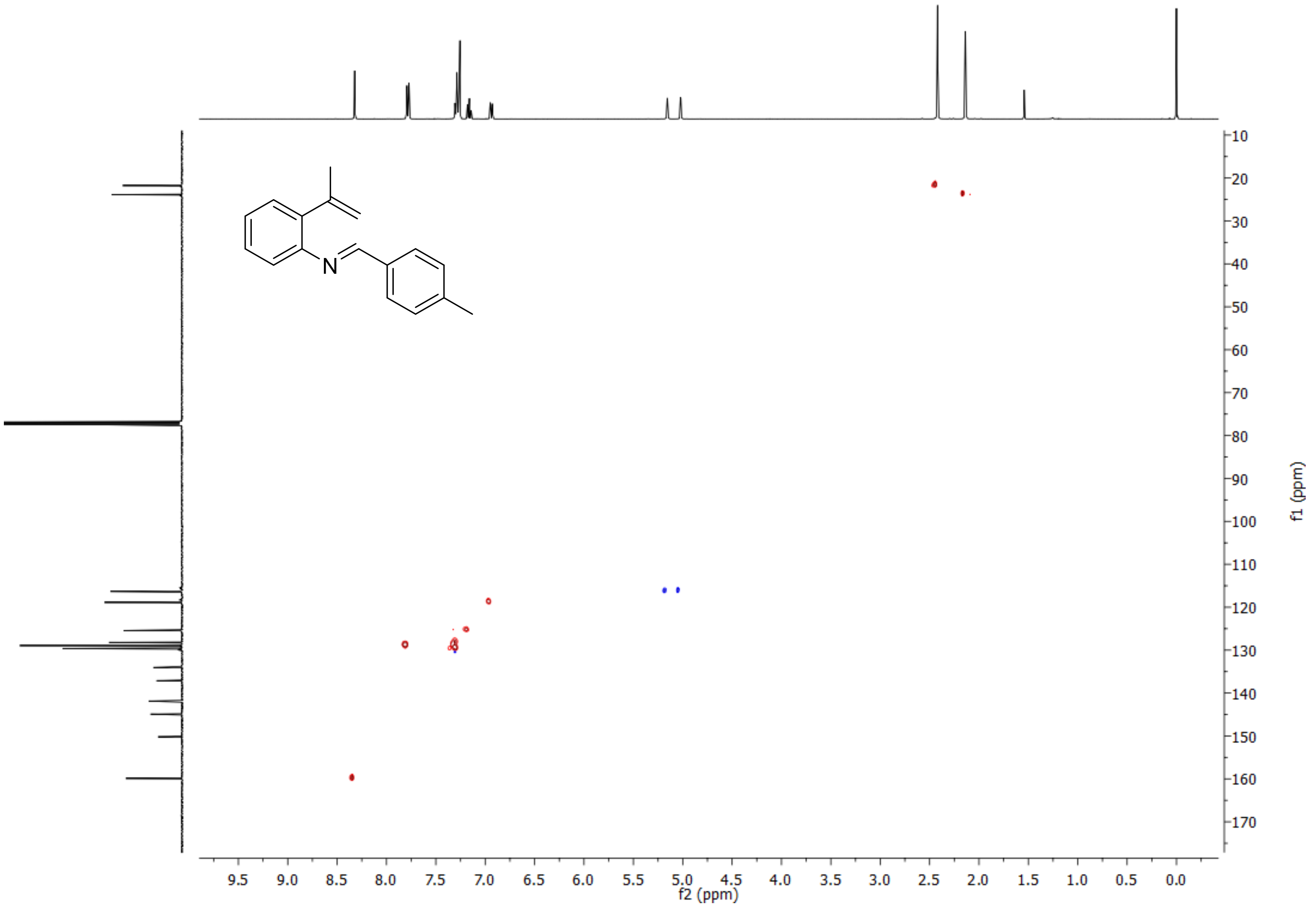

^{1}H NMR (400 MHz, $CDCl_3$) of **1e**

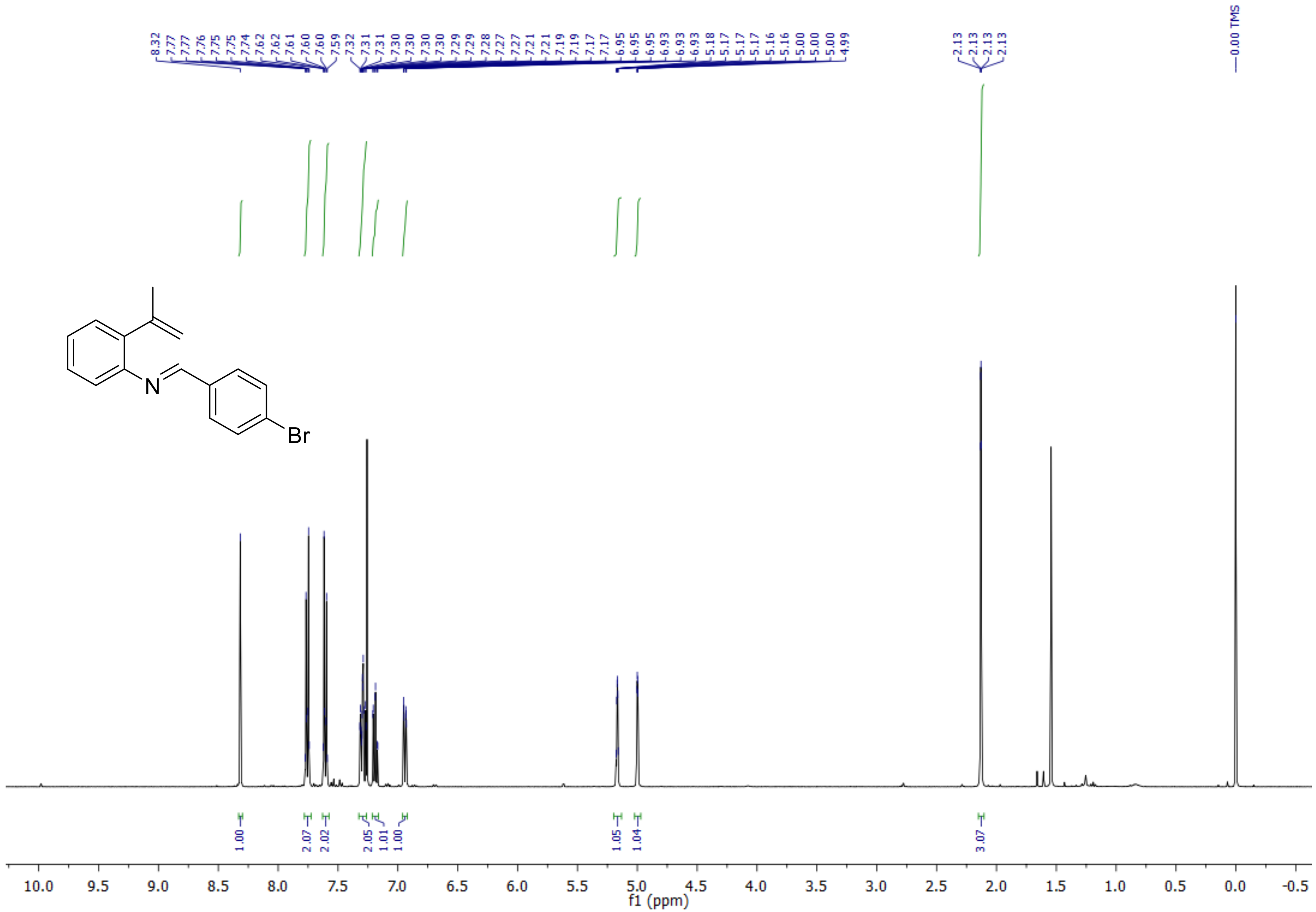

^{13}C NMR (101 MHz, CDCl$_3$) of **1e**

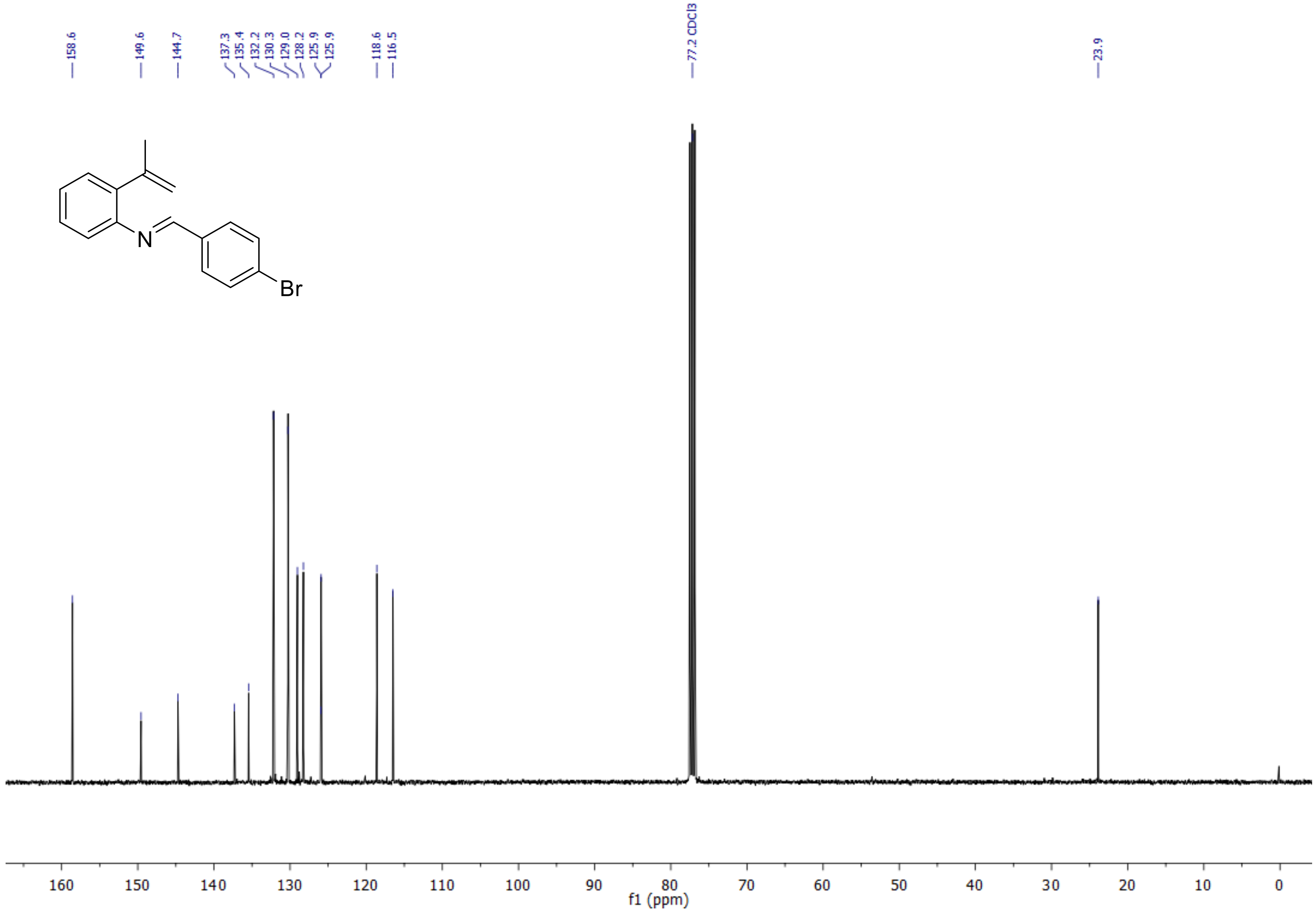

^{1}H–^{13}C HSQC-DEPT NMR (400 MHz, $CDCl_3$) of **1e**

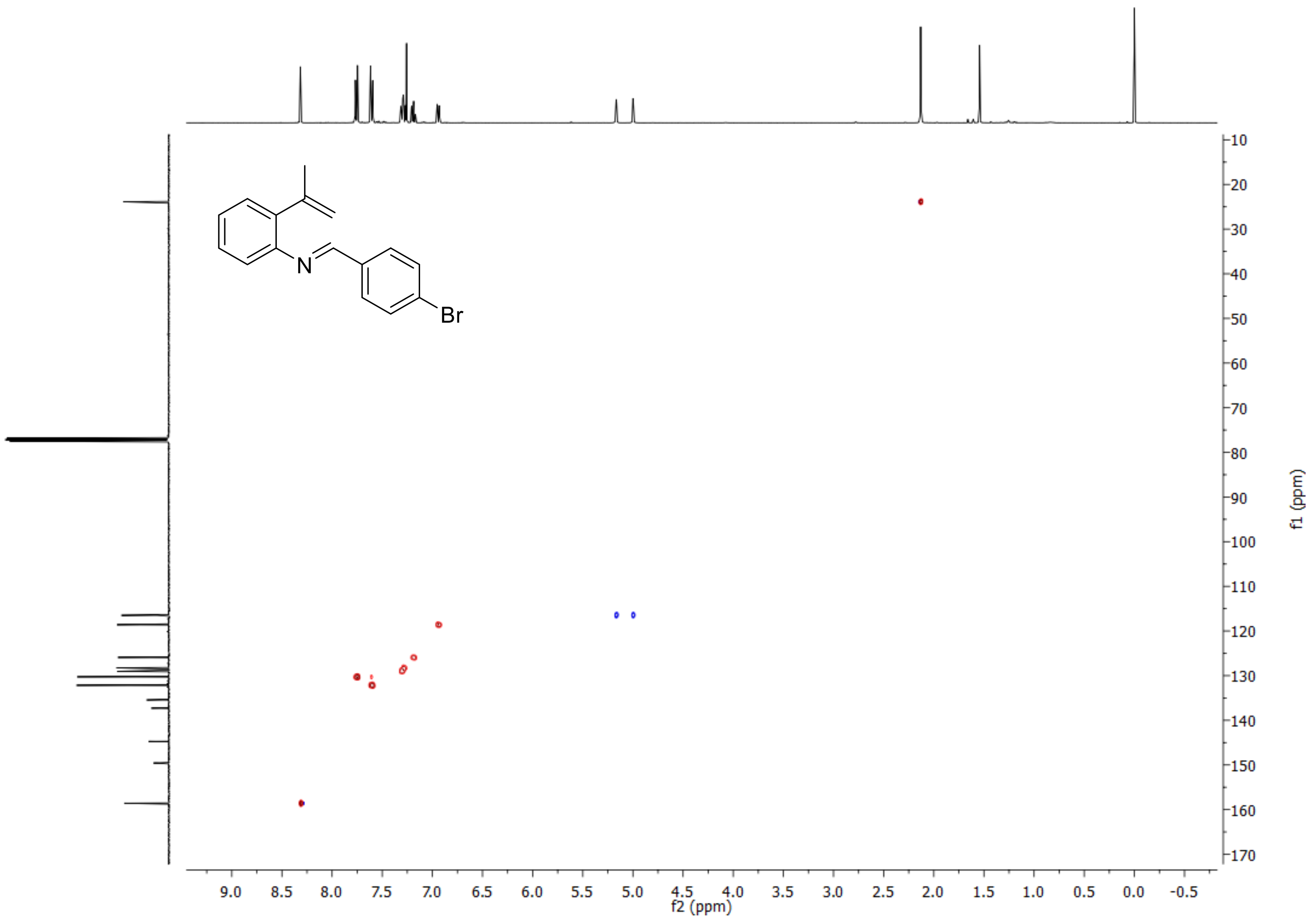

^{1}H NMR (400 MHz, $CDCl_3$) of **1f**

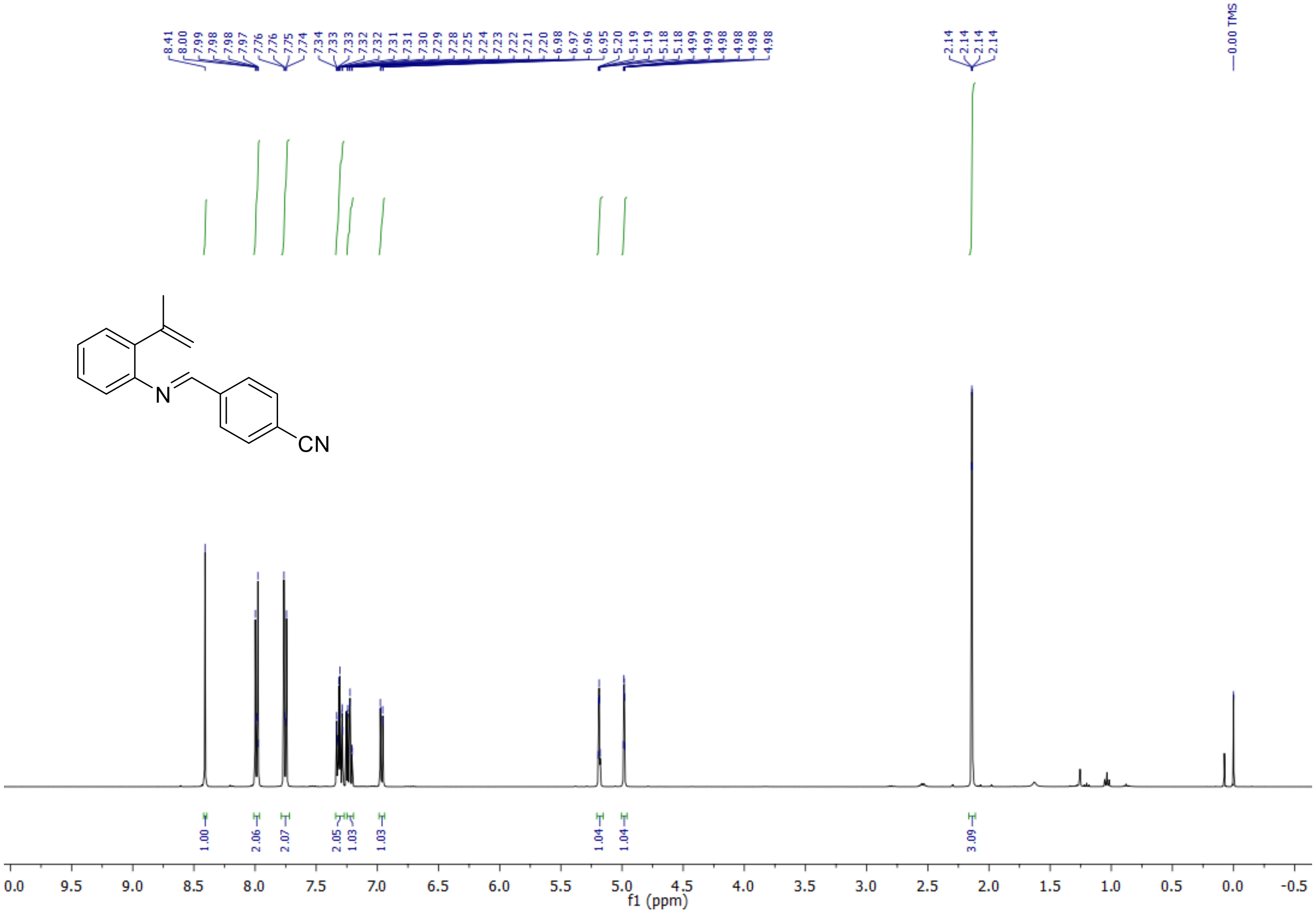

^{13}C NMR (101 MHz, CDCl$_3$) of **1f**

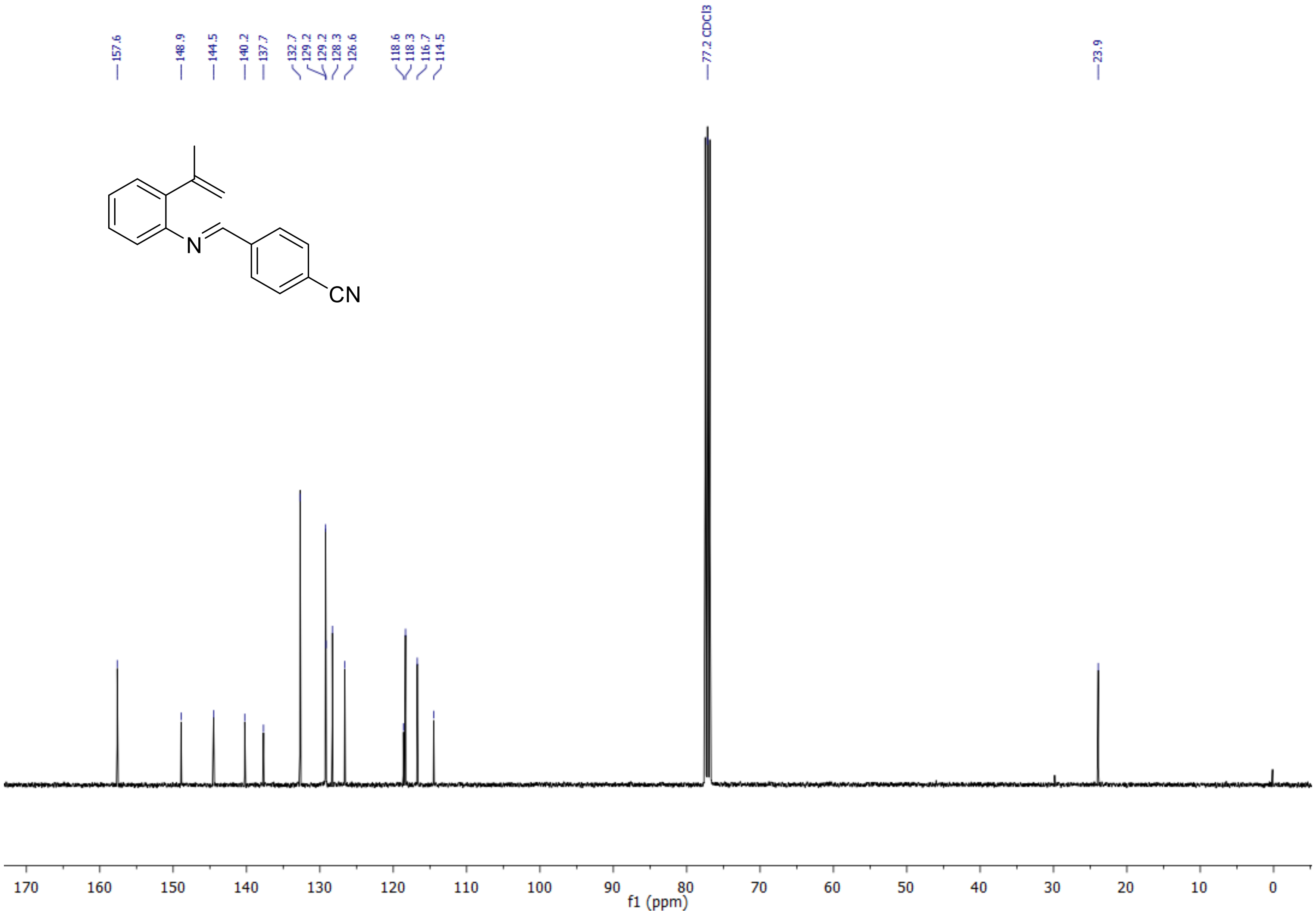

^{1}H–^{13}C HSQC-DEPT NMR (400 MHz, $CDCl_3$) of **1f**

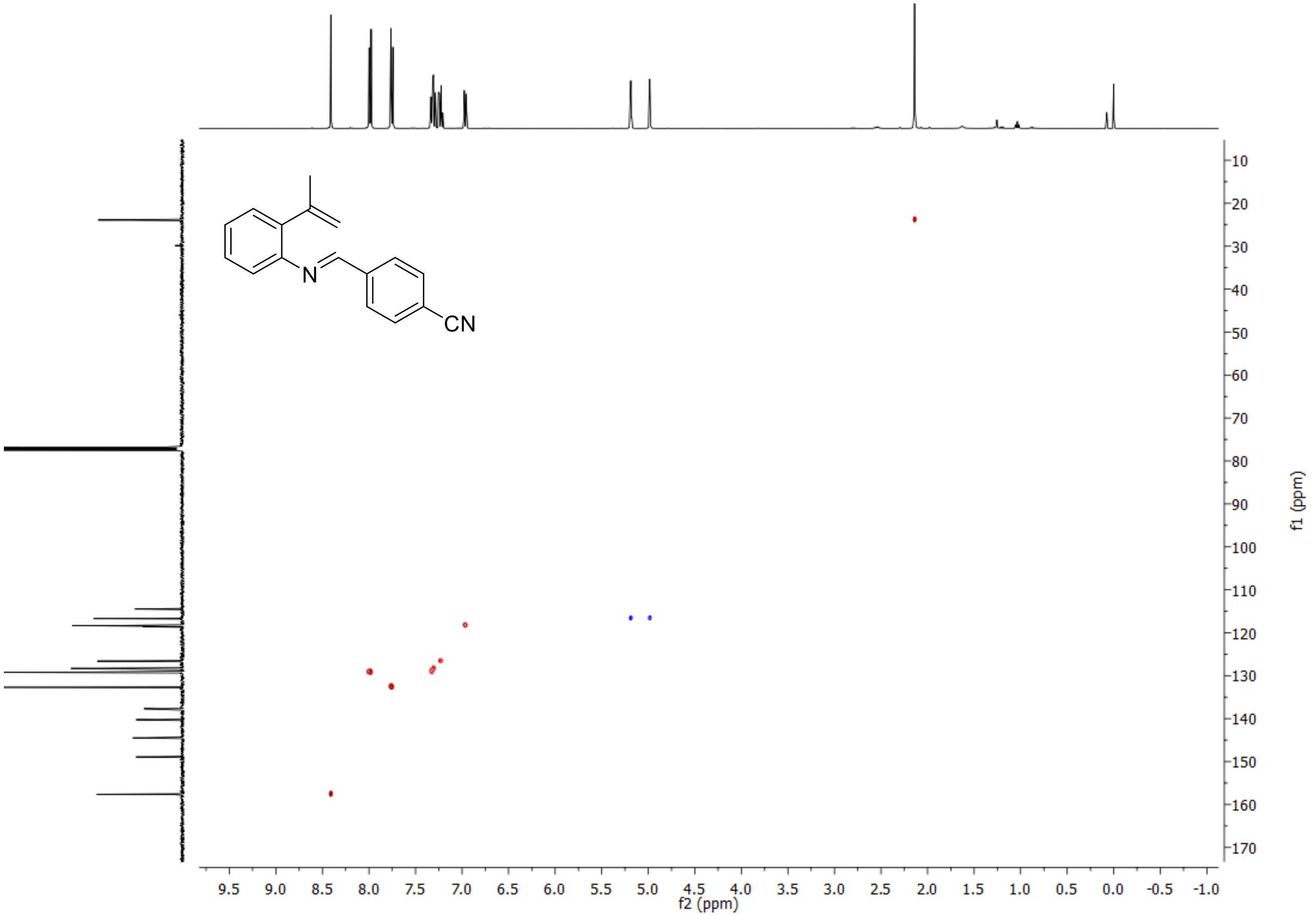

^{1}H NMR (400 MHz, $CDCl_3$) of **1g**

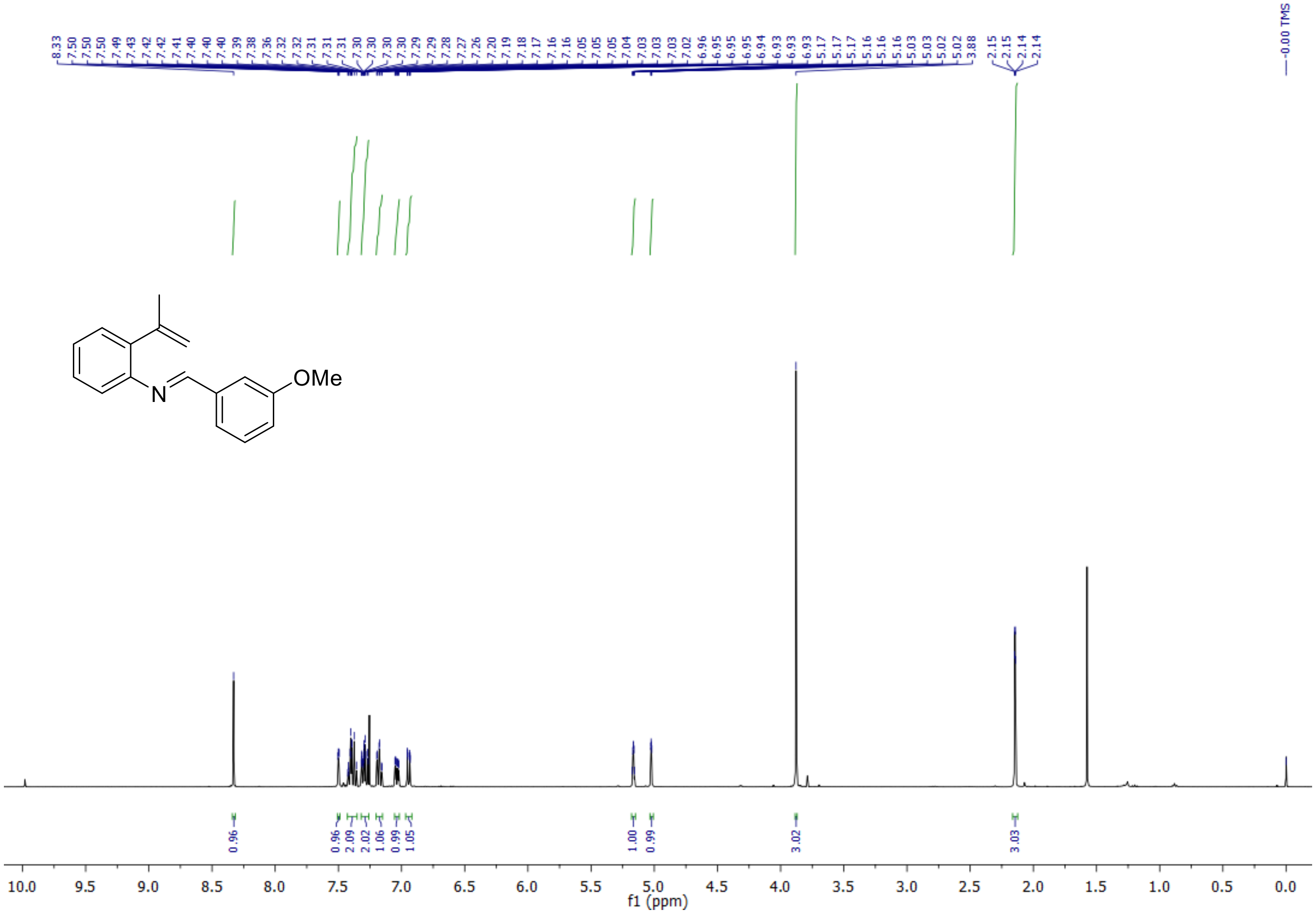

^{13}C NMR (101 MHz, $CDCl_3$) of **1g**

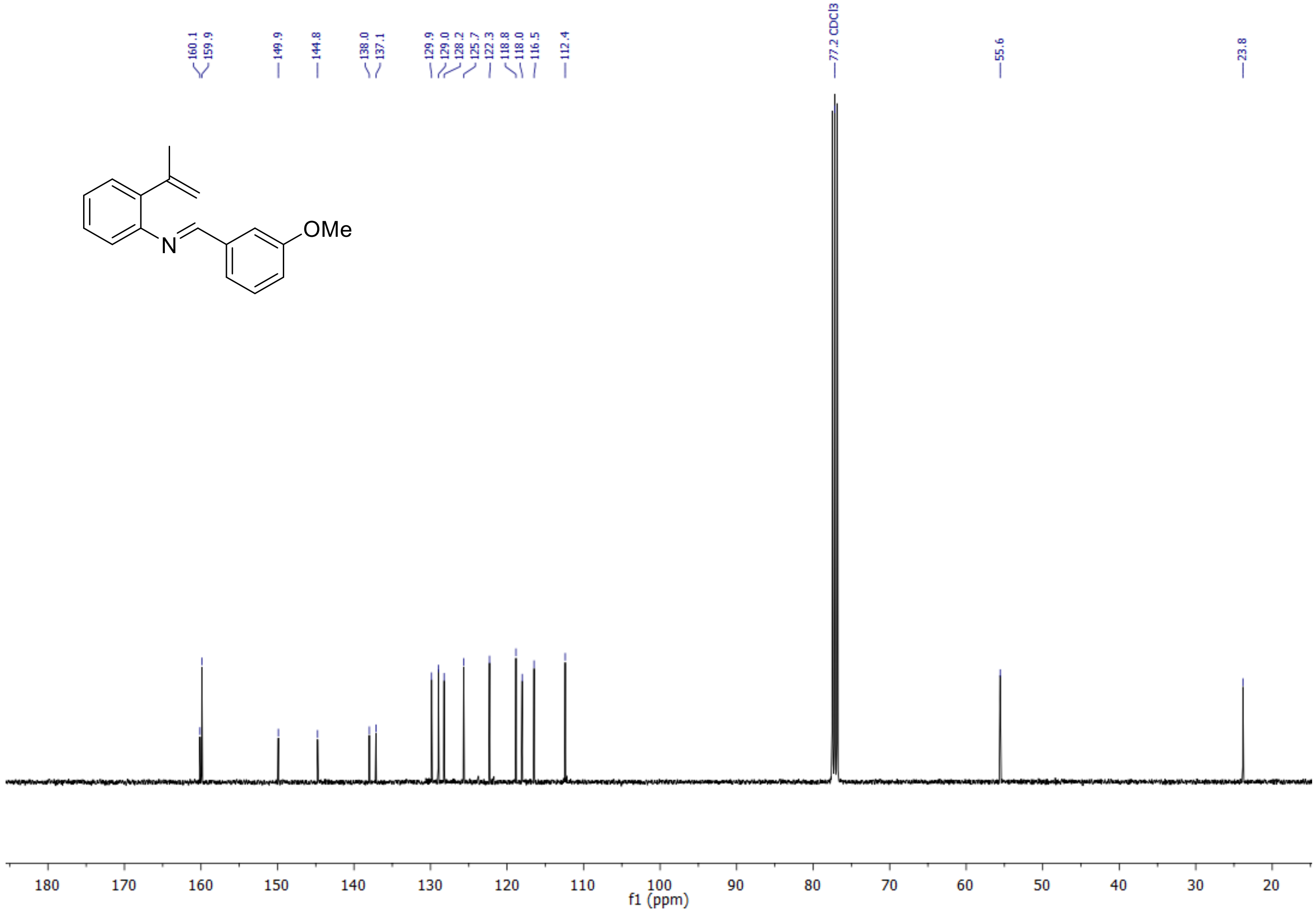

^{1}H–^{13}C HSQC-DEPT NMR (400 MHz, $CDCl_3$) of **1g**

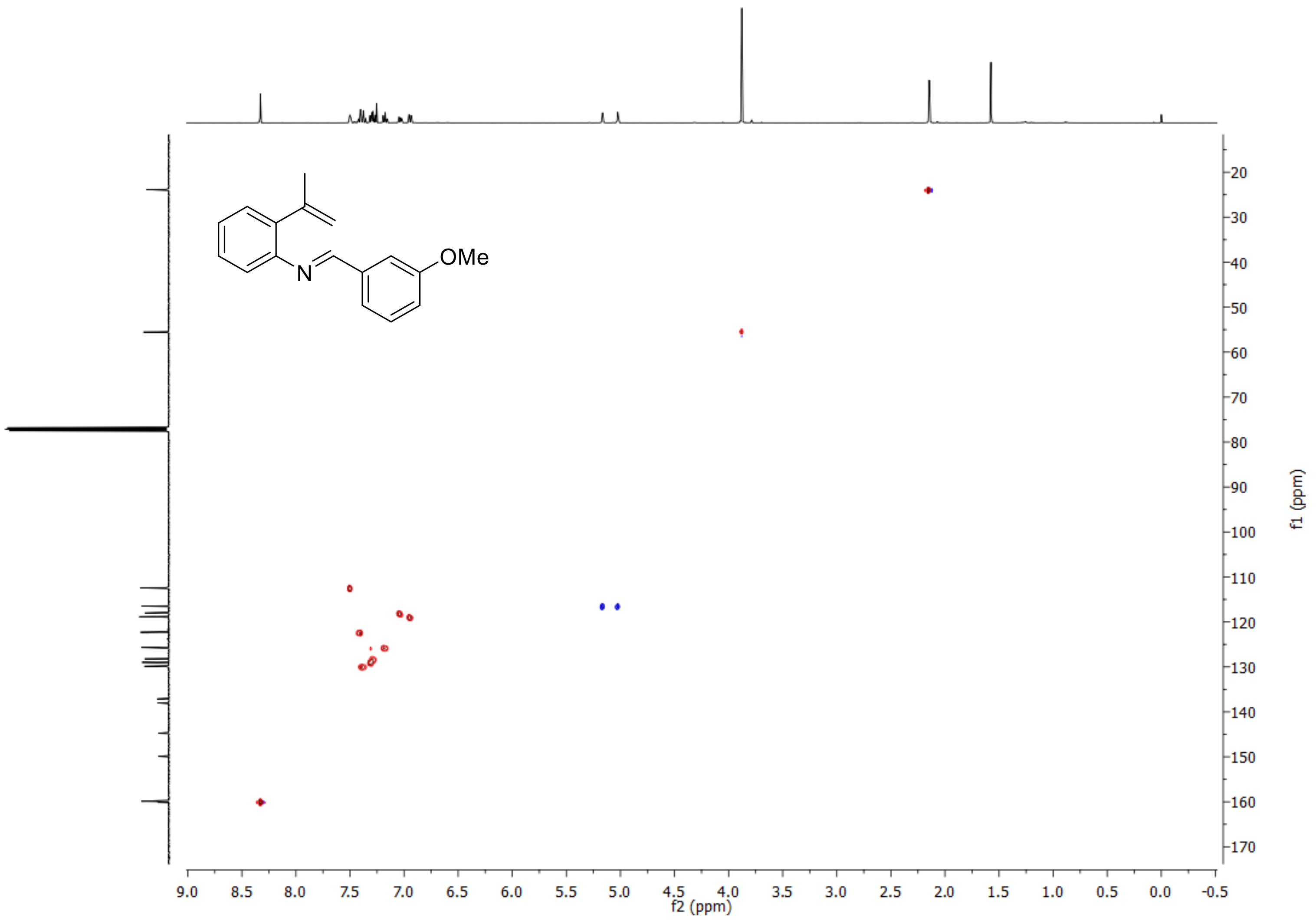

^{1}H NMR (400 MHz, $CDCl_3$) of **1h**

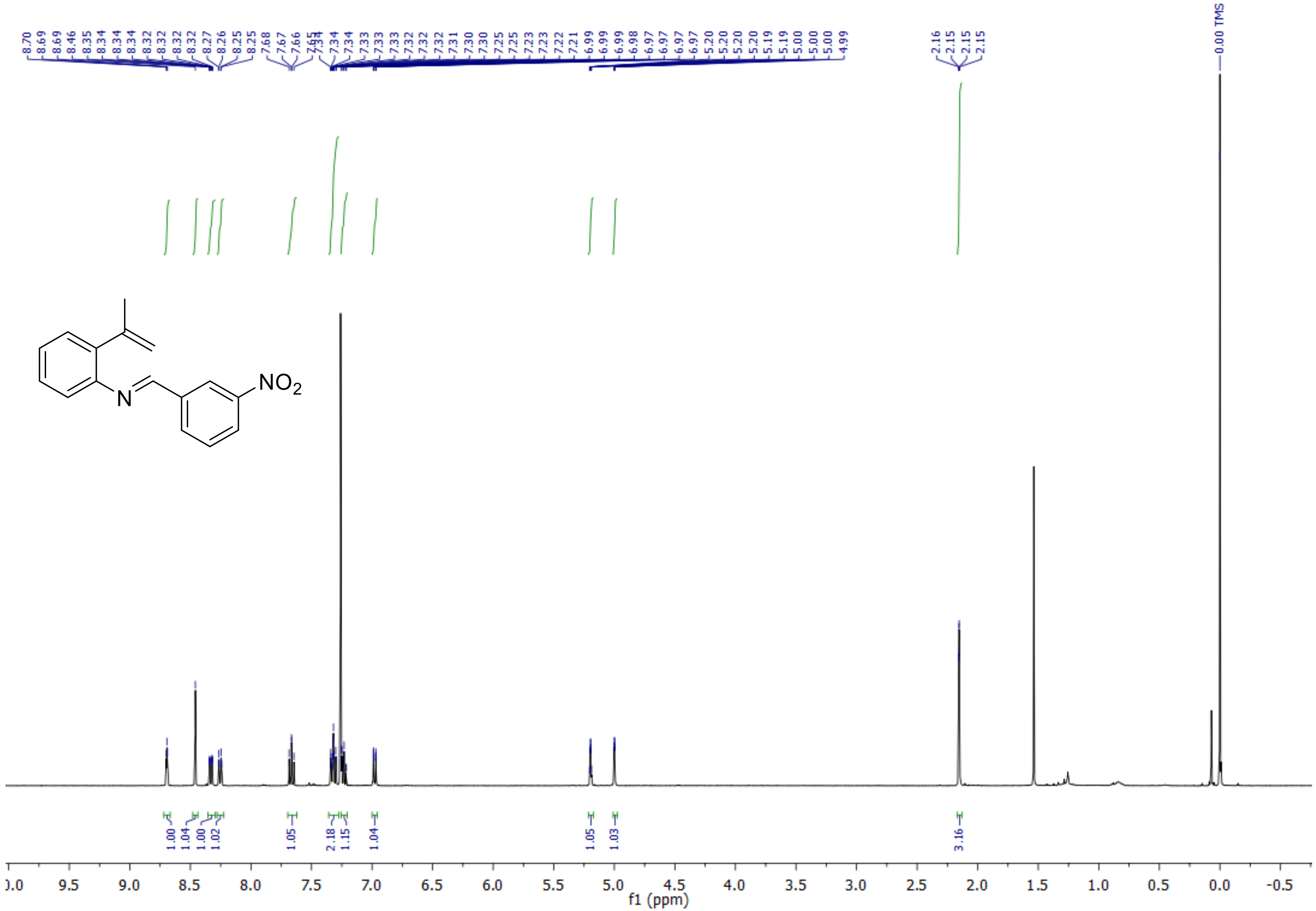

^{13}C NMR (101 MHz, $CDCl_3$) of **1h**

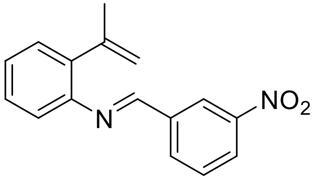

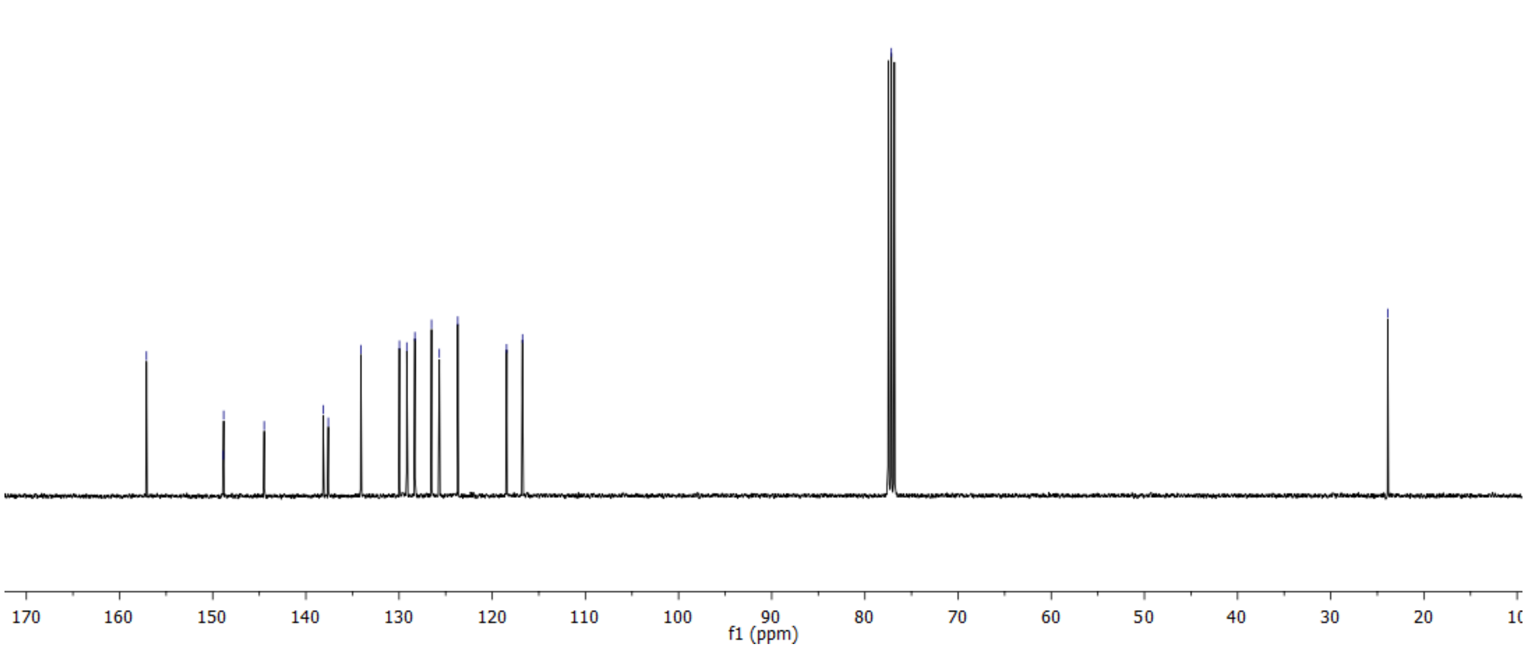

$^{1}H–^{13}C$ HSQC-DEPT NMR (400 MHz, $CDCl_3$) of **1h**

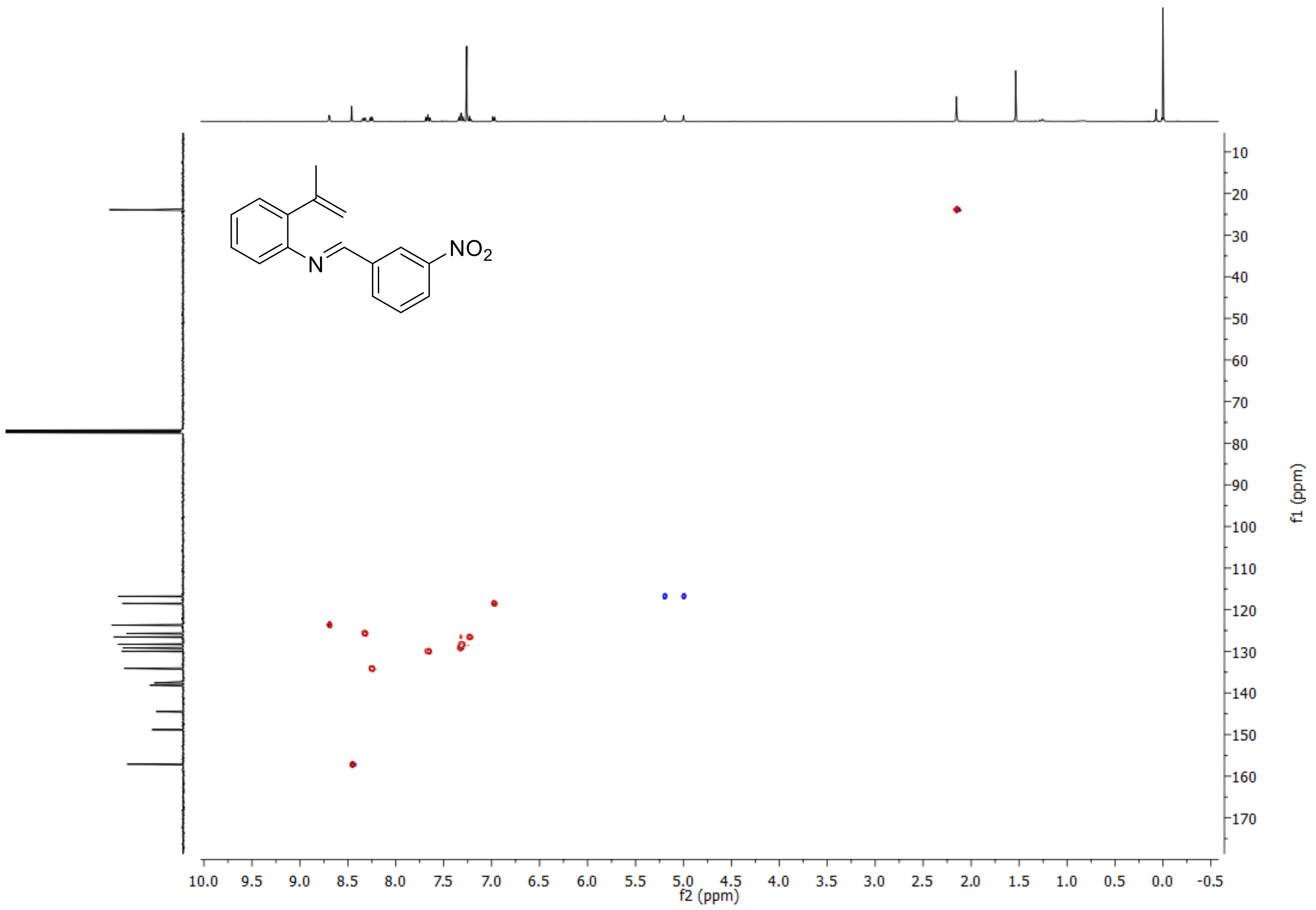

^{1}H NMR (400 MHz, $CDCl_3$) of **1j**

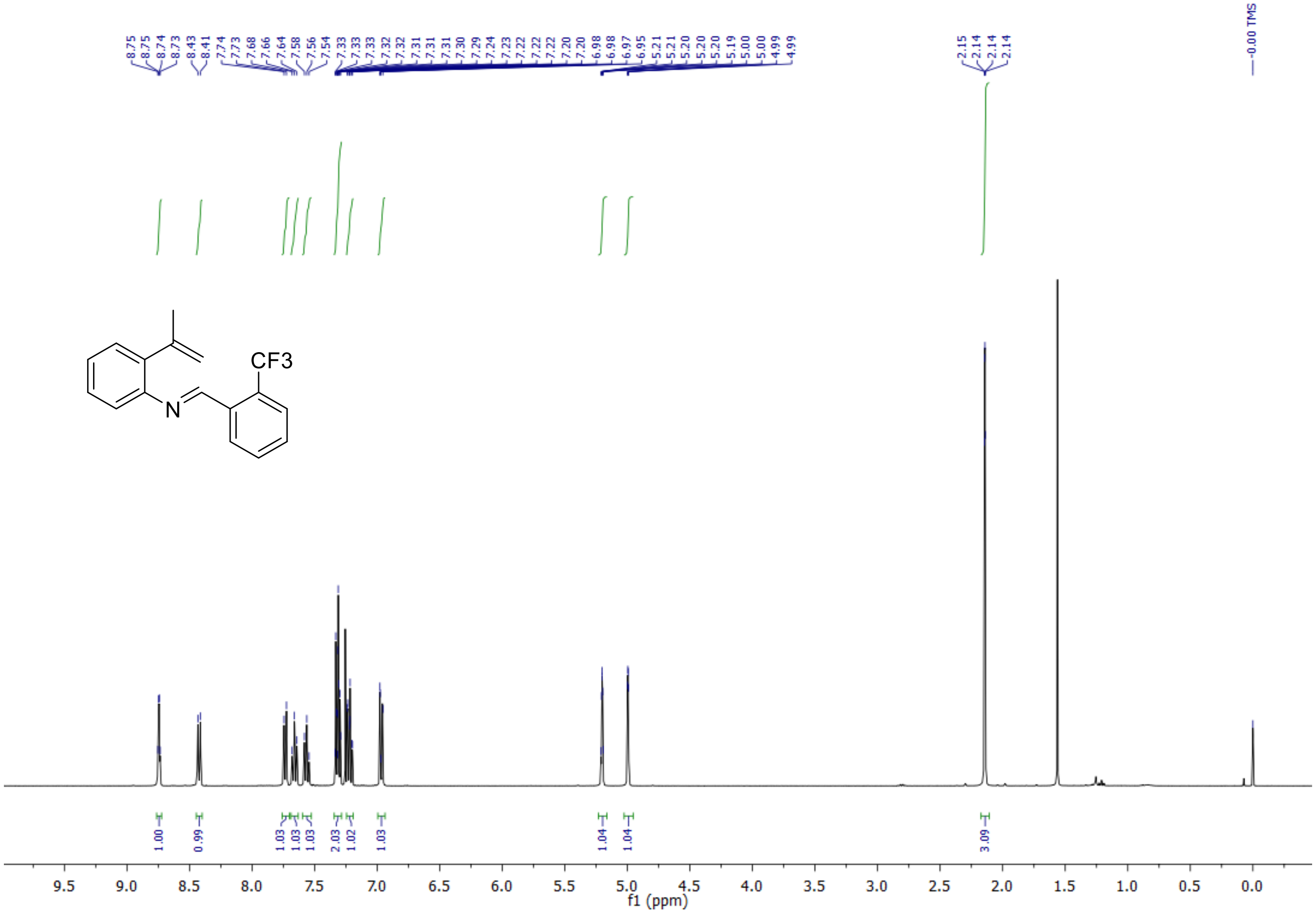

^{13}C NMR (101 MHz, $CDCl_3$) of **1j**

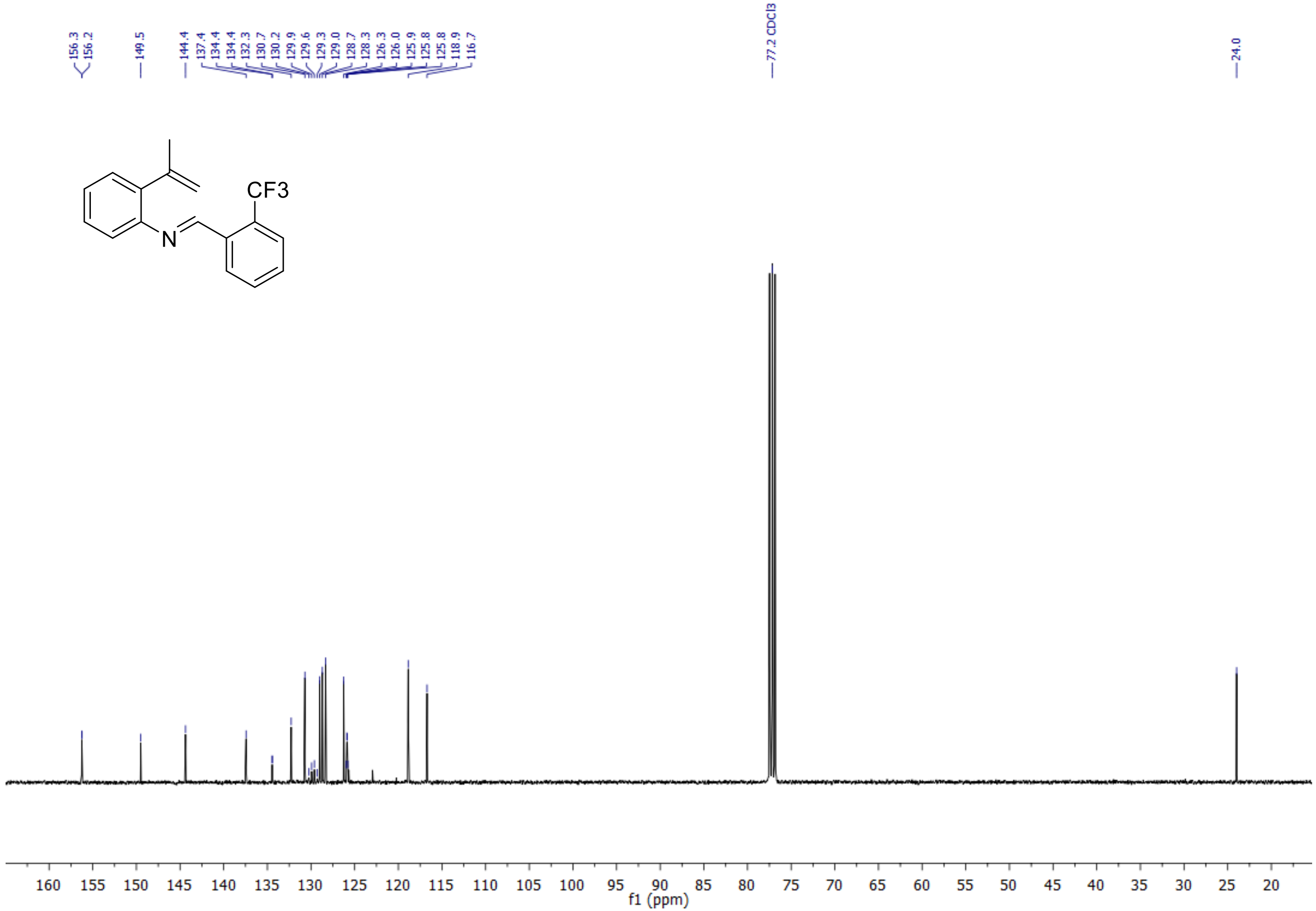

^{19}F NMR (377 MHz, $CDCl_3$) of **1j**

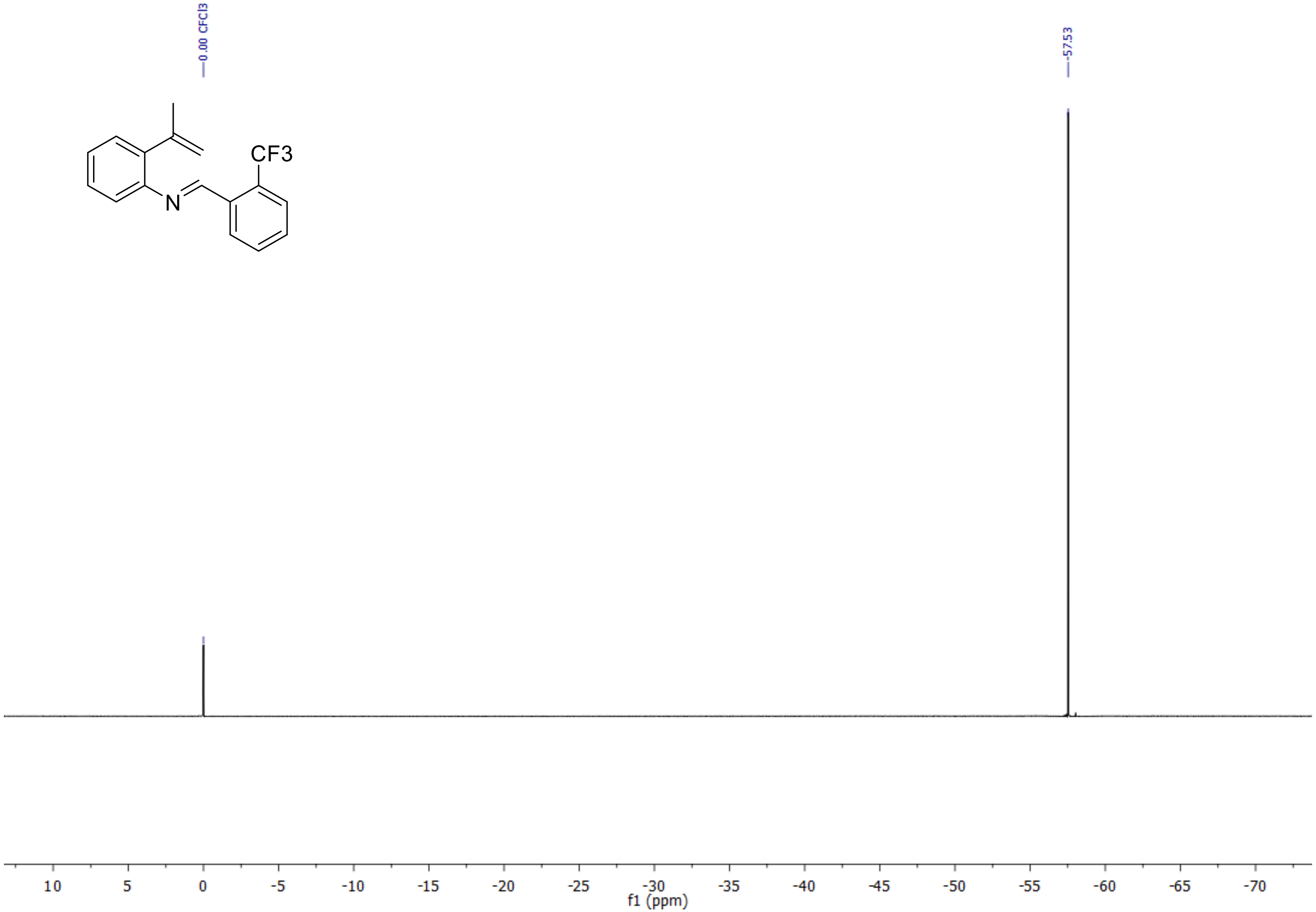

^{1}H–^{13}C HSQC-DEPT NMR (400 MHz, $CDCl_3$) of **1j**

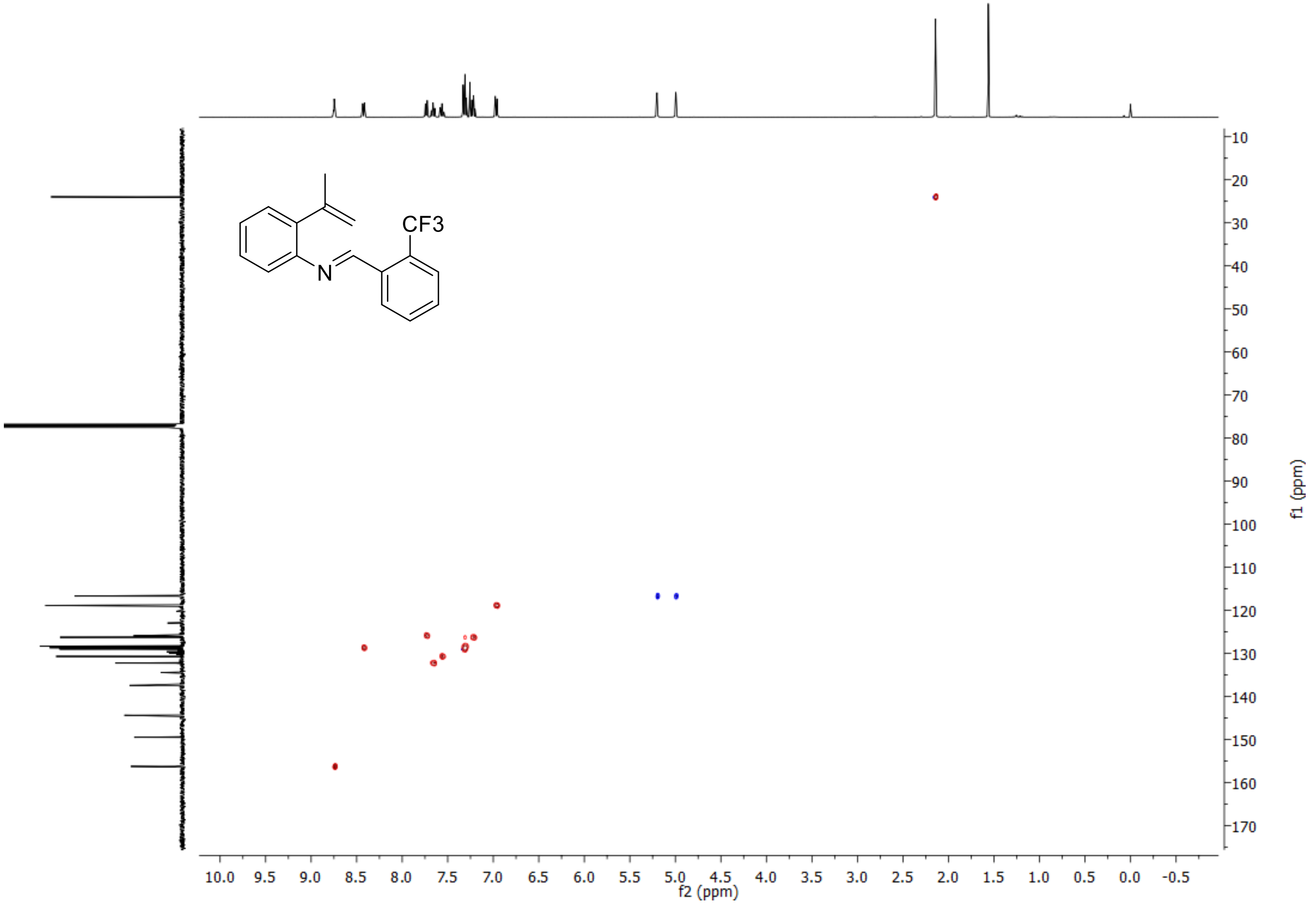

^{1}H NMR (400 MHz, $CDCl_3$) of **1k**

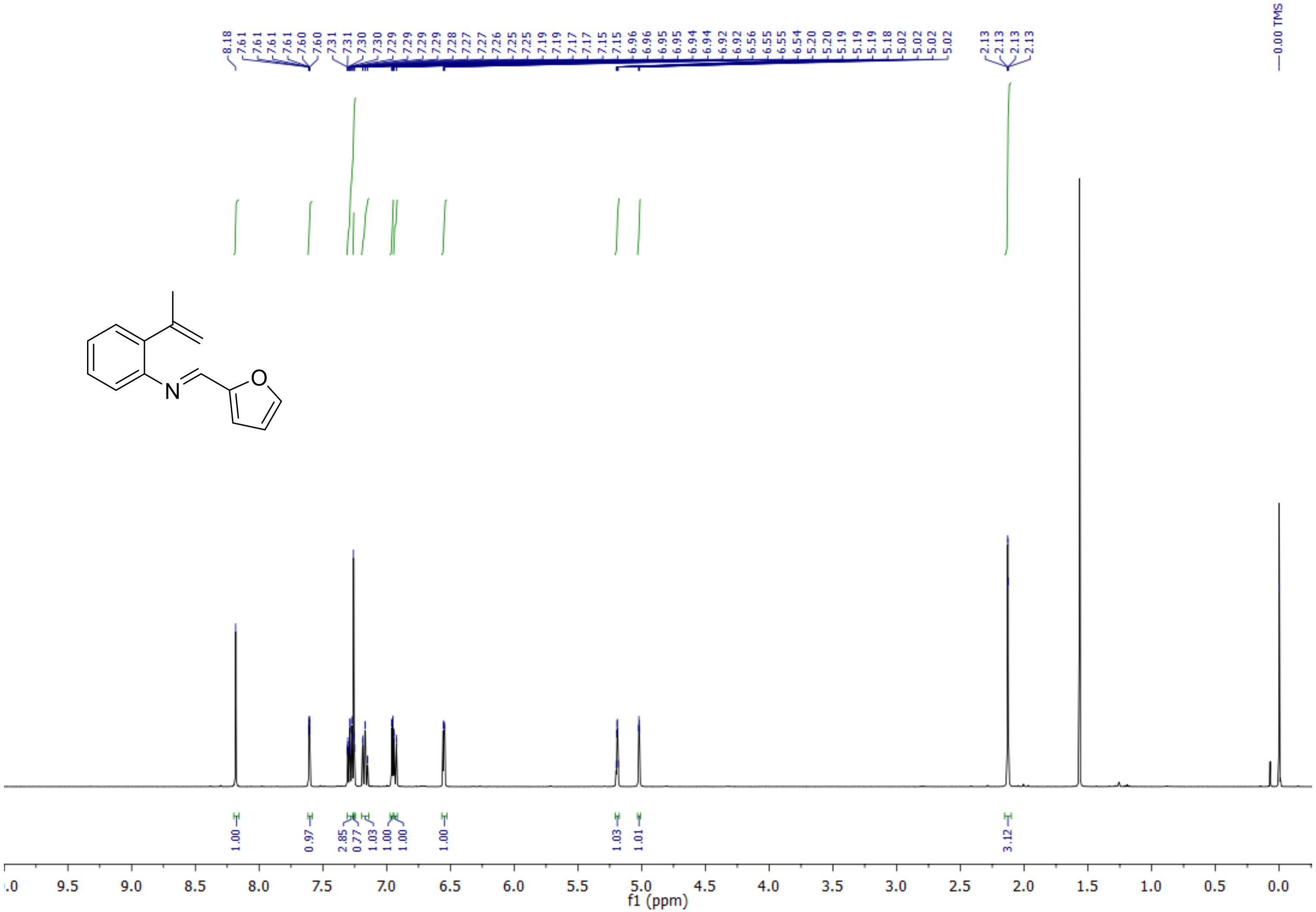

^{13}C NMR (101 MHz, $CDCl_3$) of **1k**

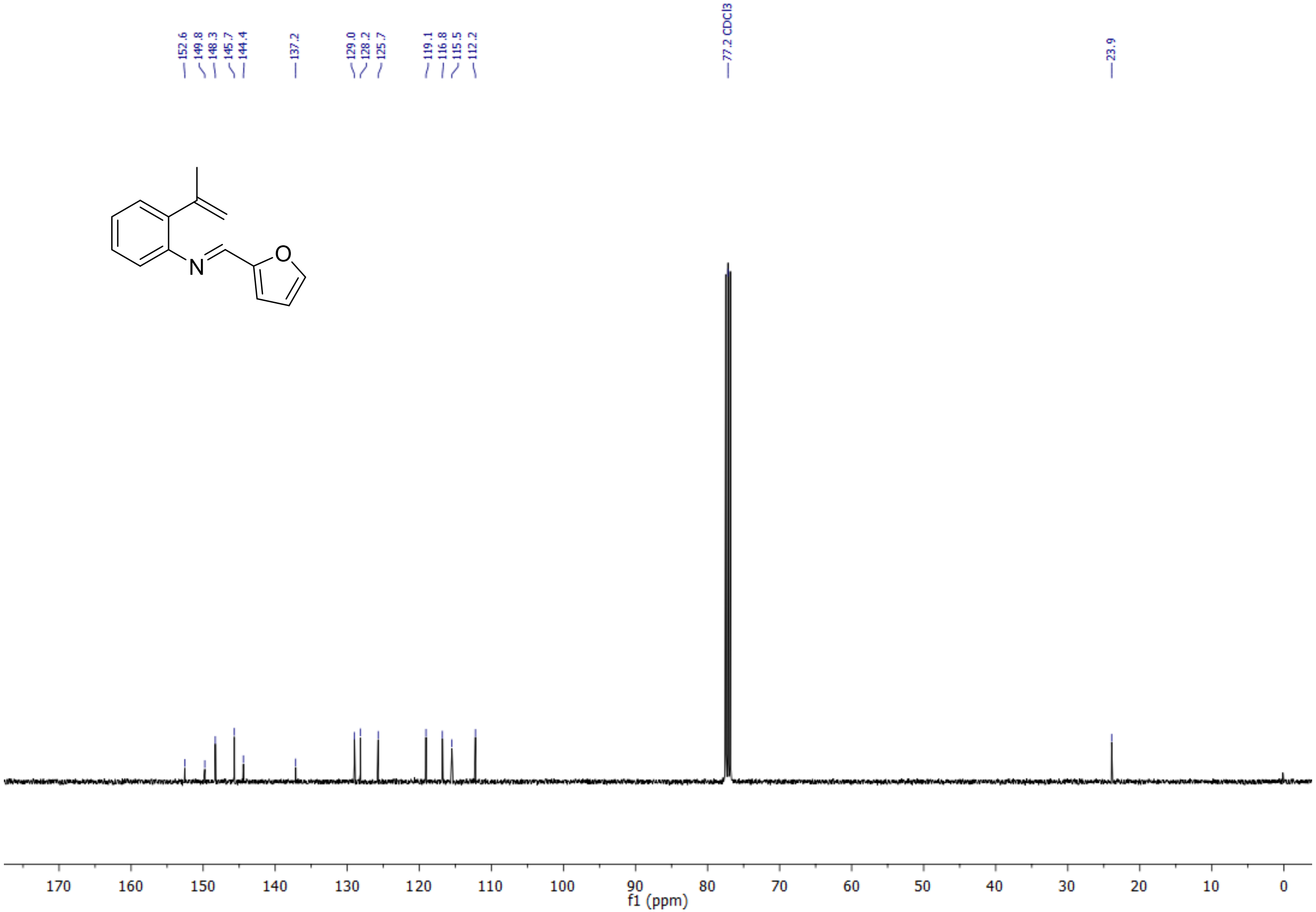

^{1}H–^{13}C HSQC-DEPT NMR (400 MHz, $CDCl_3$) of **1k**

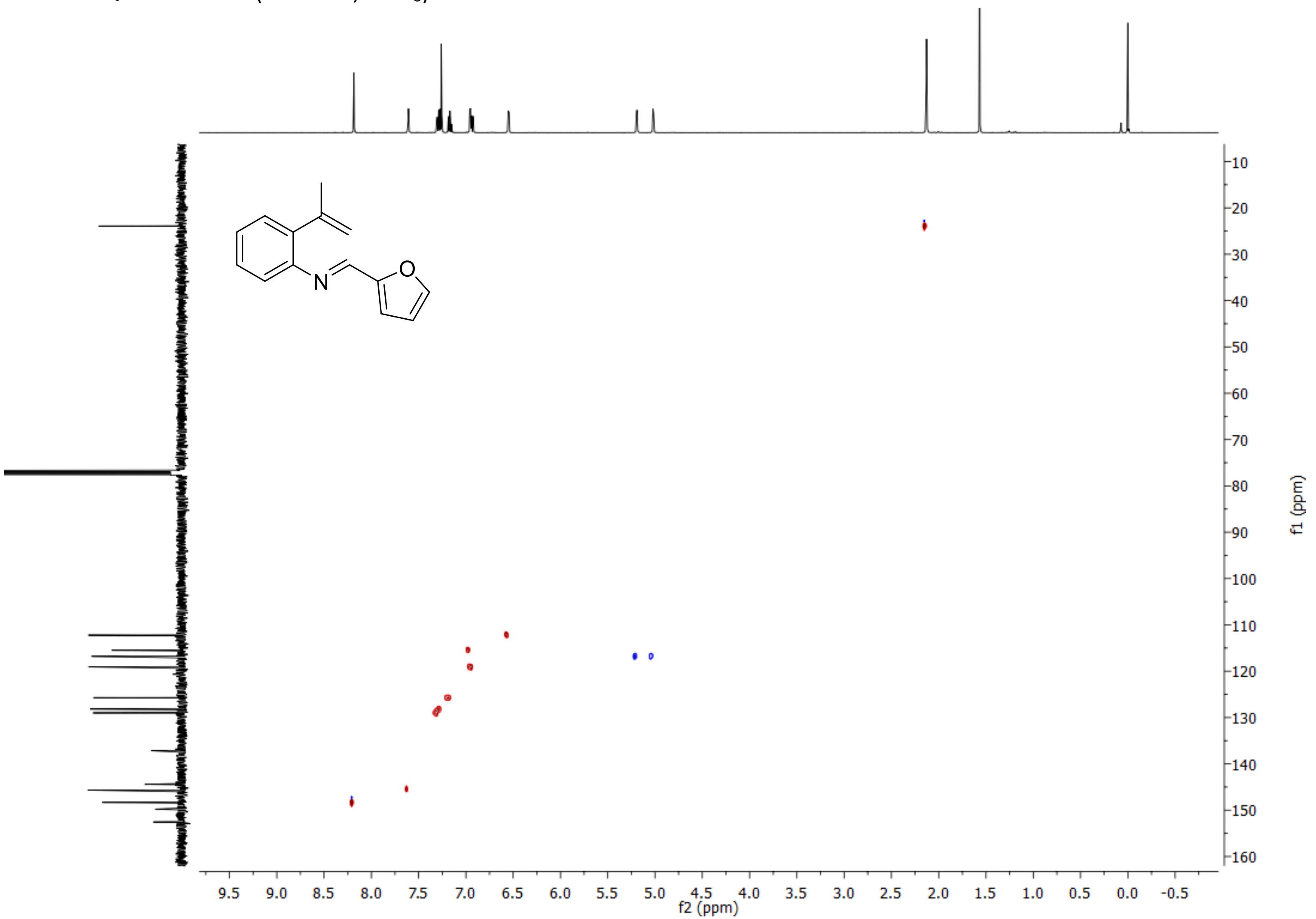

^{1}H NMR (400 MHz, $CDCl_3$) of **1l**

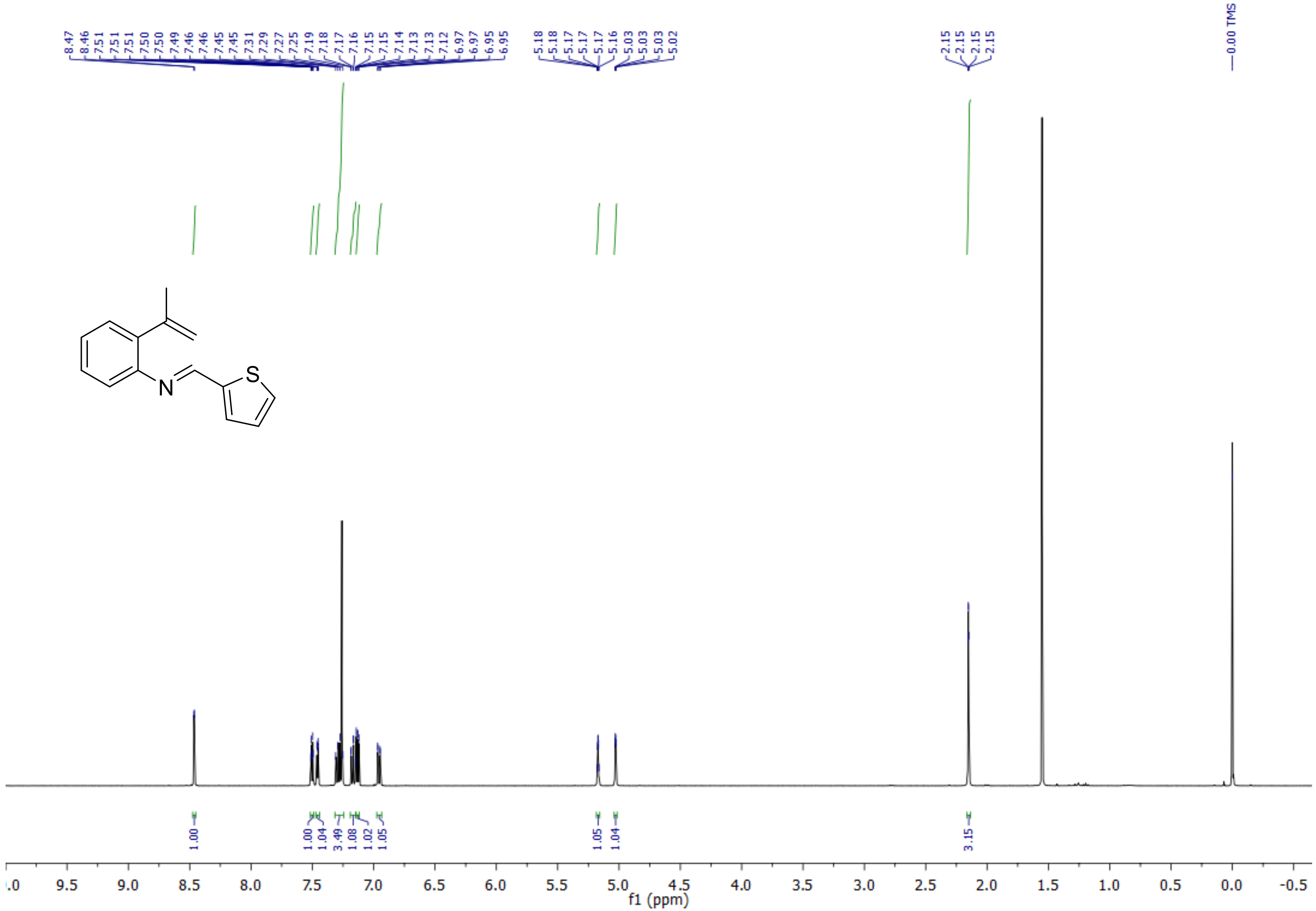

^{13}C NMR (101 MHz, $CDCl_3$) of **1l**

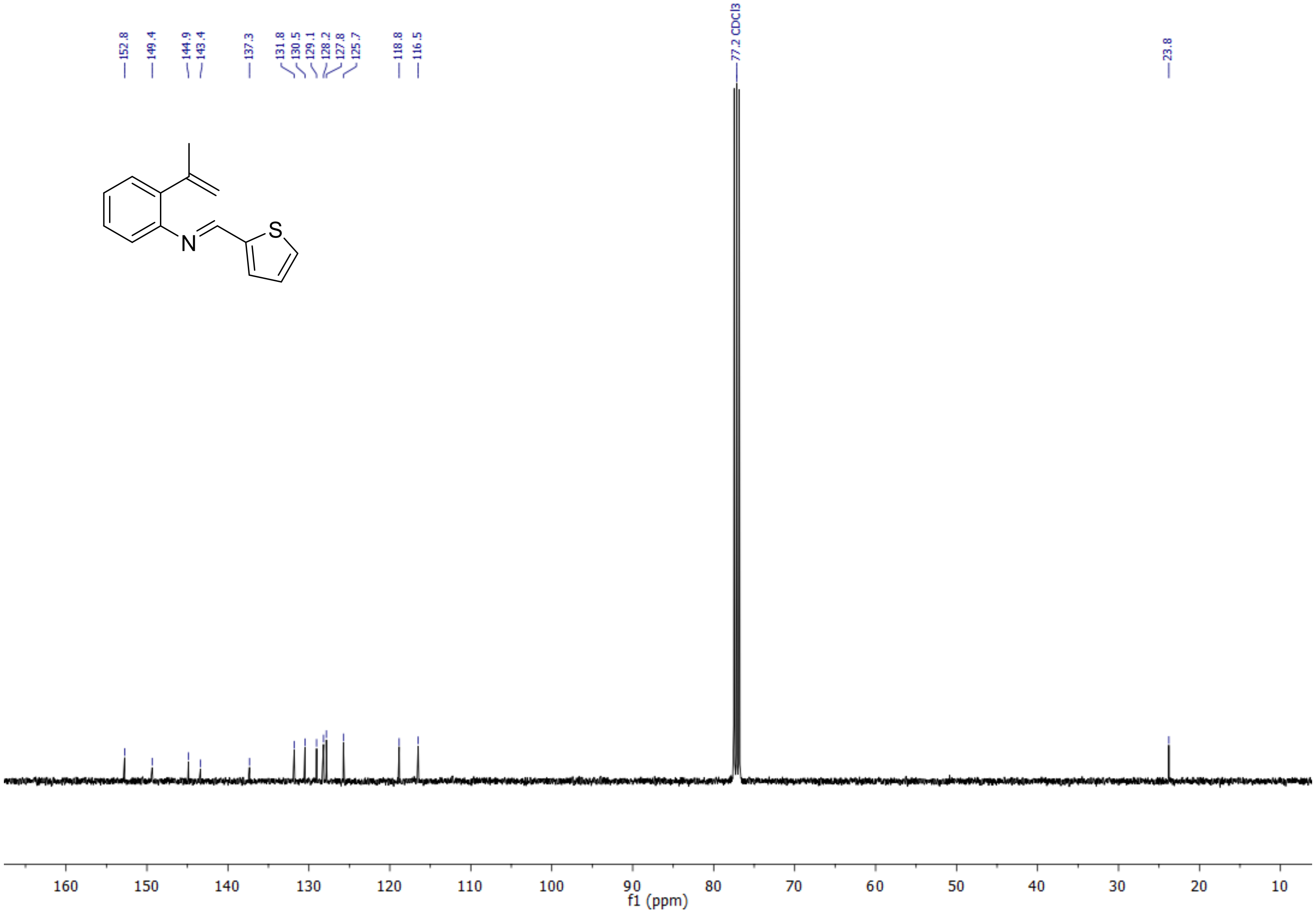

^{1}H–^{13}C HSQC-DEPT NMR (400 MHz, $CDCl_3$) of **1l**

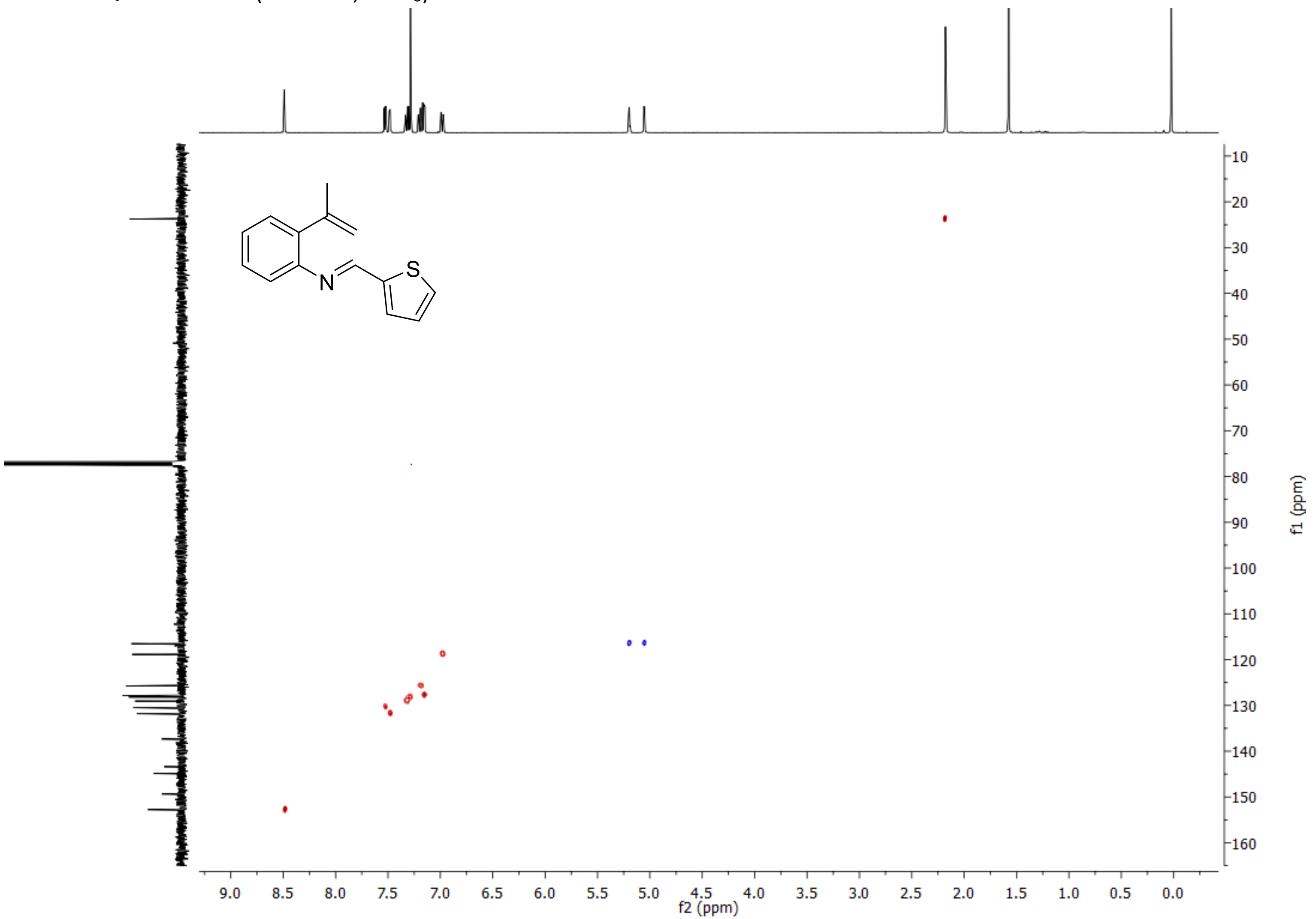

^{1}H NMR (400 MHz, $CDCl_3$) of **1m**

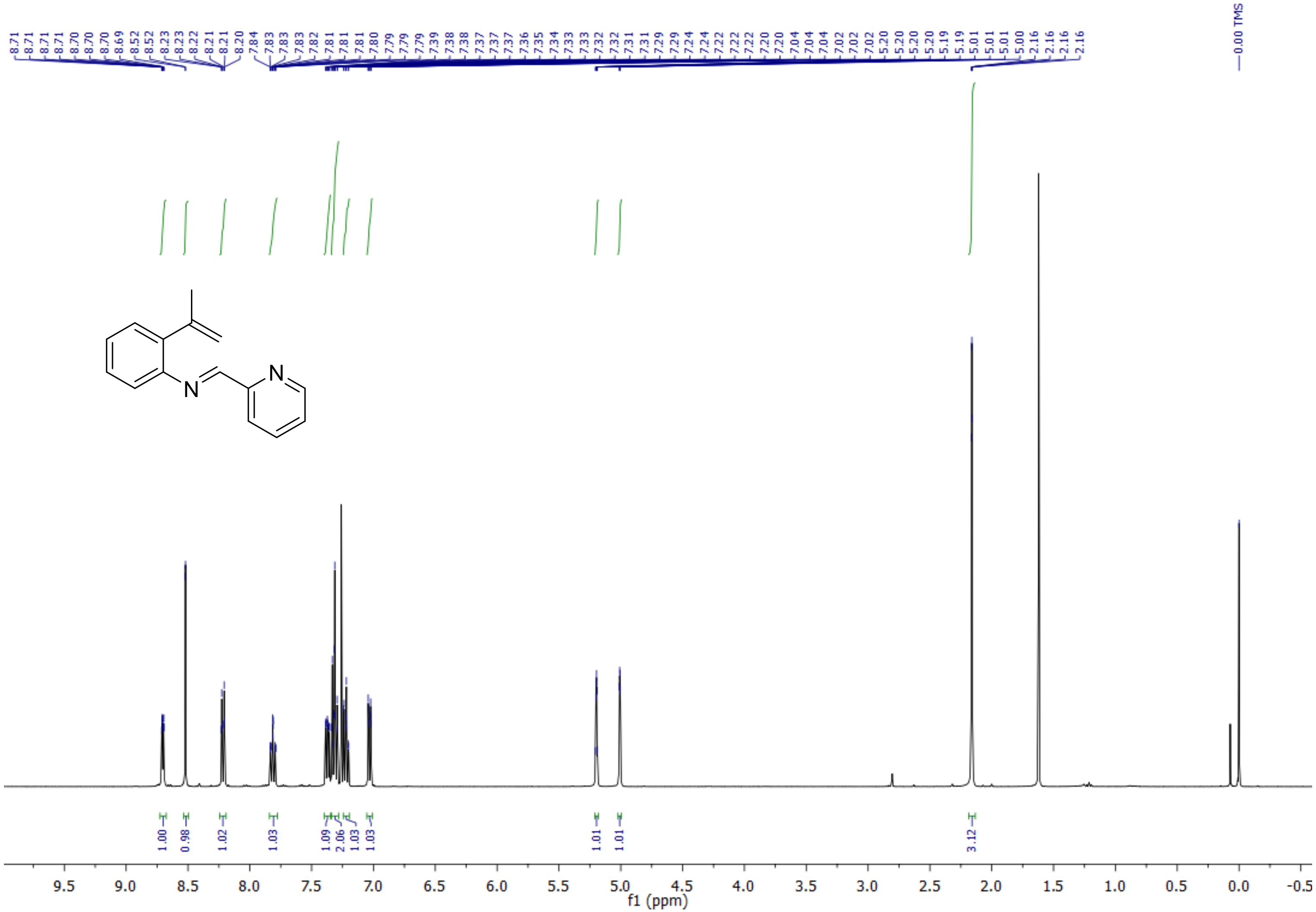

^{13}C NMR (101 MHz, $CDCl_3$) of **1m**

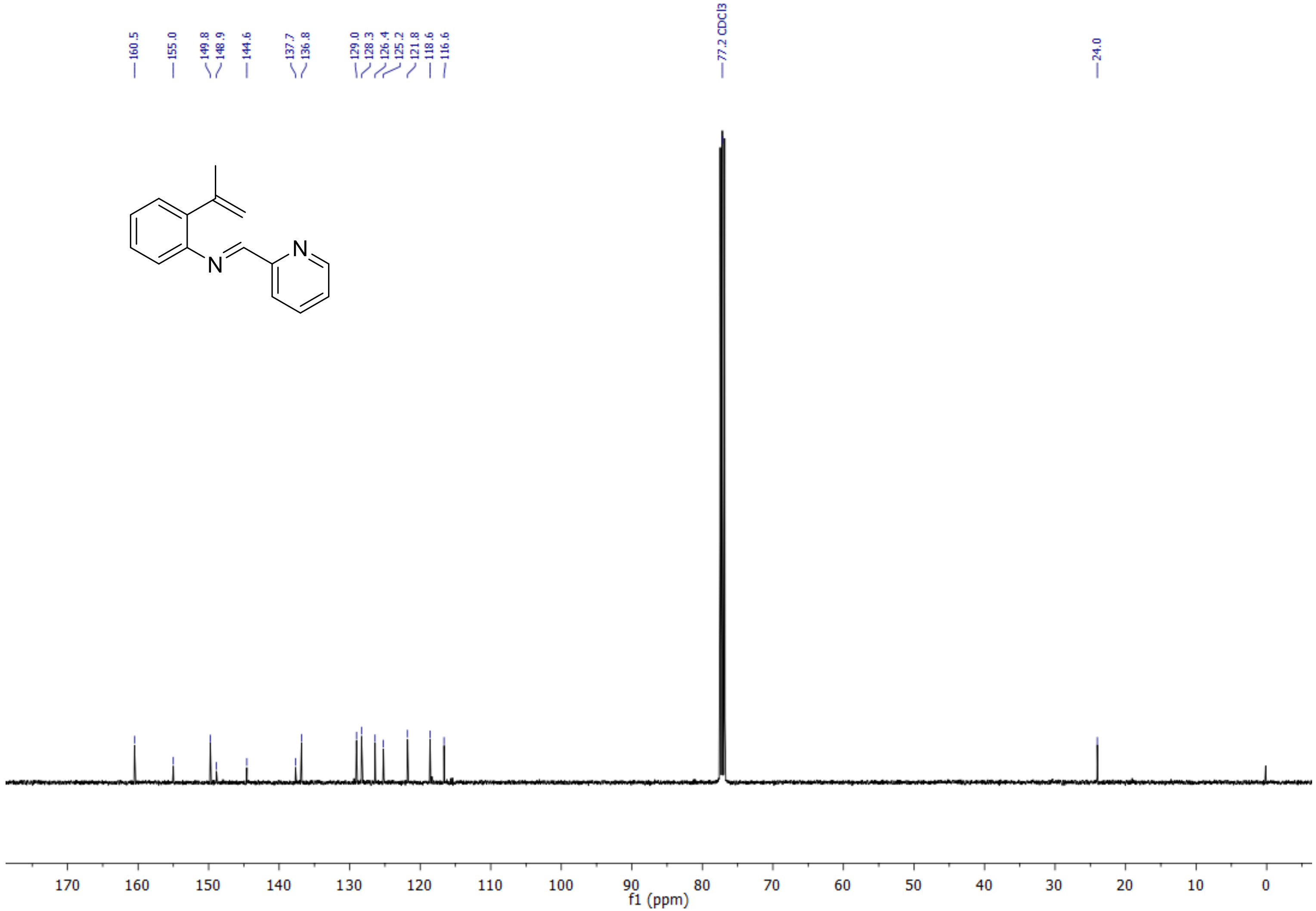

^{1}H–^{13}C HSQC-DEPT NMR (400 MHz, $CDCl_3$) of **1m**

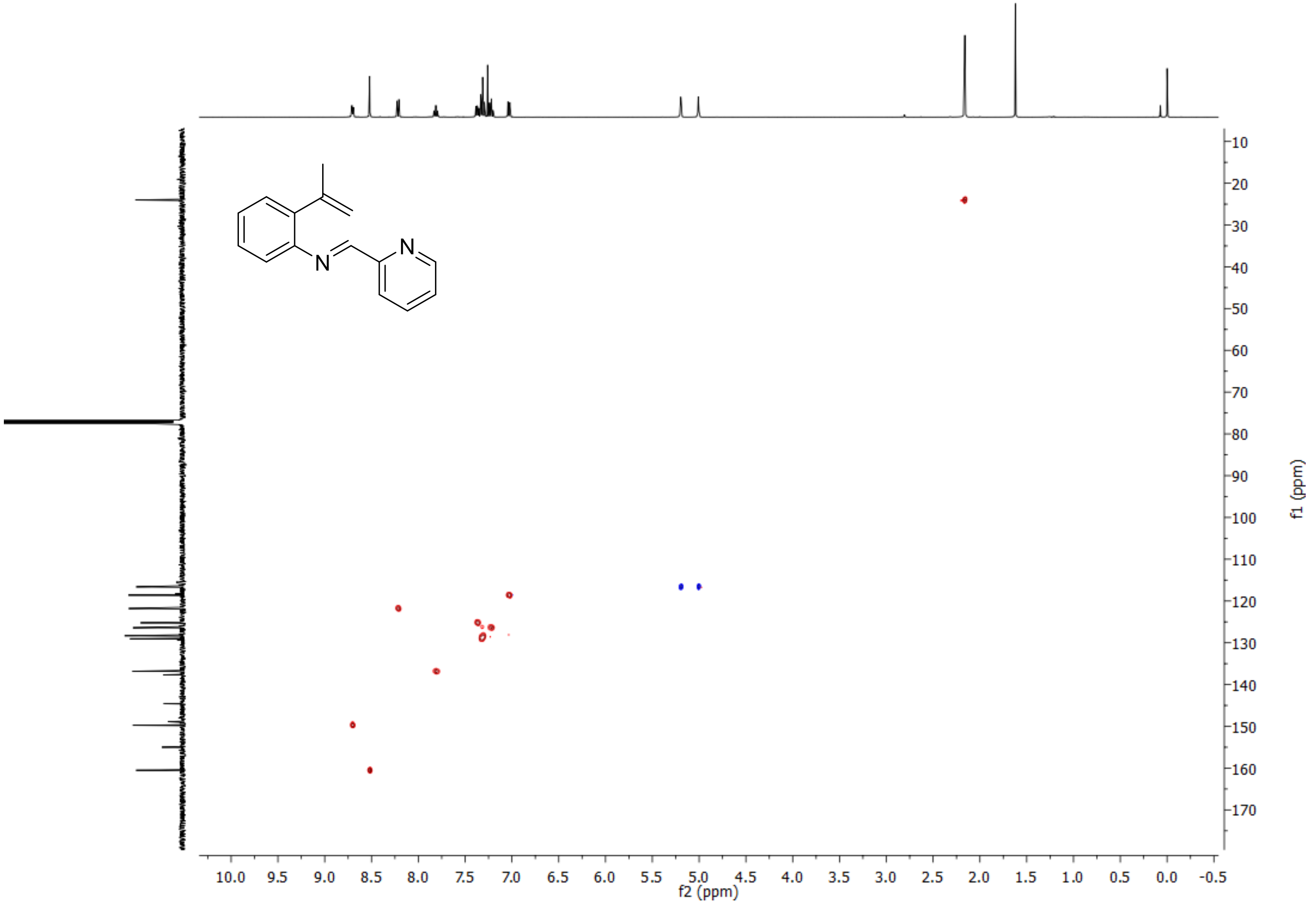

^{1}H NMR (400 MHz, $CDCl_3$) of **1n**

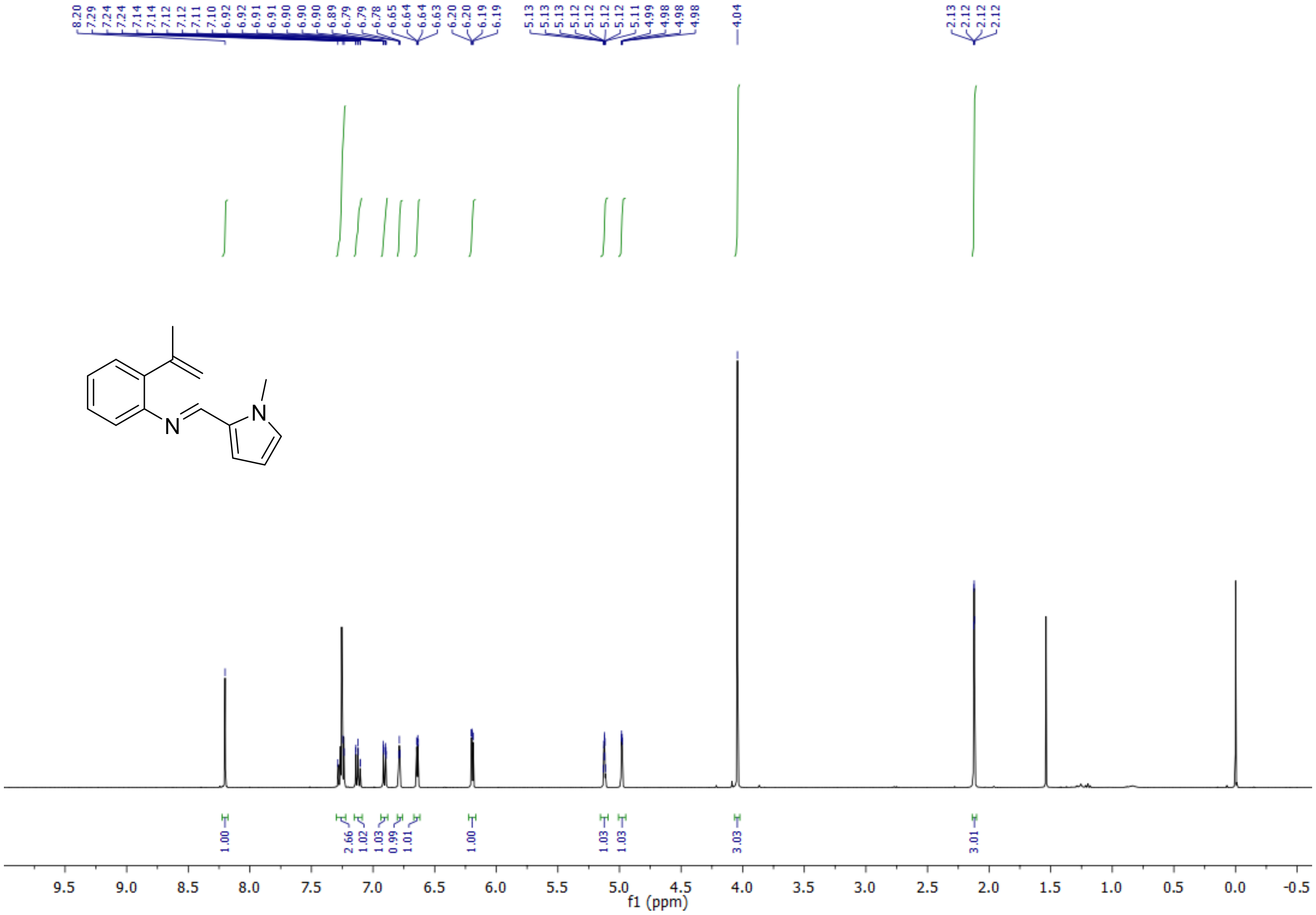

^{13}C NMR (101 MHz, $CDCl_3$) of **1n**

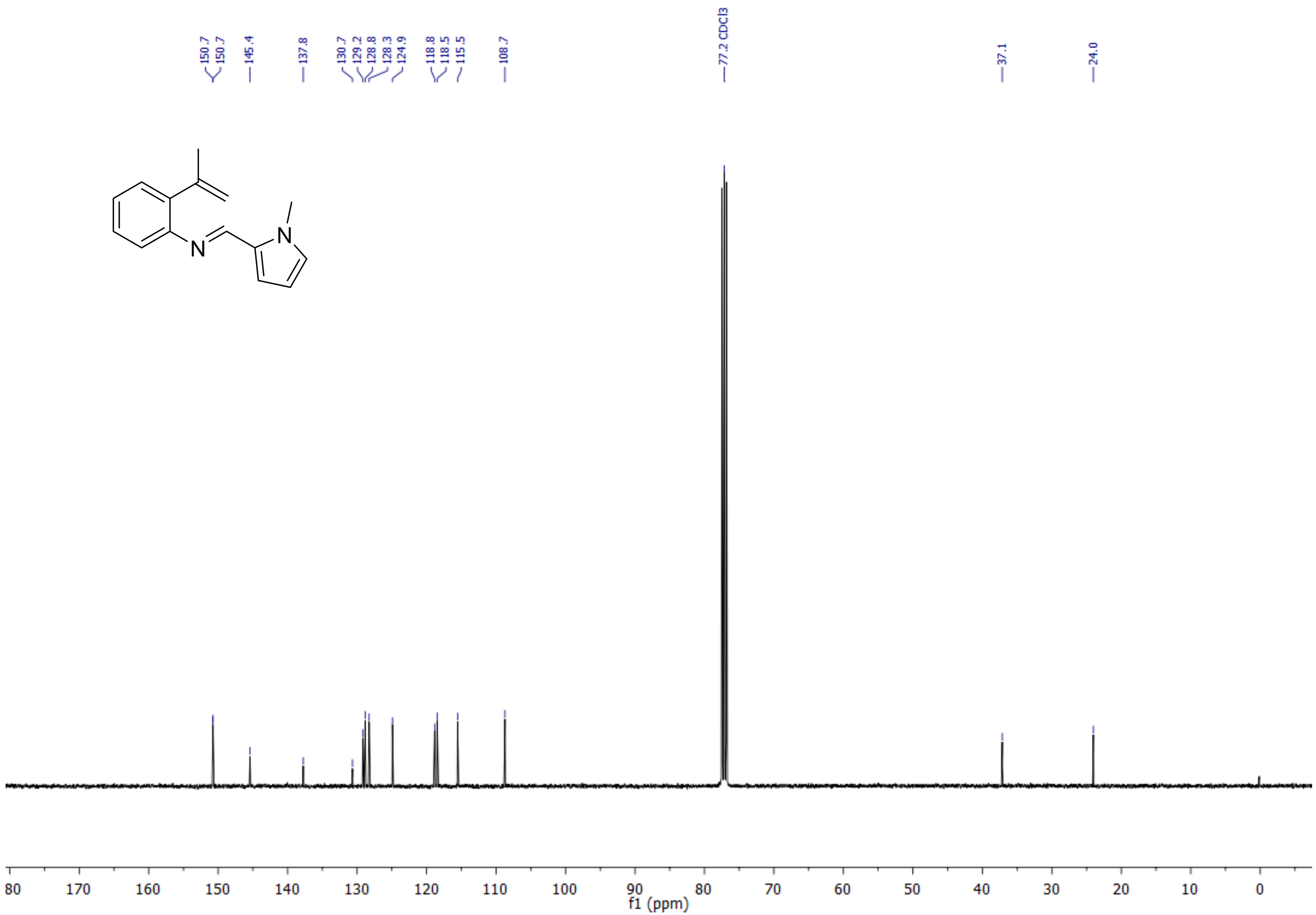

$^{1}H–^{13}C$ HSQC-DEPT NMR (400 MHz, $CDCl_3$) of **1n**

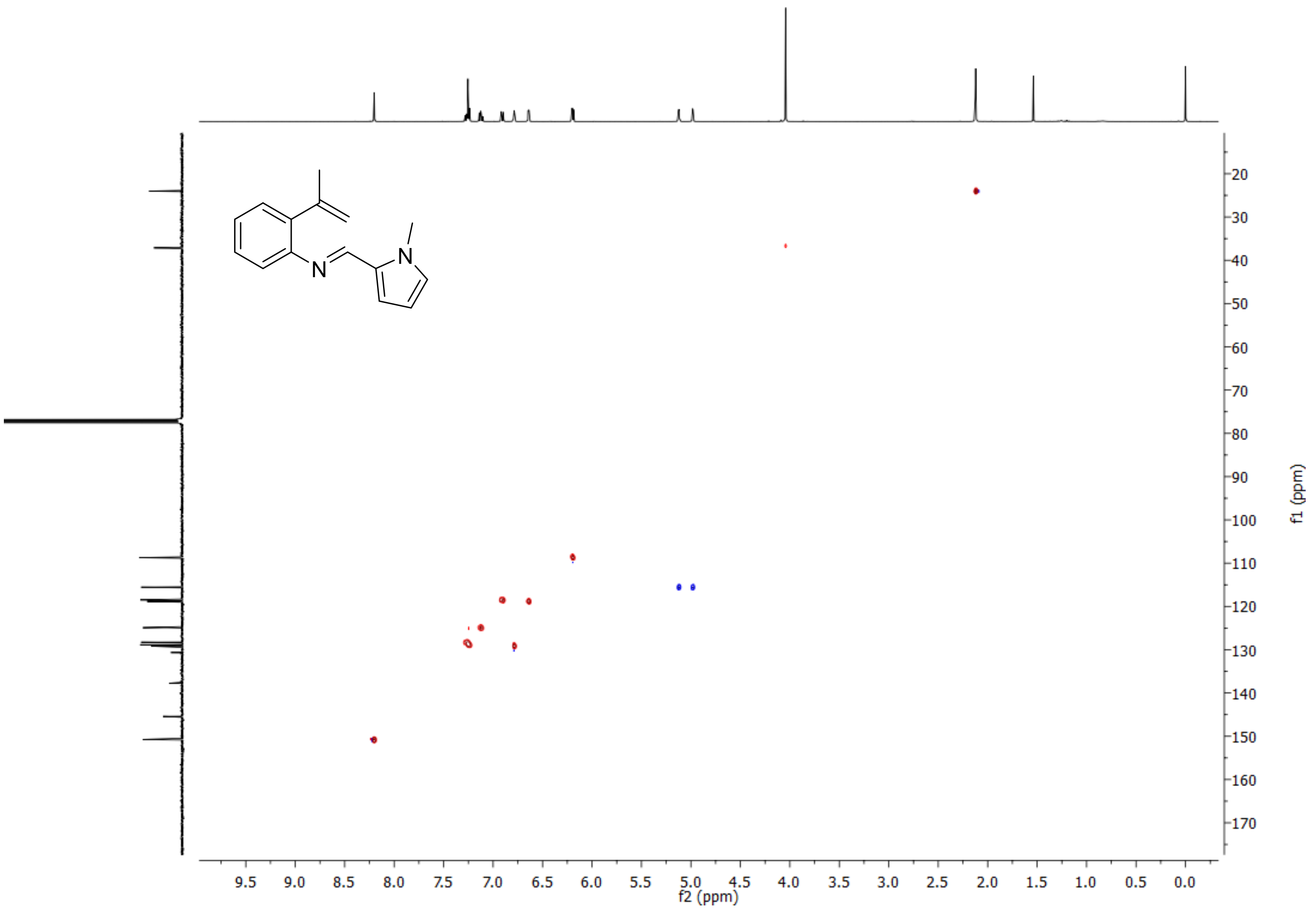

^{1}H NMR (400 MHz, acetone-d_6) of **1o**

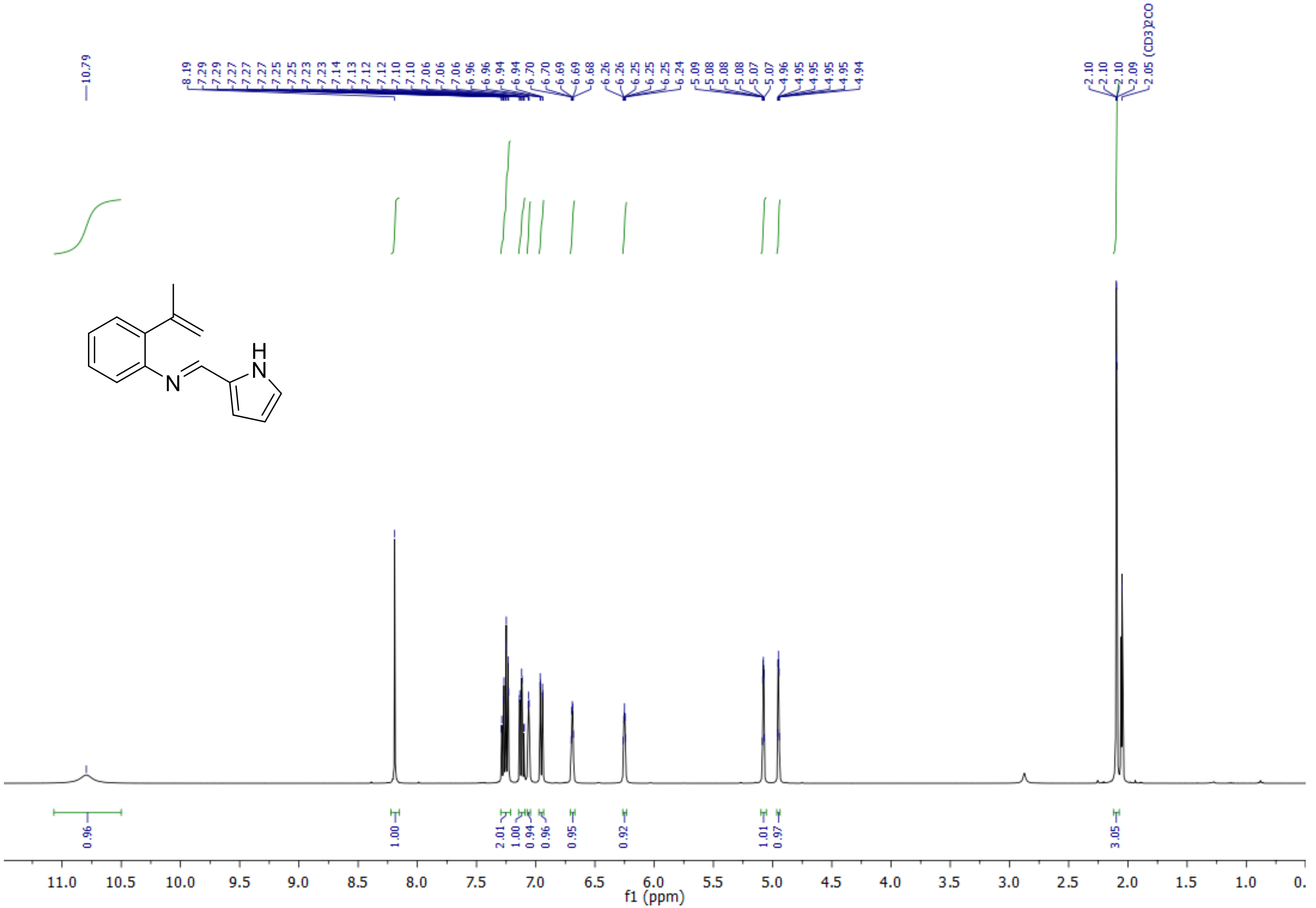

^{13}C NMR (101 MHz, acetone-d_6) of **1o**

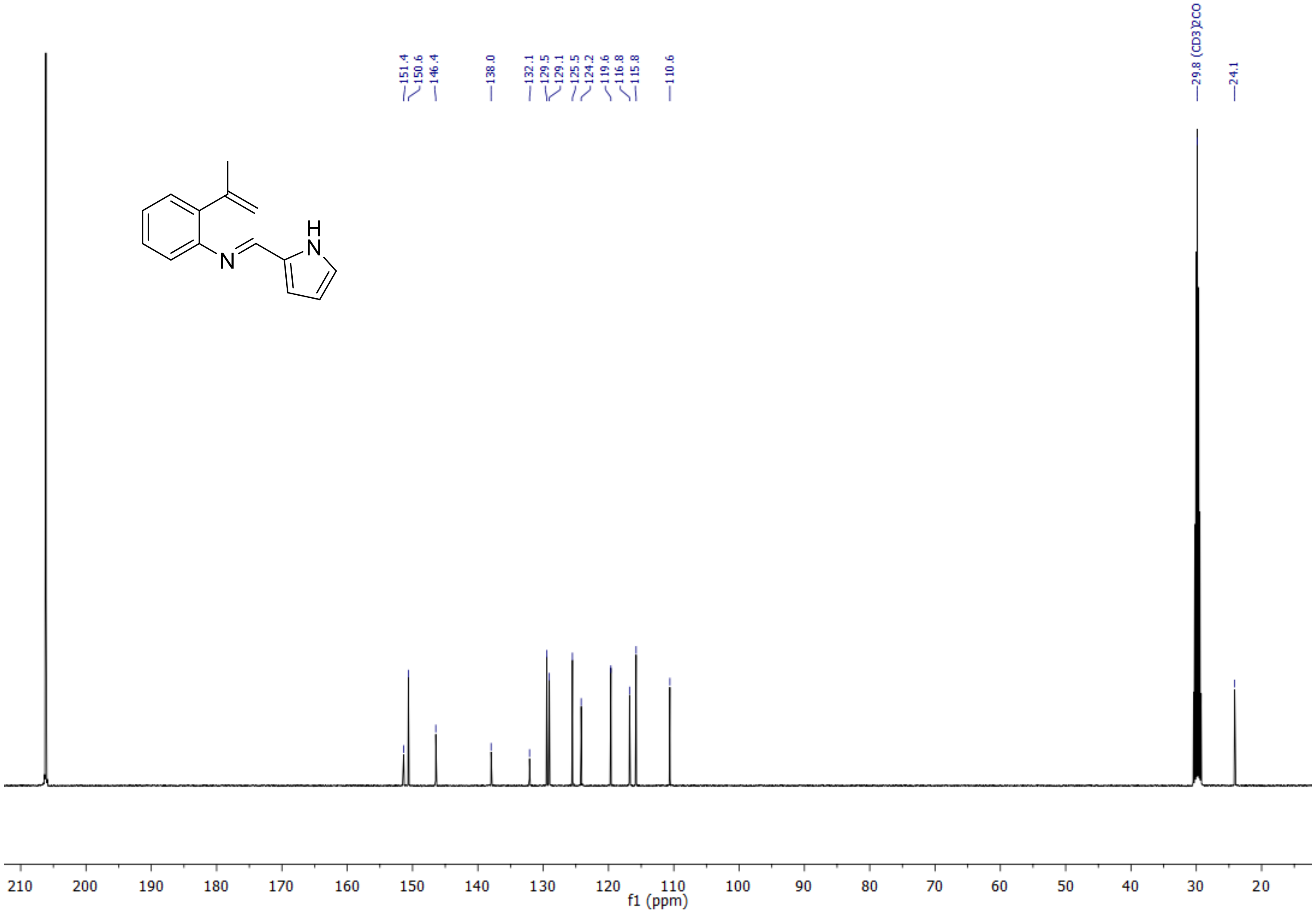

^{1}H–^{13}C HSQC-DEPT NMR (400 MHz, acetone-d_6) of **1o**

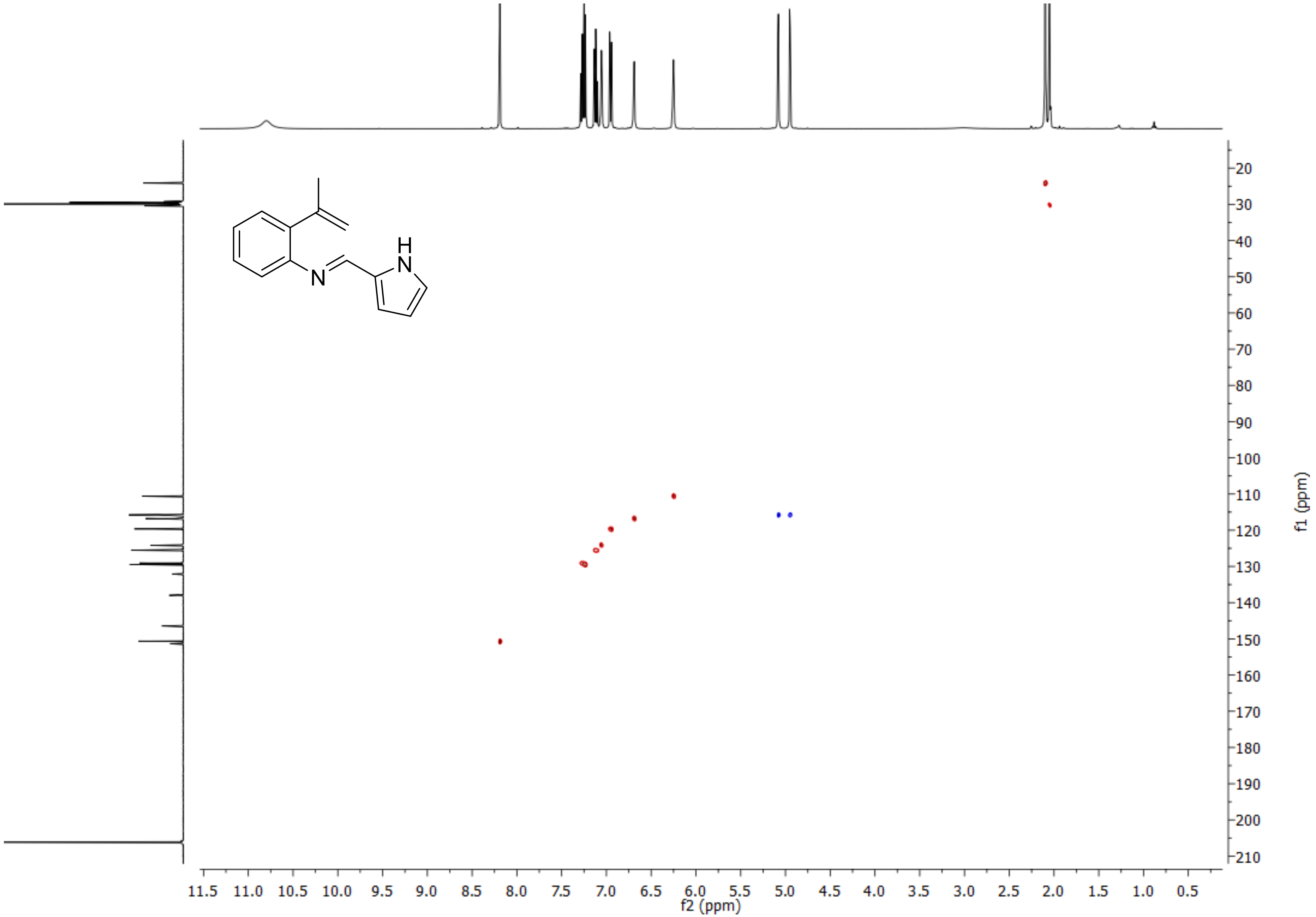

^{1}H NMR (400 MHz, $CDCl_3$) of **1p**

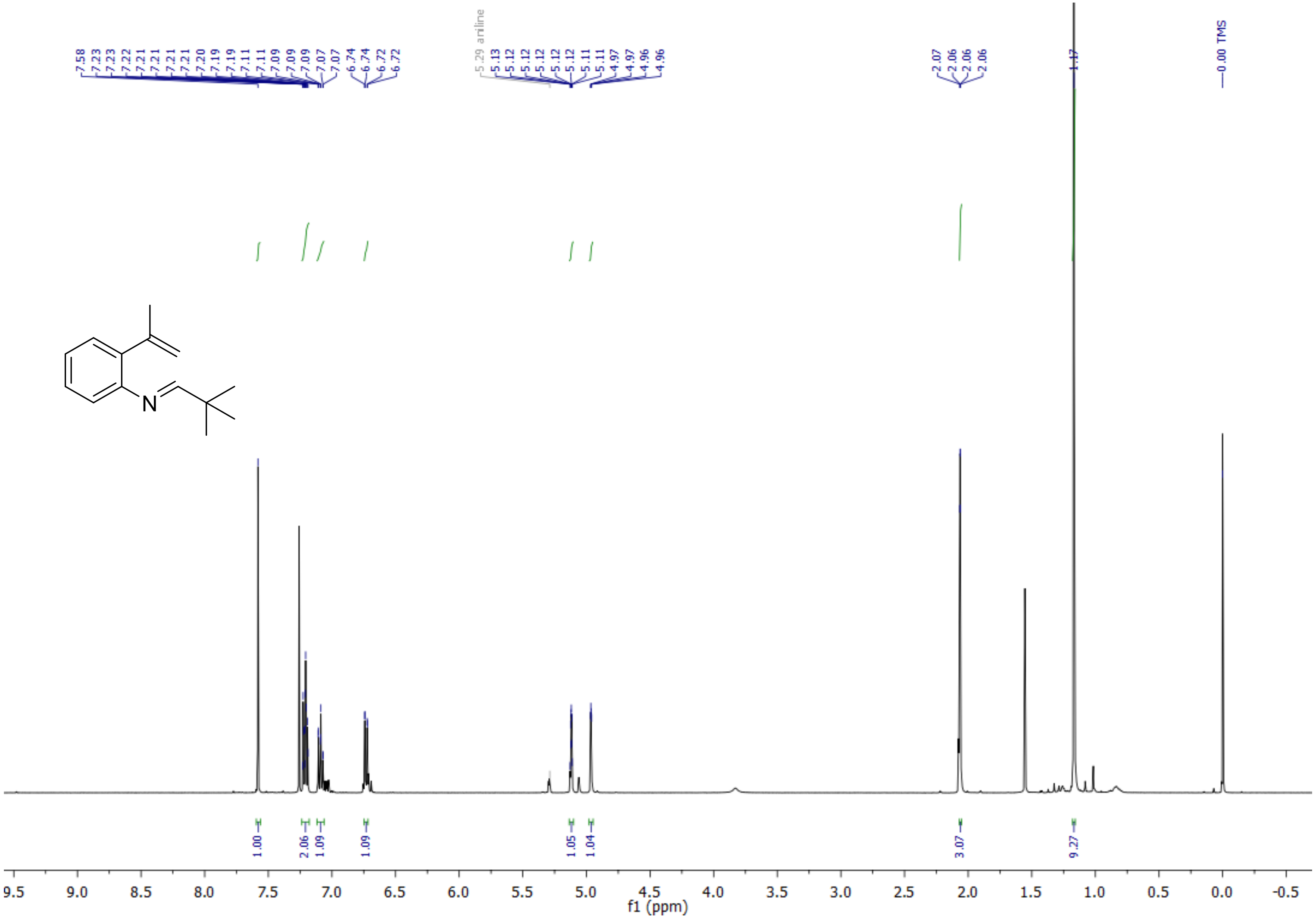

^{13}C NMR (101 MHz, $CDCl_3$) of **1p**

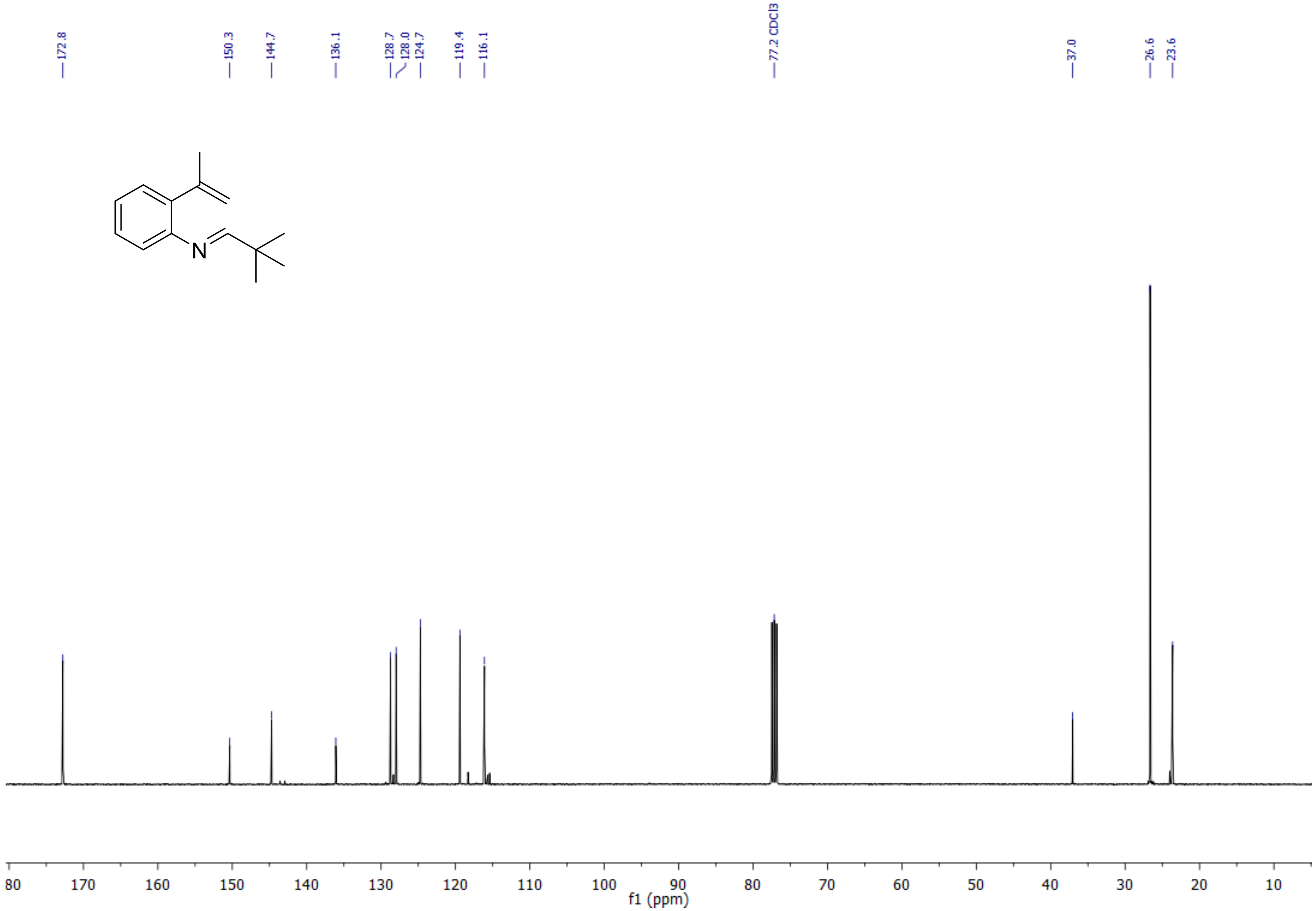

$^{1}H–^{13}C$ HSQC-DEPT NMR (400 MHz, $CDCl_3$) of **1p**

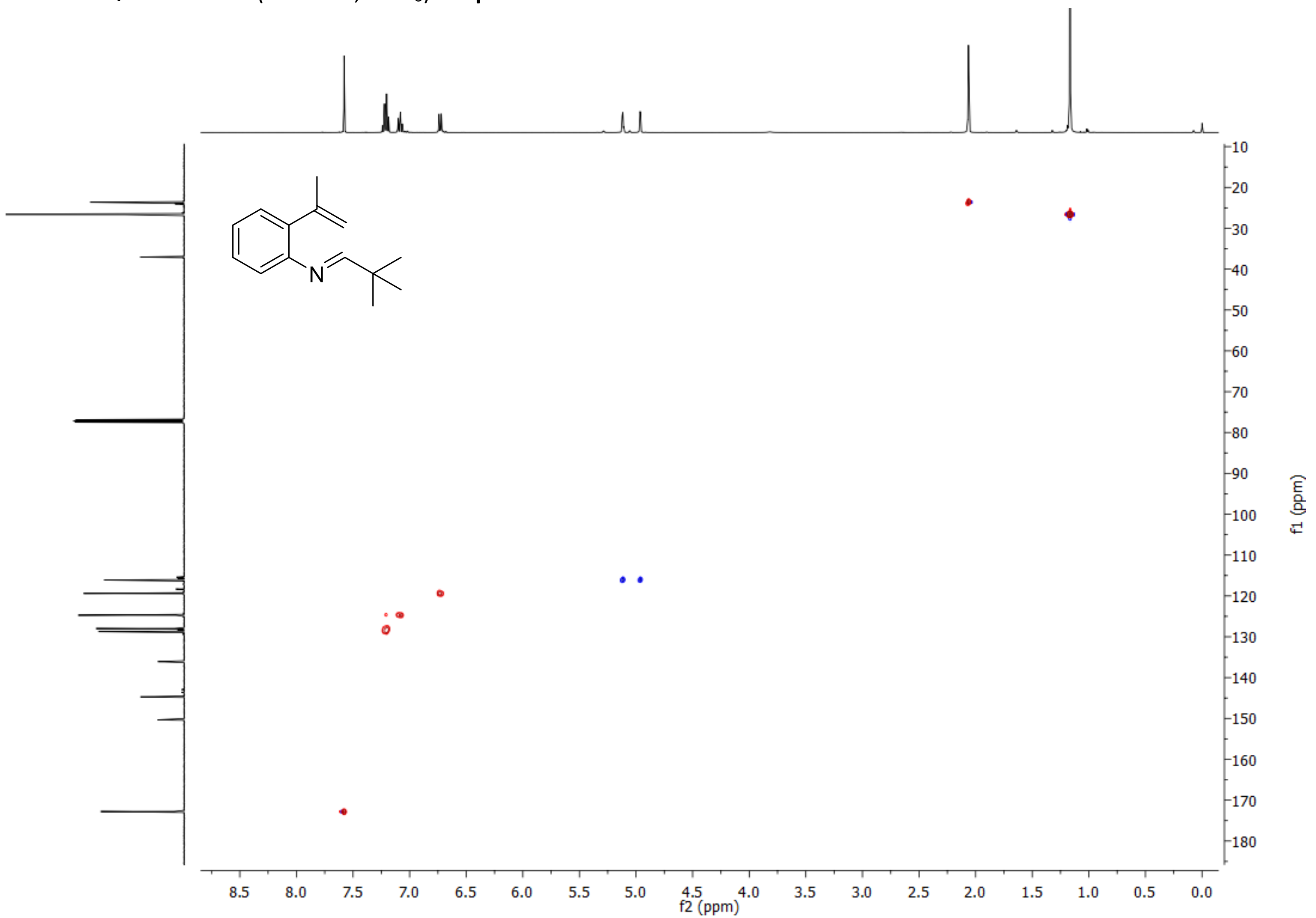

^{1}H NMR (400 MHz, acetone-d_6) of **1q**

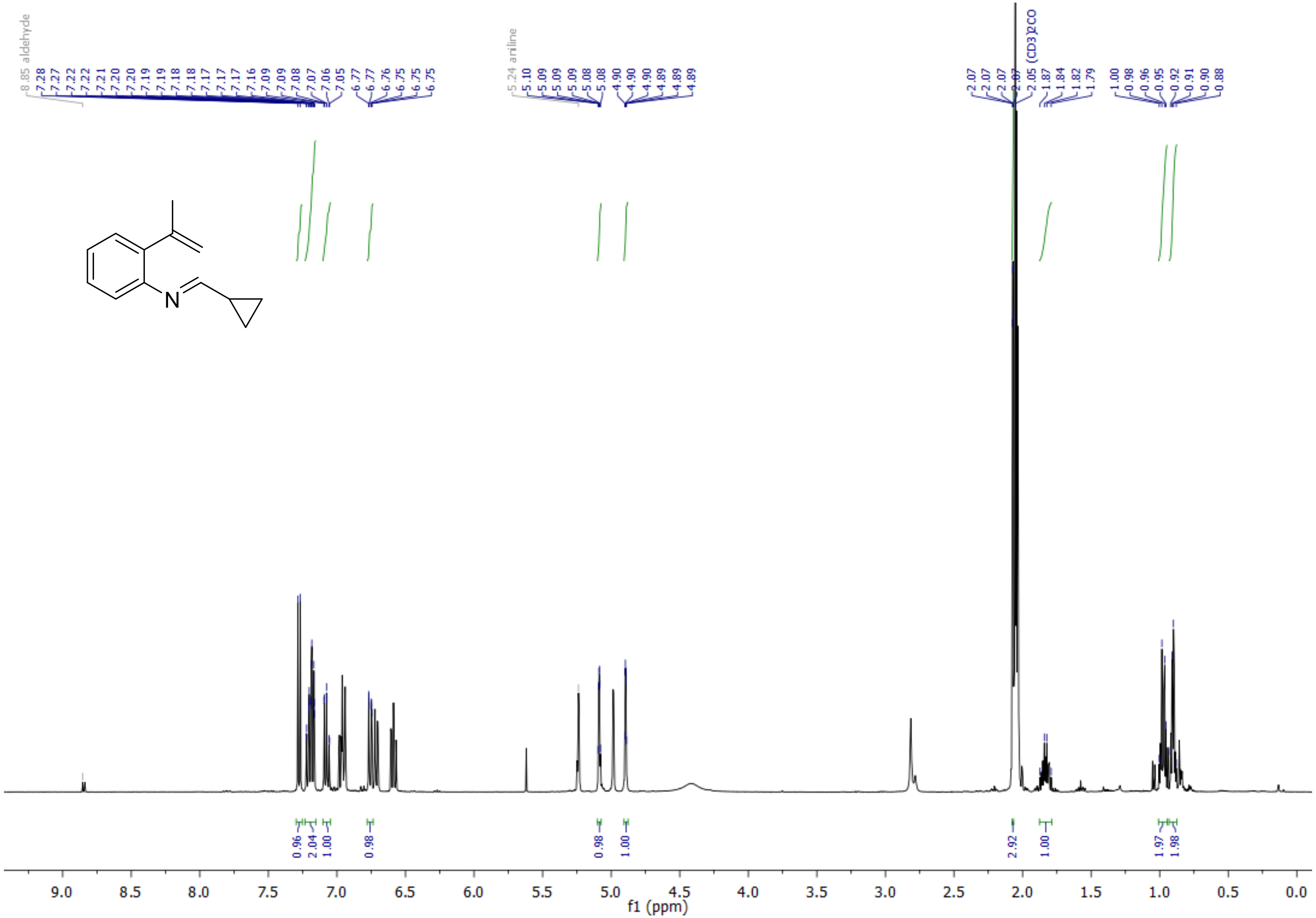

^{13}C NMR (101 MHz, acetone-d_6) of **1q**

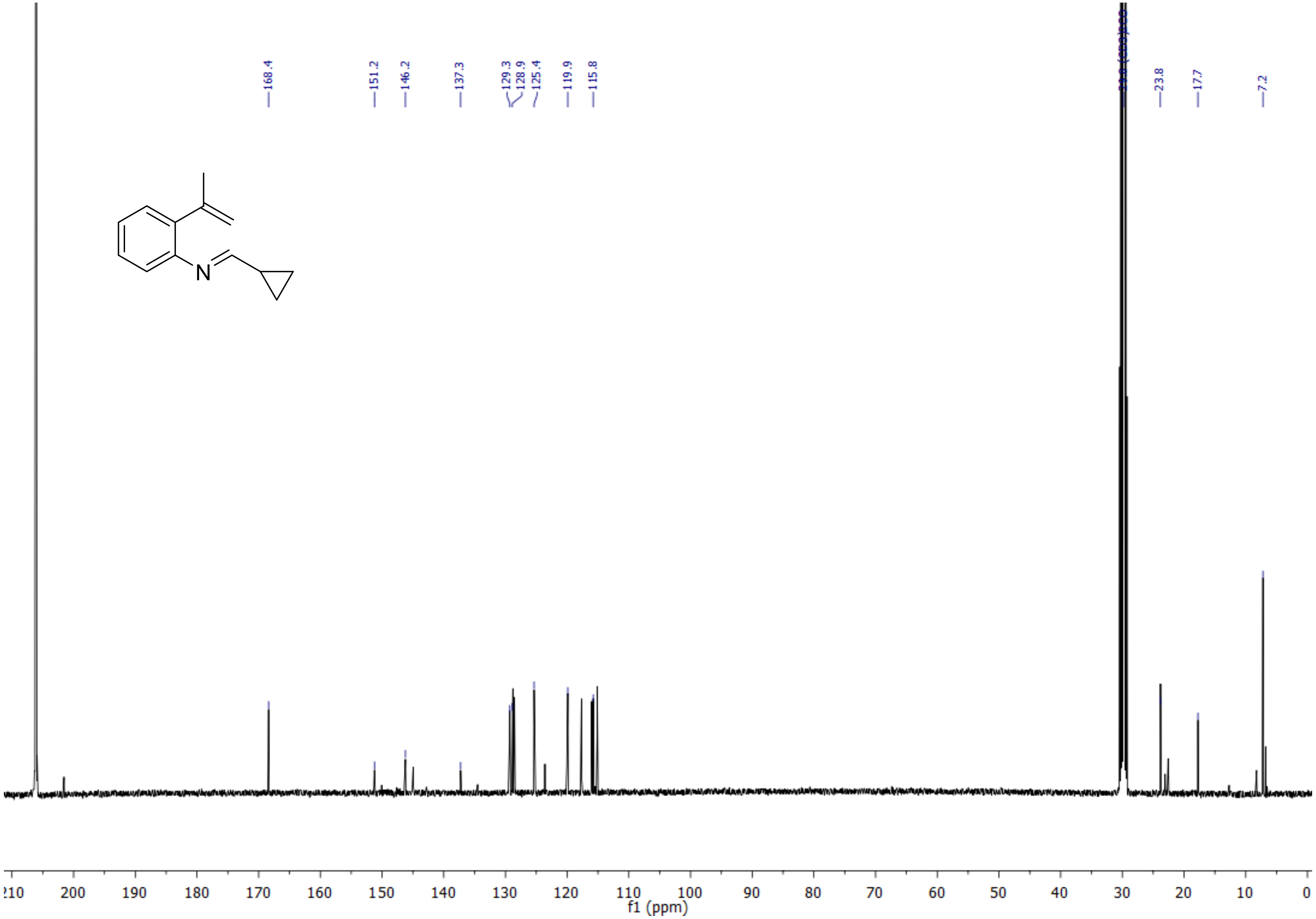

$^{1}H–^{13}C$ HSQC-DEPT NMR (400 MHz, acetone-d_6) of **1q**

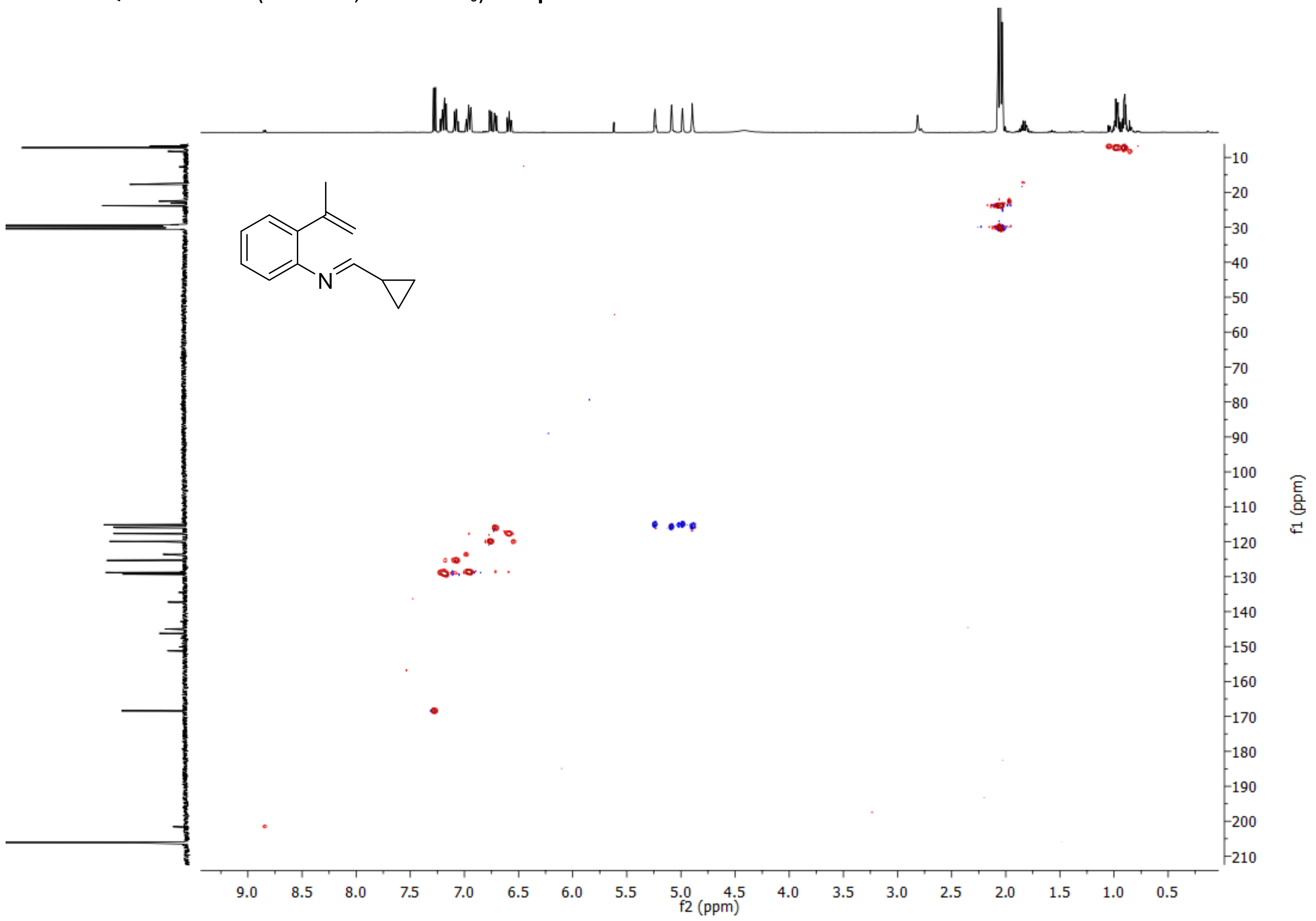

^{1}H NMR (400 MHz, acetone-d_6) of **1s**

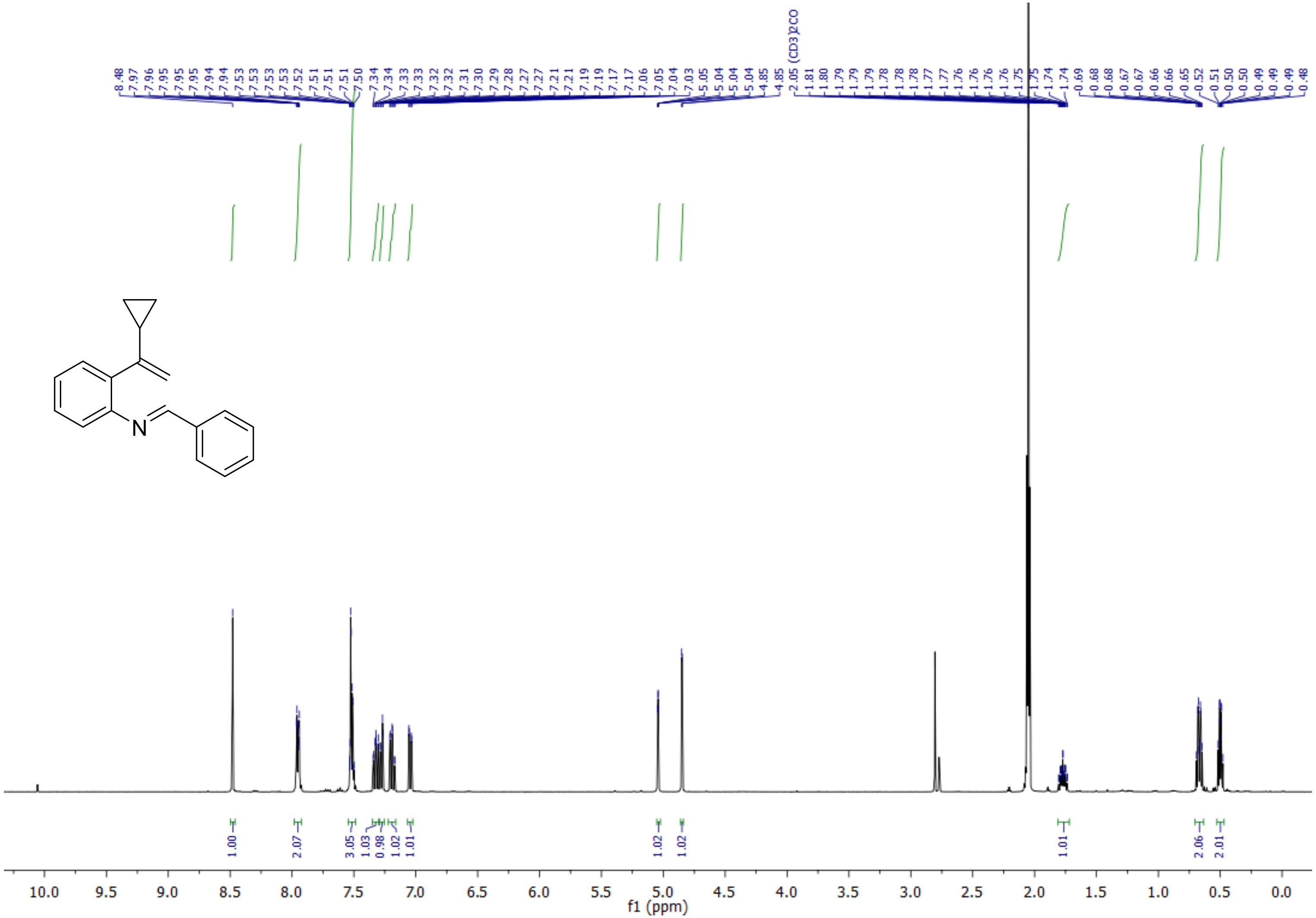

^{13}C NMR (101 MHz, acetone-d_6) of **1s**

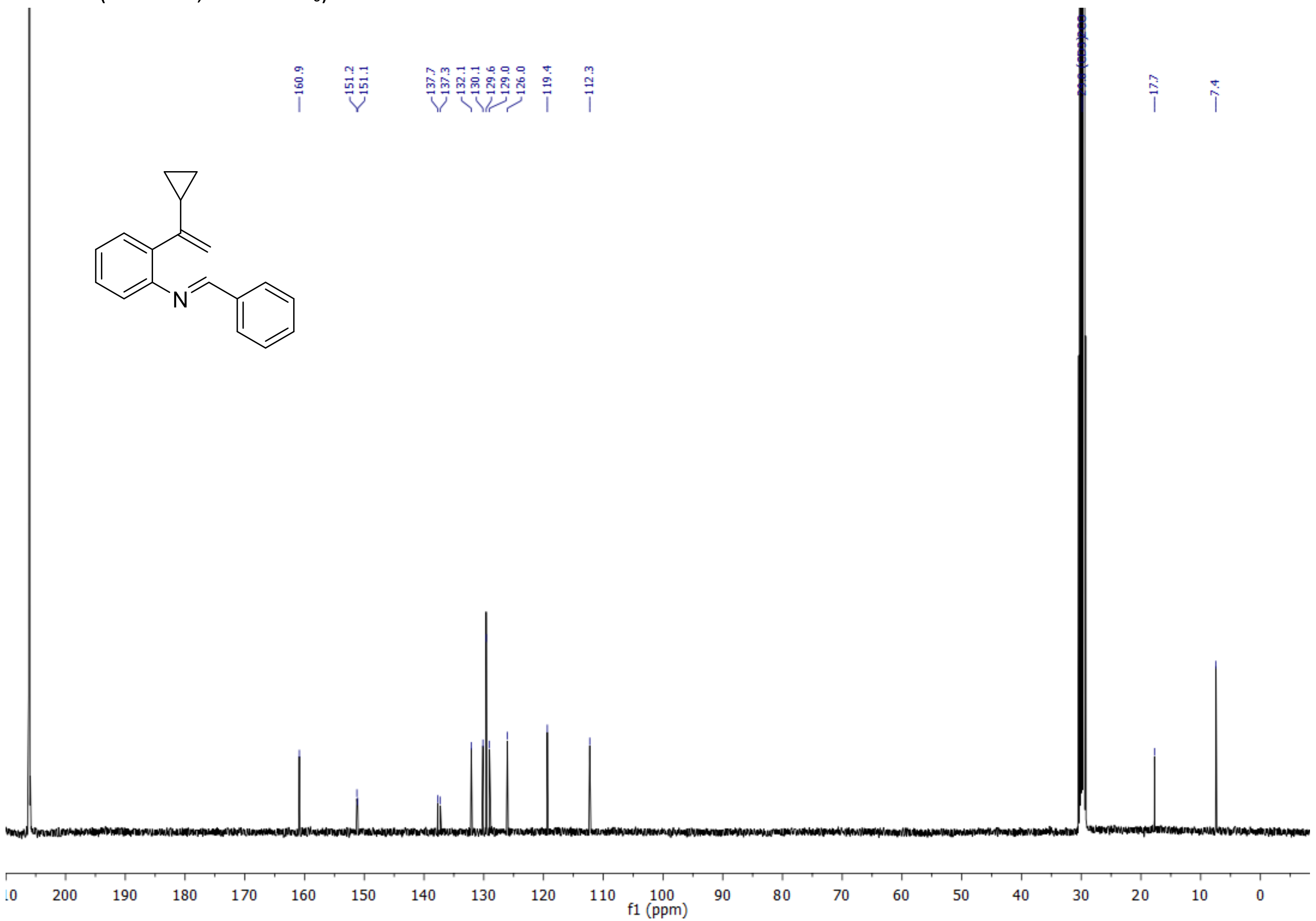

^{1}H–^{13}C HSQC-DEPT NMR (400 MHz, acetone-d_6) of **1s**

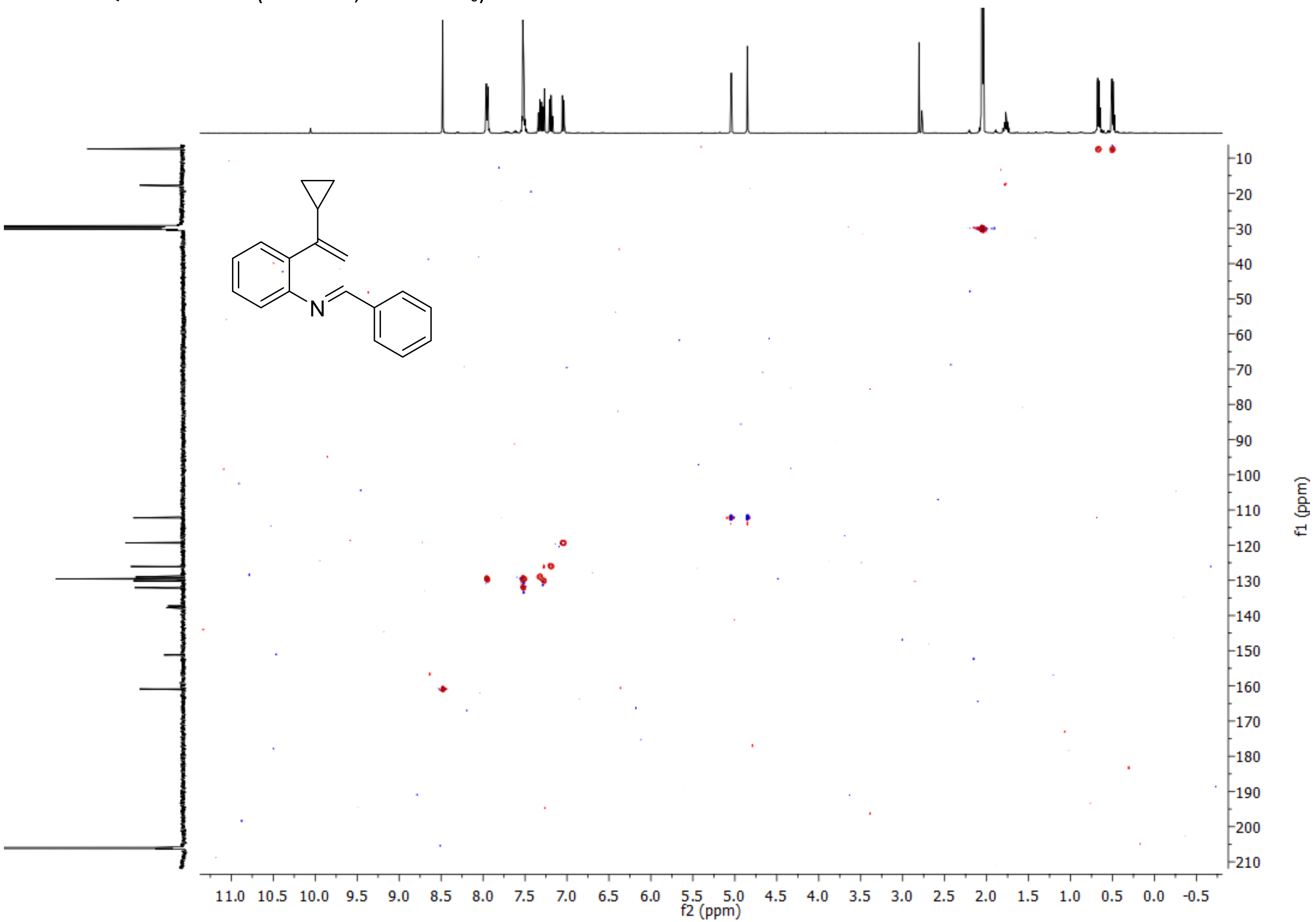

^{1}H NMR (400 MHz, acetone-d_6) of **1u**

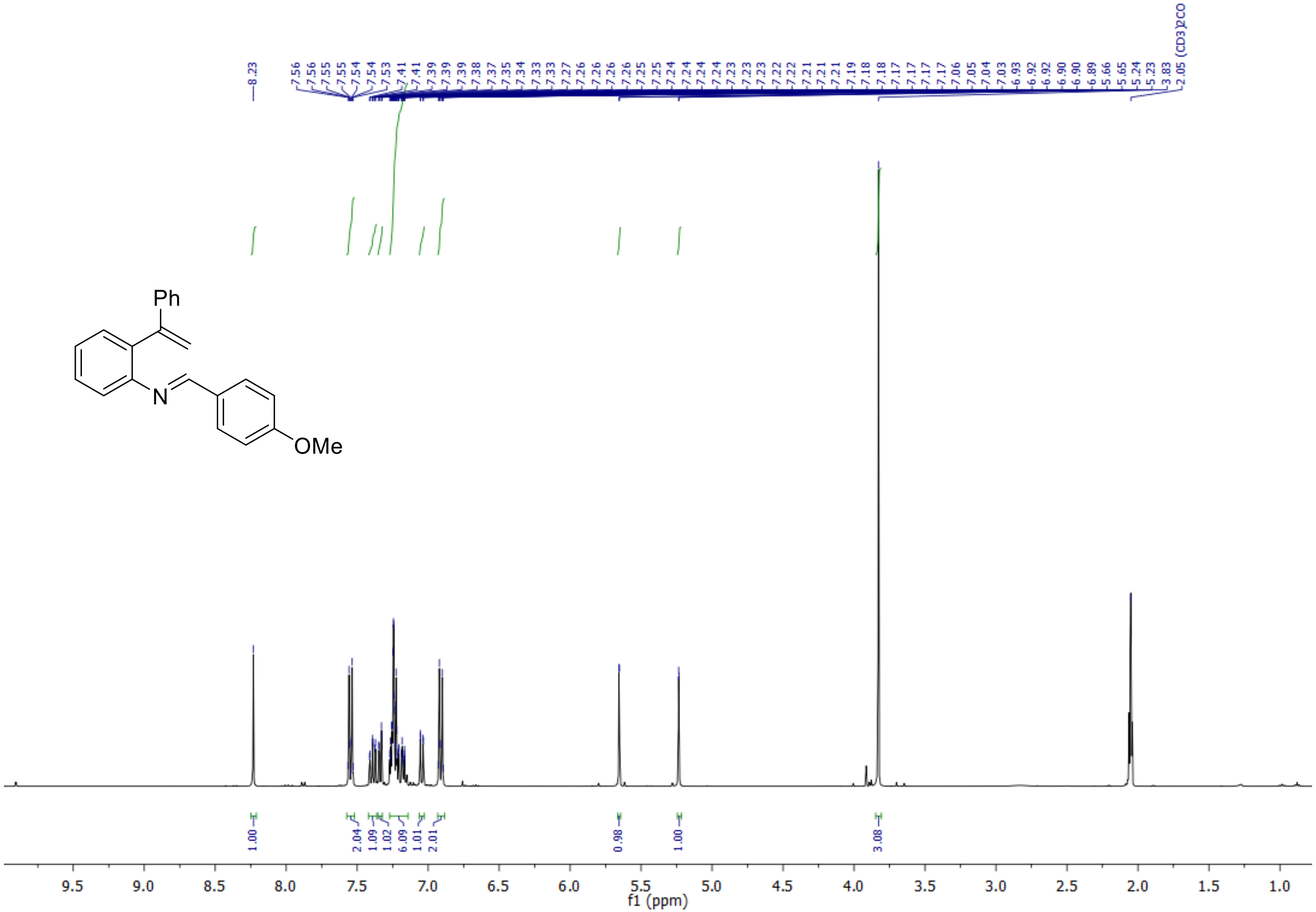

^{13}C NMR (101 MHz, acetone-d_6) of **1u**

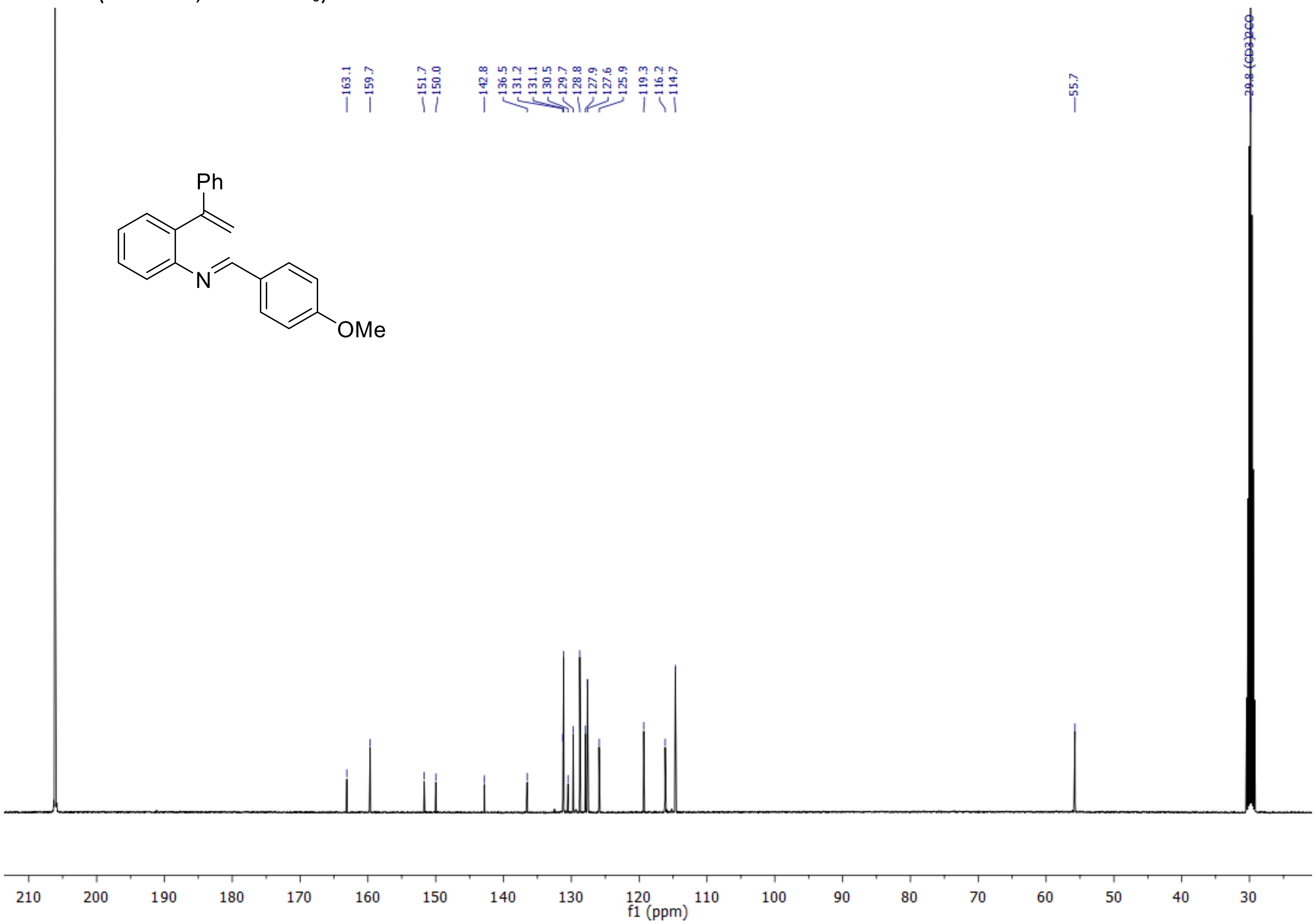

^{1}H–^{13}C HSQC-DEPT NMR (400 MHz, acetone-d_6) of **1u**

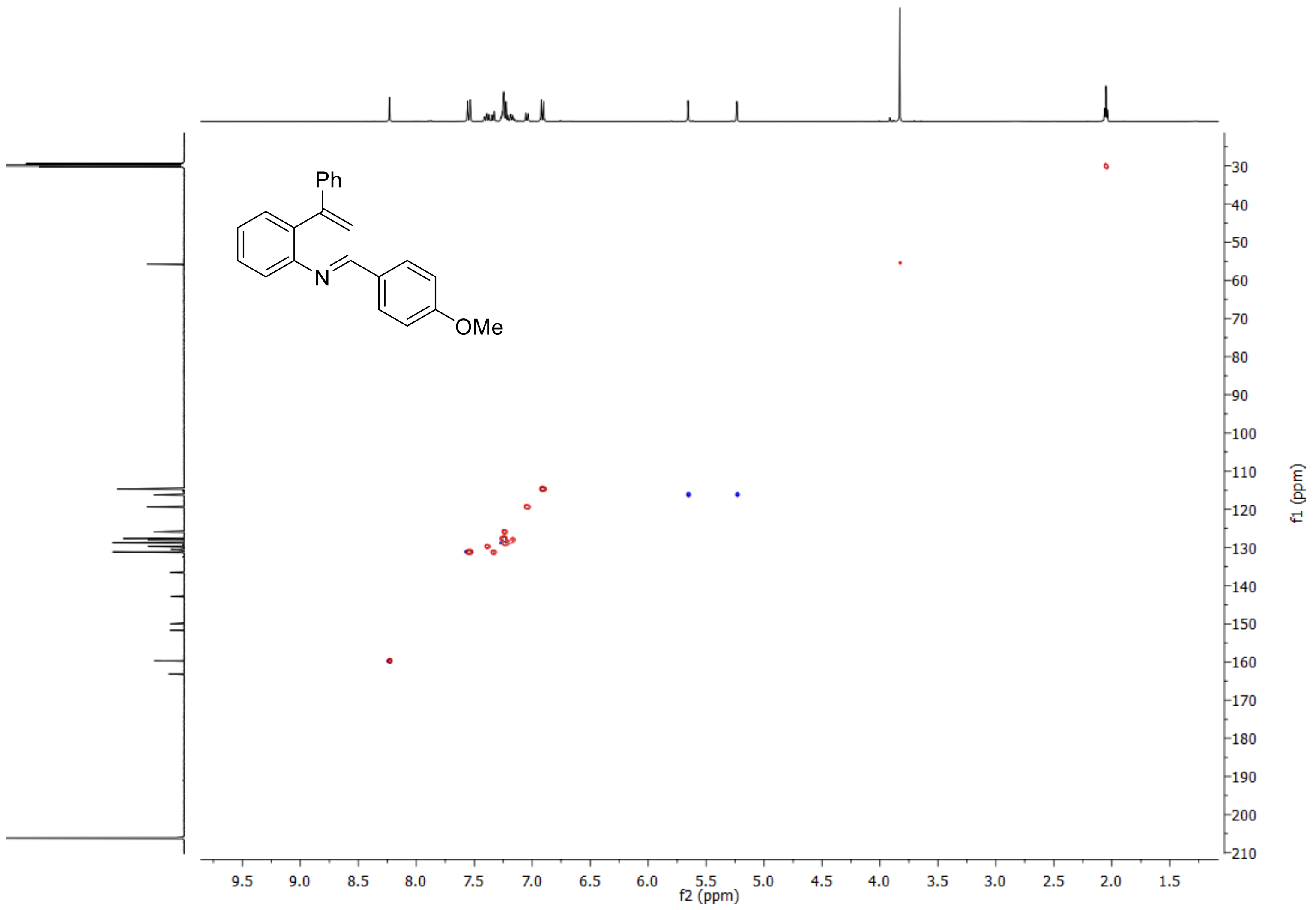

^{1}H NMR (400 MHz, $CDCl_3$) of **1v**

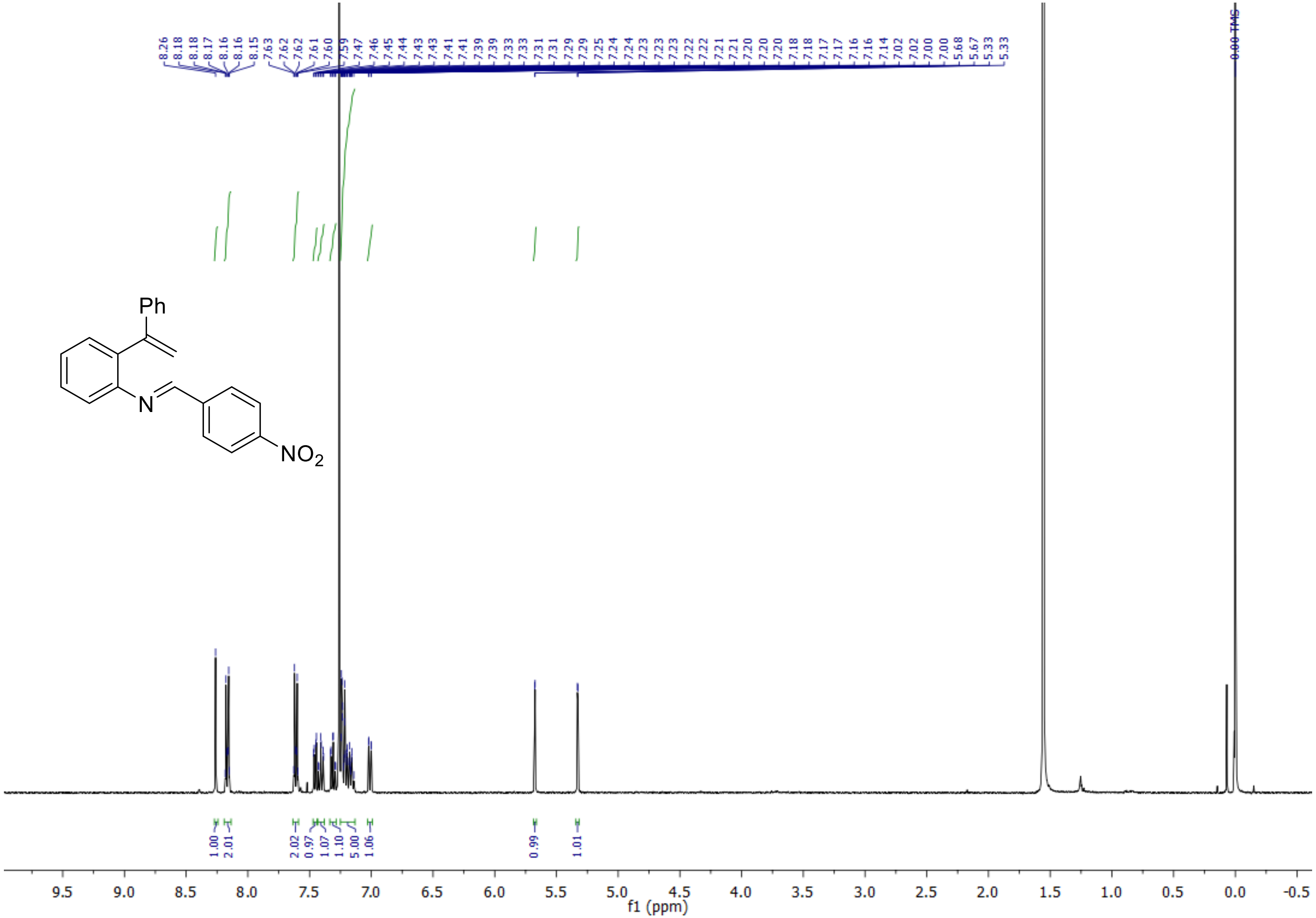

^{13}C NMR (101 MHz, $CDCl_3$) of **1v**

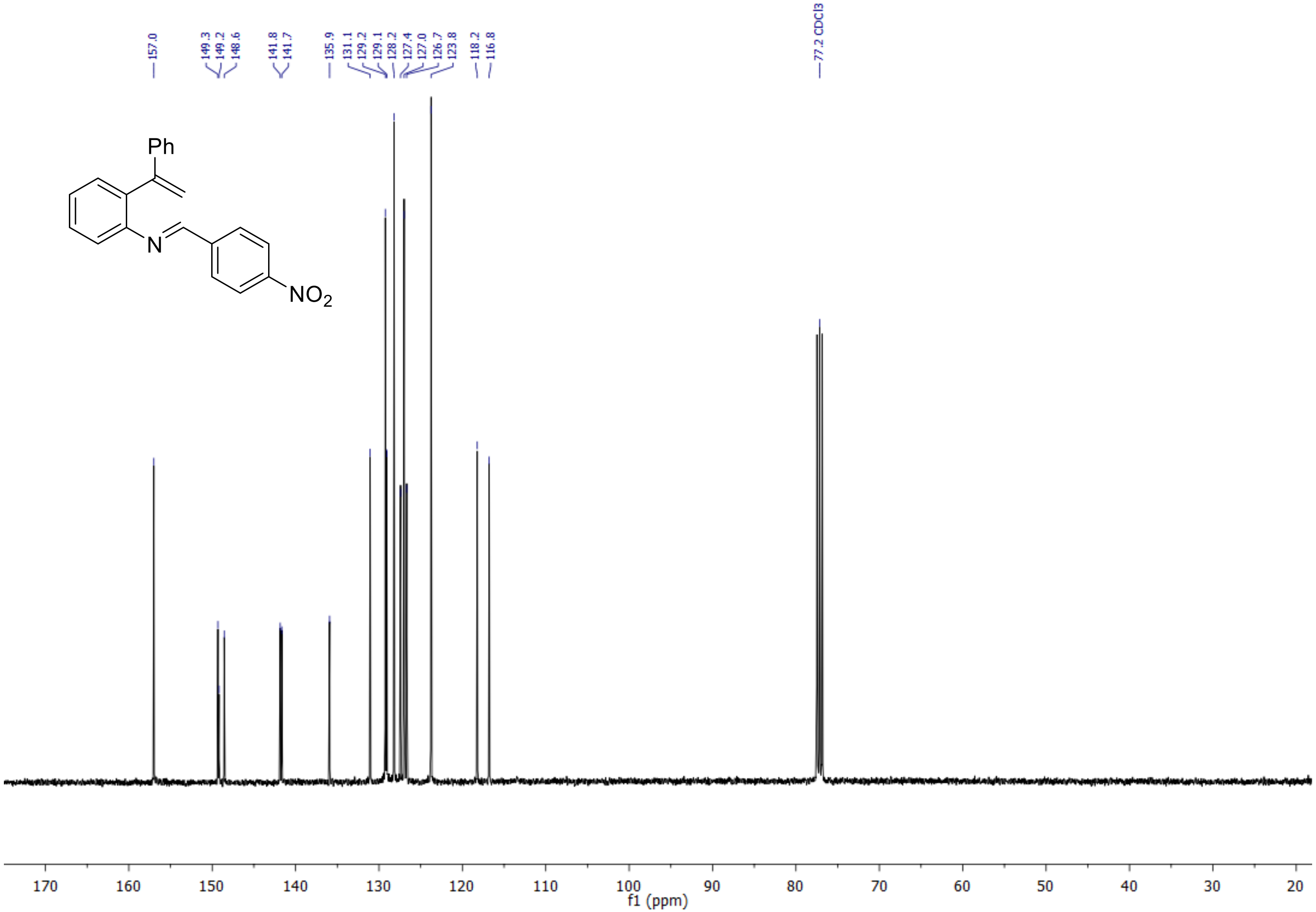

^{1}H–^{13}C HSQC-DEPT NMR (400 MHz, $CDCl_3$) of **1v**

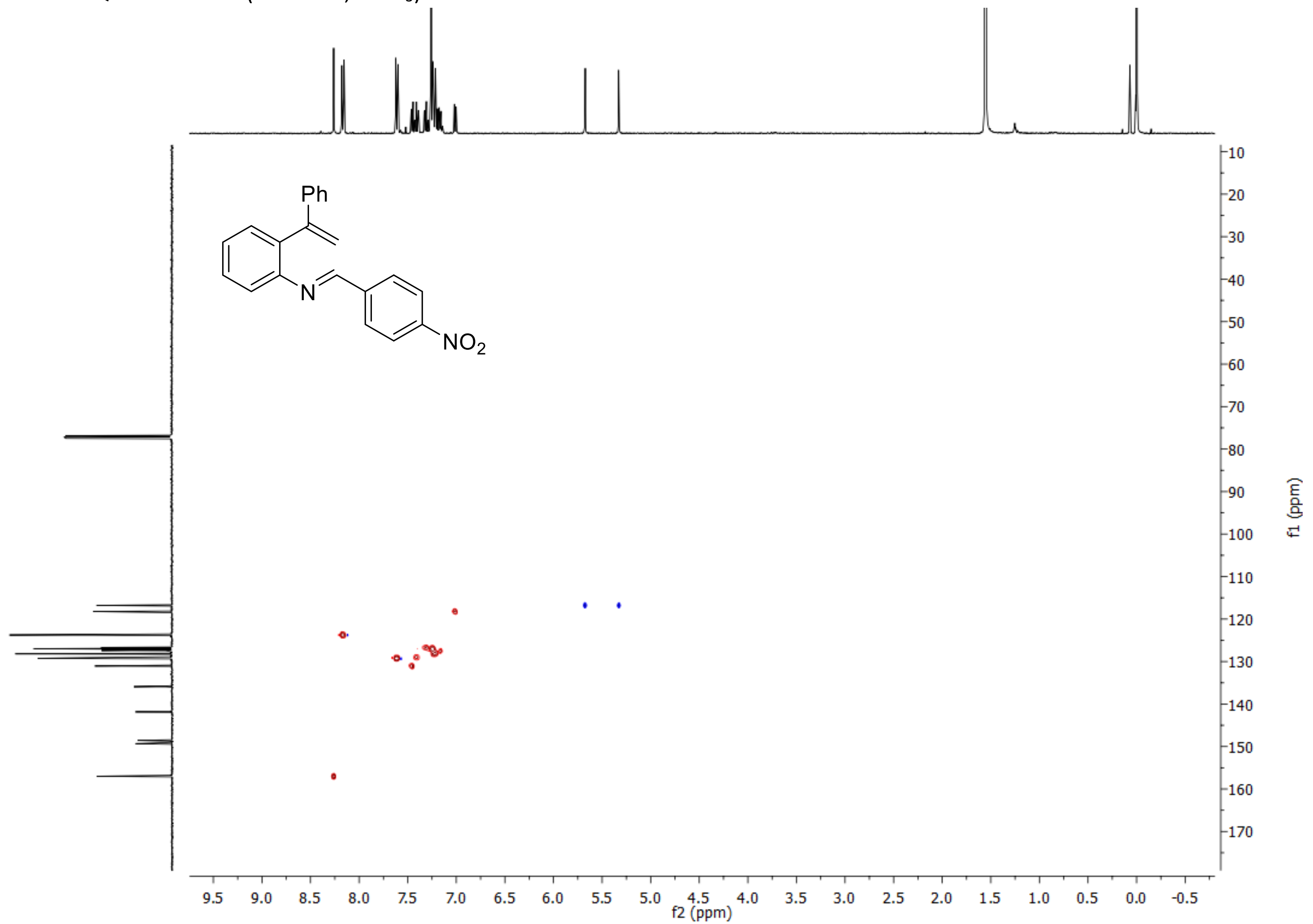

^{1}H NMR (400 MHz, $CDCl_3$) of **1w**

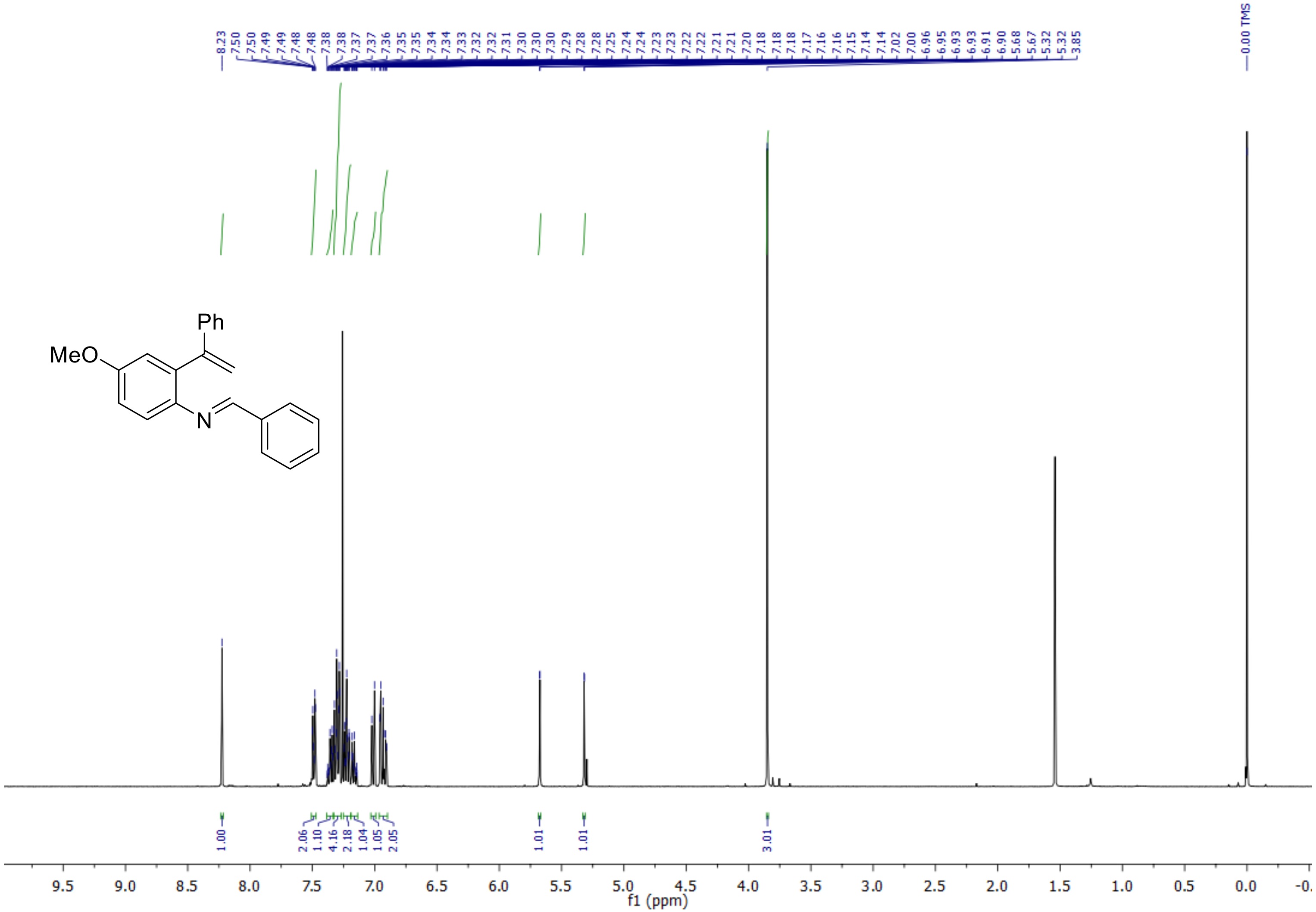

^{13}C NMR (101 MHz, $CDCl_3$) of **1w**

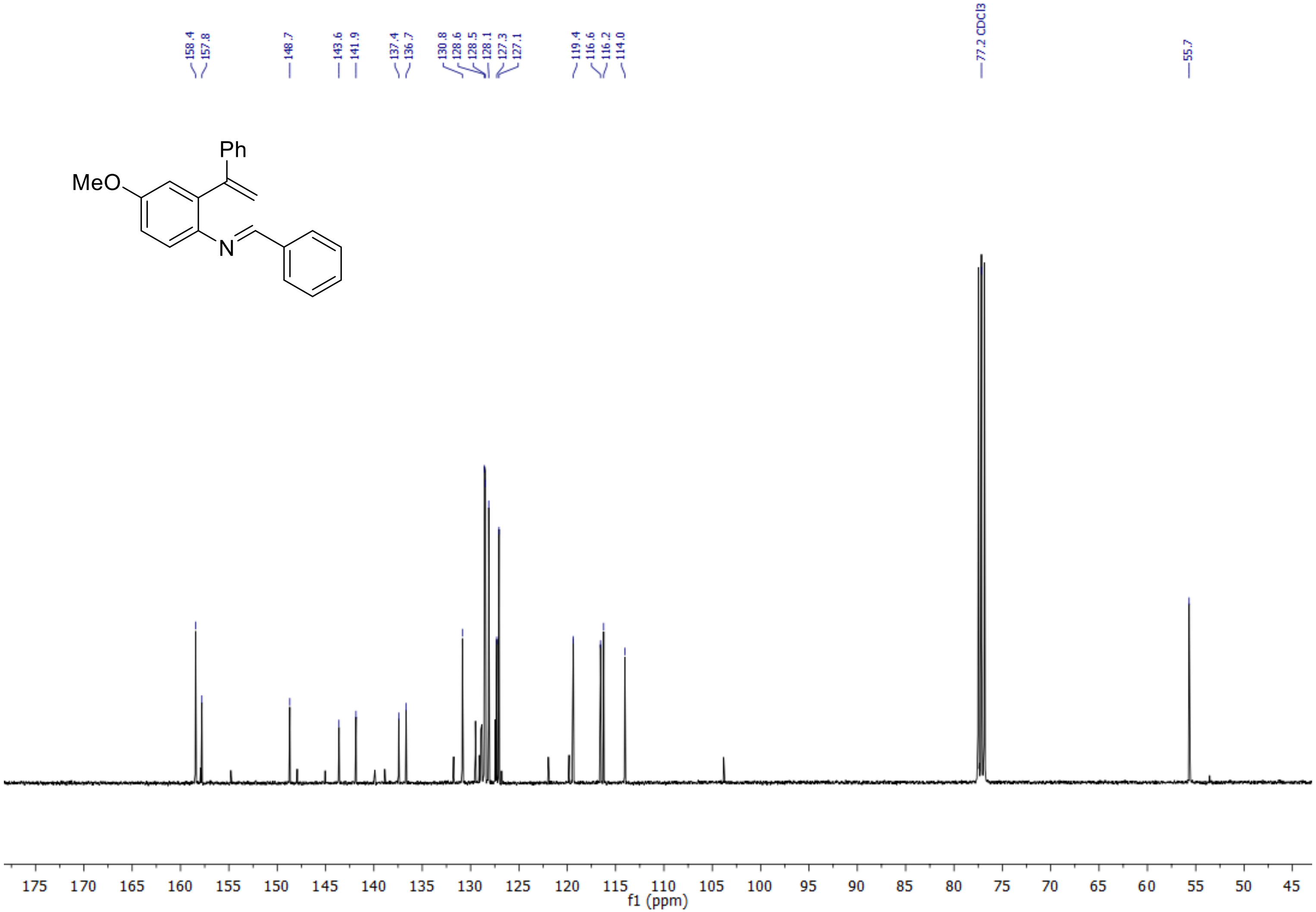

^{1}H–^{13}C HSQC-DEPT NMR (400 MHz, $CDCl_3$) of **1w**

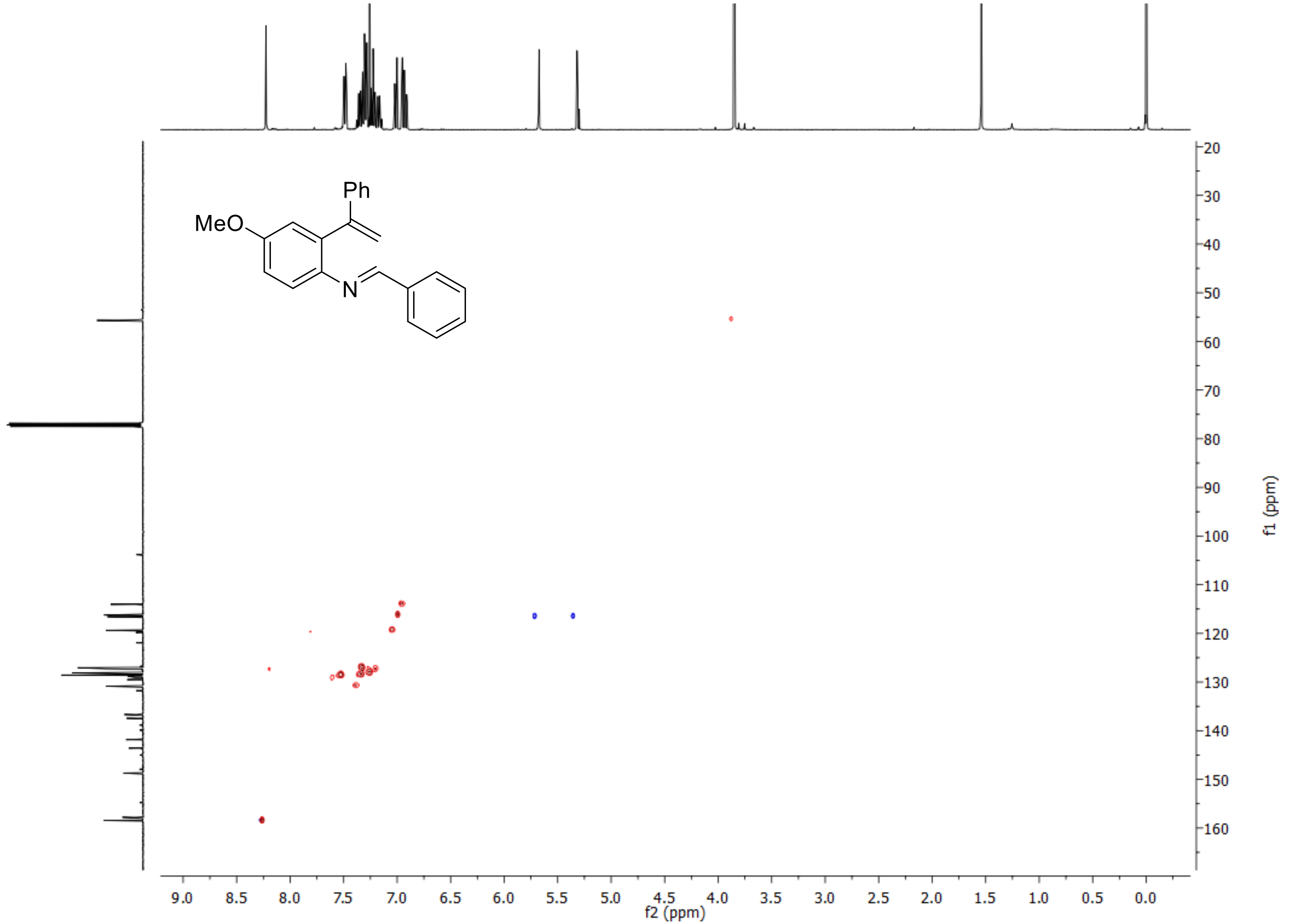

^{1}H NMR (400 MHz, $CDCl_3$) of **1x**

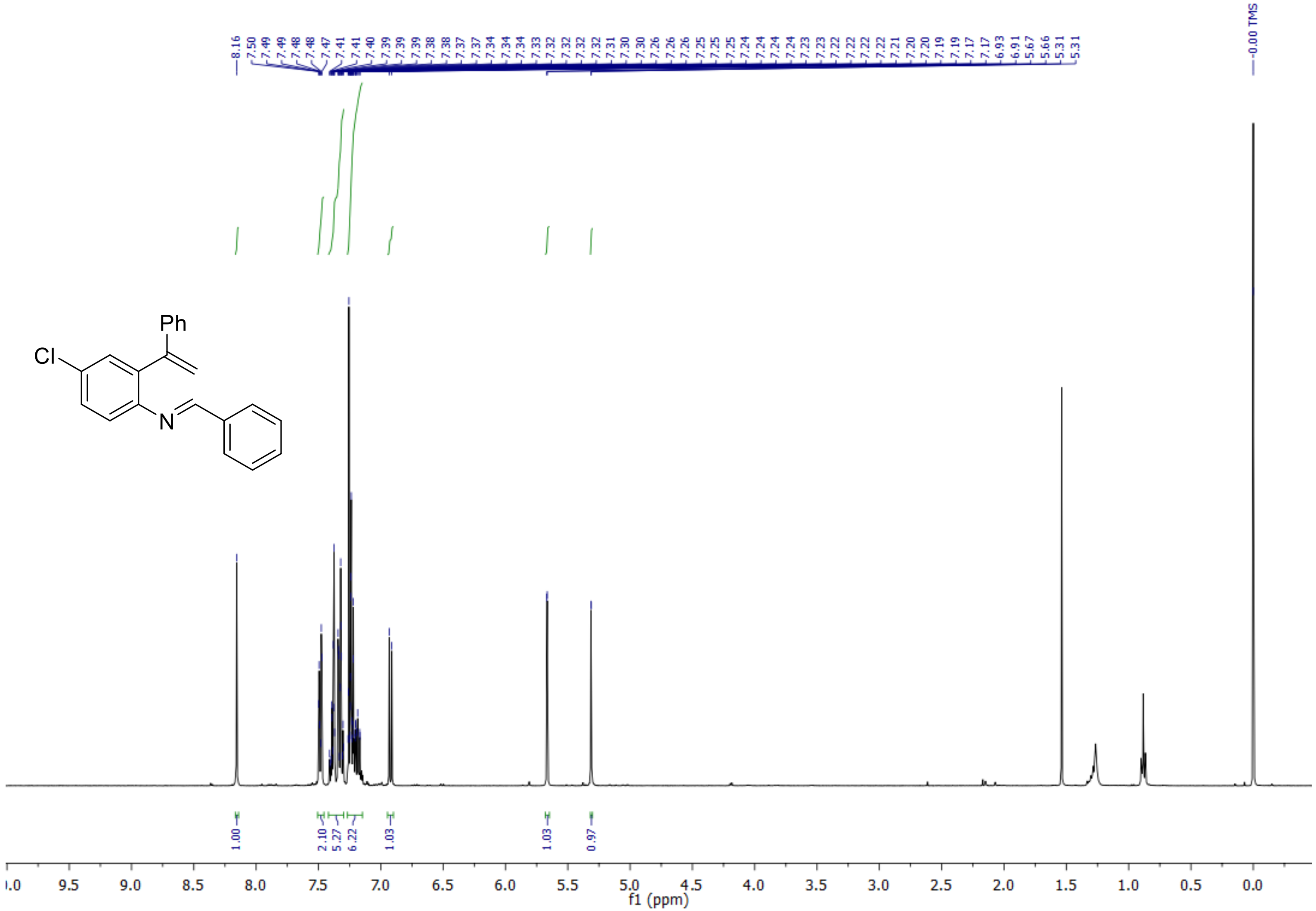

^{13}C NMR (101 MHz, acetone-d_6) of **1x**

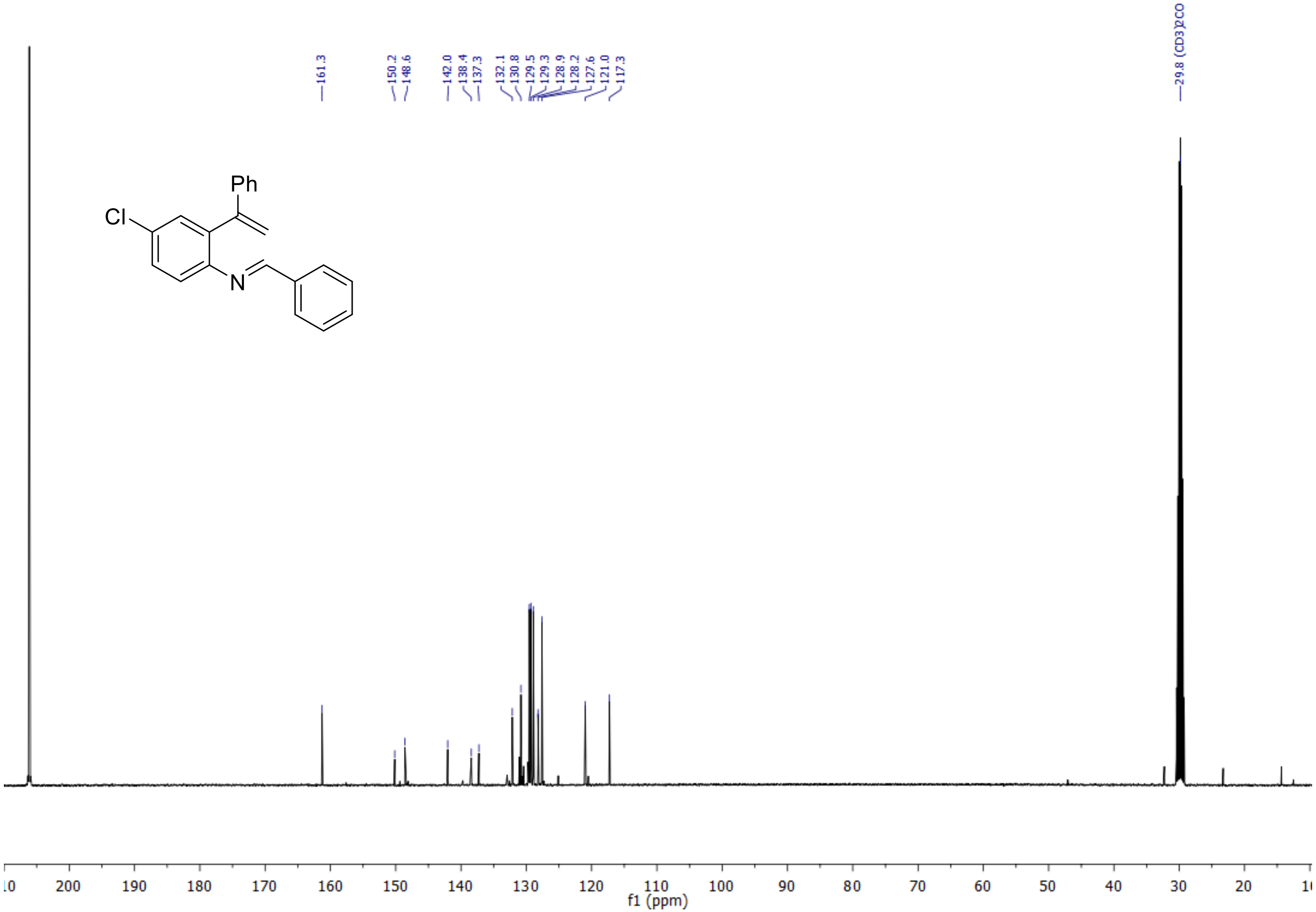

^{1}H–^{13}C HSQC-DEPT NMR (400 MHz, $CDCl_3$) of **1x**

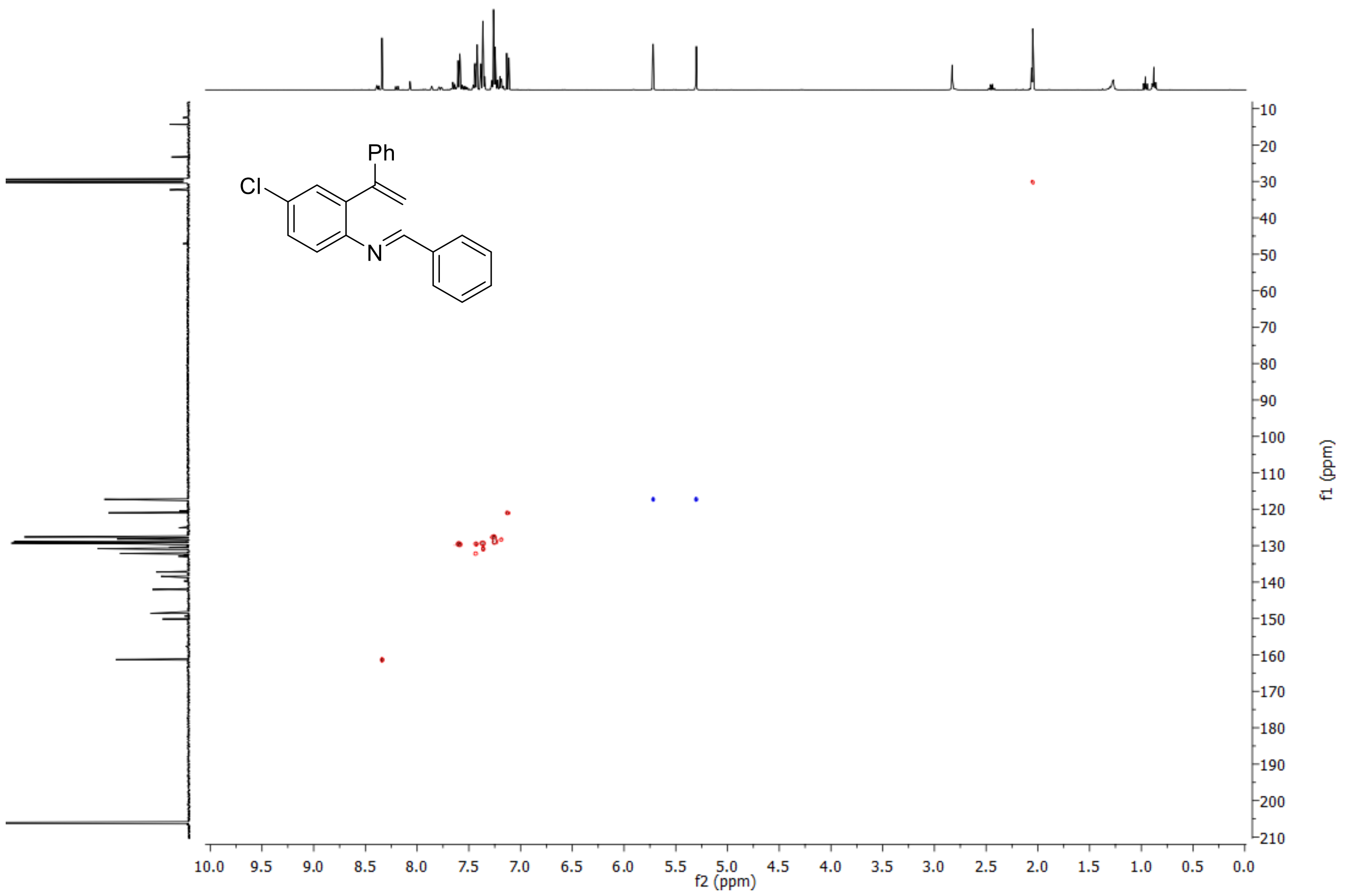

^{1}H NMR (400 MHz, $CDCl_3$) of **1y**

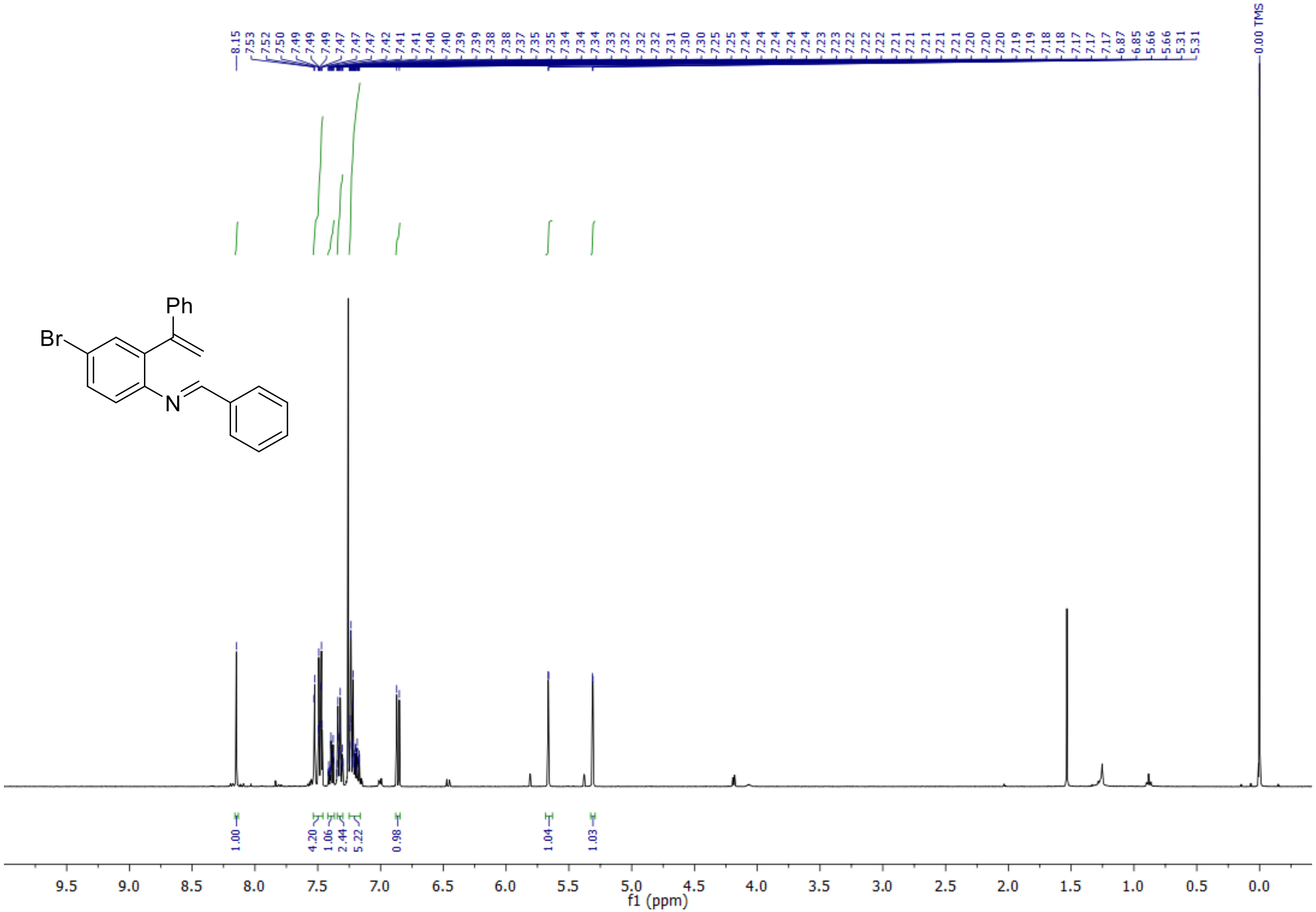

^{13}C NMR (101 MHz, $CDCl_3$) of **1y**

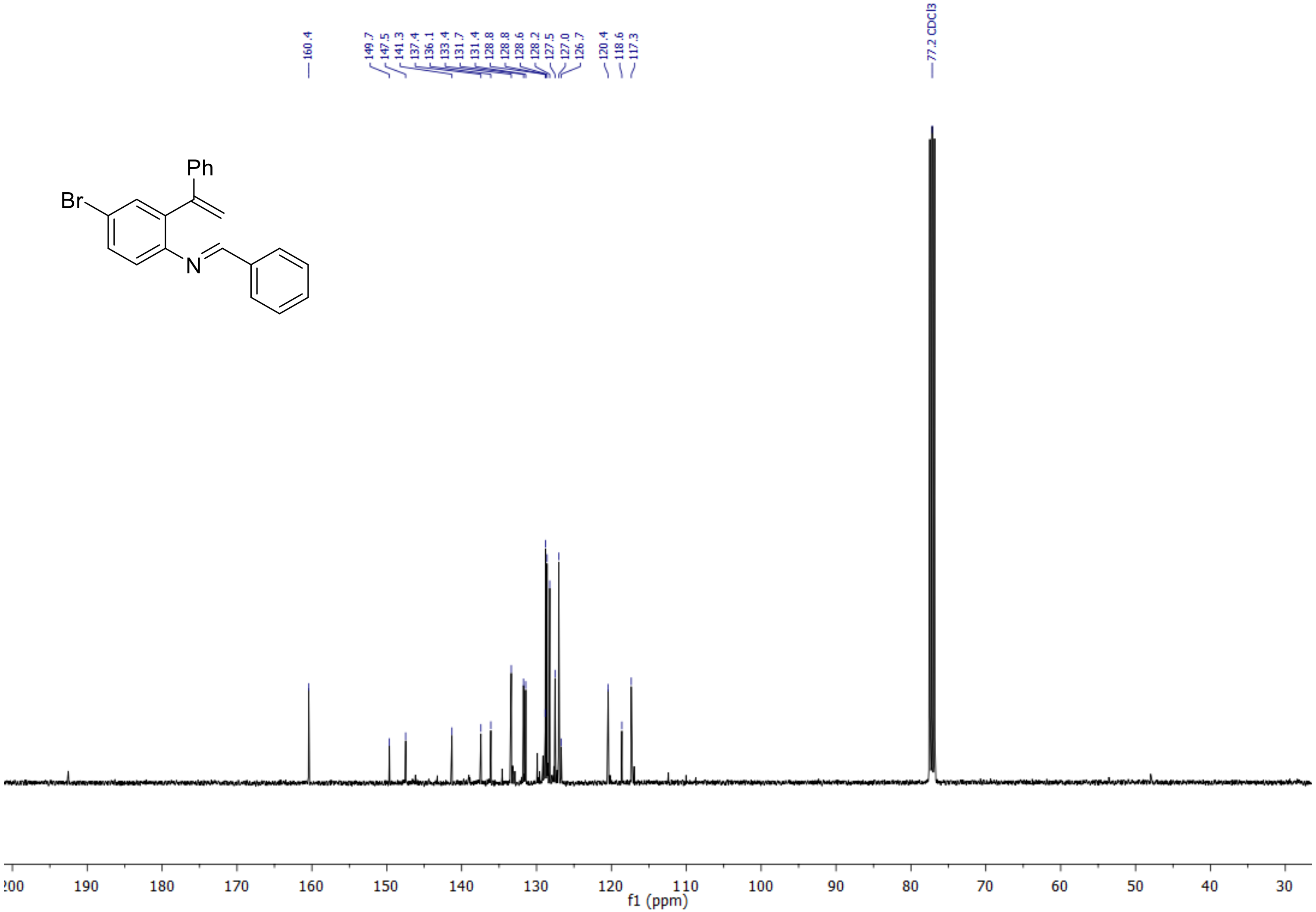

^{1}H–^{13}C HSQC-DEPT NMR (400 MHz, $CDCl_3$) of **1y**

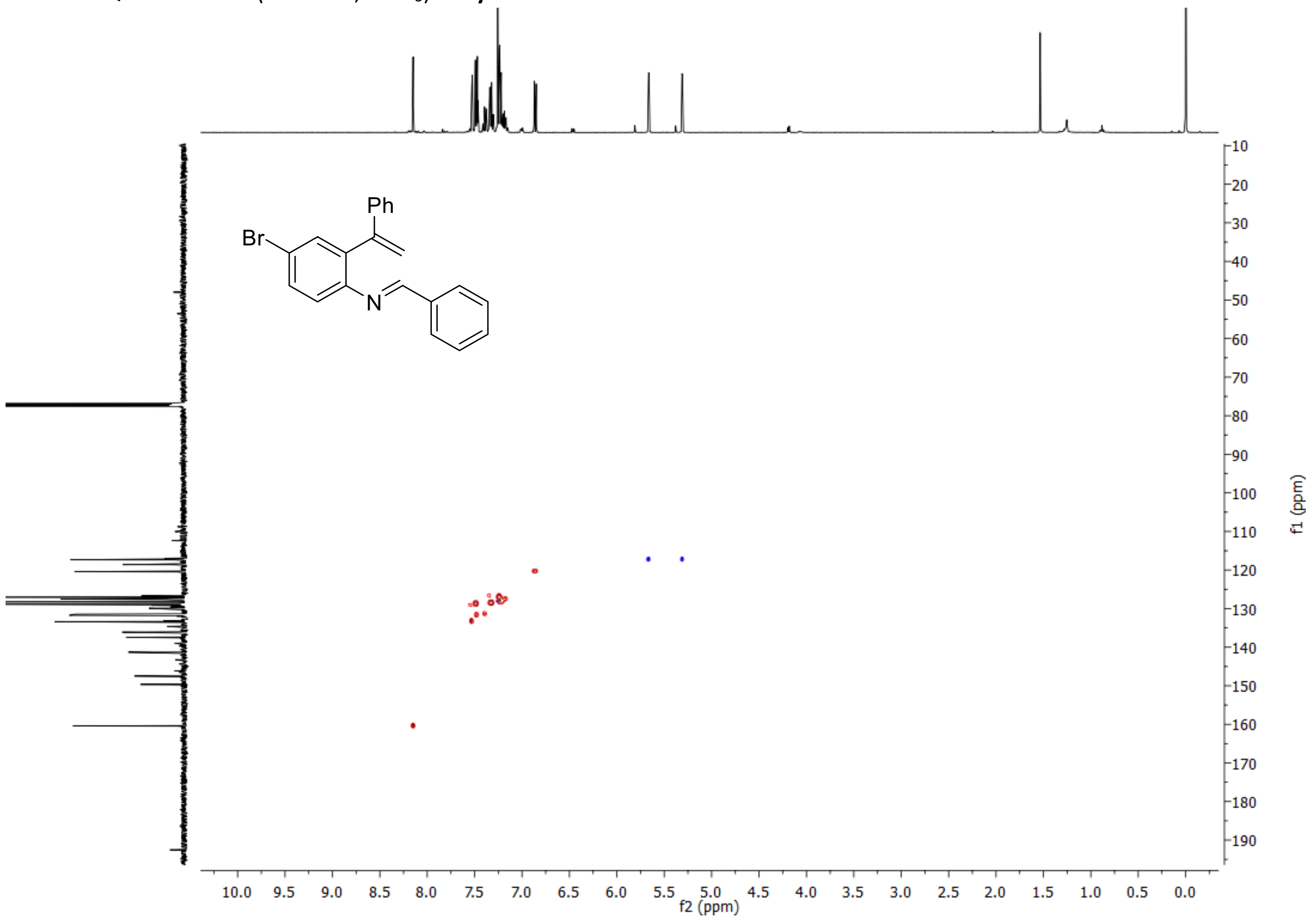

^{1}H NMR (400 MHz, $CDCl_3$) of **1z**

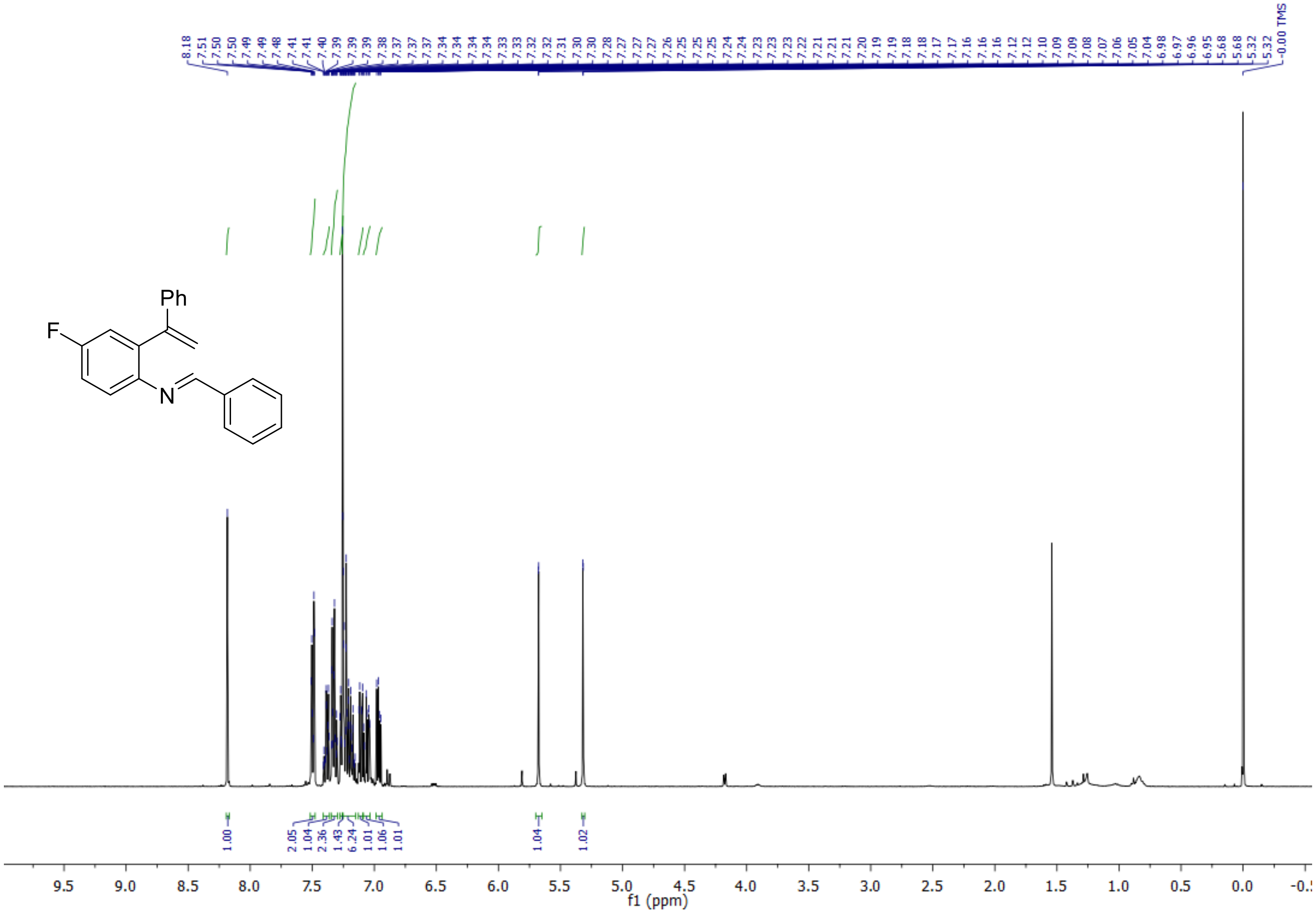

^{13}C NMR (101 MHz, acetone-d_6) of **1z**

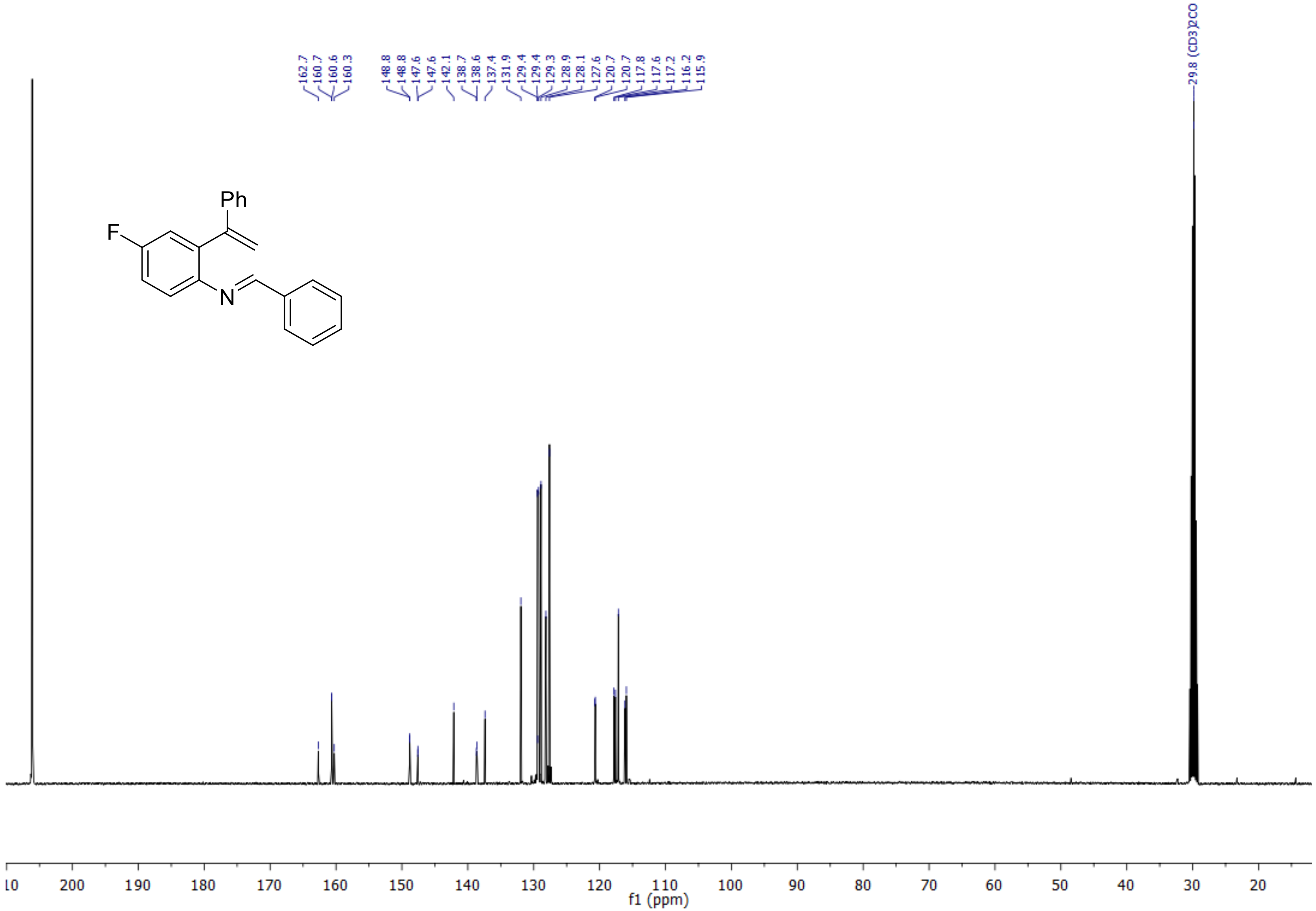

^{19}F NMR (377 MHz, acetone-d_6) of **1z**

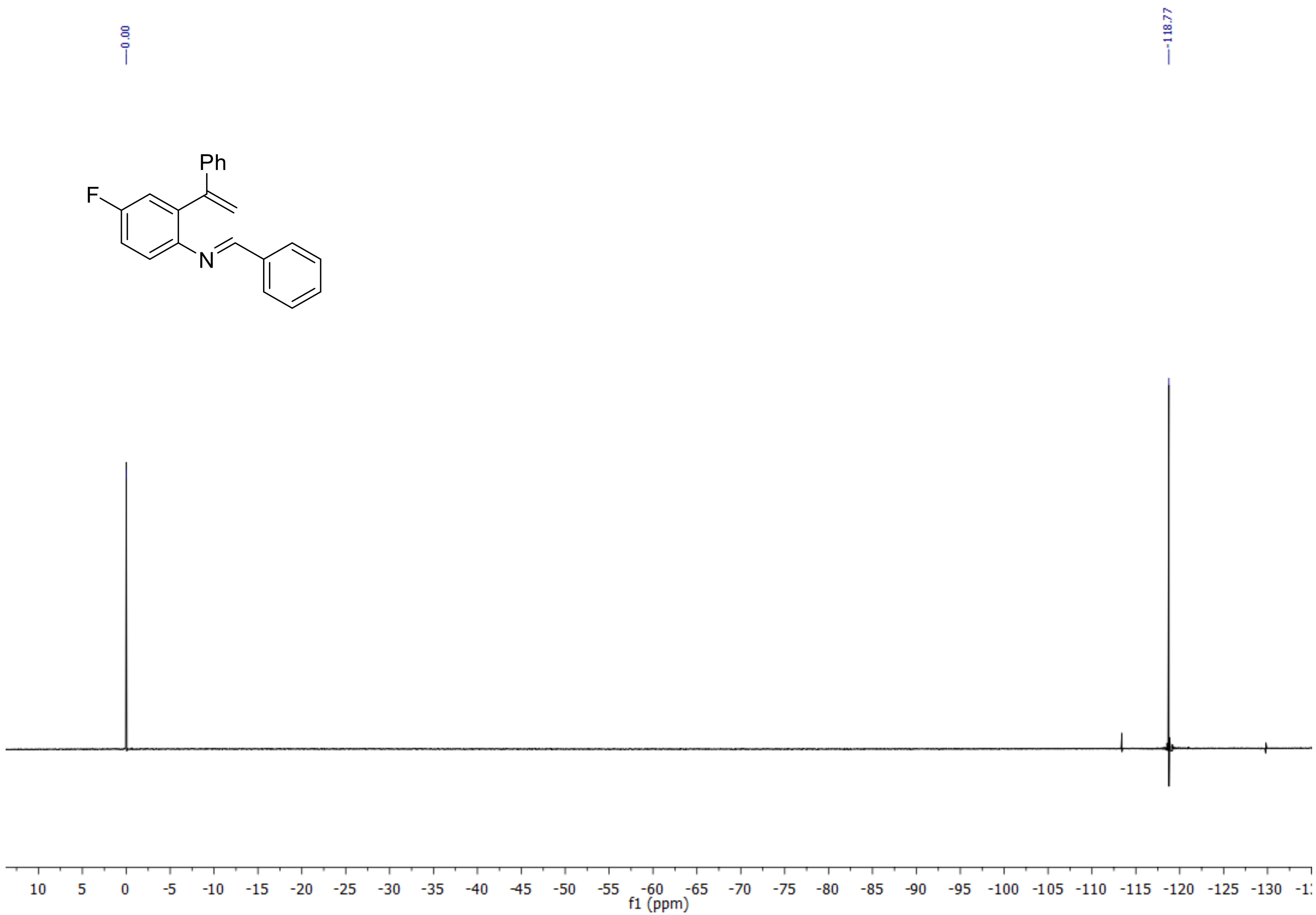

^{1}H–^{13}C HSQC-DEPT NMR (400 MHz, acetone-d_6) of **1z**

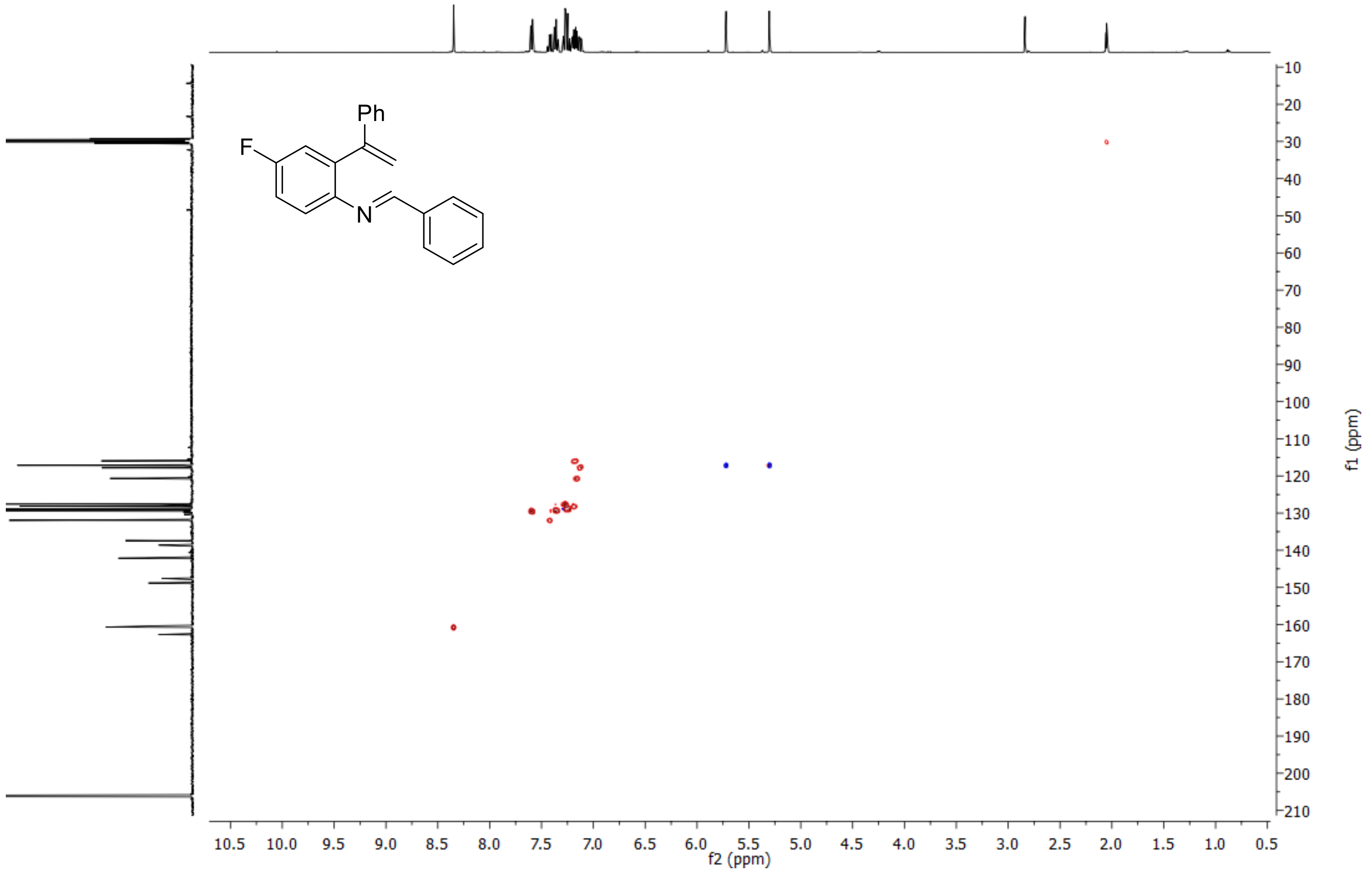

^{1}H NMR (400 MHz, $CDCl_3$) of **1ab**

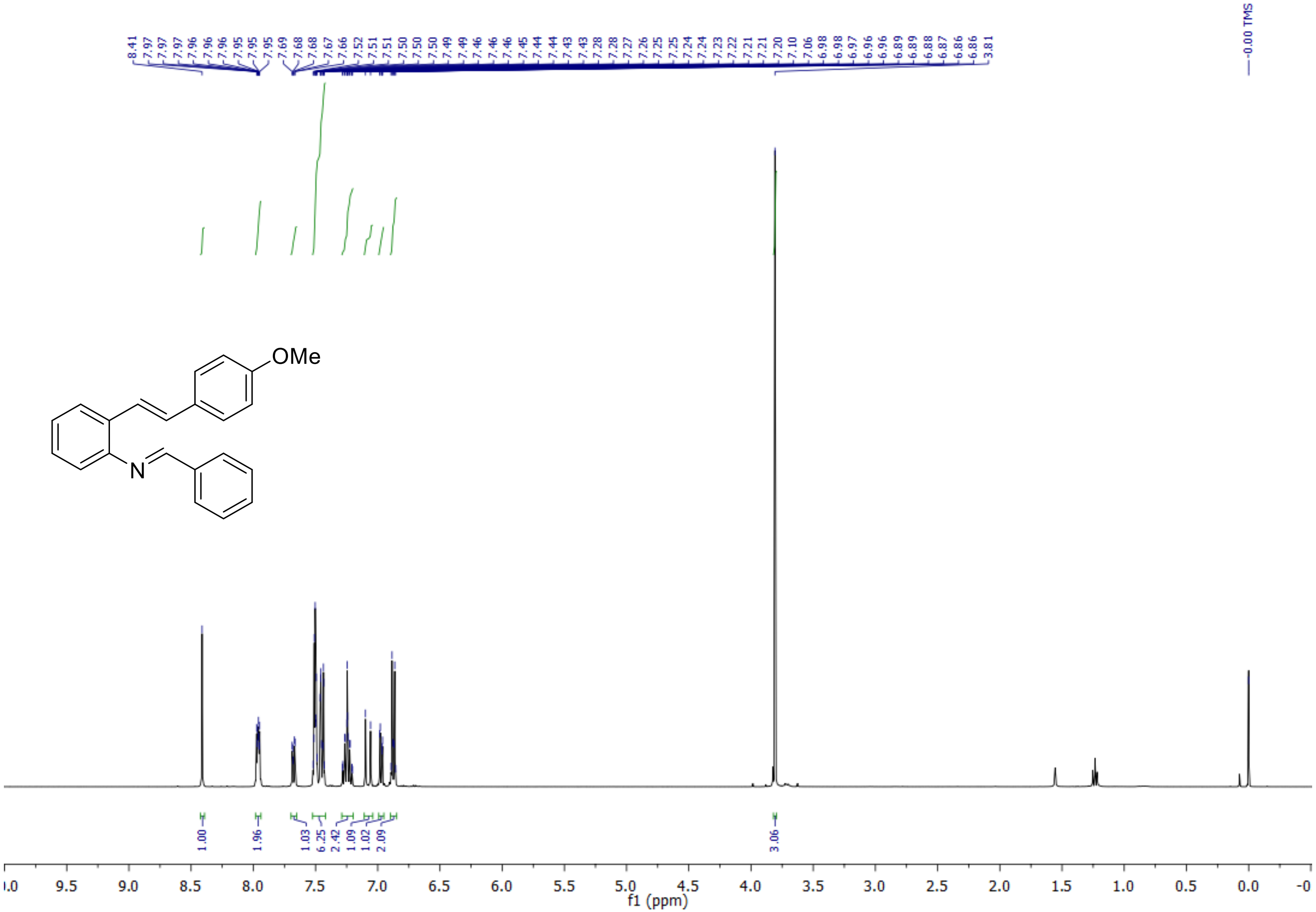

^{13}C NMR (101 MHz, $CDCl_3$) of **1ab**

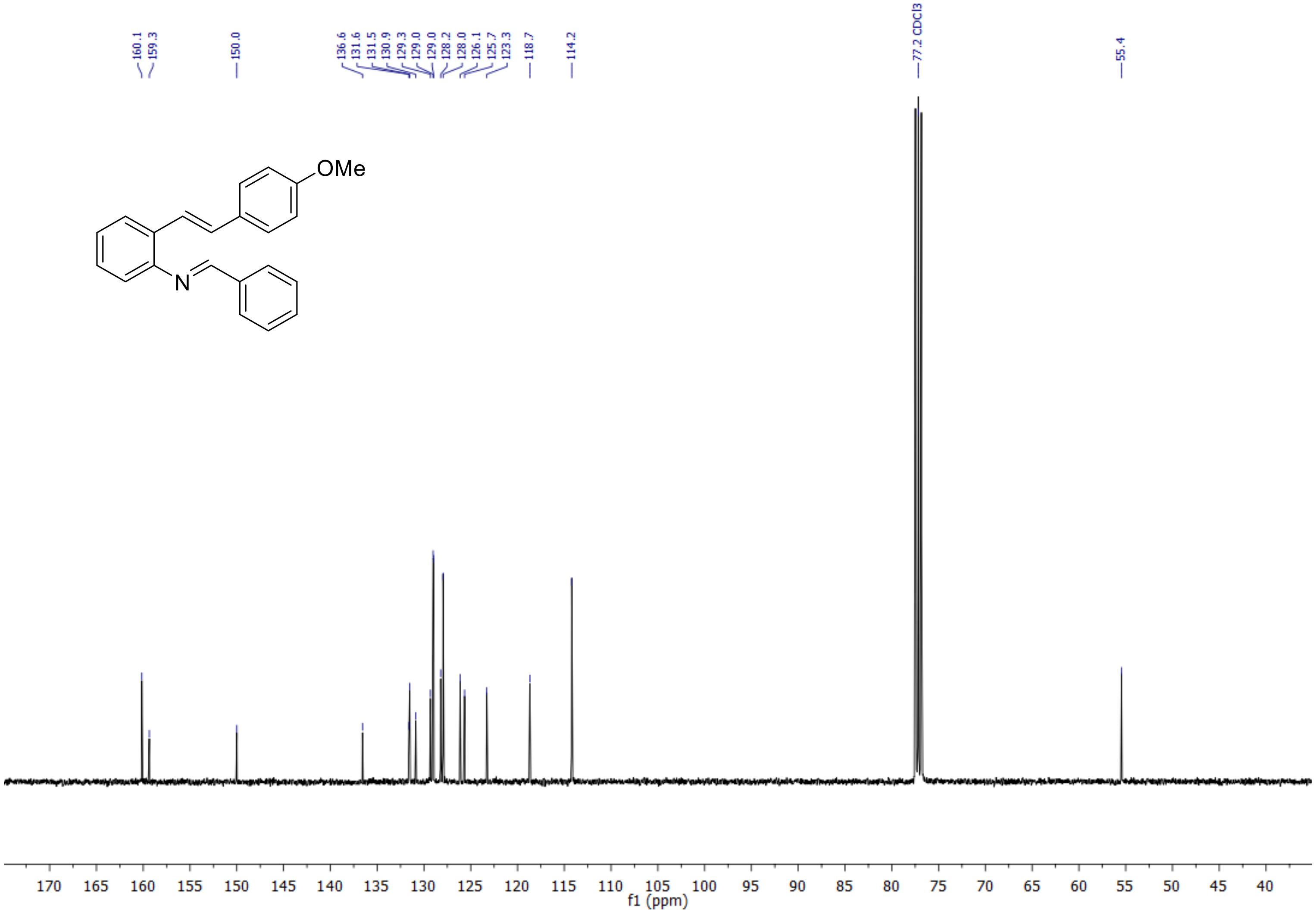

^{1}H–^{13}C HSQC-DEPT NMR (400 MHz, $CDCl_3$) of **1ab**

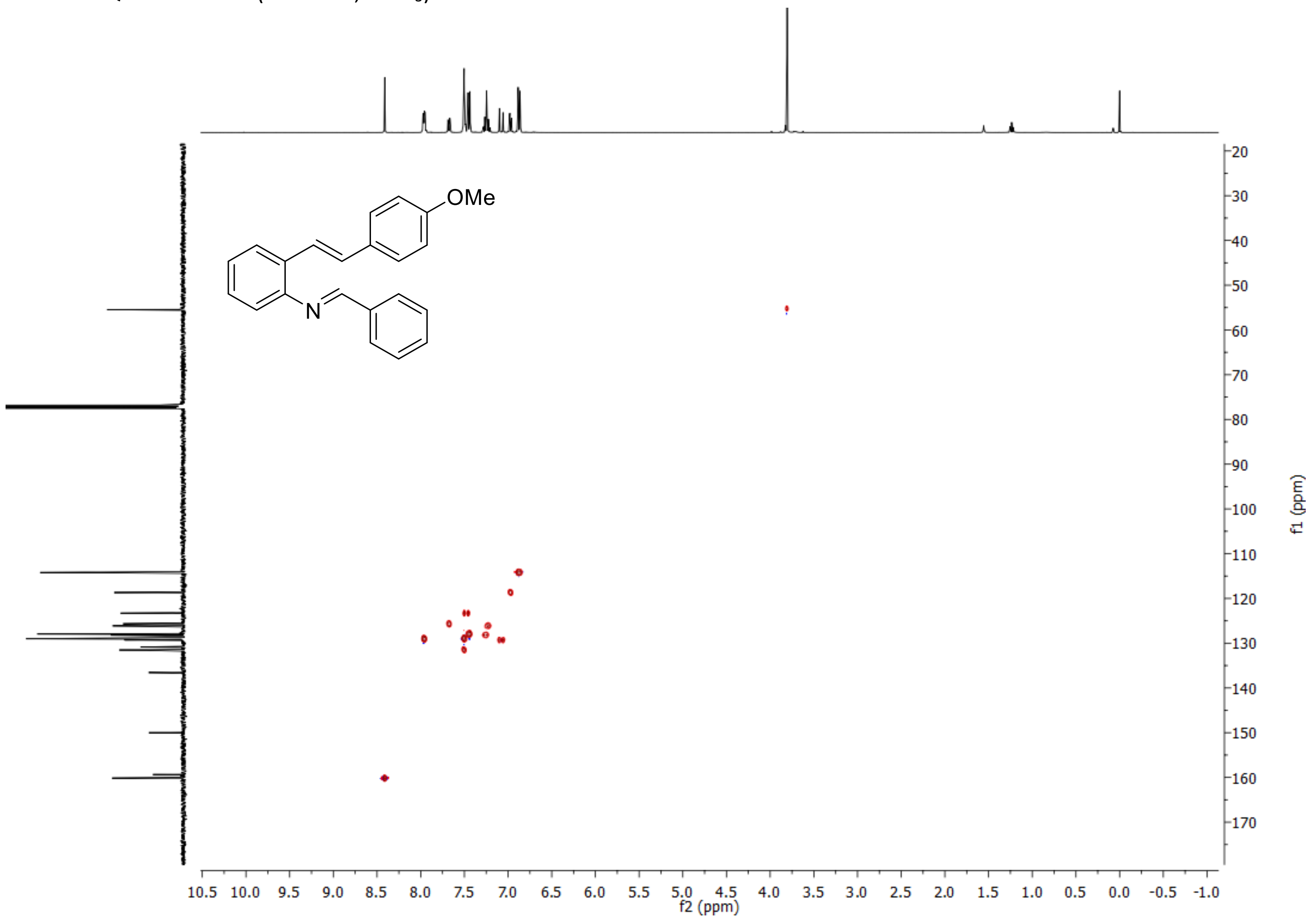

^{1}H NMR (400 MHz, $CDCl_3$) of **1ac**

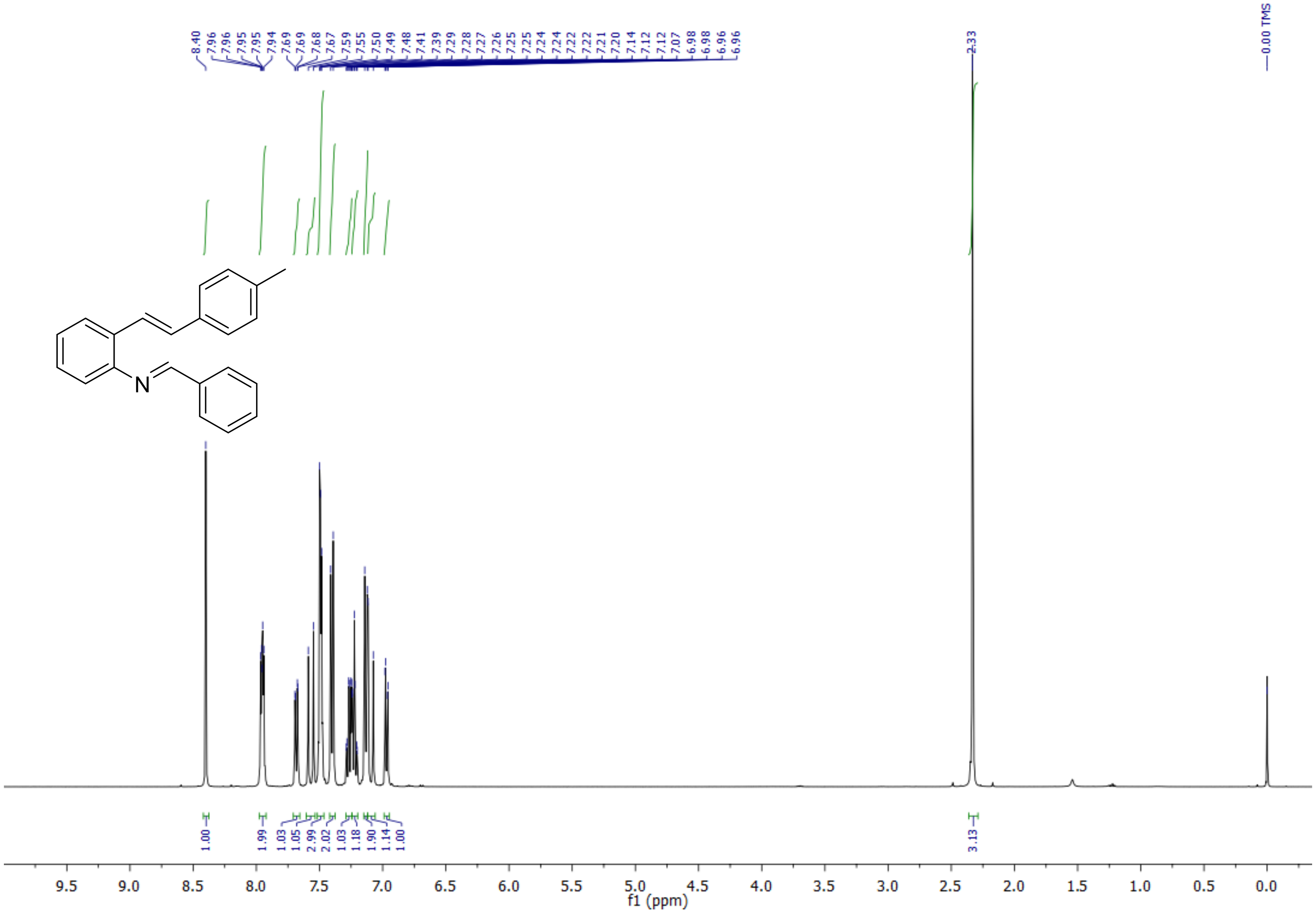

^{13}C NMR (101 MHz, $CDCl_3$) of **1ac**

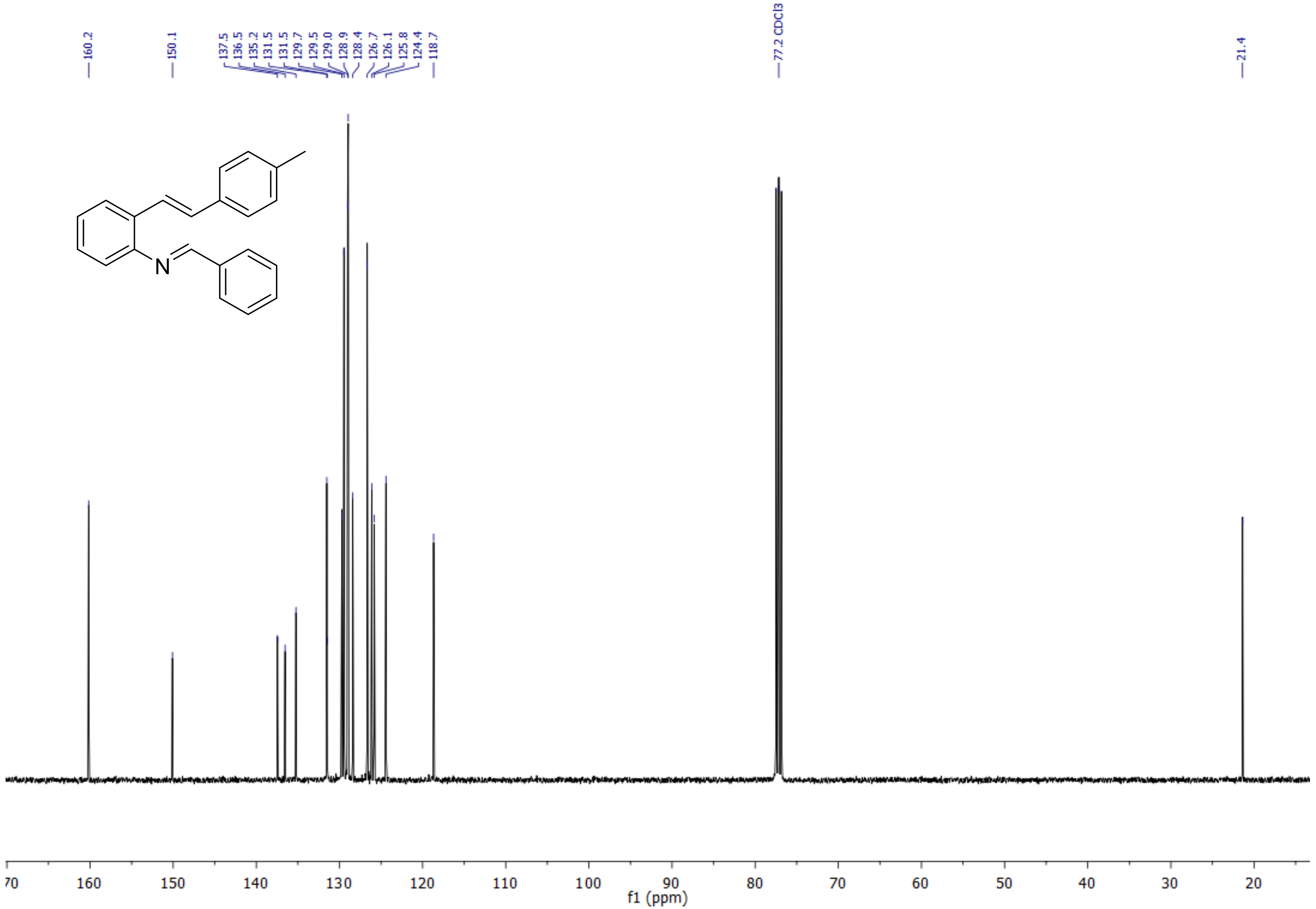

^{1}H–^{13}C HSQC-DEPT NMR (400 MHz, $CDCl_3$) of **1ac**

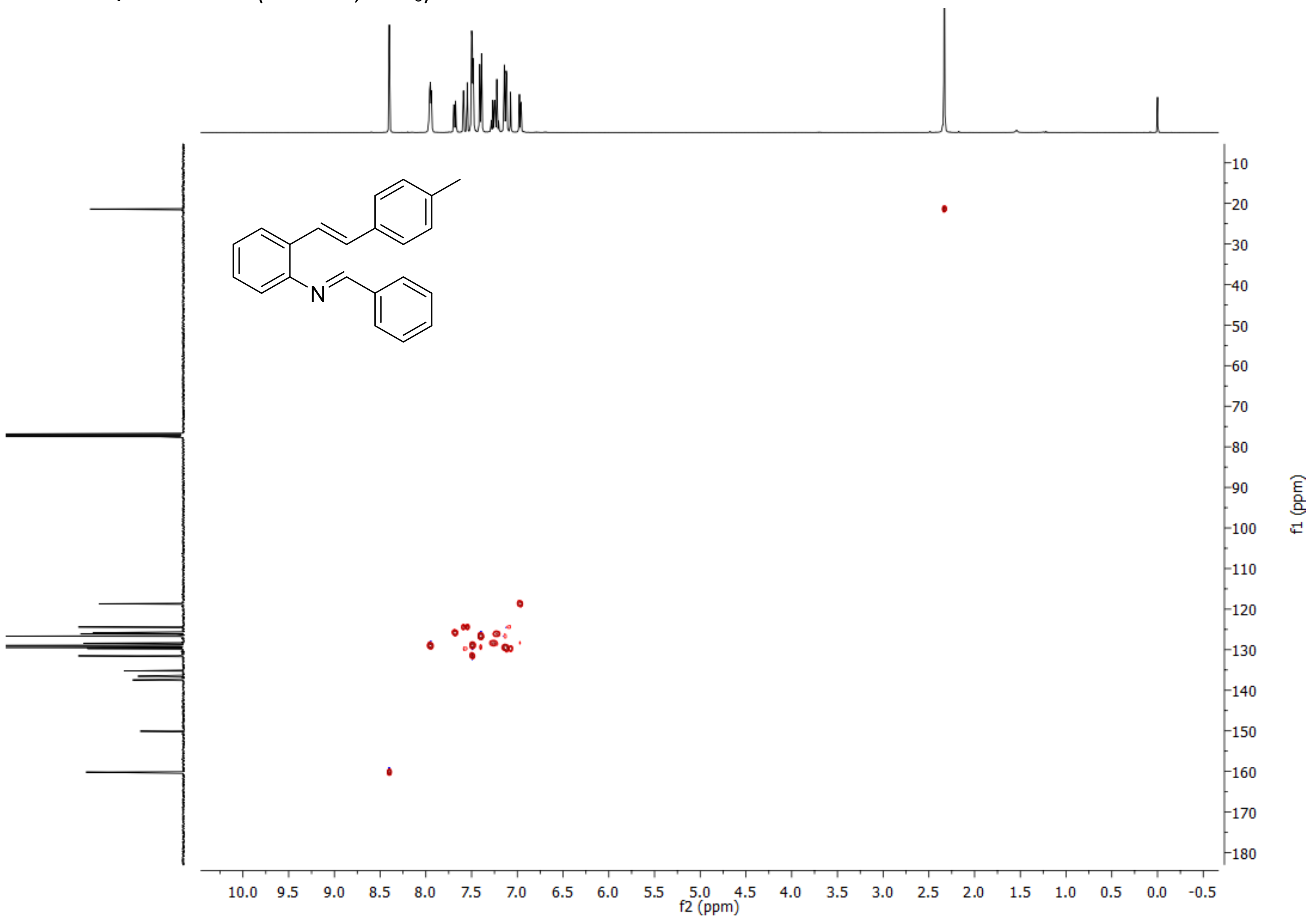

^{1}H NMR (400 MHz, $CDCl_3$) of **1ae**

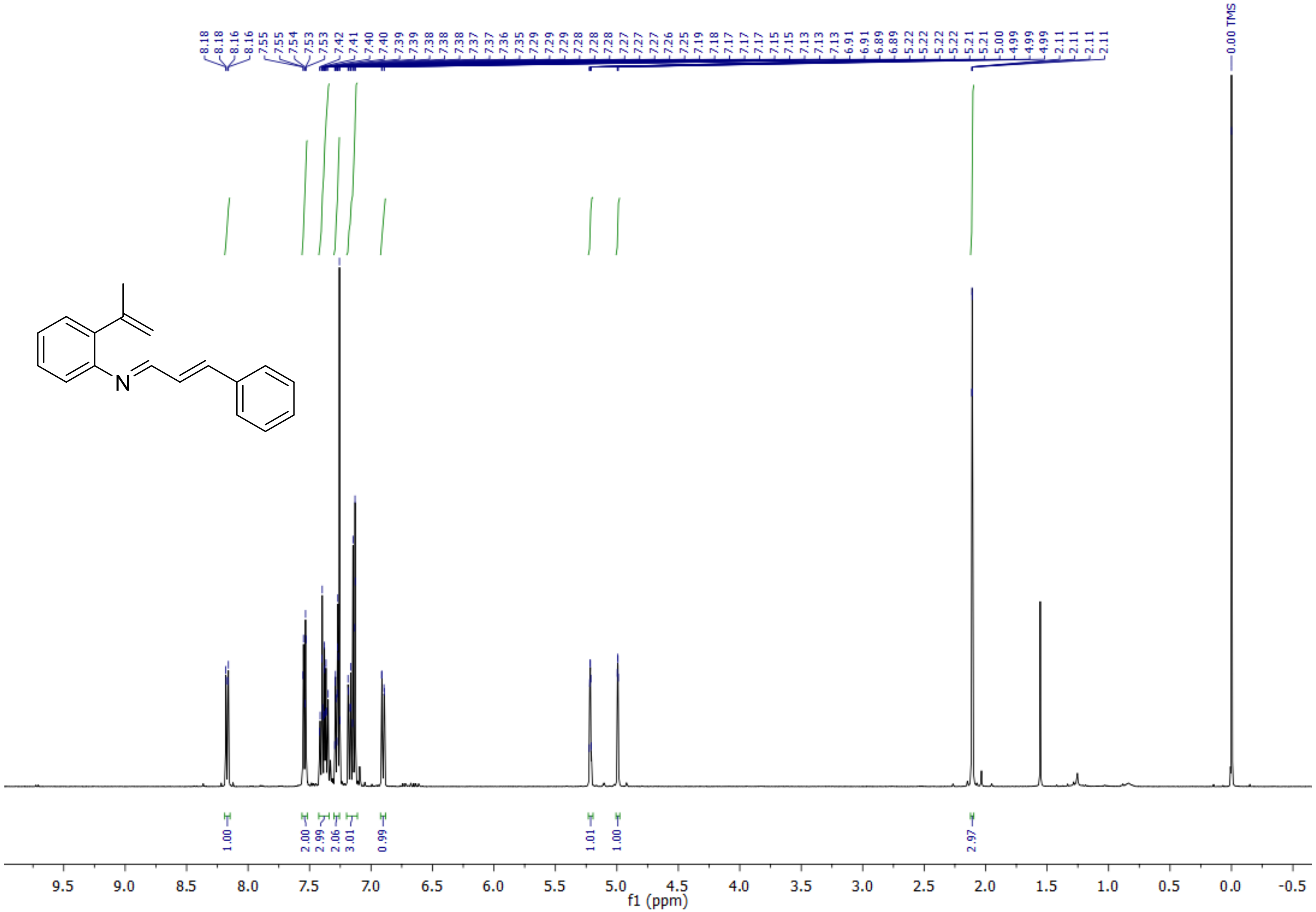

^{13}C NMR (101 MHz, $CDCl_3$) of **1ae**

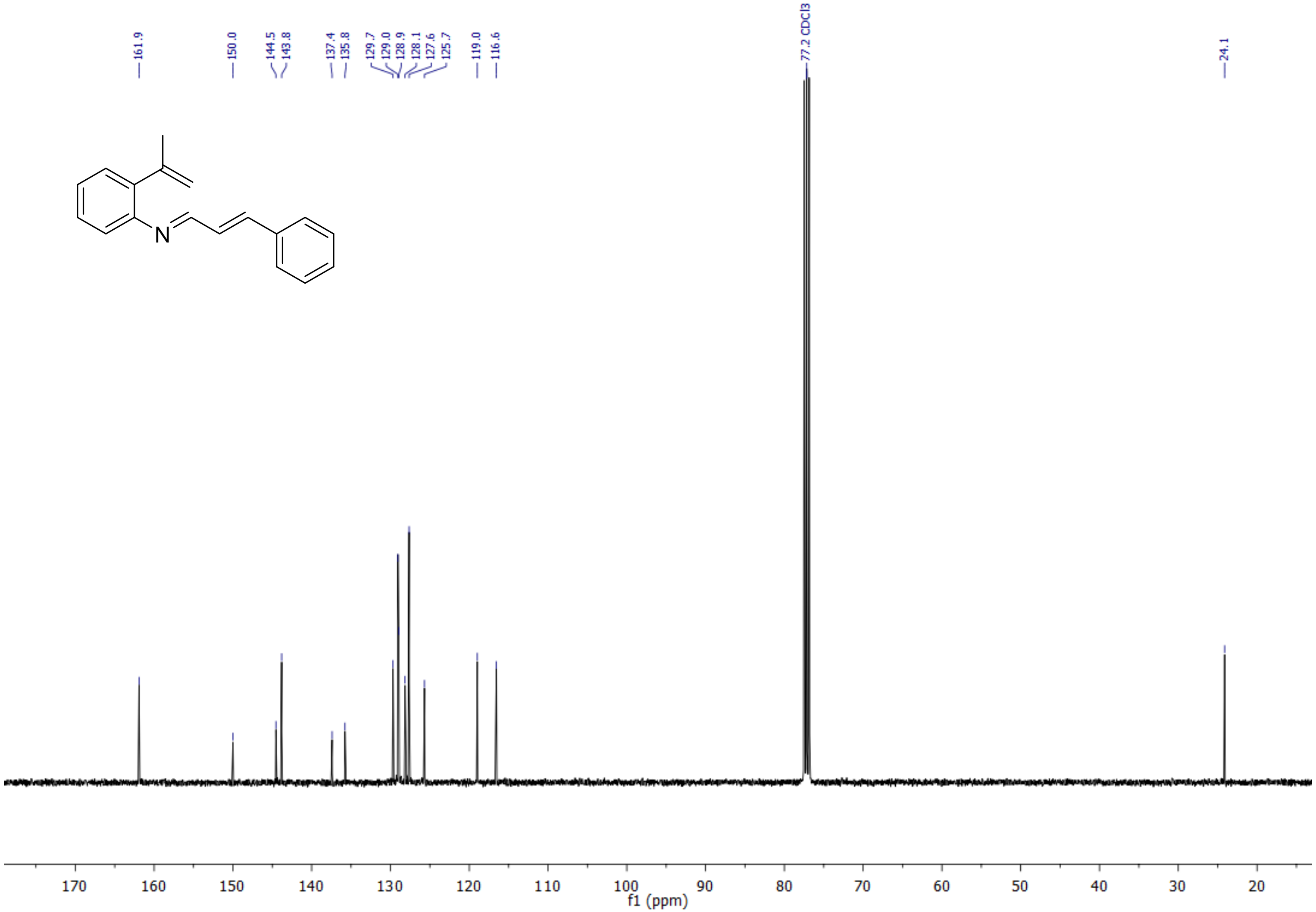

^{1}H–^{13}C HSQC-DEPT NMR (400 MHz, $CDCl_3$) of **1ae**

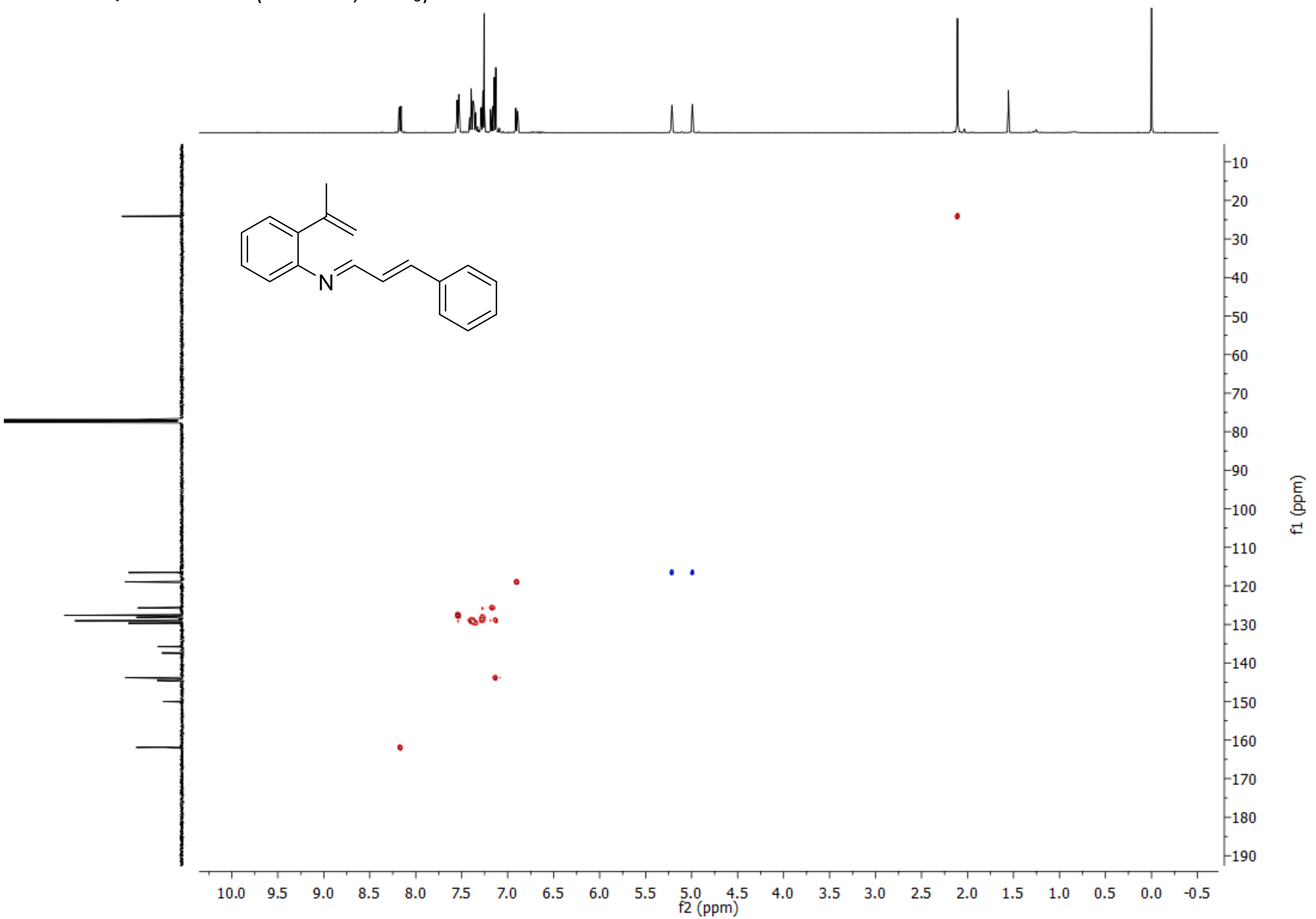

^{1}H NMR (400 MHz, $CDCl_3$) of **1af**

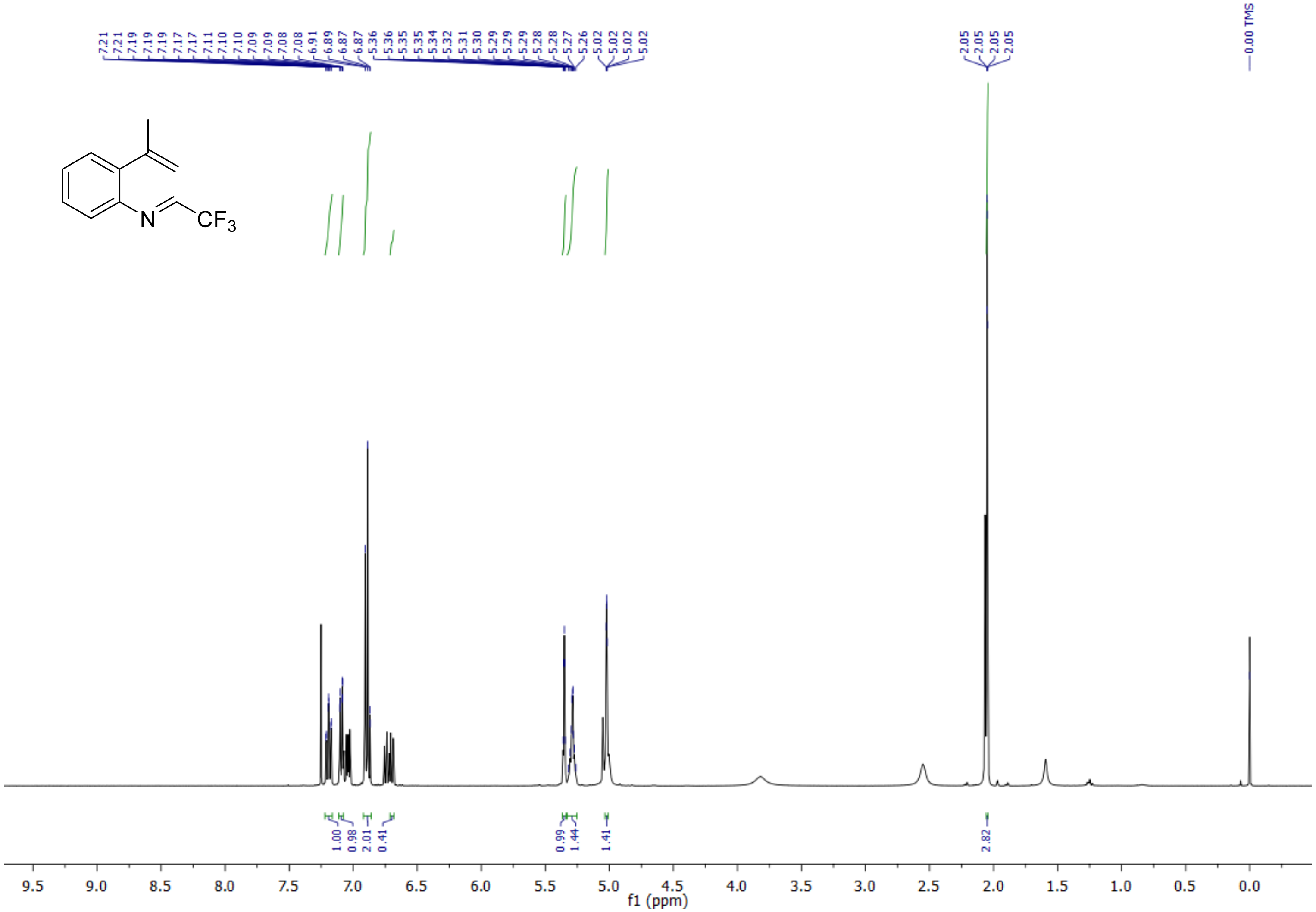

^{13}C NMR (101 MHz, $CDCl_3$) of **1af**

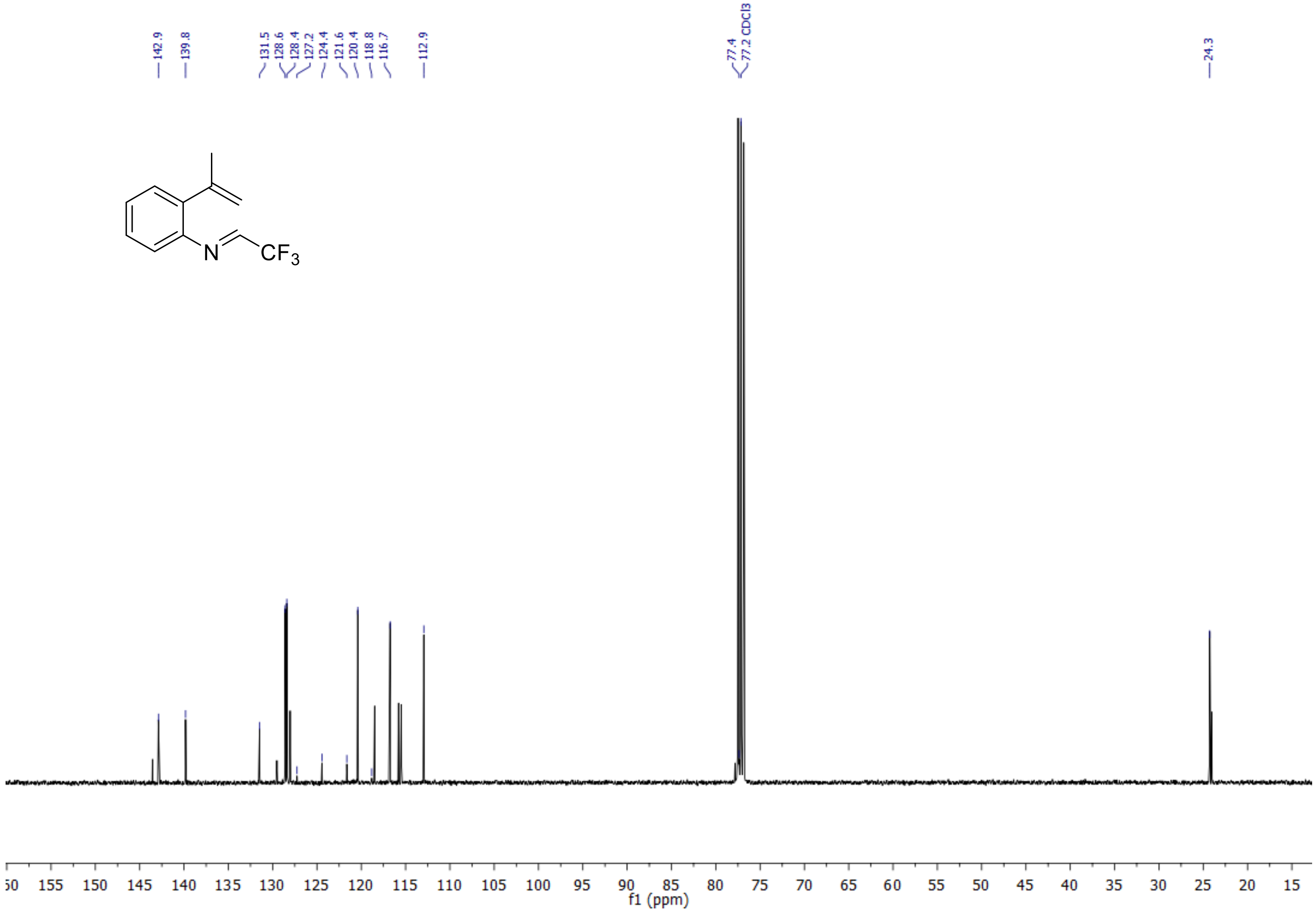

^{19}F NMR (377 MHz, $CDCl_3$) of **1af**

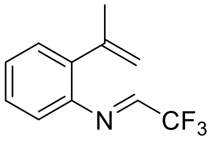

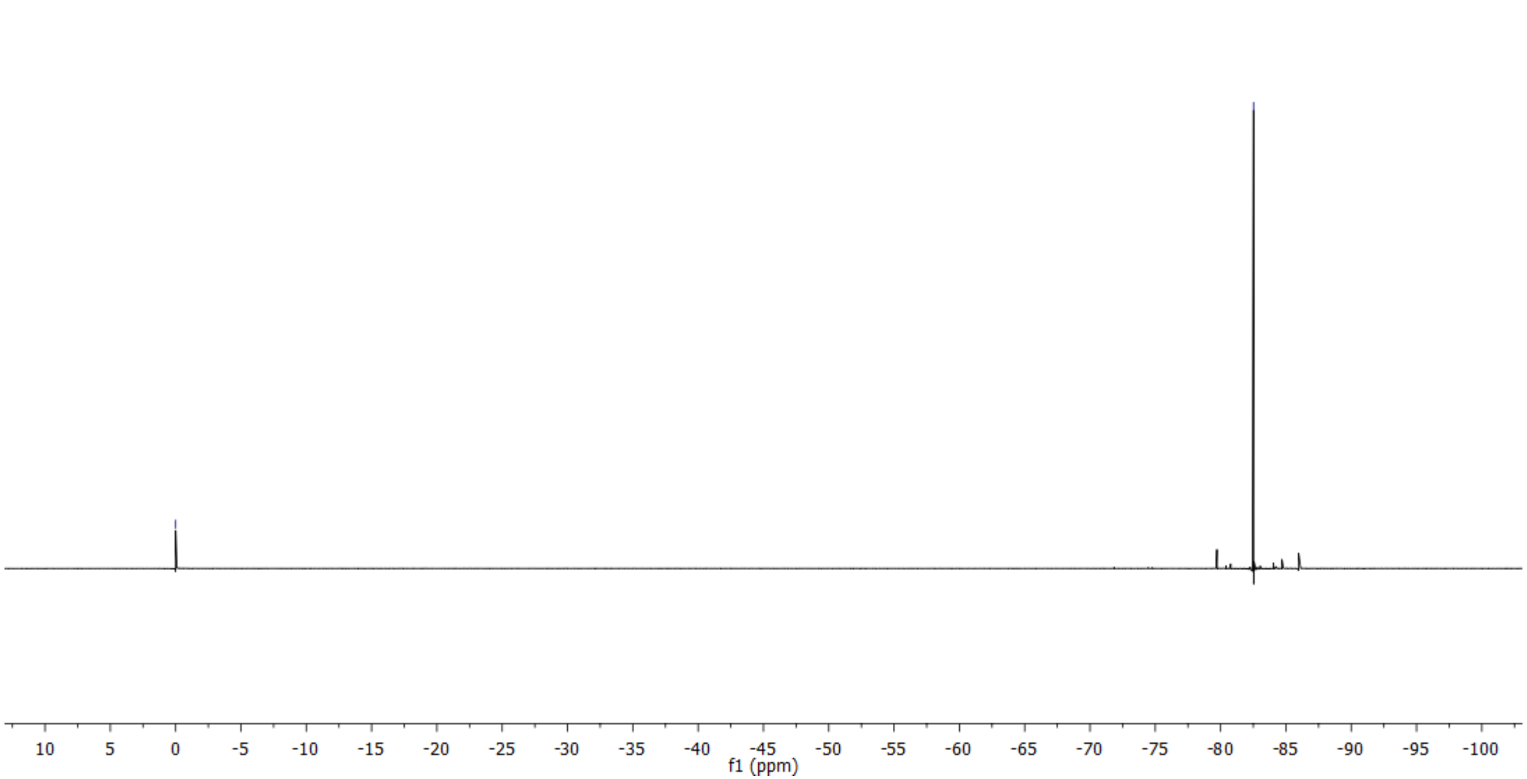

$^{1}H–^{13}C$ HSQC-DEPT NMR (400 MHz, $CDCl_3$) of **1af**

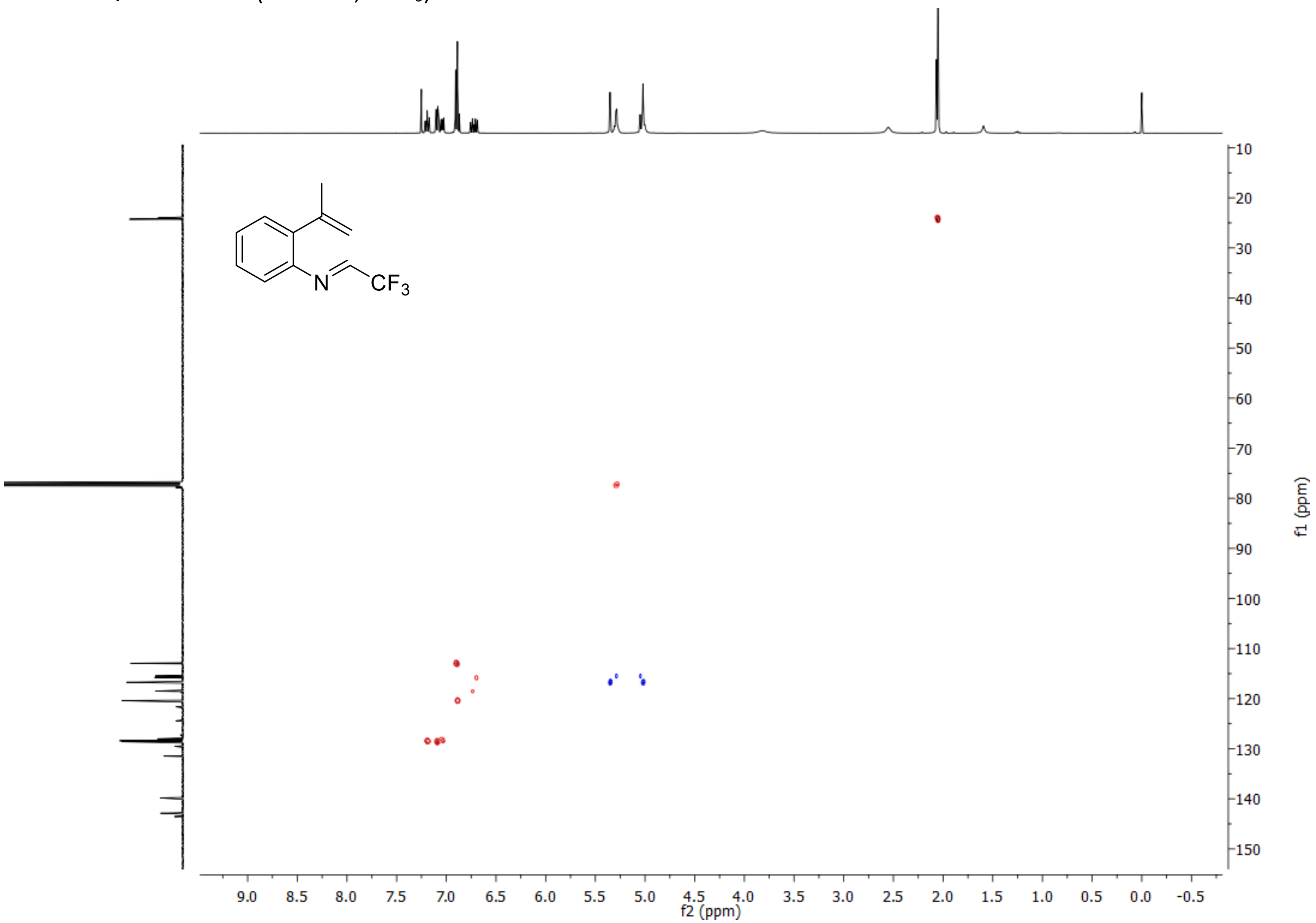

^{1}H NMR (400 MHz, acetone-d_6) of **1ag**

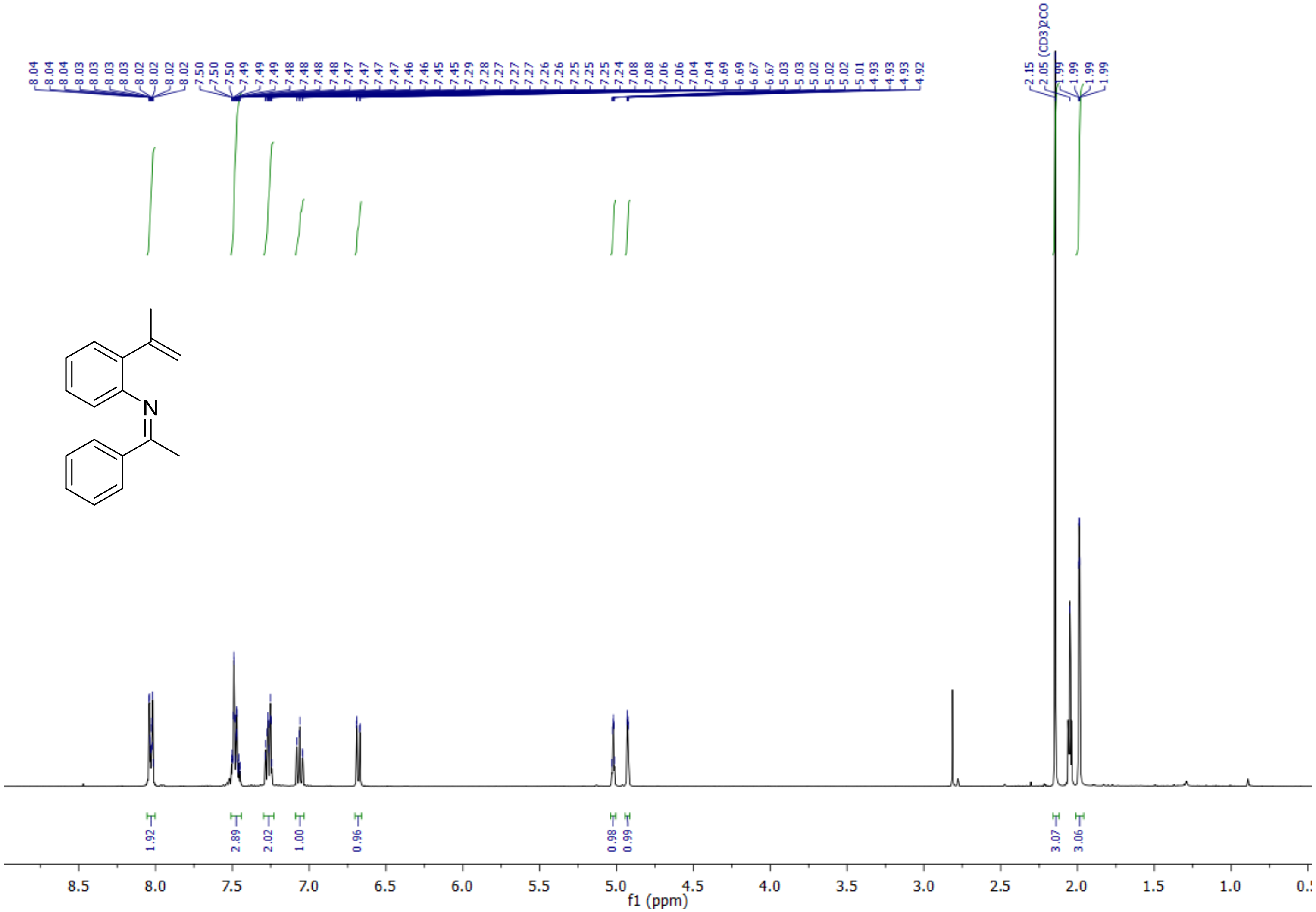

^{13}C NMR (101 MHz, acetone-d_6) of **1ag**

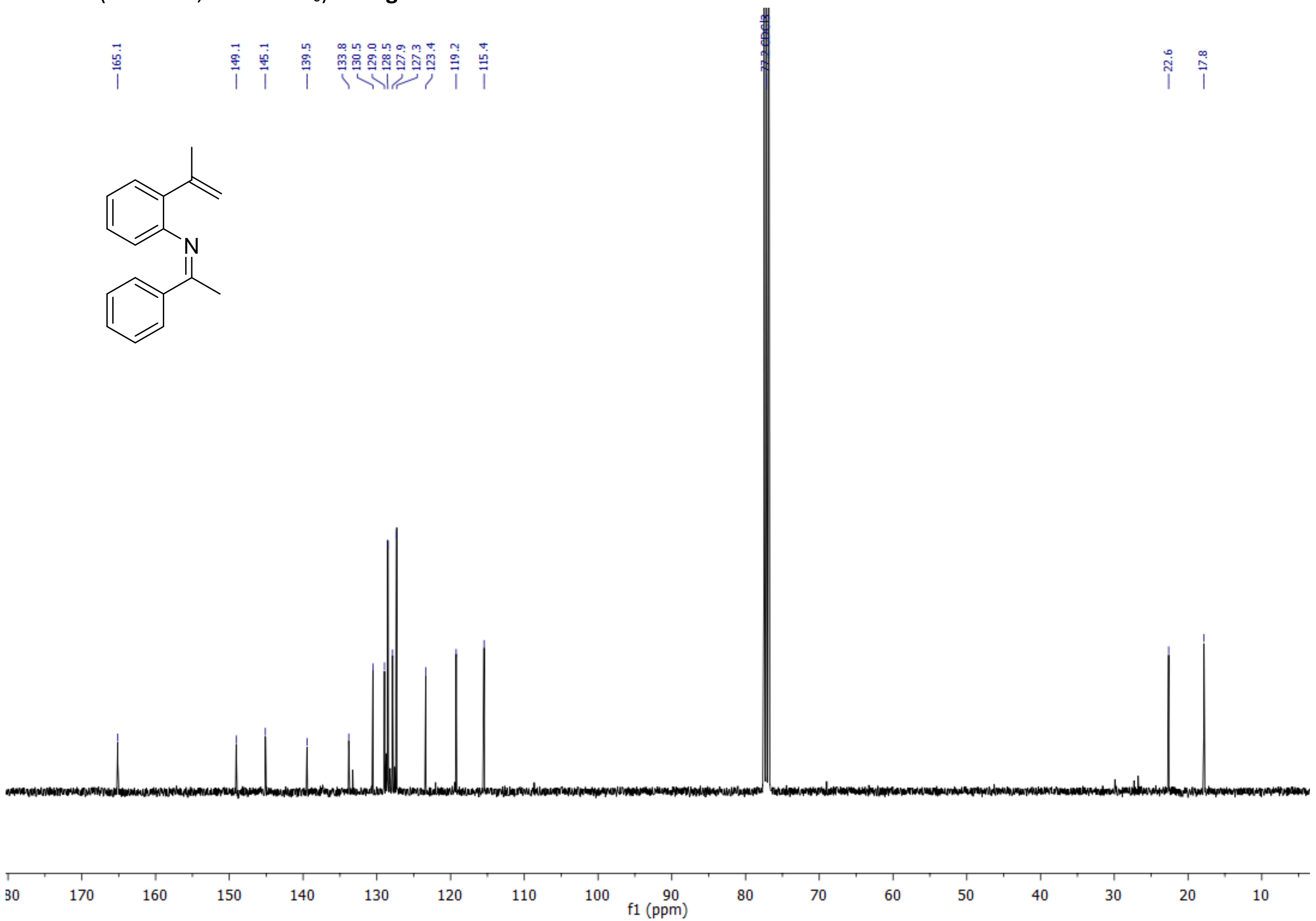

^{1}H–^{13}C HSQC-DEPT NMR (400 MHz, acetone-d_6) of **1ag**

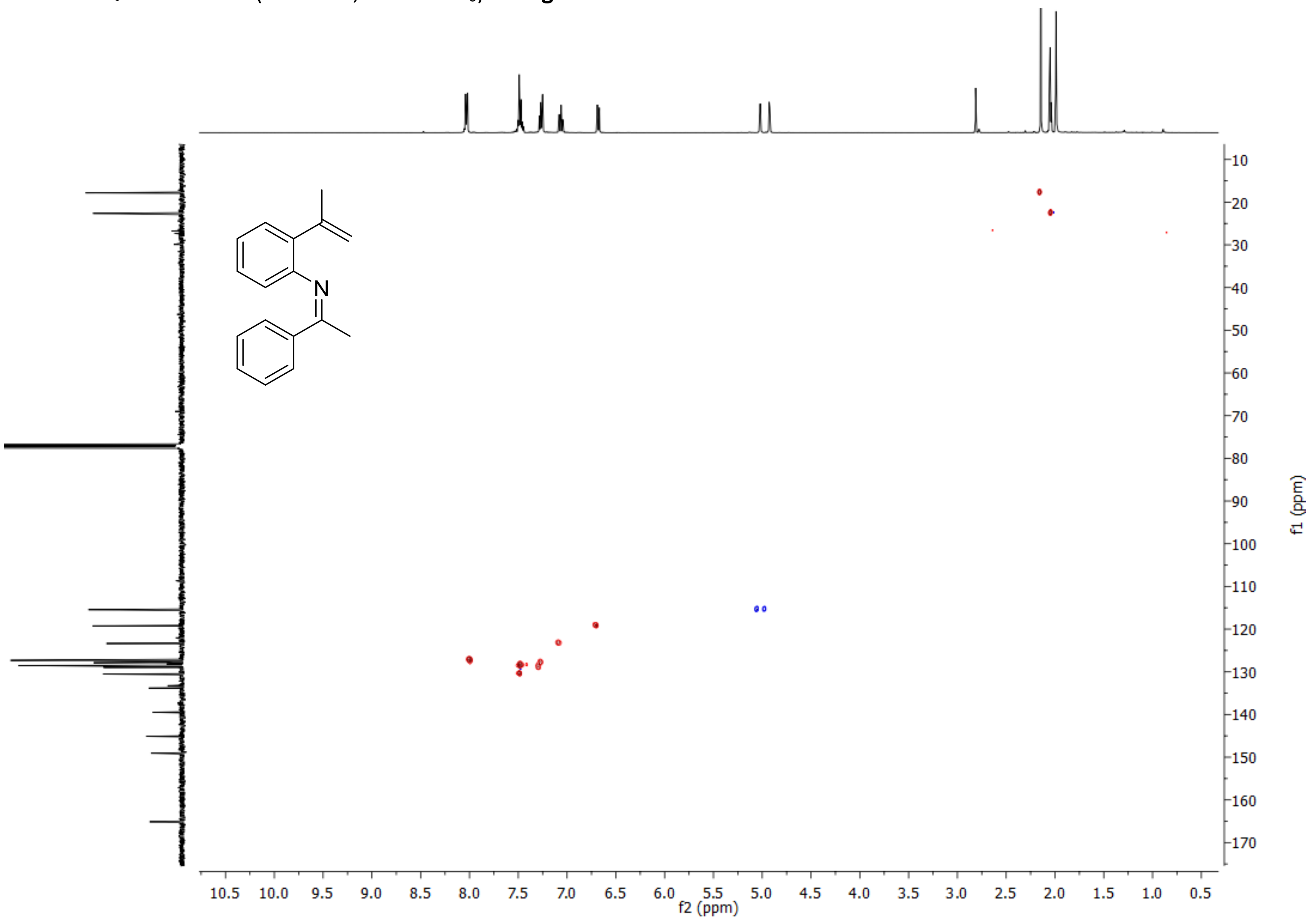

^{1}H NMR (400 MHz, $CDCl_3$) of **1ah**

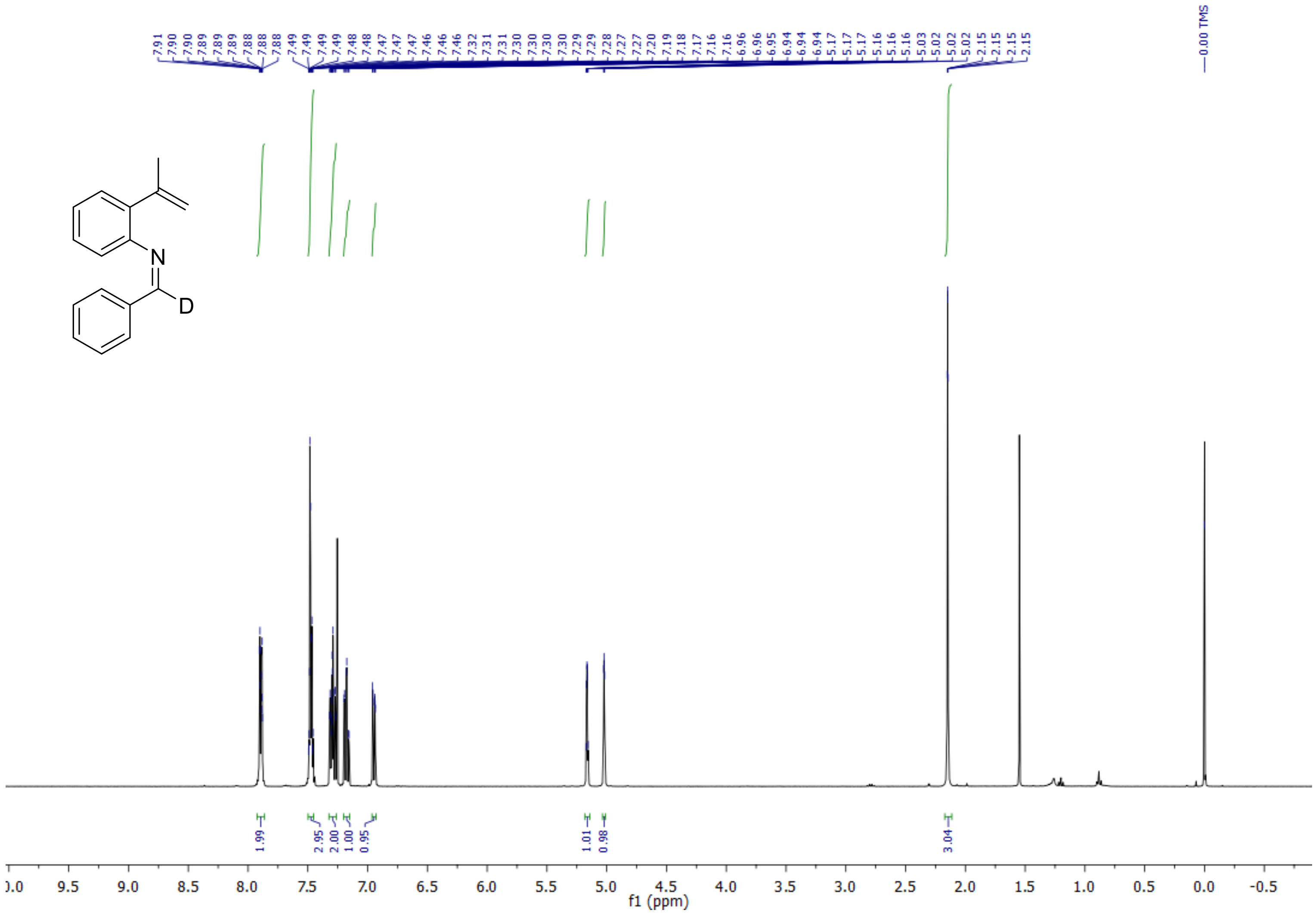

^{13}C NMR (101 MHz, $CDCl_3$) of **1ah**

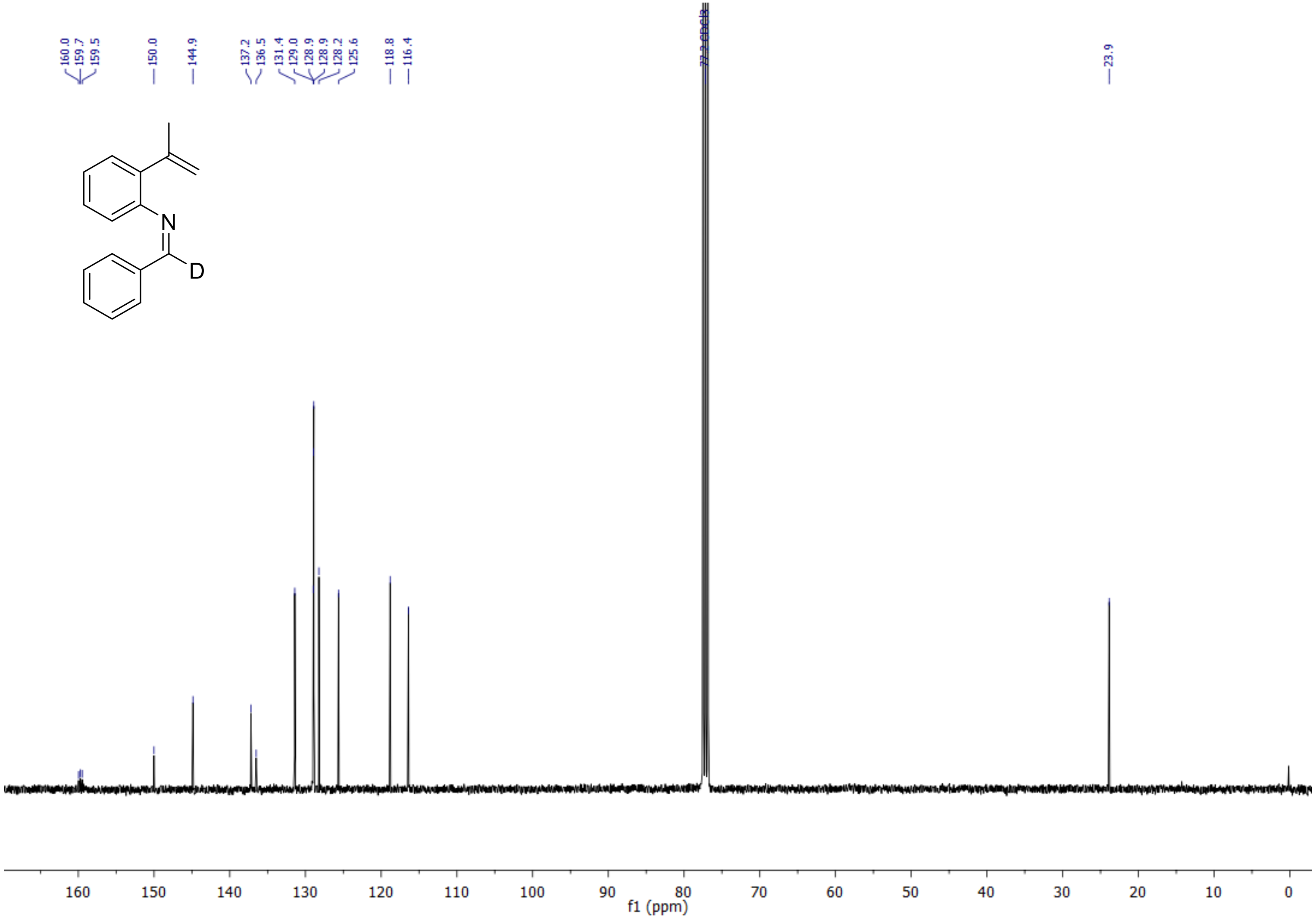

^{1}H–^{13}C HSQC-DEPT NMR (400 MHz, $CDCl_3$) of **1ah**

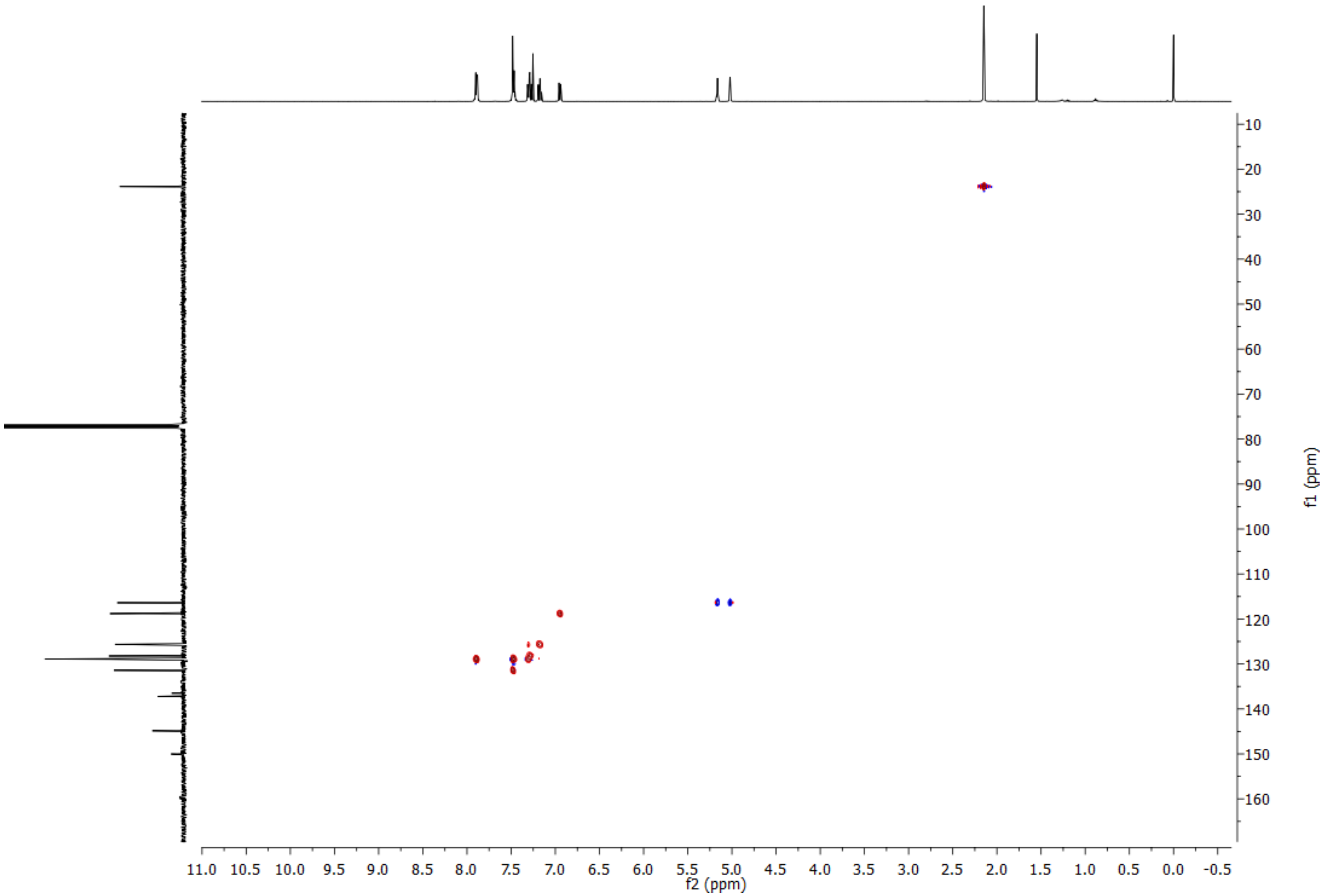

^{1}H NMR (400 MHz, acetone-d_6) of **2a**

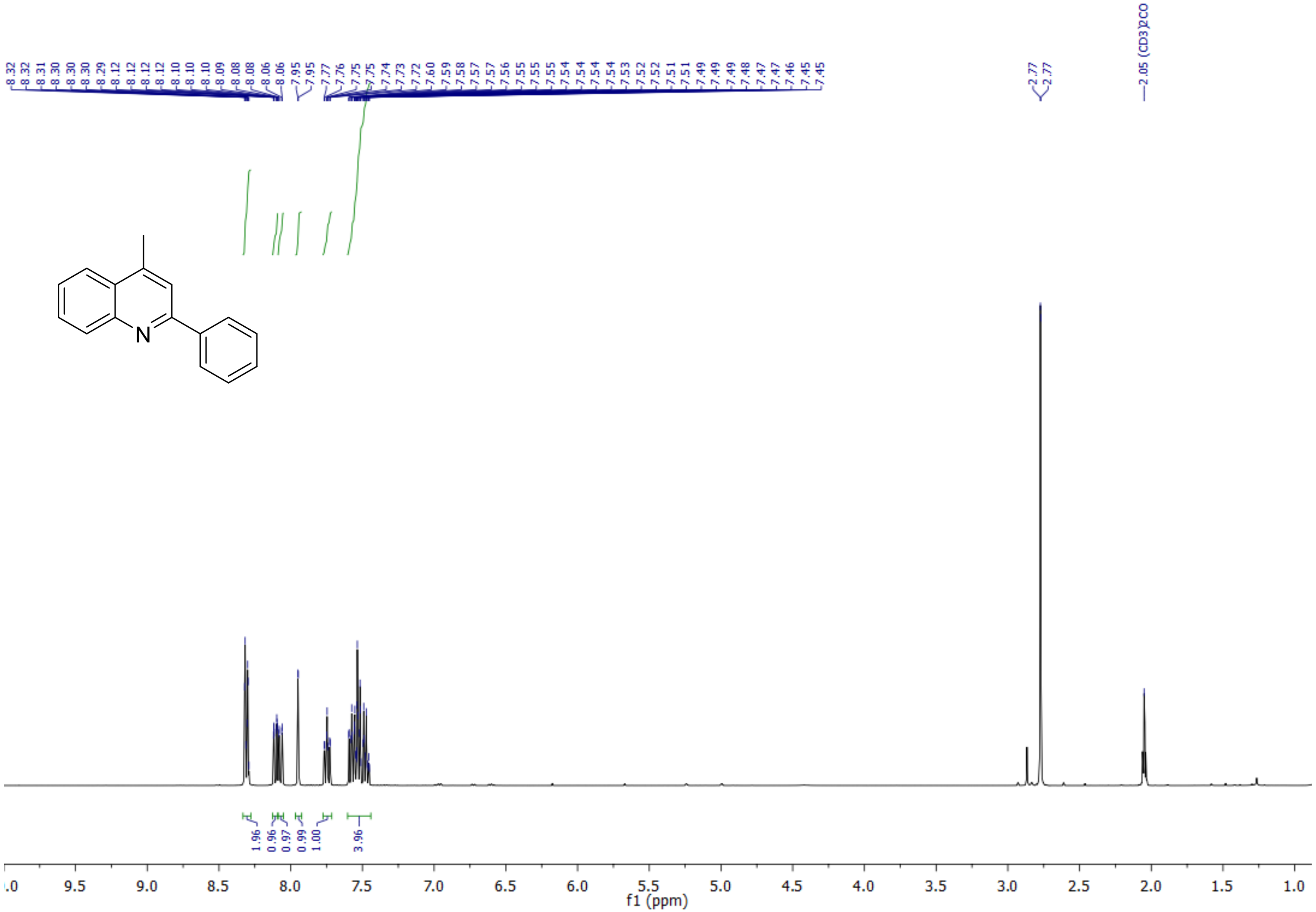

^{13}C NMR (101 MHz, acetone-d_6) of **2a**

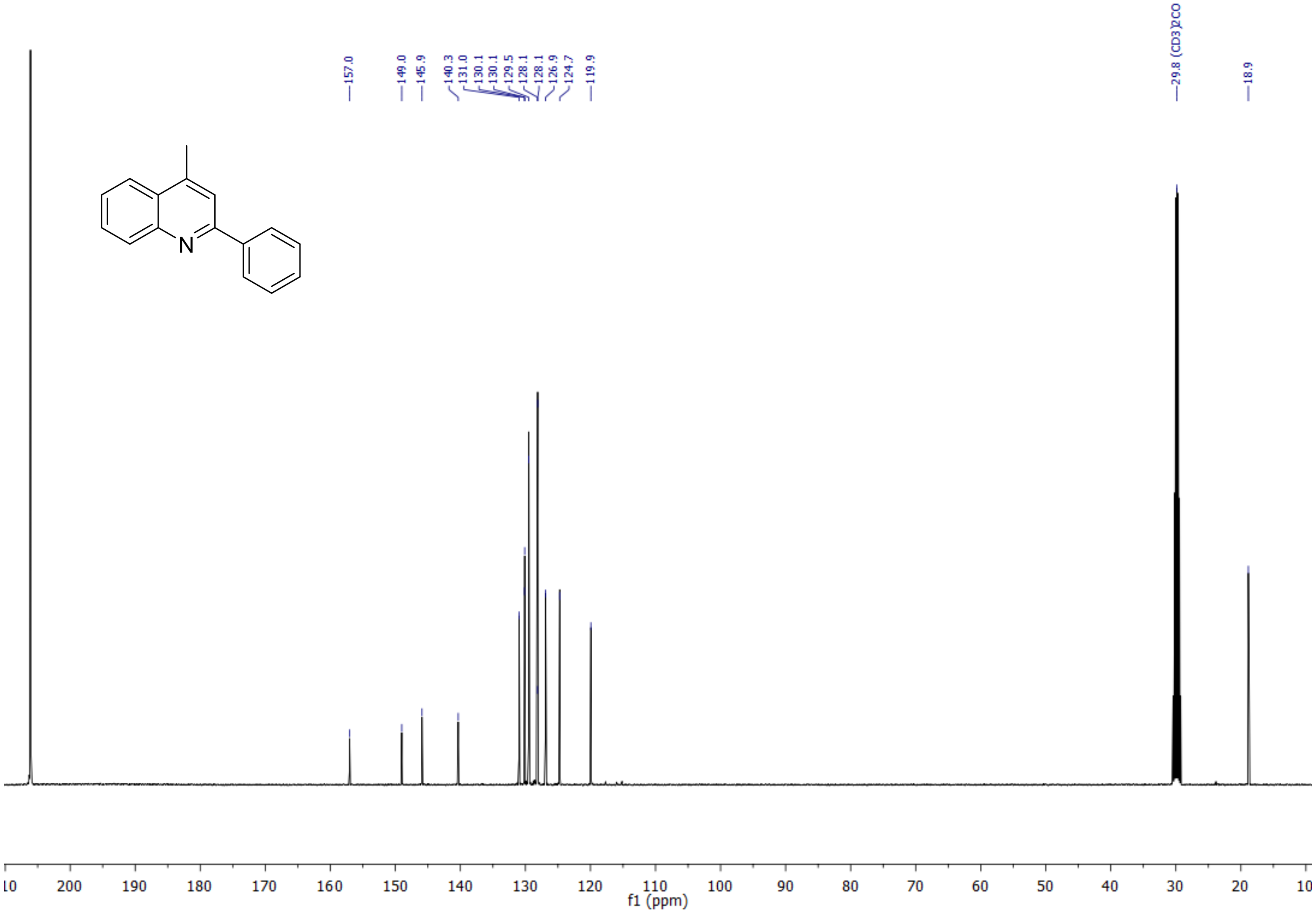

^{1}H NMR (400 MHz, acetone-d_6) of **2b**

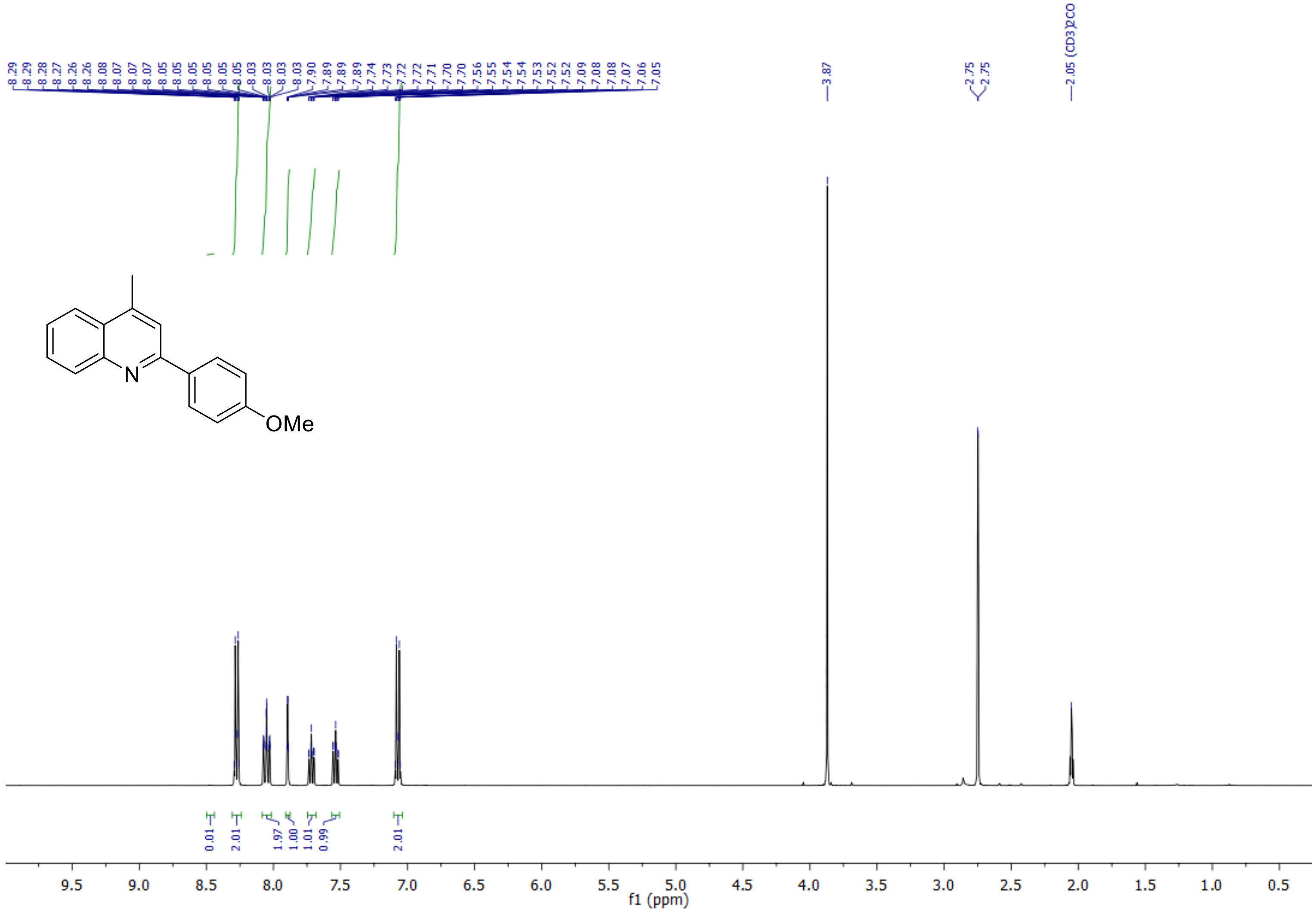

^{13}C NMR (101 MHz, acetone-d_6) of **2b**

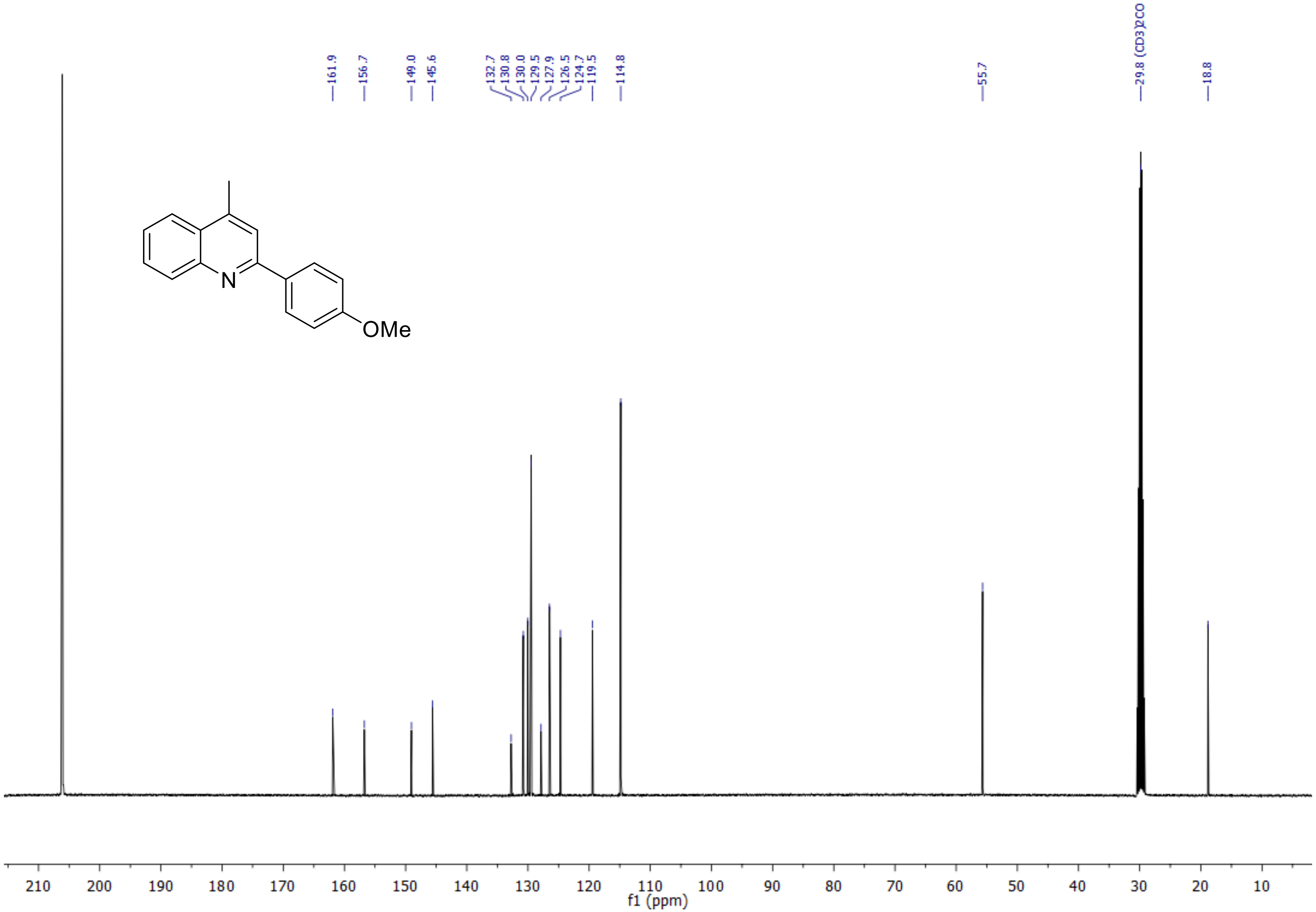

^{1}H NMR (400 MHz, $CDCl_3$) of **2c**

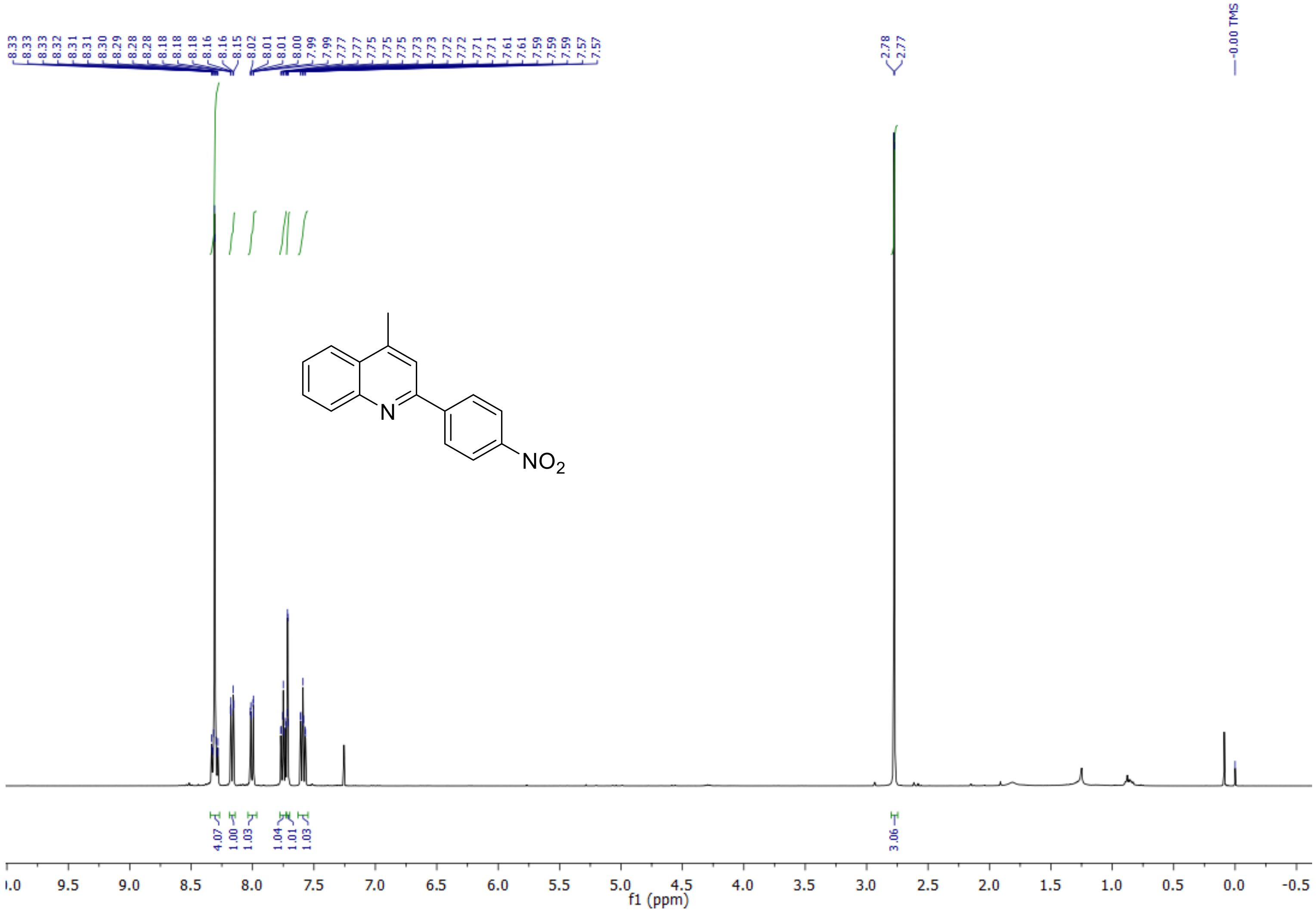

^{13}C NMR (101 MHz, $CDCl_3$) of **2c**

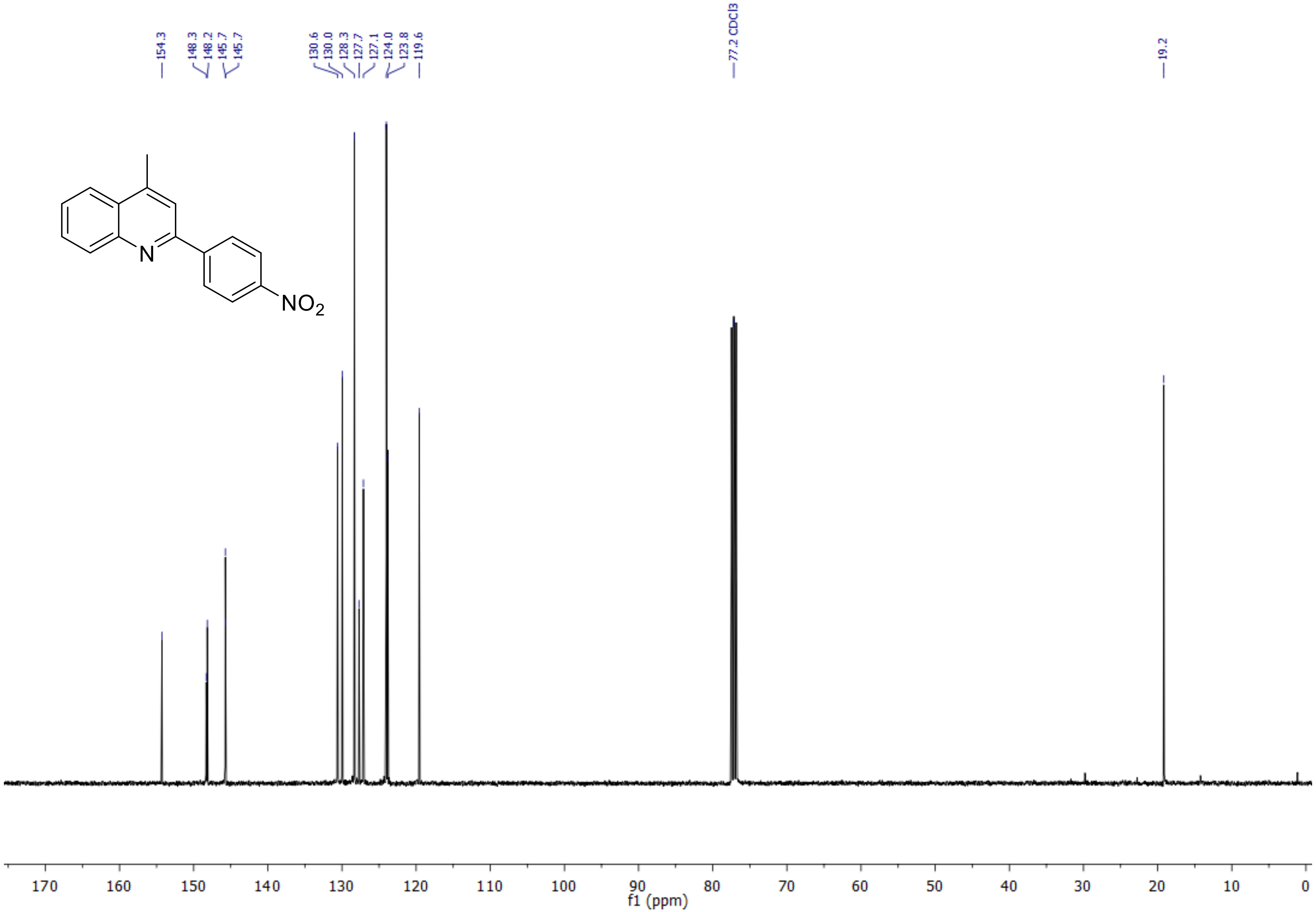

^{1}H NMR (400 MHz, $CDCl_3$) of **2d**

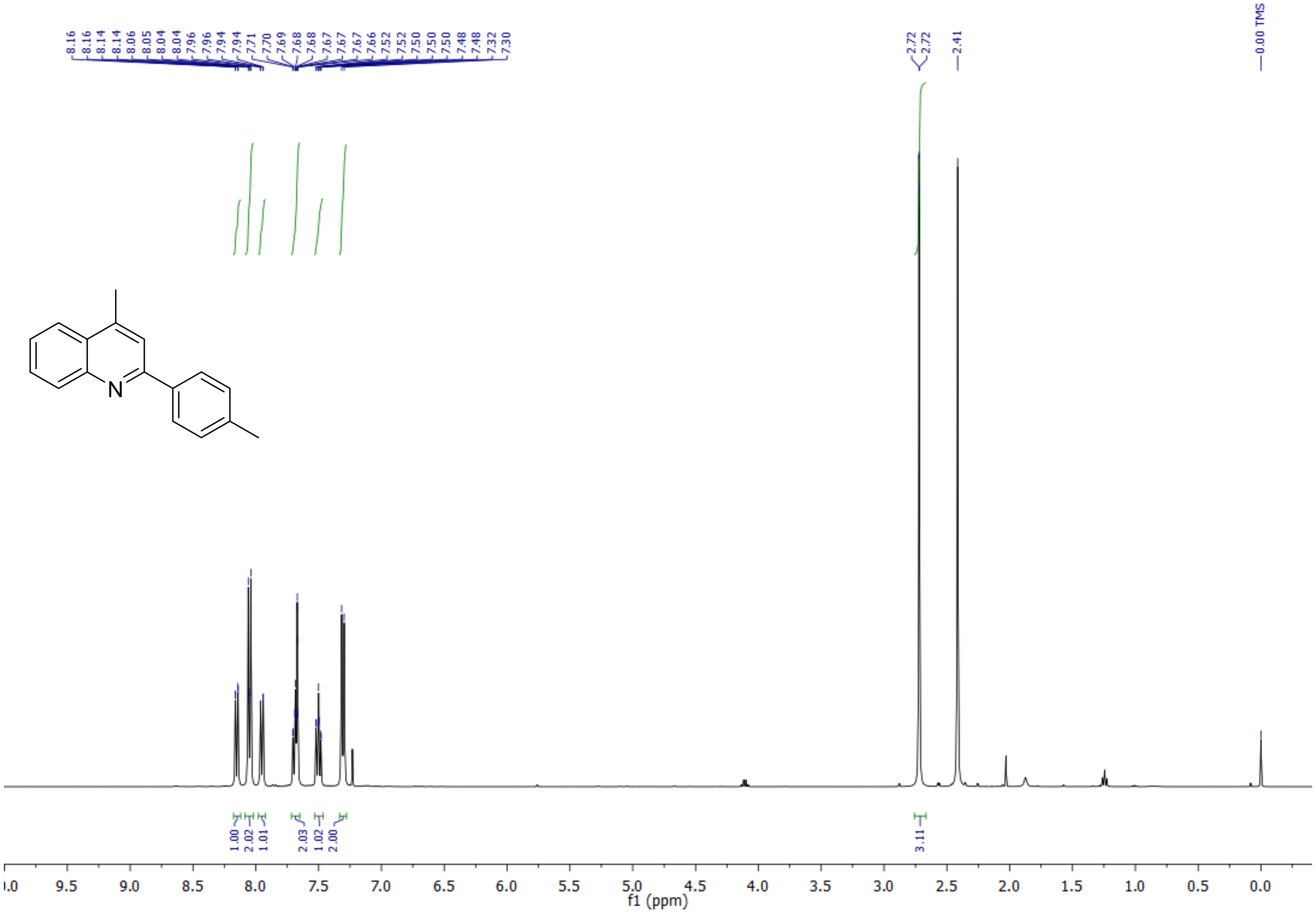

^{13}C NMR (101 MHz, $CDCl_3$) of **2d**

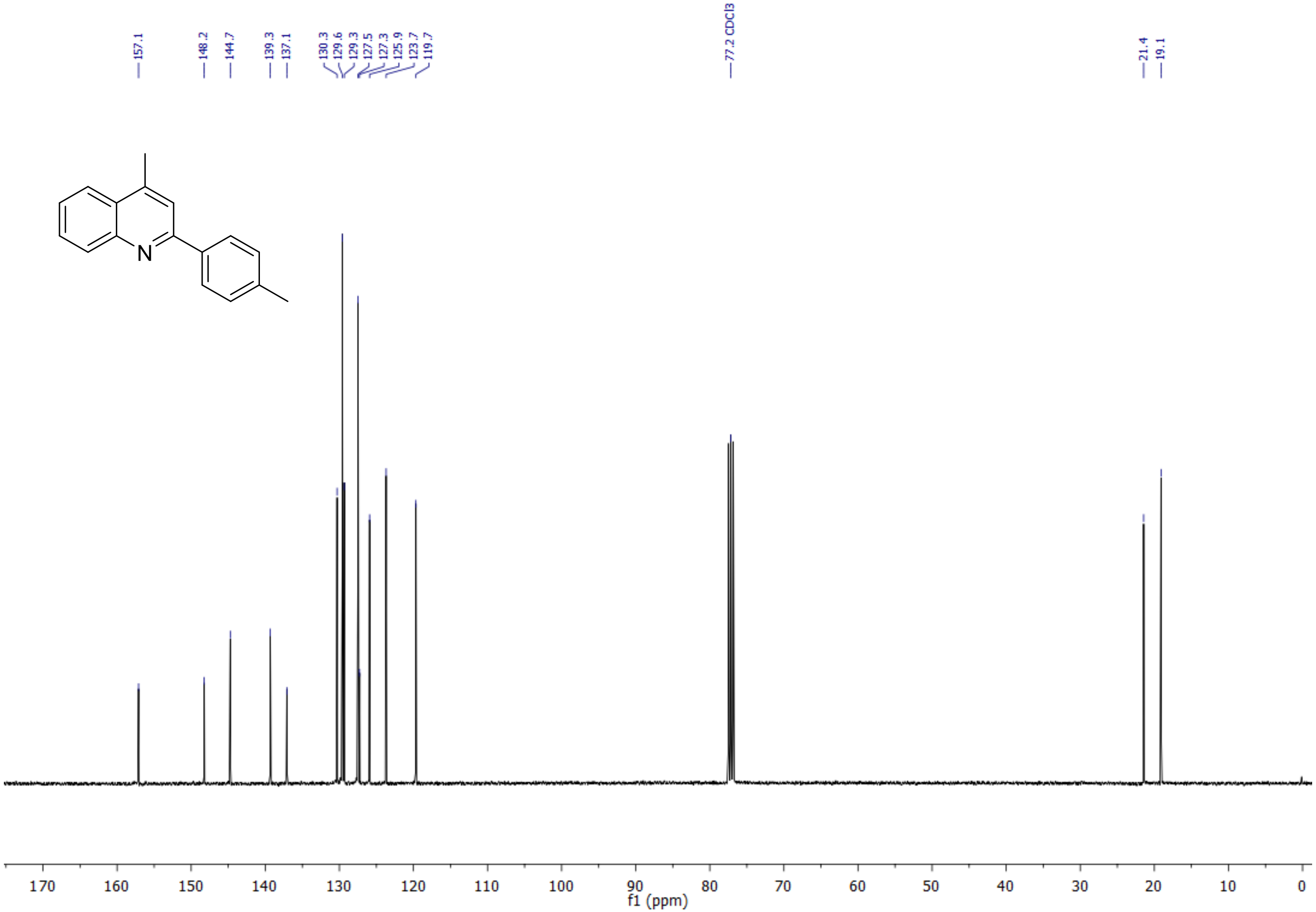

^{1}H NMR (400 MHz, $CDCl_3$) of **2e**

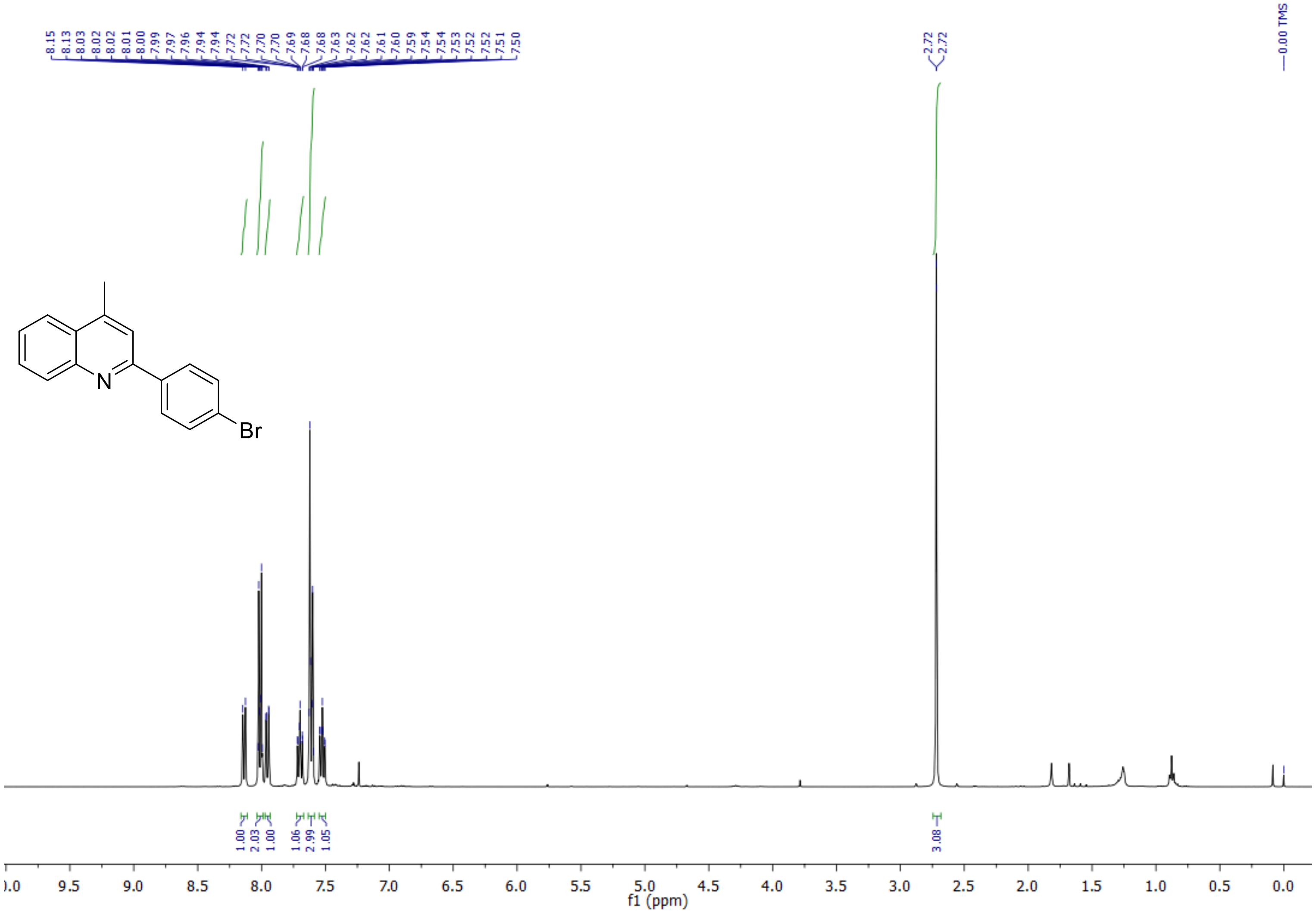

^{13}C NMR (101 MHz, $CDCl_3$) of **2e**

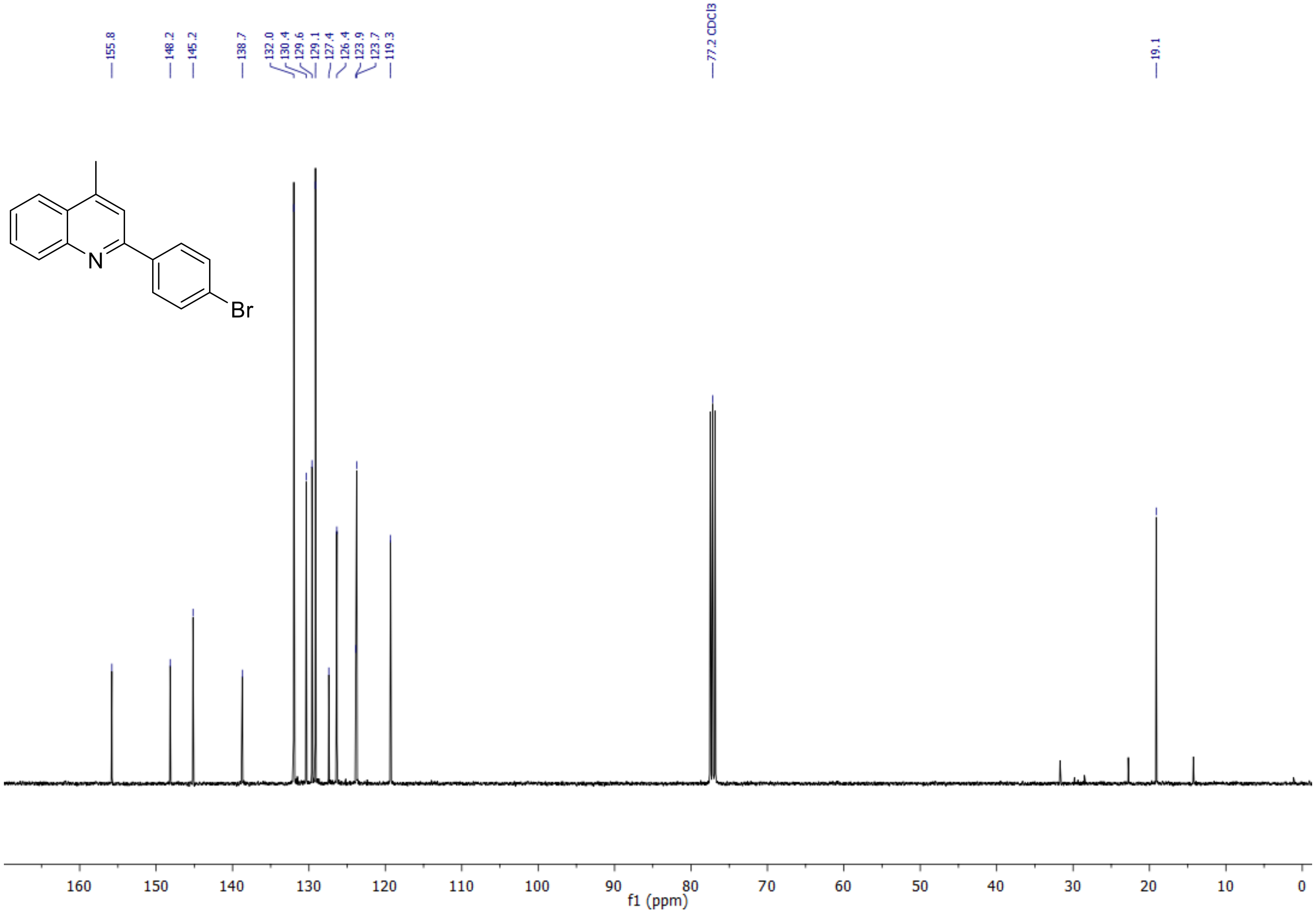

^{1}H NMR (400 MHz, $CDCl_3$) of **2f**

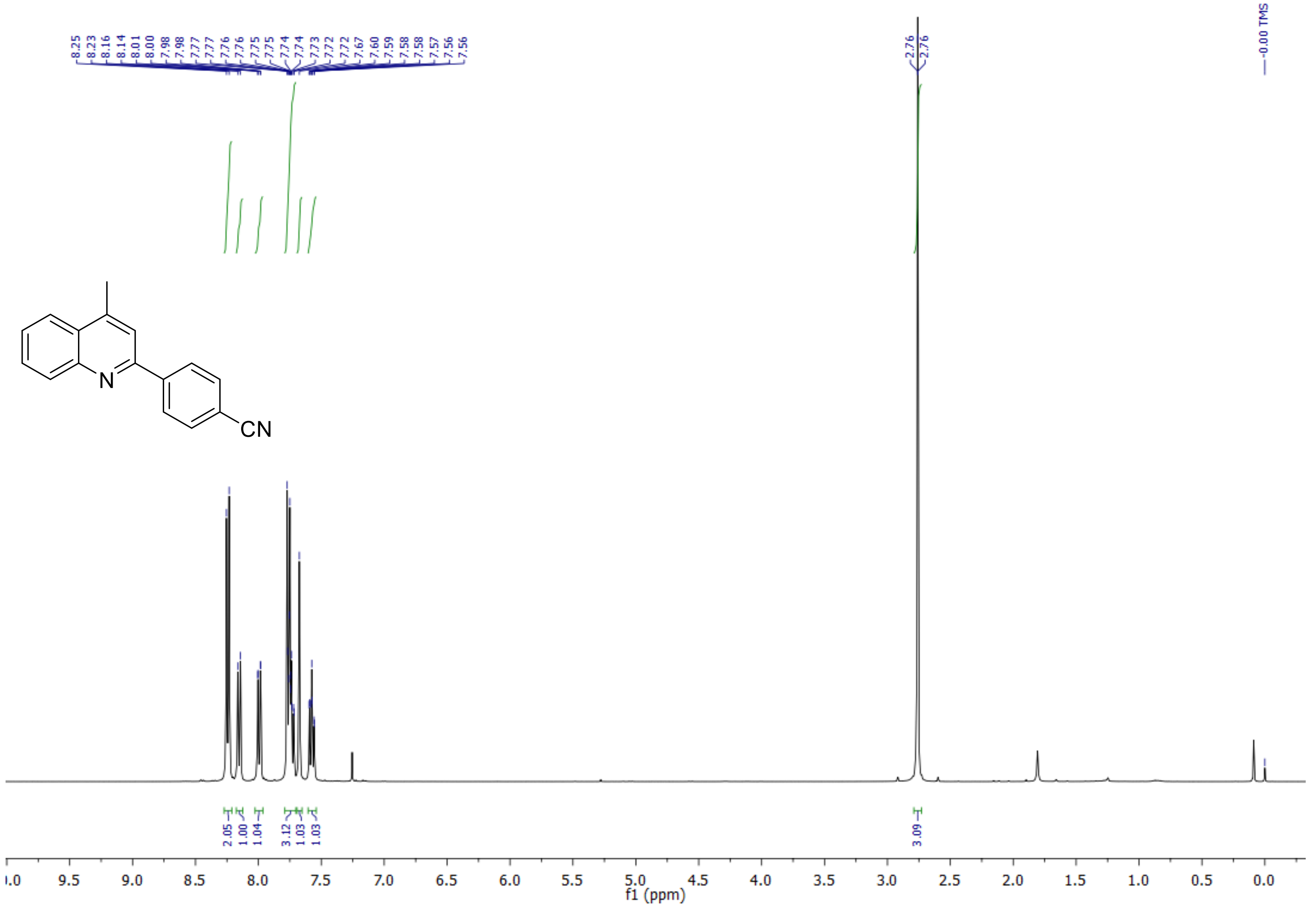

^{13}C NMR (101 MHz, $CDCl_3$) of **2f**

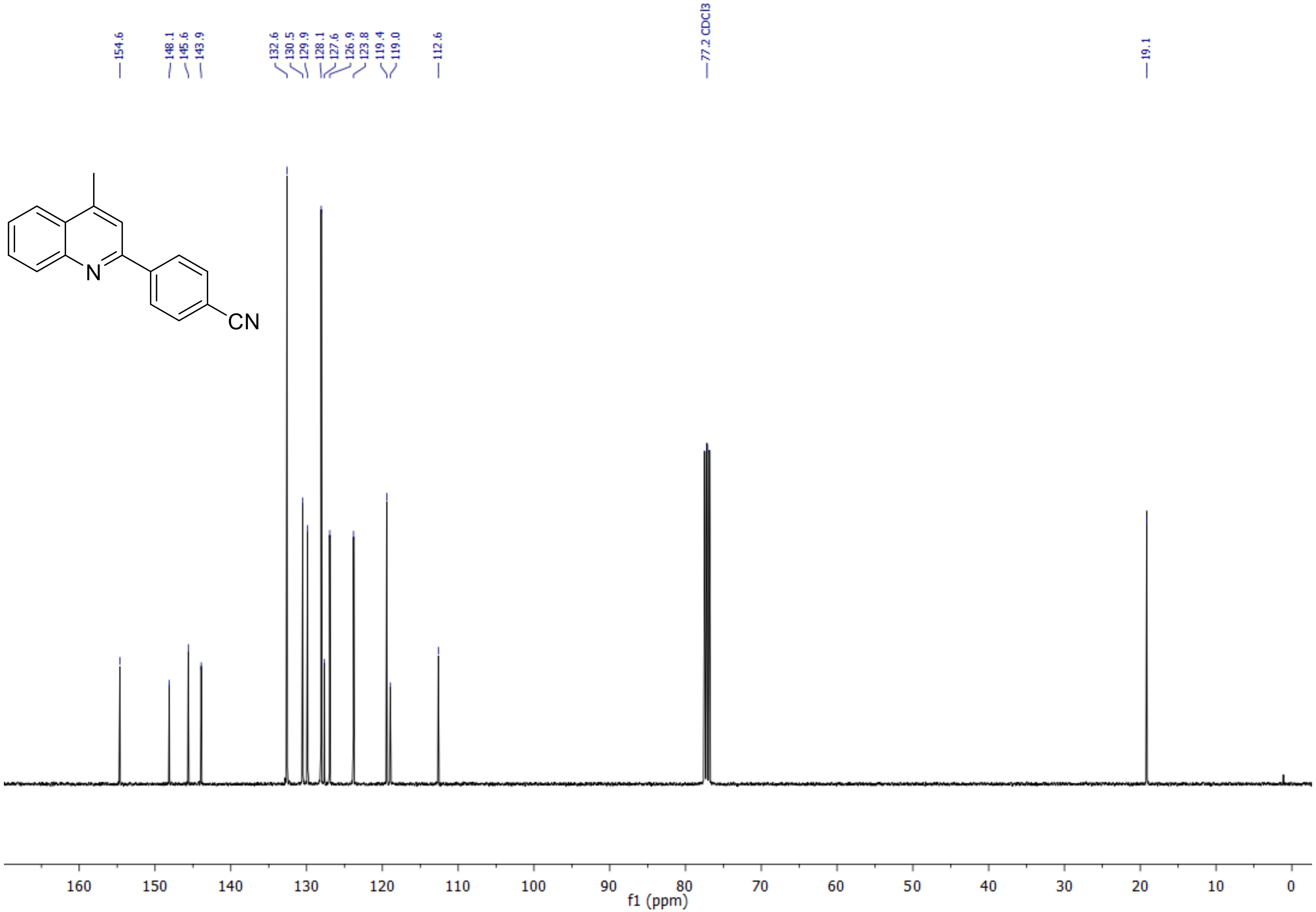

^{1}H NMR (400 MHz, acetone-d_6) of **2g**

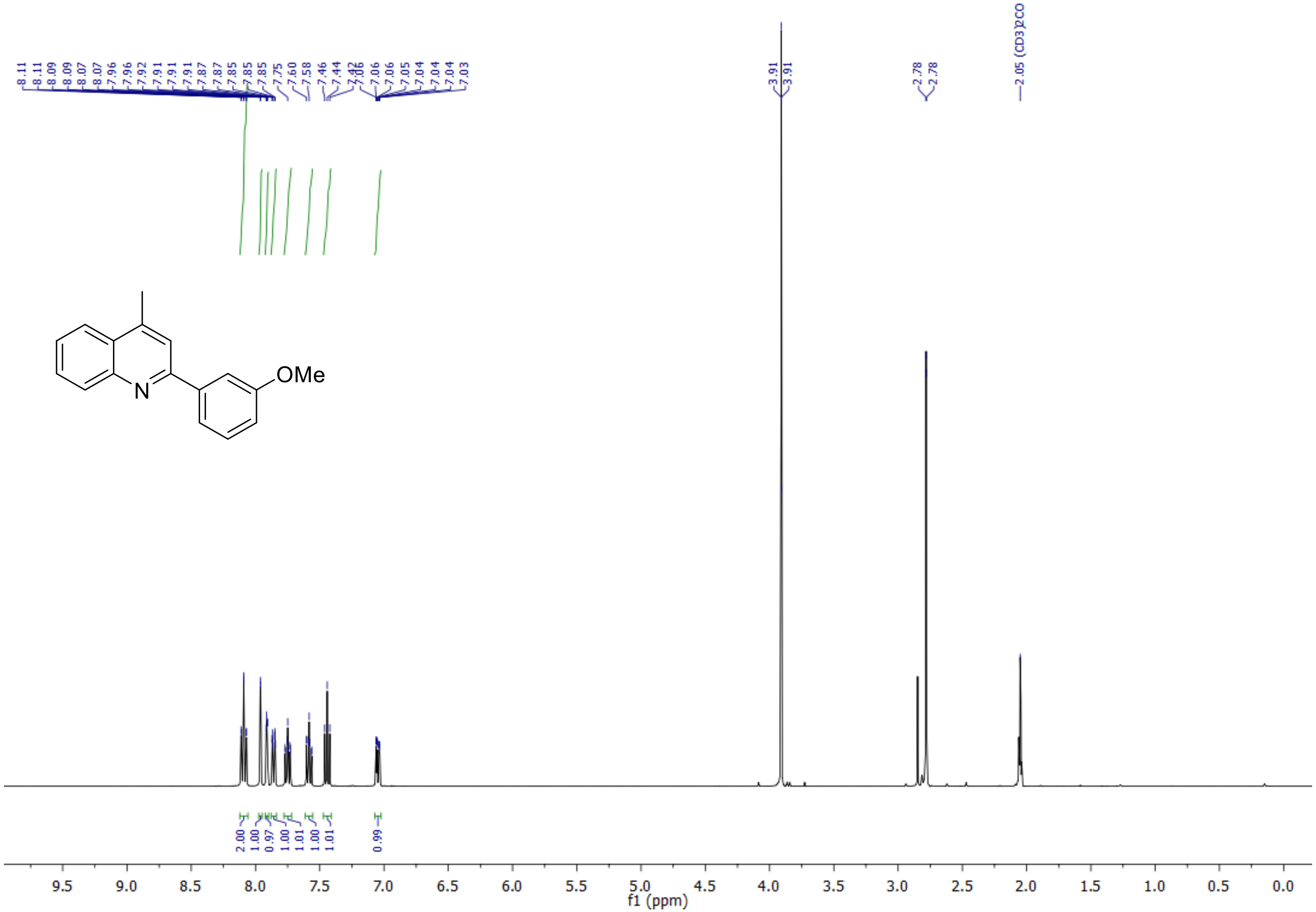

^{13}C NMR (101 MHz, acetone-d_6) of **2g**

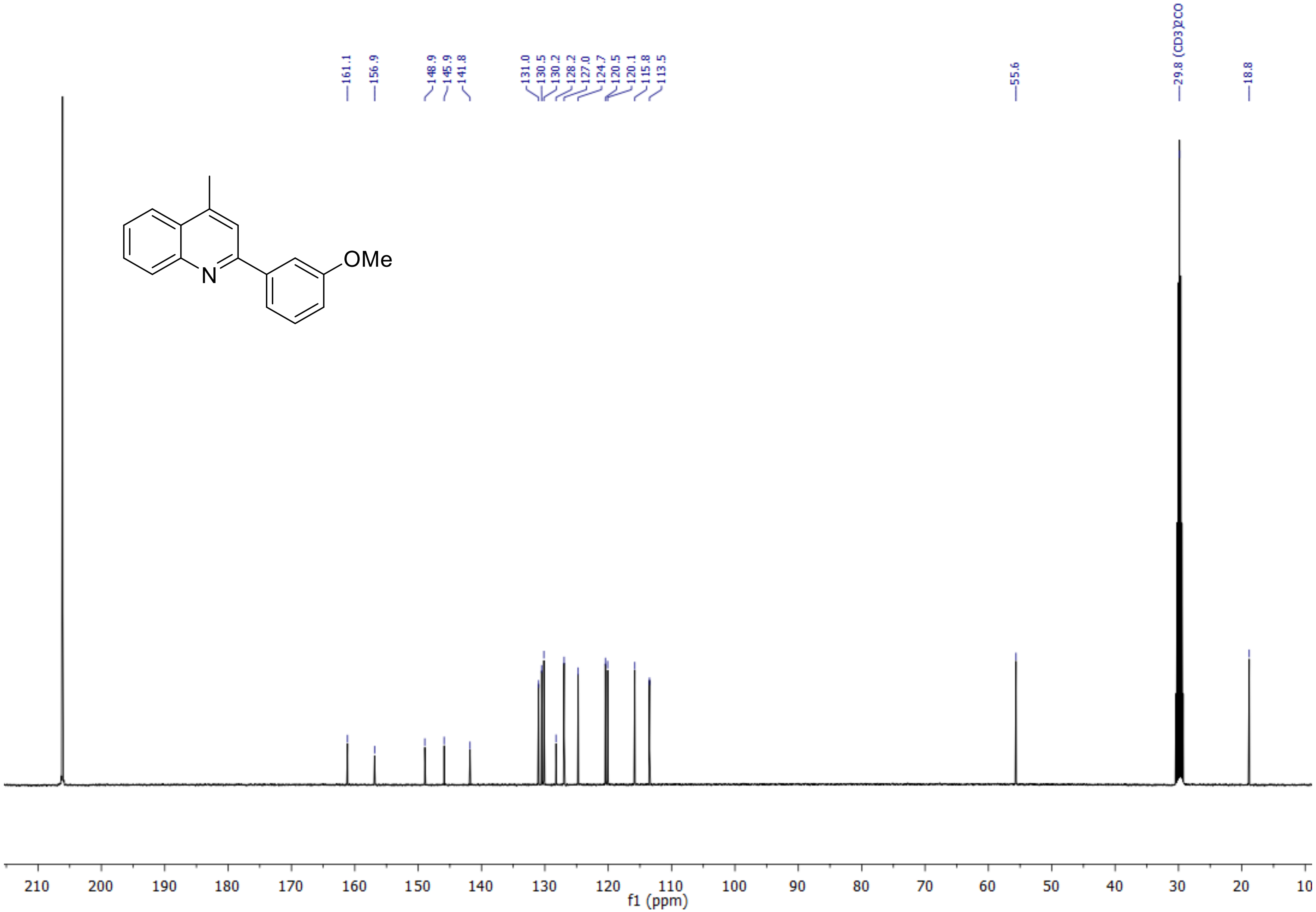

^1H NMR (400 MHz, $CDCl_3$) of **2h**

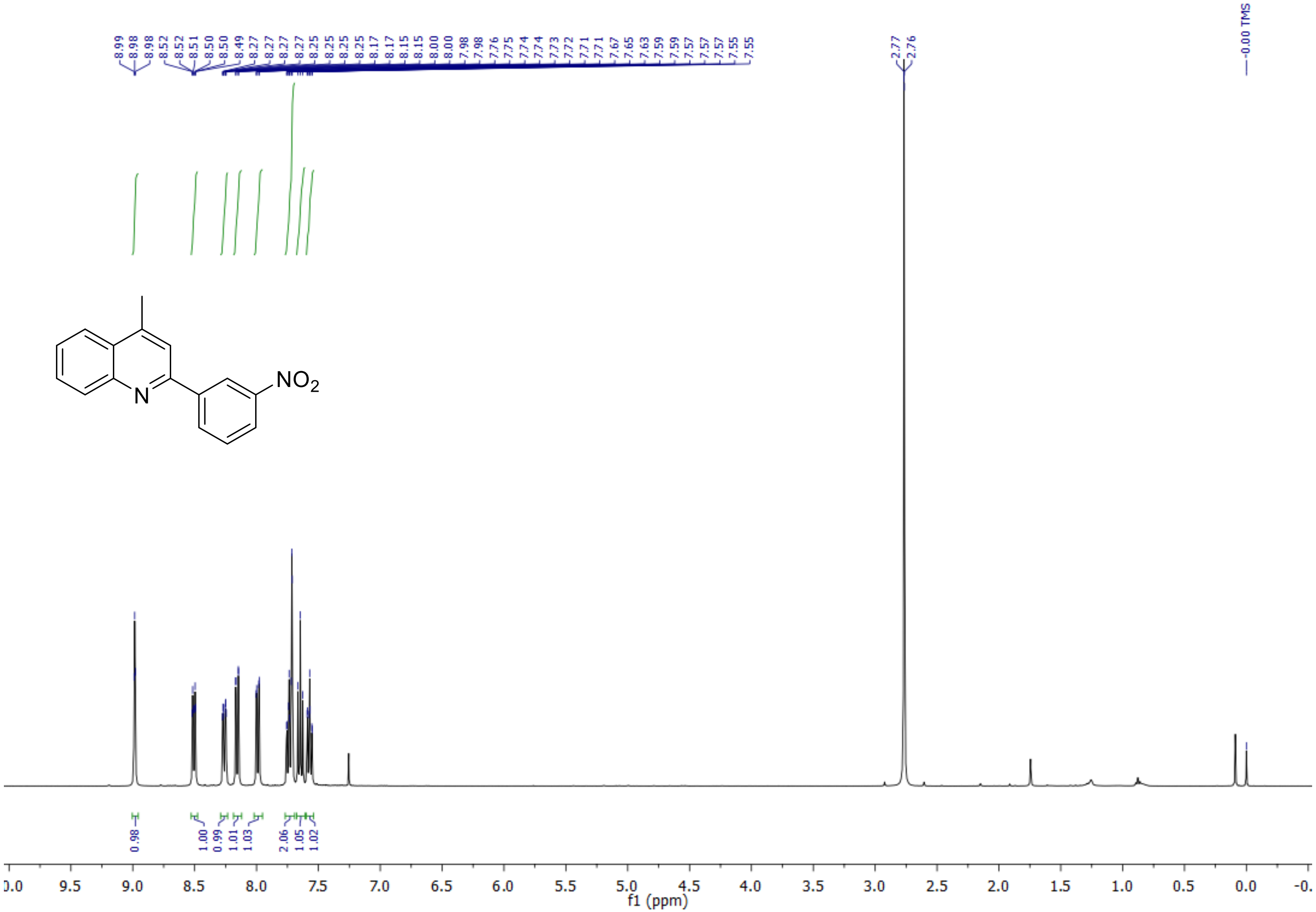

^{13}C NMR (101 MHz, $CDCl_3$) of **2h**

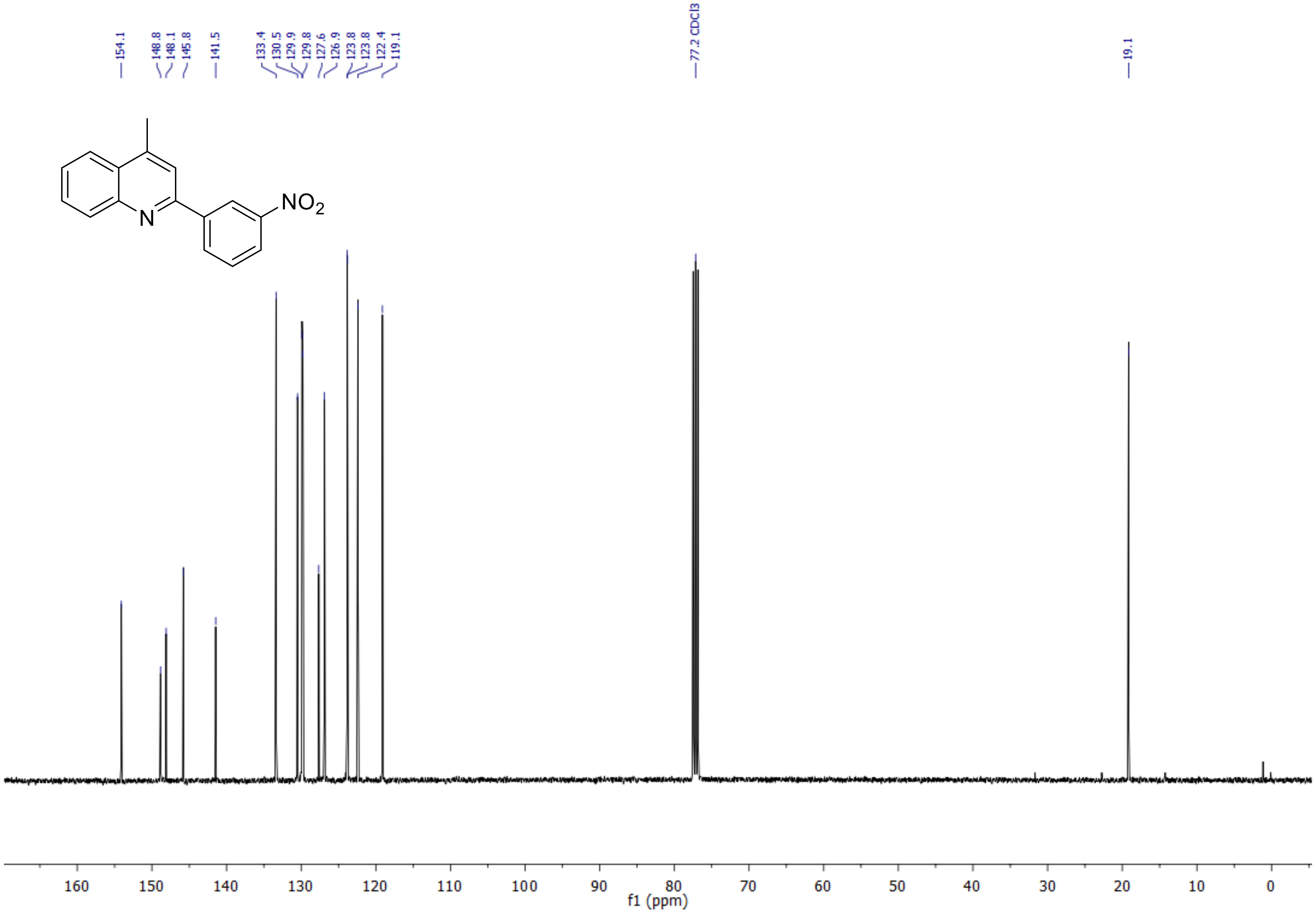

$^{1}H–^{13}C$ HSQC-DEPT NMR (400 MHz, $CDCl_3$) of **2h**

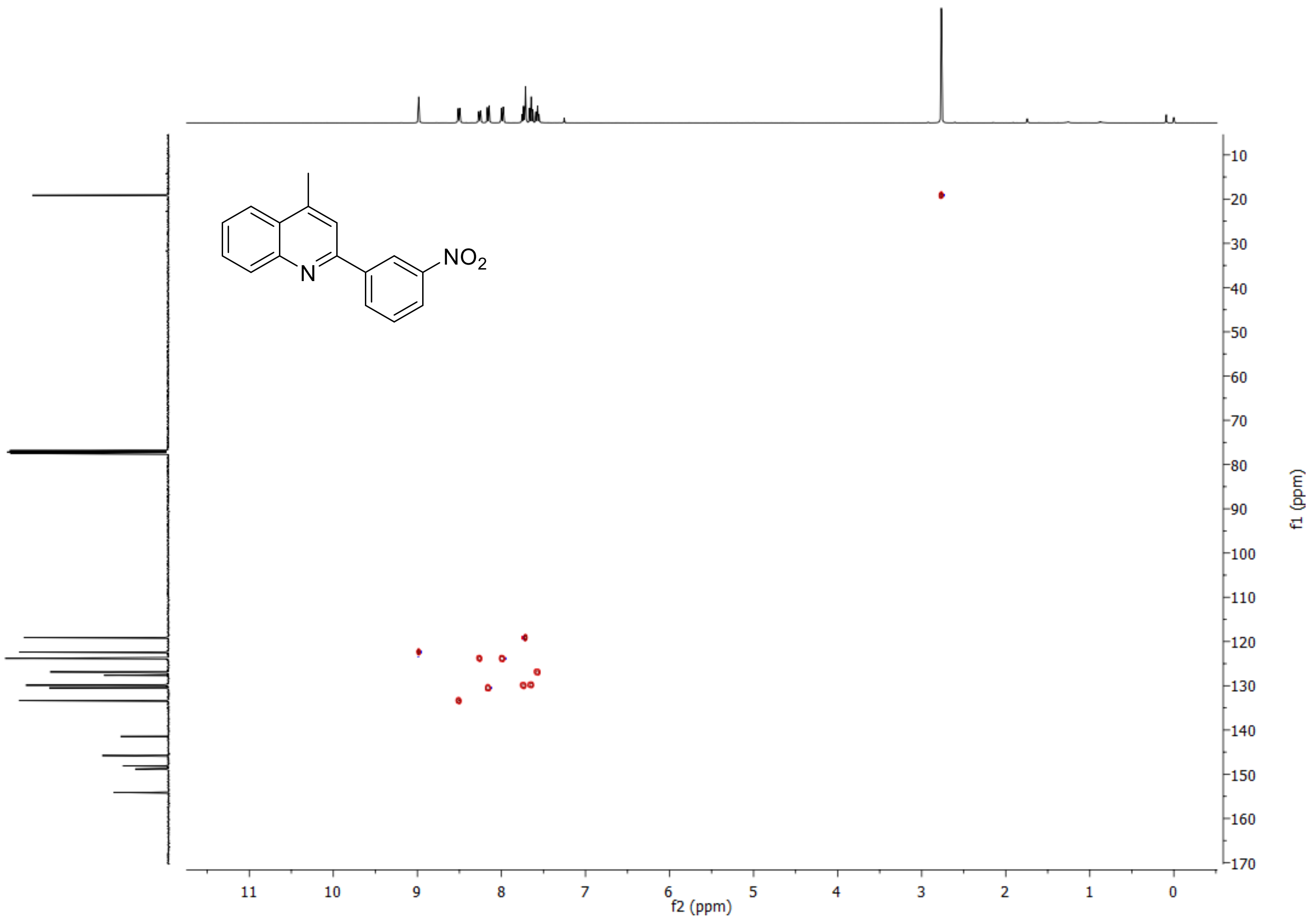

^{1}H NMR (400 MHz, acetone-d_6) of **2i**

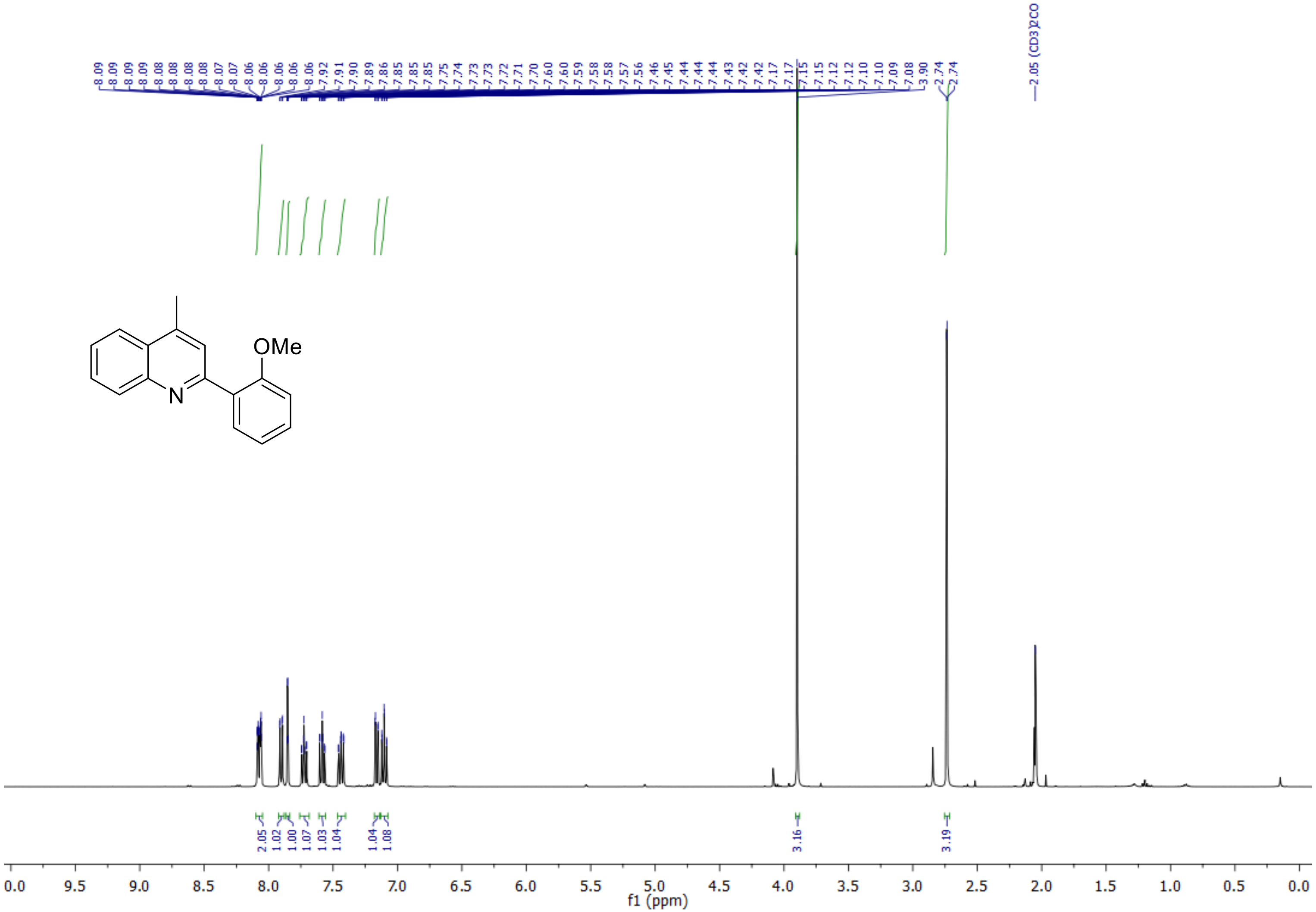

^{13}C NMR (101 MHz, acetone-d_6) of **2i**

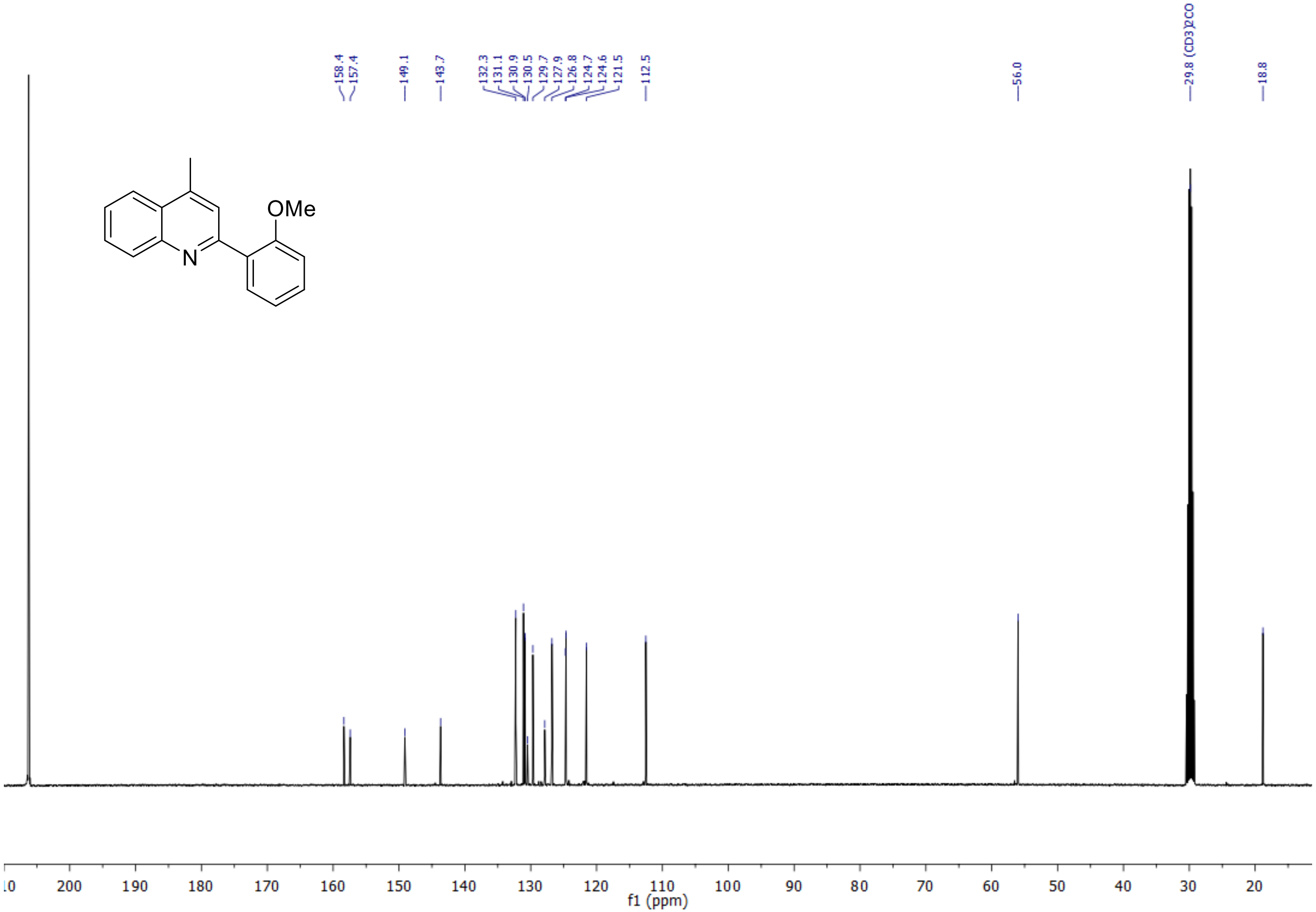

^{1}H NMR (400 MHz, acetone-d_6) of **2j**

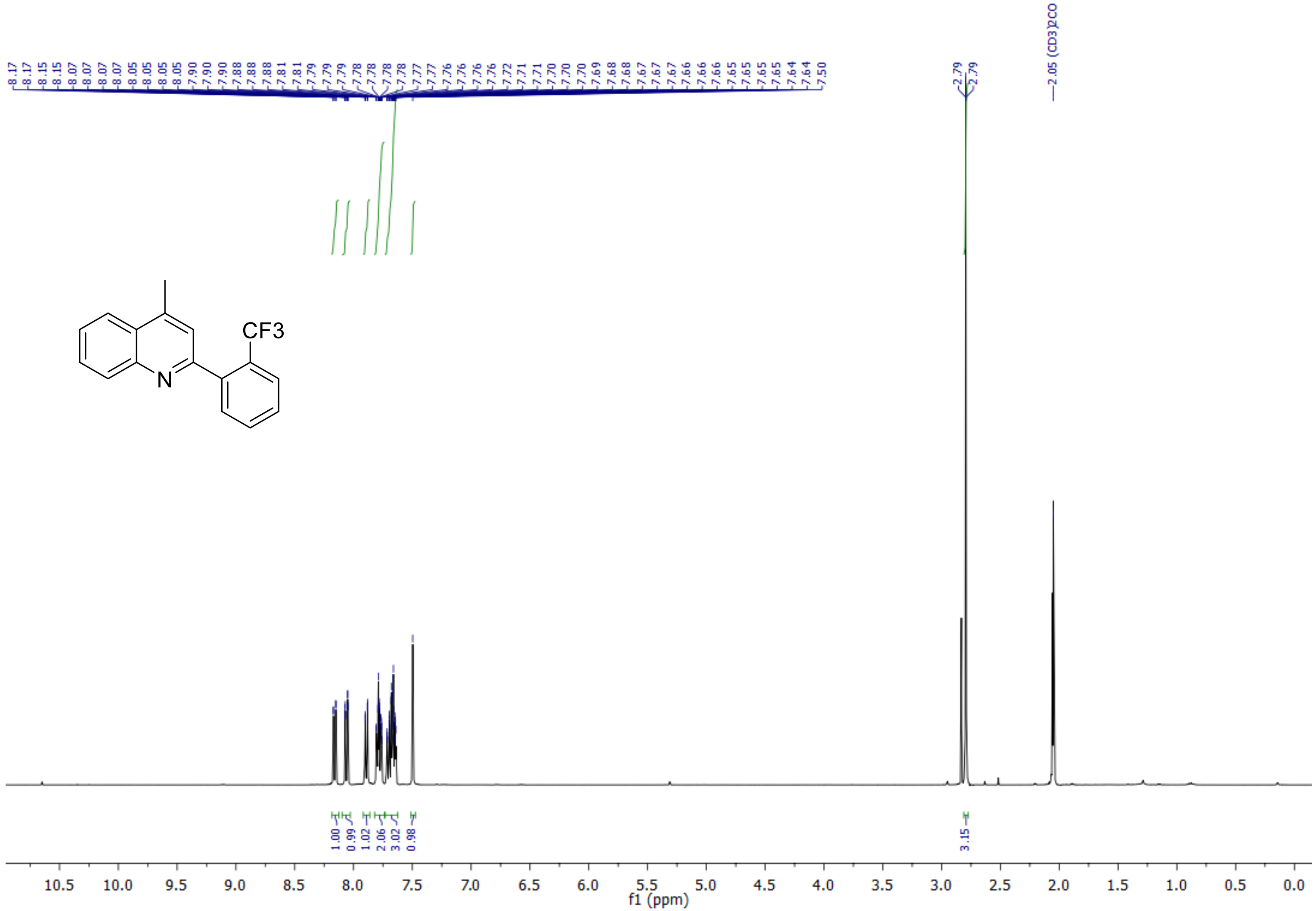

^{13}C NMR (101 MHz, acetone-d_6) of **2j**

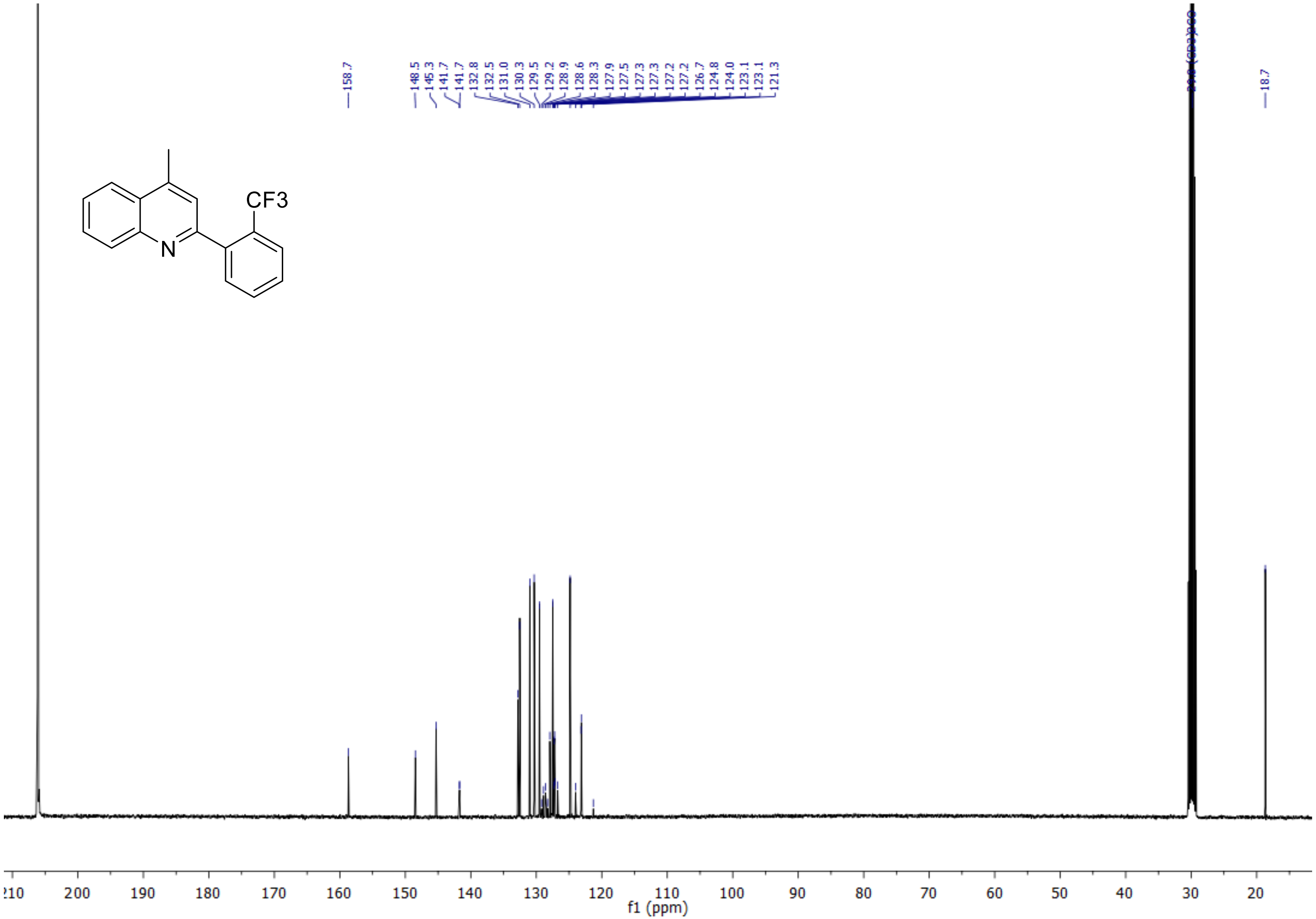

^{19}F NMR (377 MHz, acetone-d_6) of **2j**

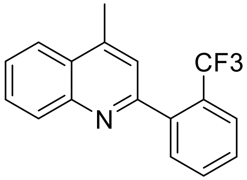

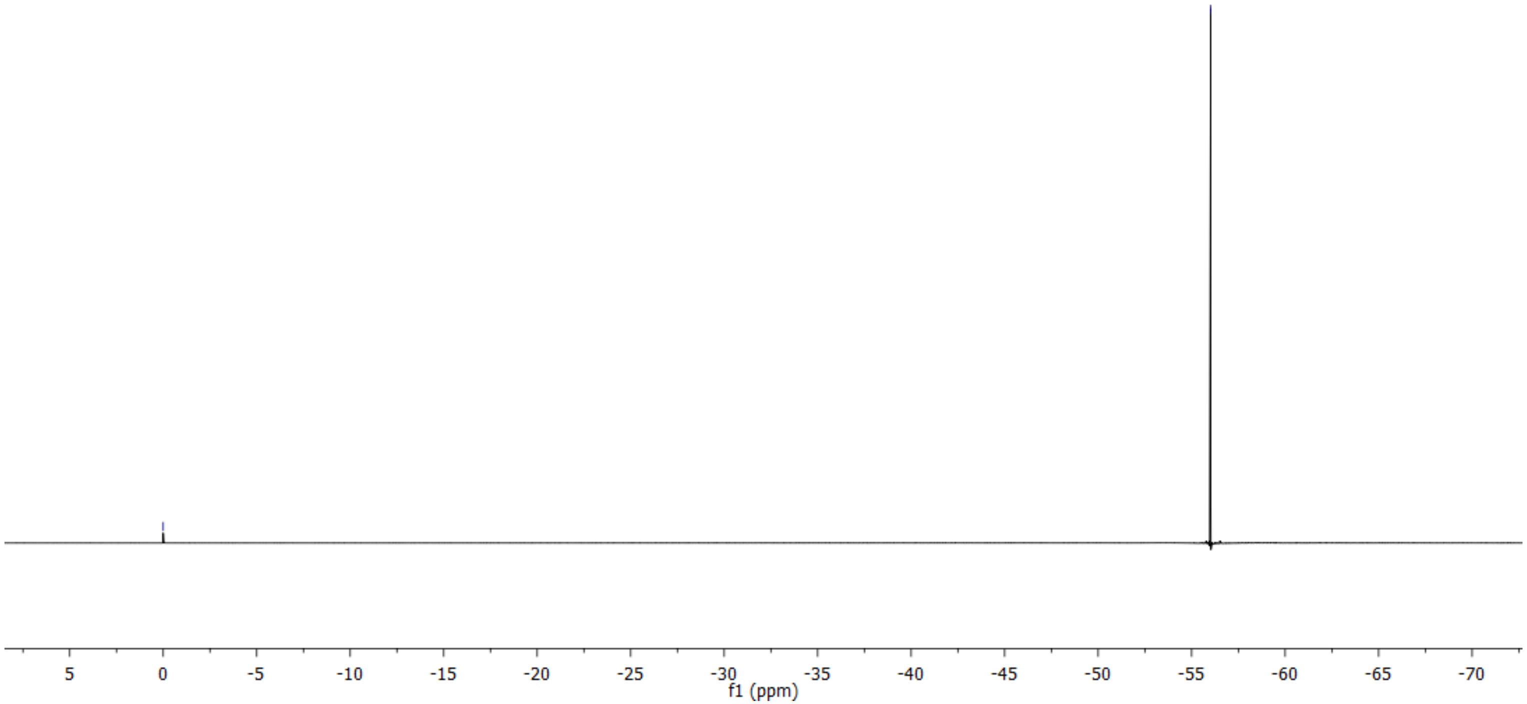

$^{1}H–^{13}C$ HSQC-DEPT NMR (400 MHz, acetone-d_6) of **2j**

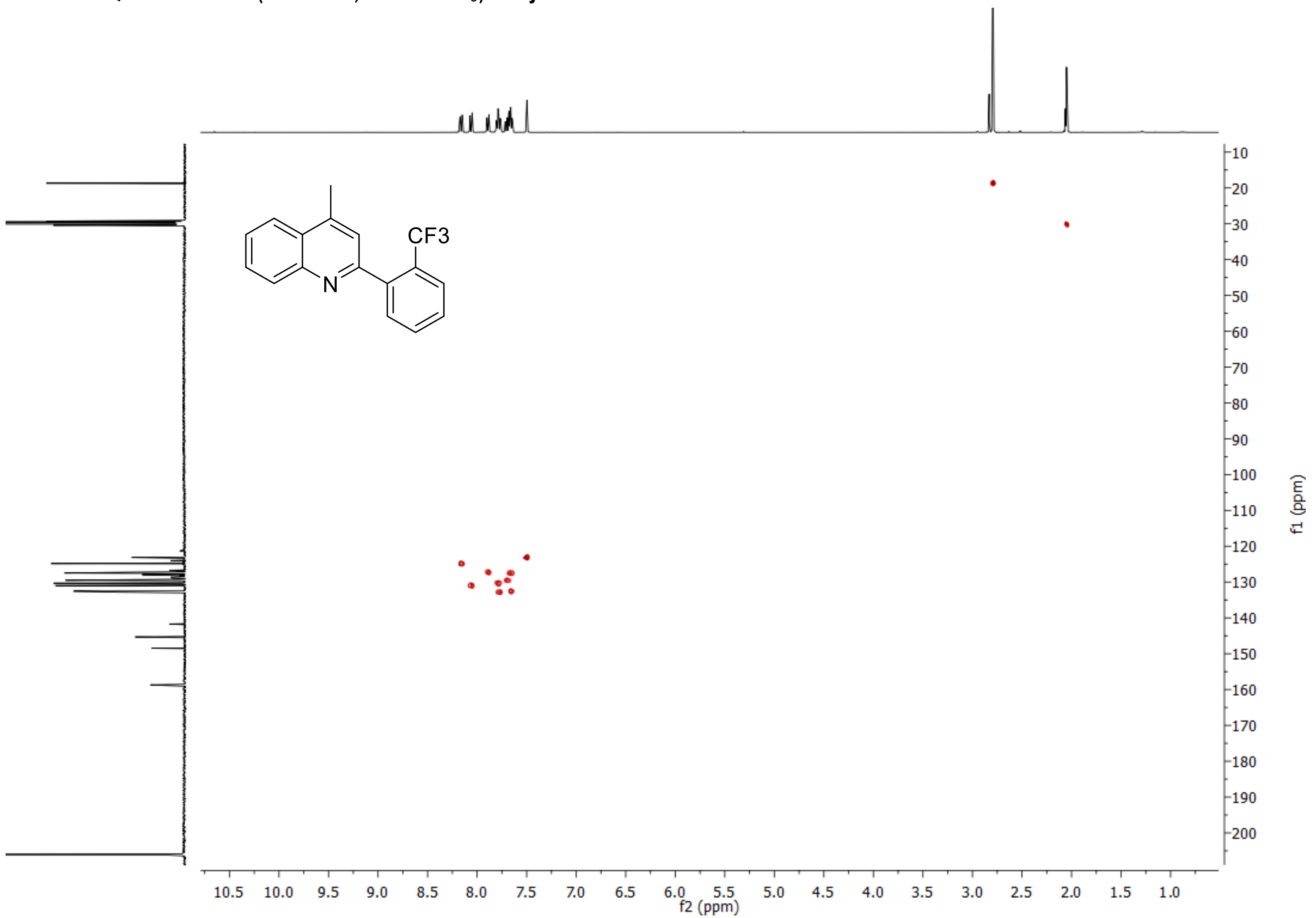

^{1}H NMR (400 MHz, $CDCl_3$) of **2k**

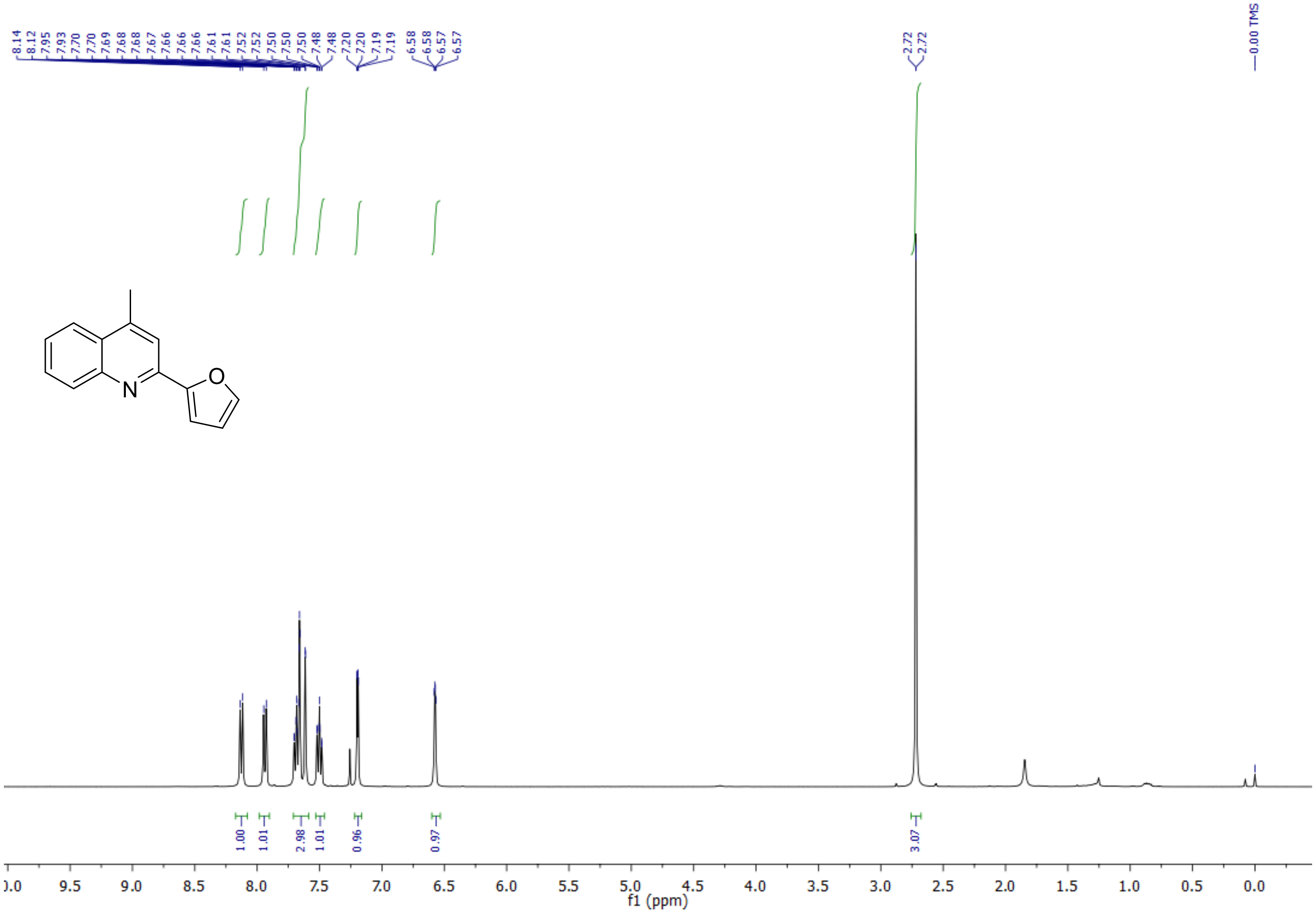

^{13}C NMR (101 MHz, $CDCl_3$) of **2k**

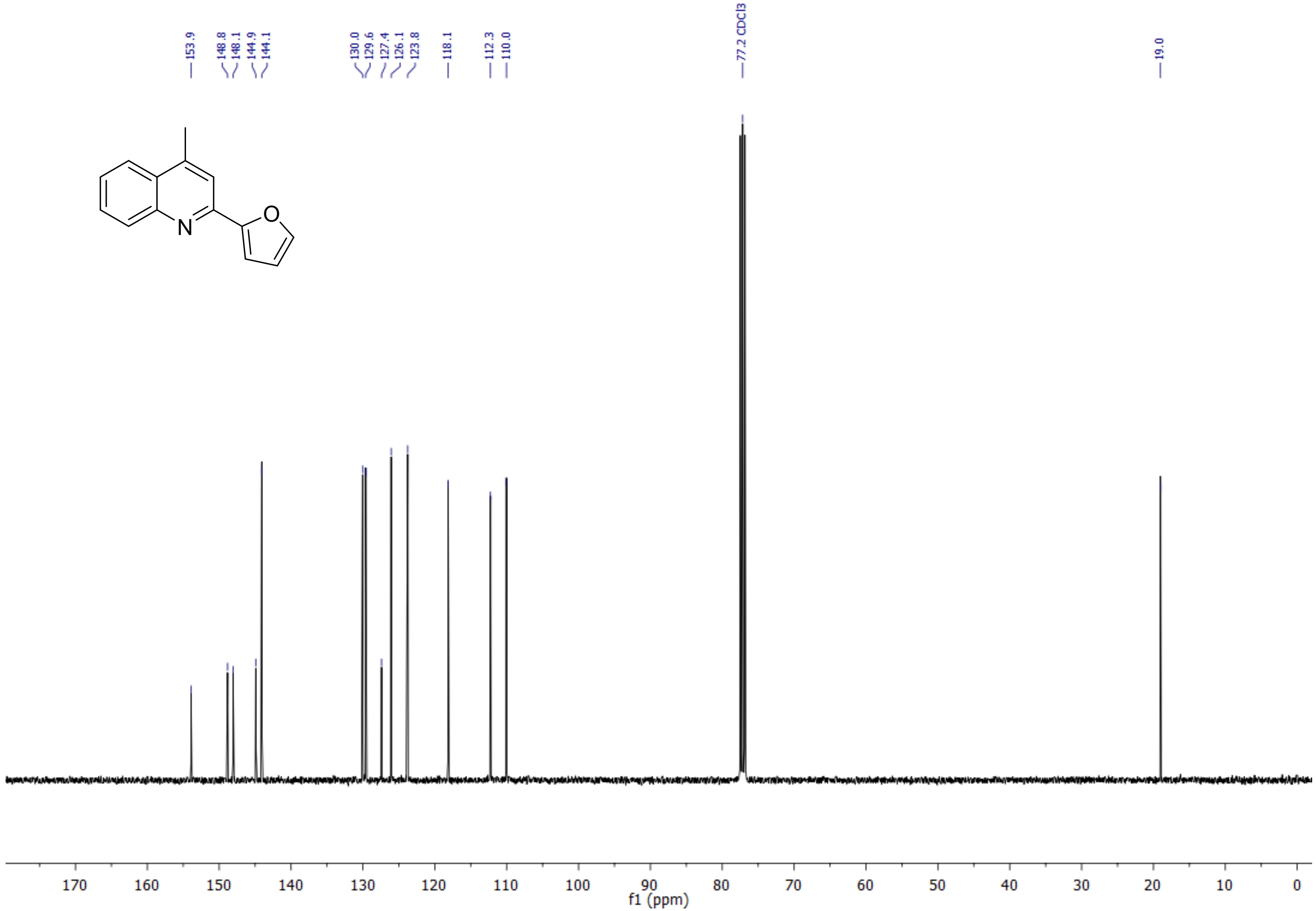

^{1}H NMR (400 MHz, $CDCl_3$) of **2l**

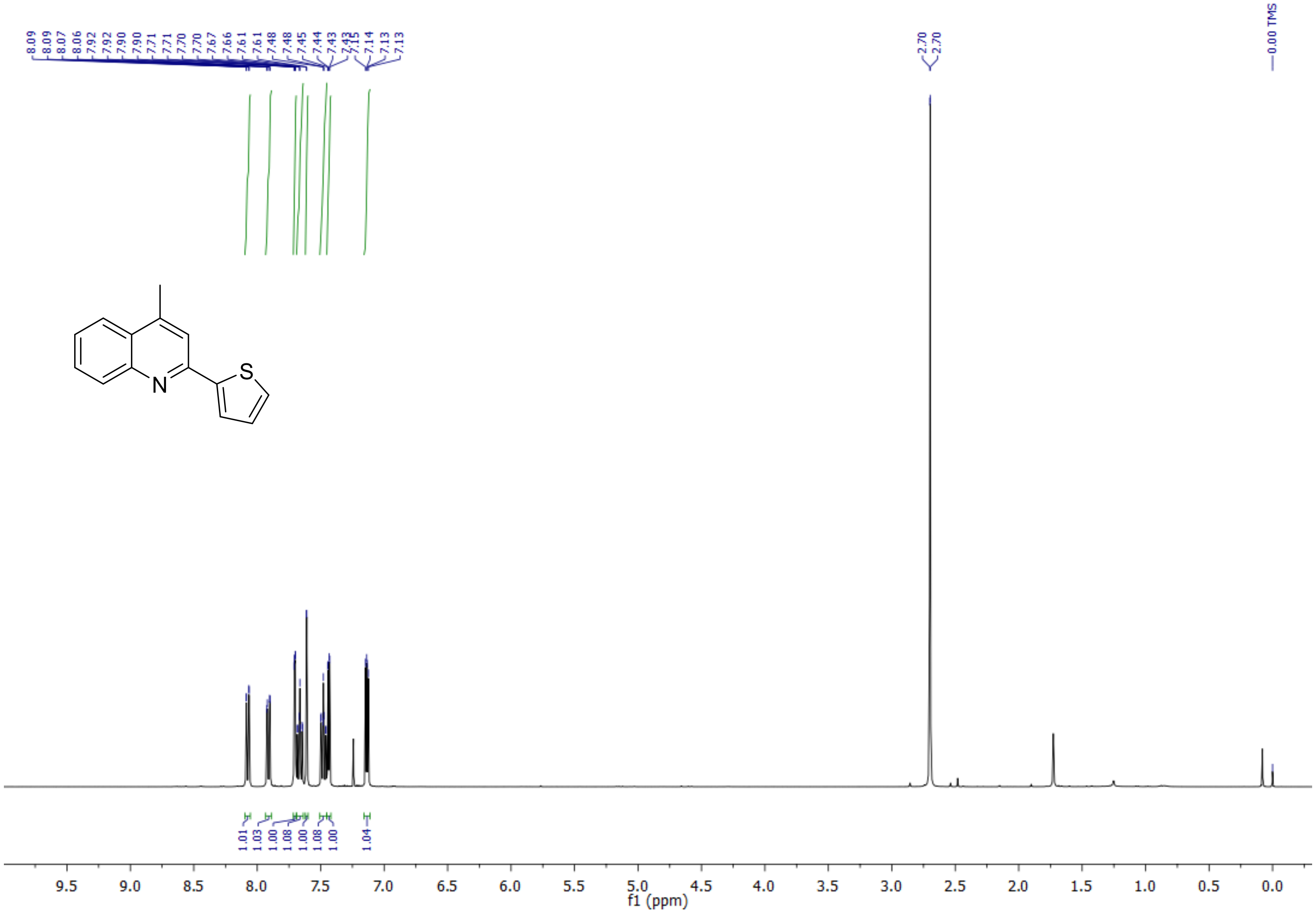

^{13}C NMR (101 MHz, $CDCl_3$) of **2l**

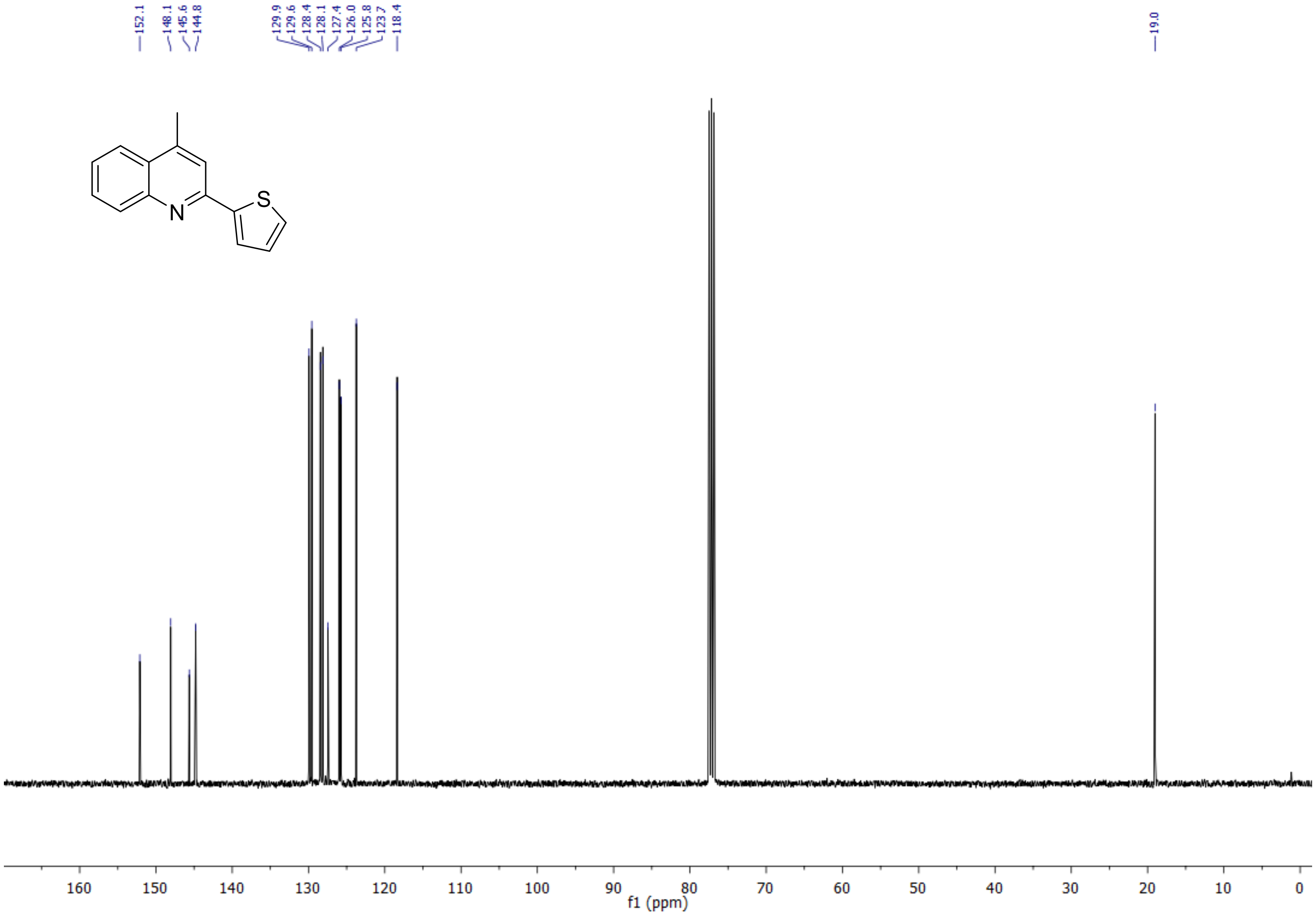

^{1}H NMR (400 MHz, acetone-d_6) of **2m**

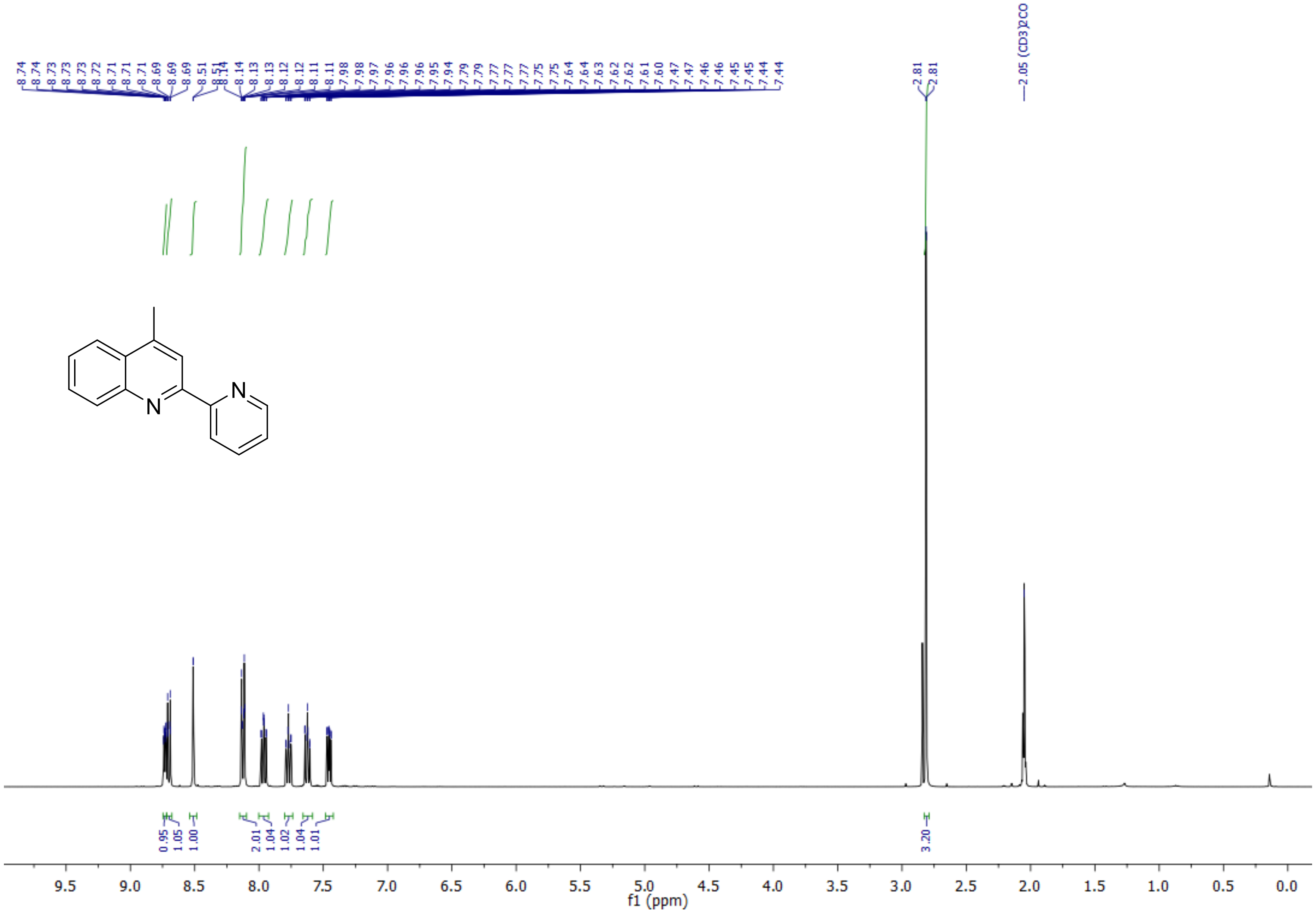

^{13}C NMR (101 MHz, acetone-d_6) of **2m**

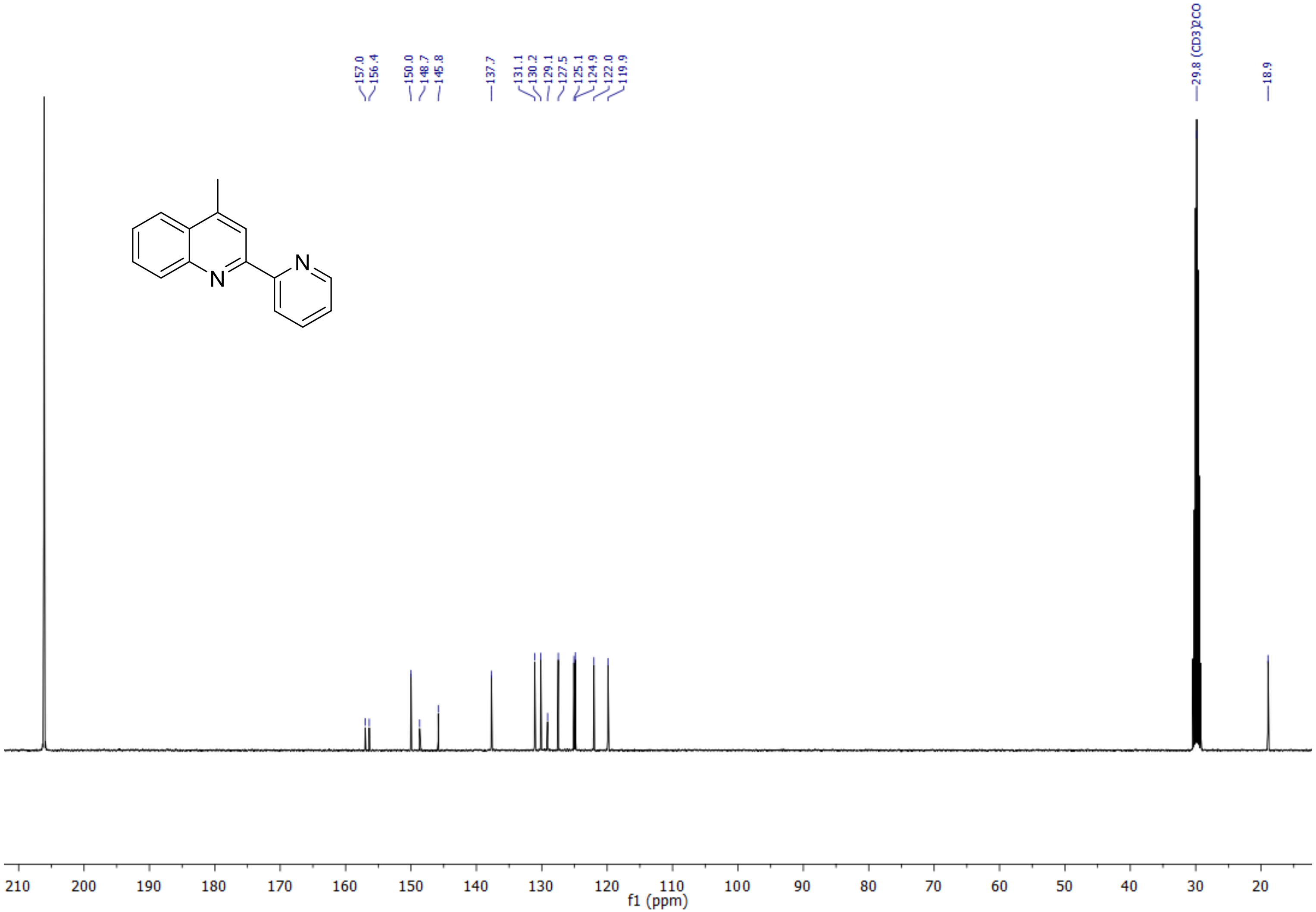

^{1}H NMR (400 MHz, $CDCl_3$) of **2n**

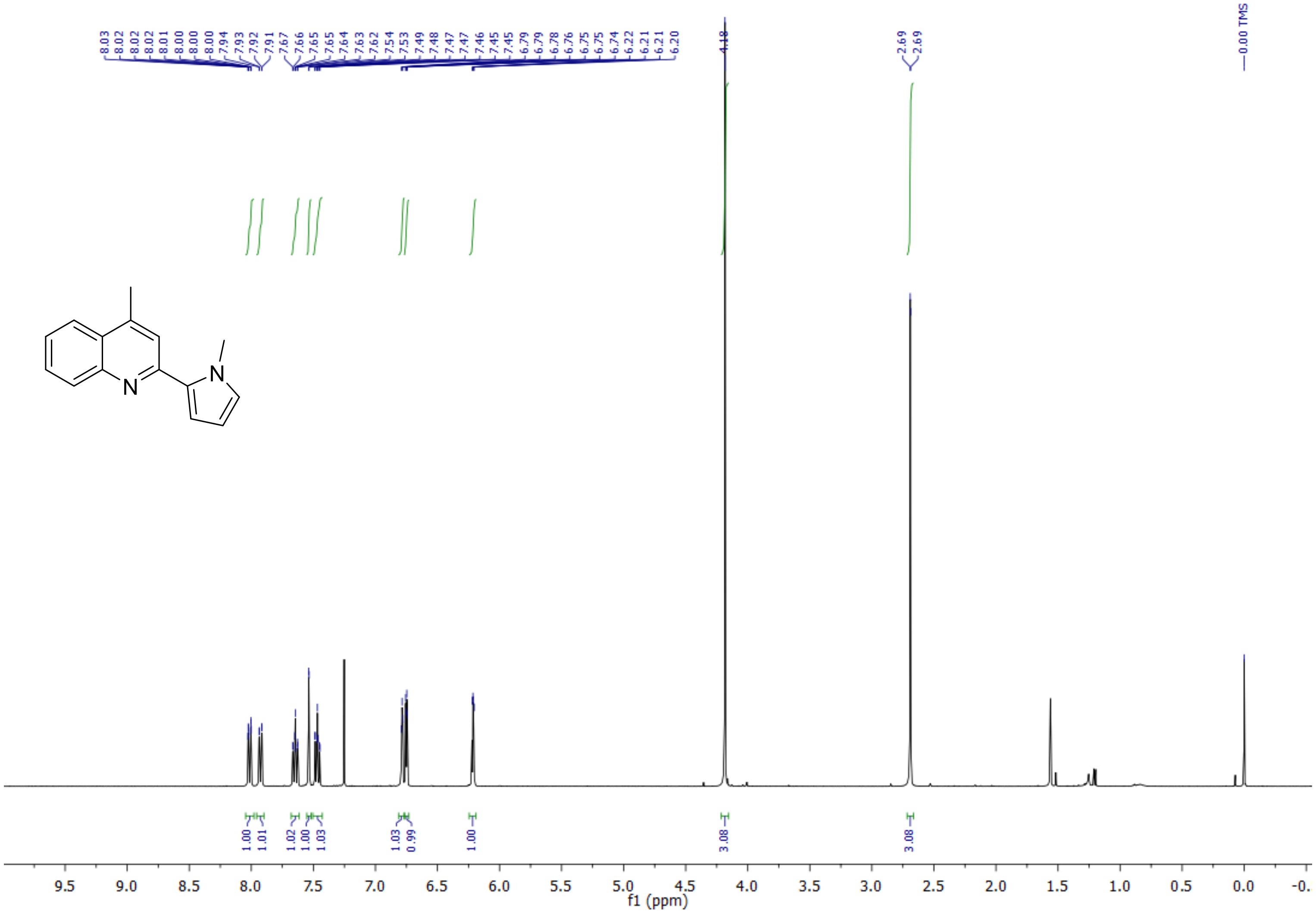

^{13}C NMR (101 MHz, $CDCl_3$) of **2n**

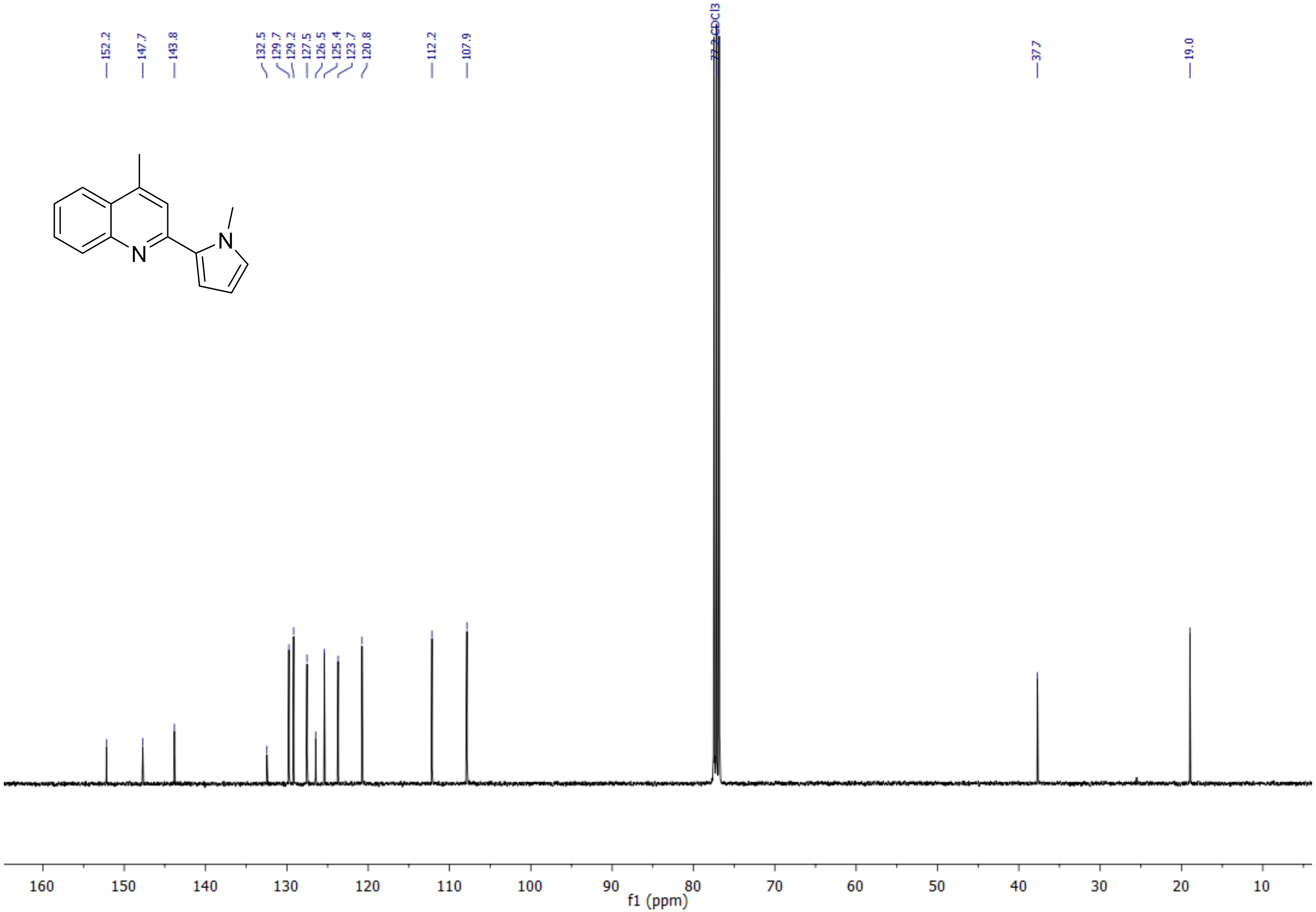

^{1}H–^{13}C HSQC-DEPT NMR (400 MHz, $CDCl_3$) of **2n**

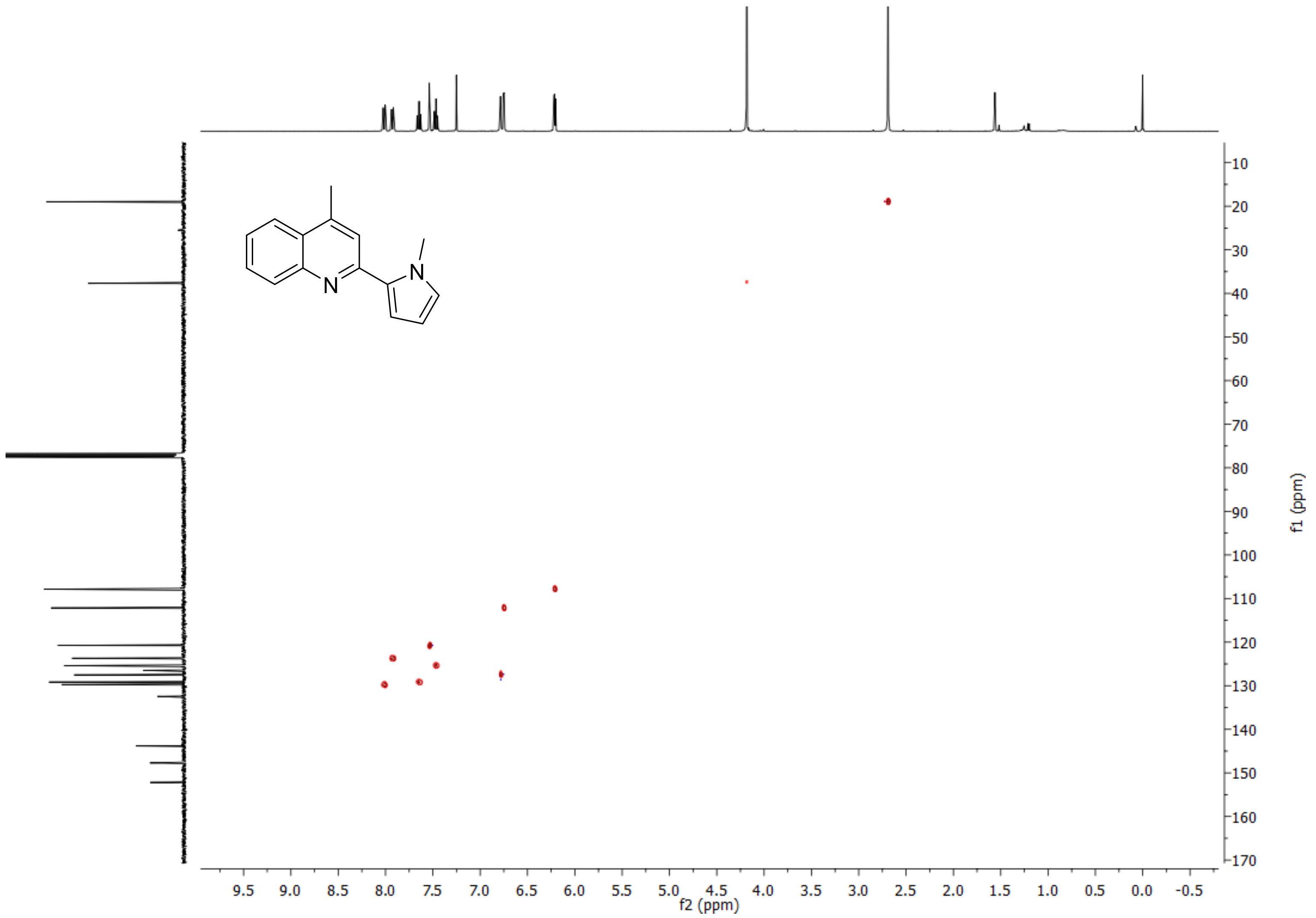

^{1}H NMR (400 MHz, acetone-d_6) of **2o**

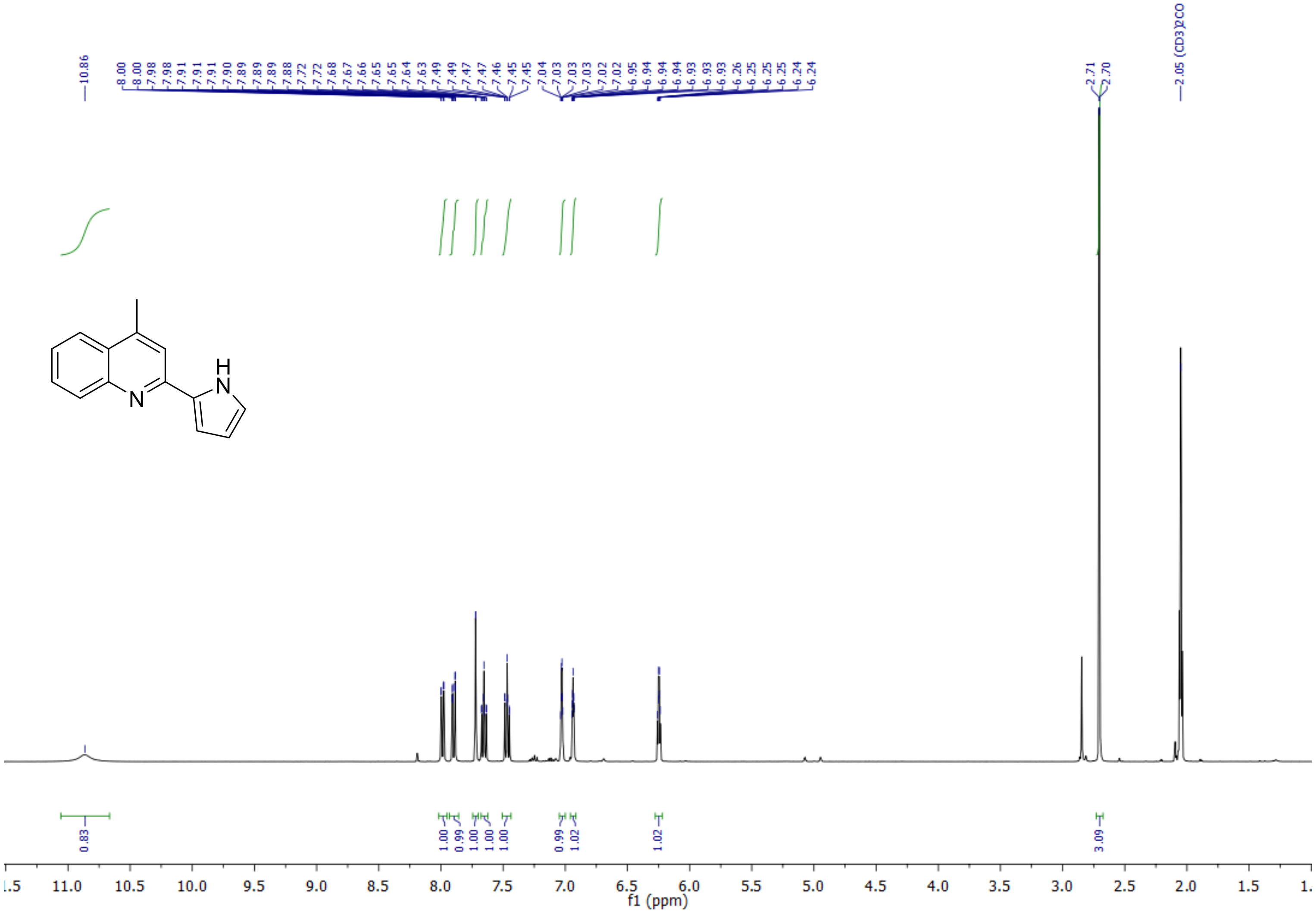

^{13}C NMR (101 MHz, acetone-d_6) of **2o**

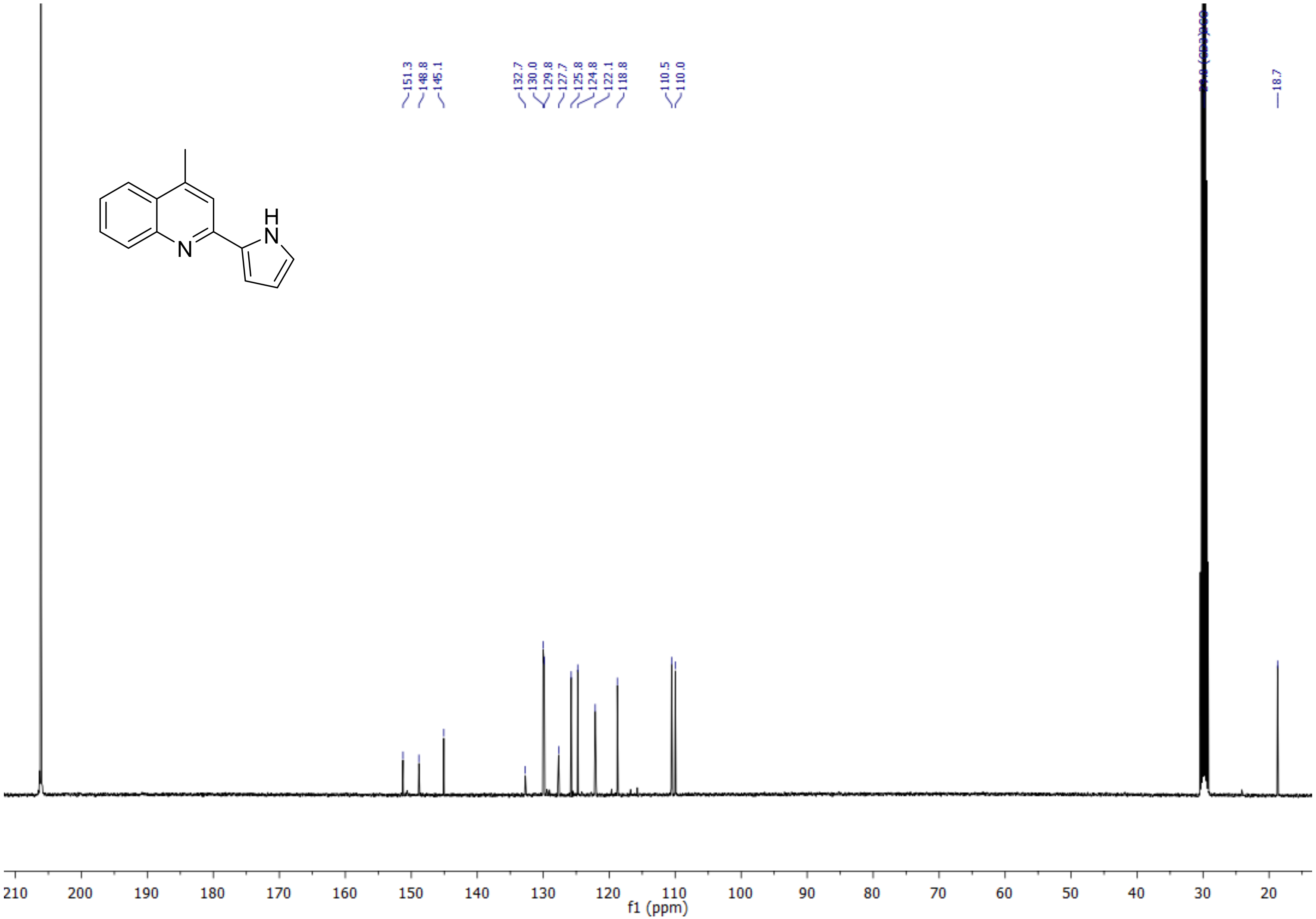

$^{1}H–^{13}C$ HSQC-DEPT NMR (400 MHz, acetone-d_6) of **2o**

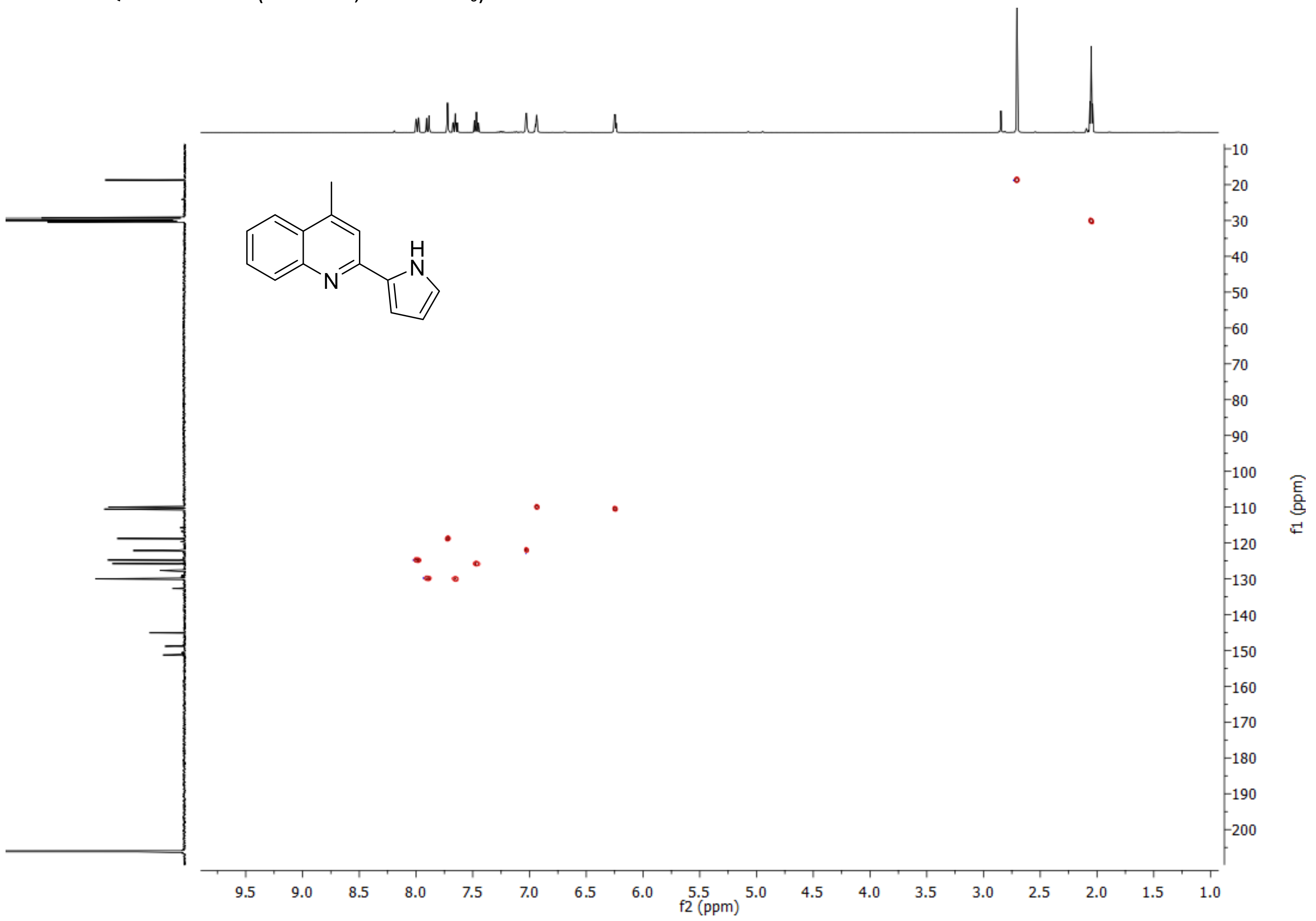

^{1}H NMR (400 MHz, acetone-d_6) of **2p**

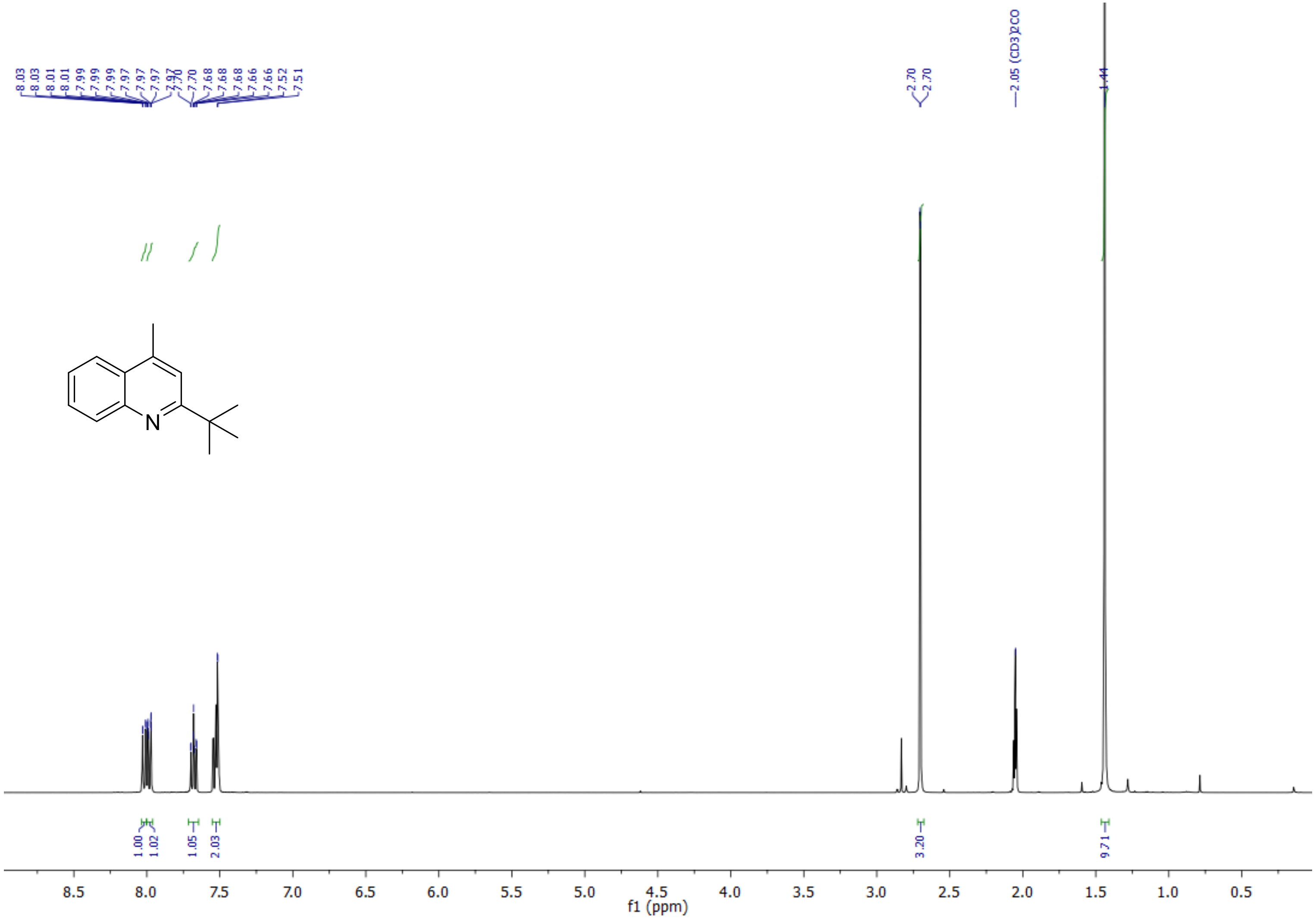

^{13}C NMR (101 MHz, acetone-d_6) of **2p**

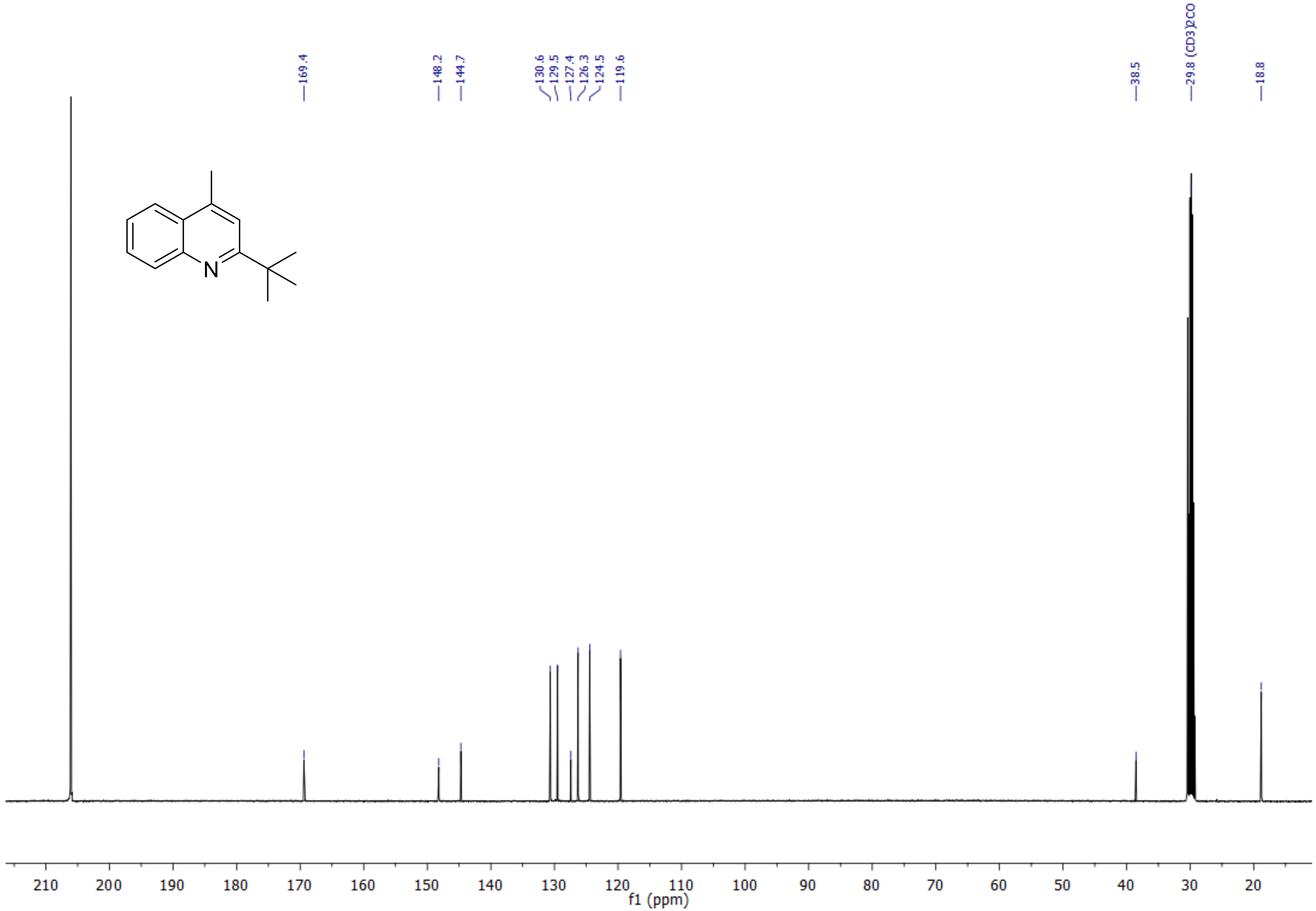

^{1}H NMR (400 MHz, acetone-d_6) of **2q**

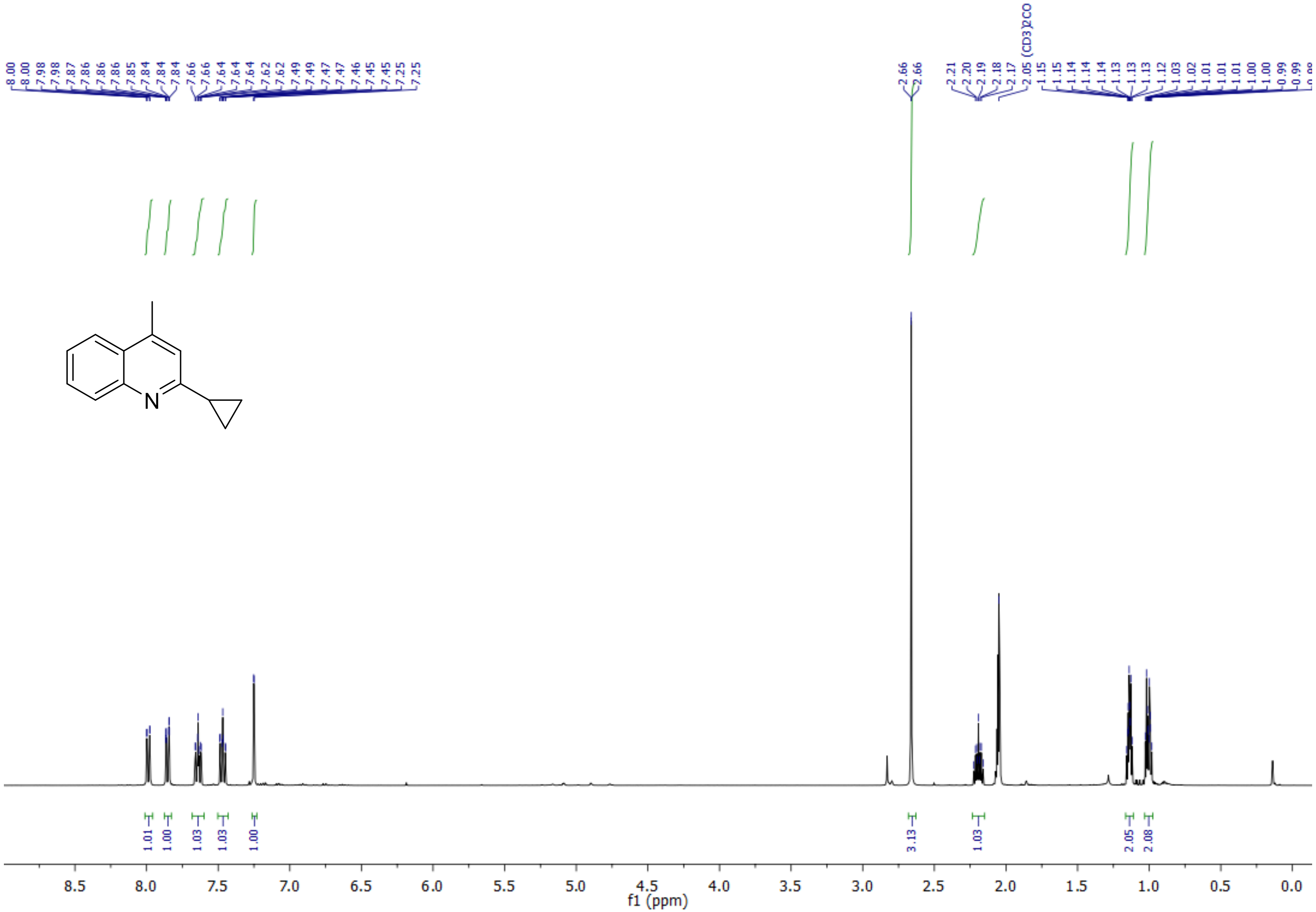

^{13}C NMR (101 MHz, acetone-d_6) of **2q**

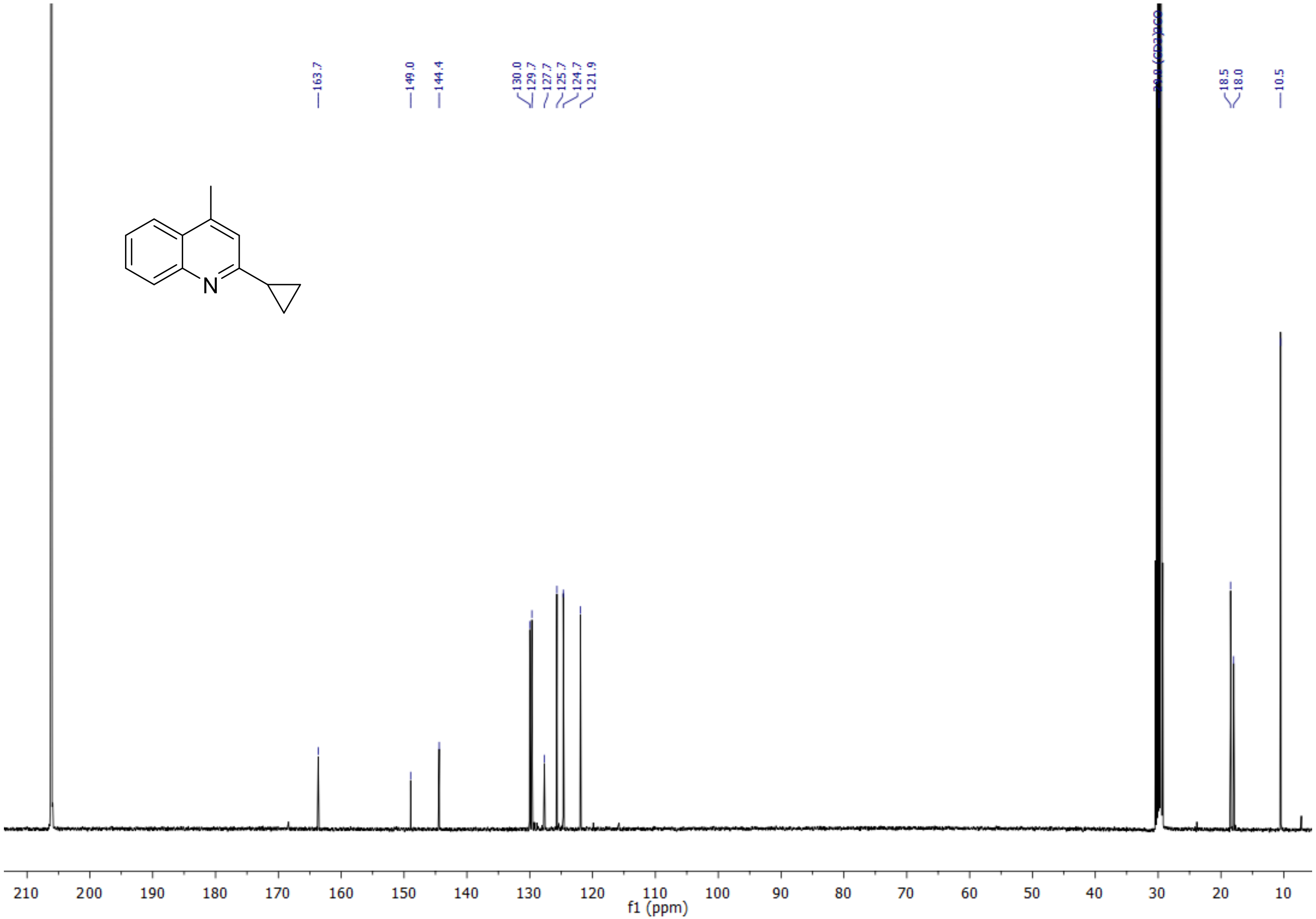

^{1}H NMR (400 MHz, acetone-d_6) of **2r**

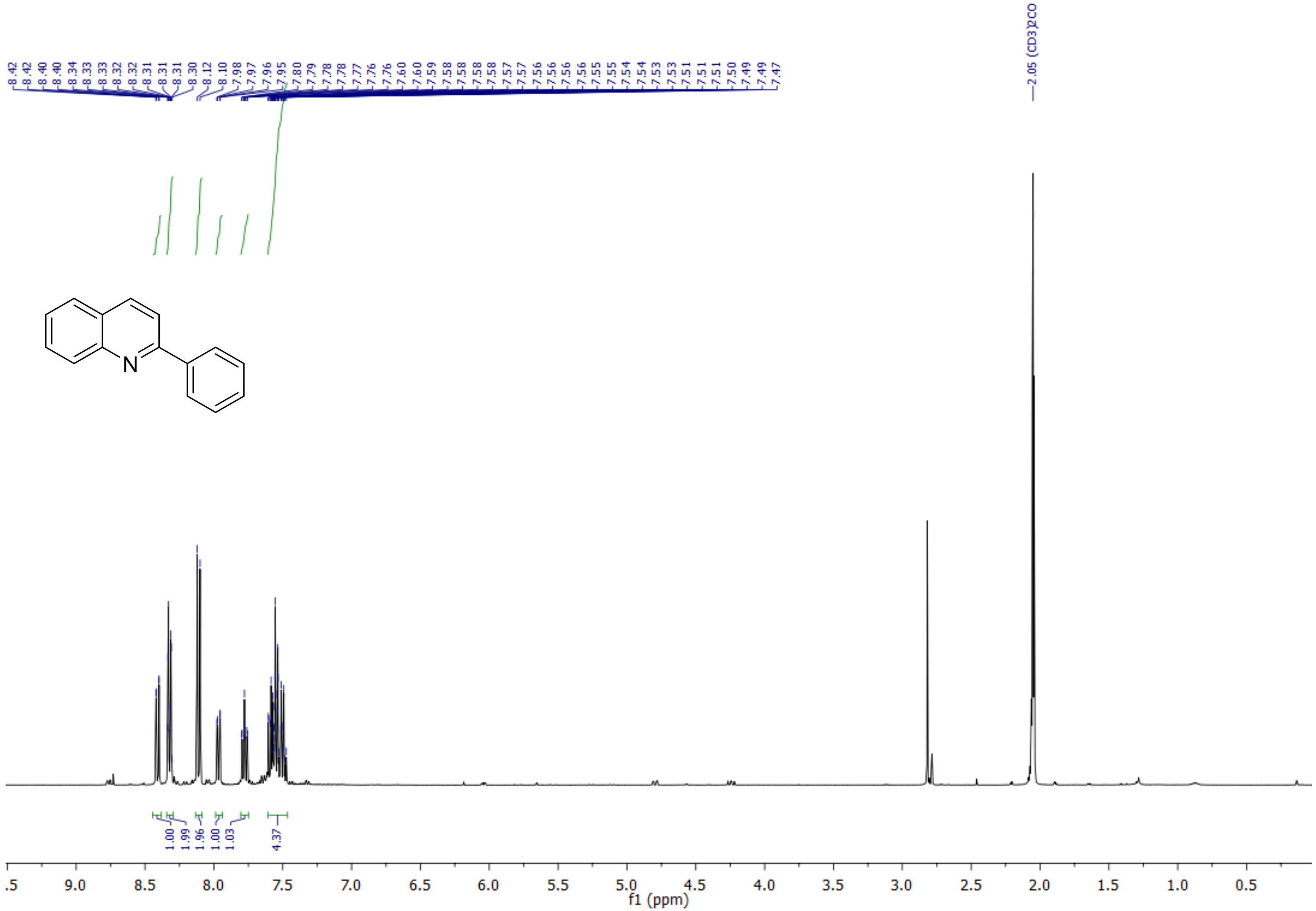

^{13}C NMR (101 MHz, acetone-d_6) of **2r**

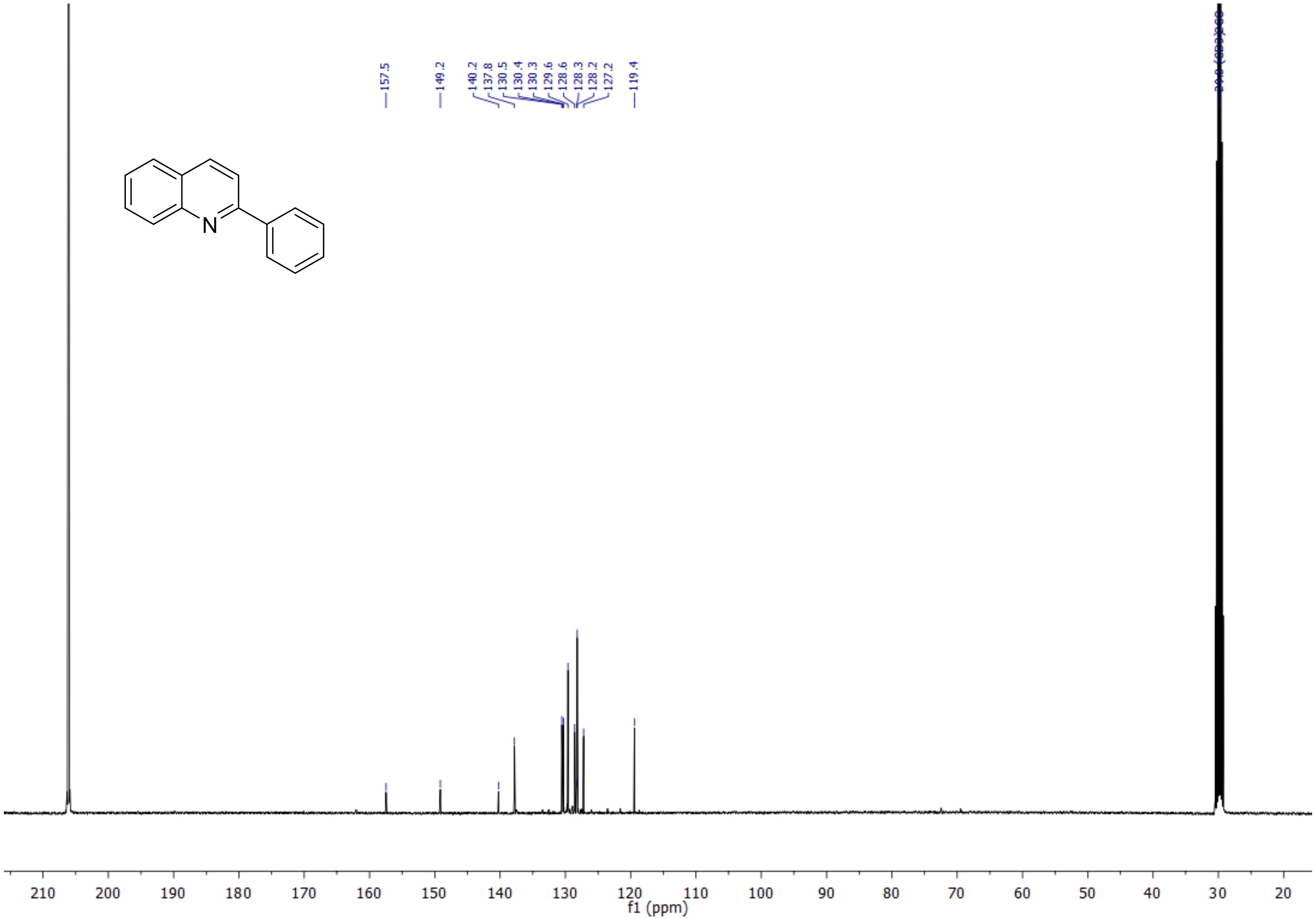

^{1}H NMR (400 MHz, acetone-d_6) of **2s**

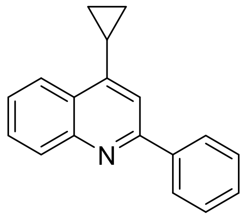

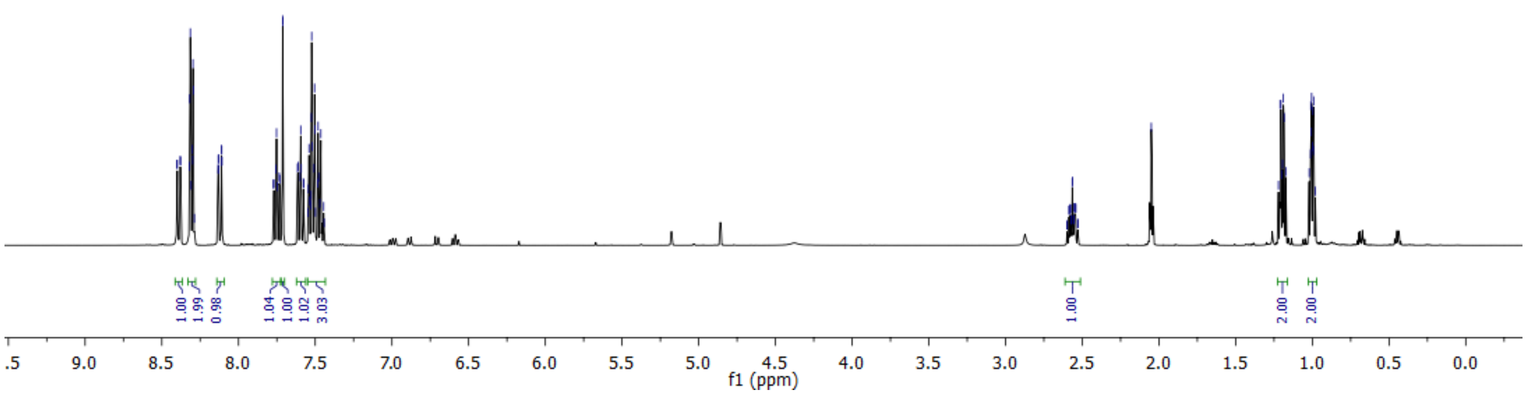

^{13}C NMR (101 MHz, acetone-d_6) of **2s**

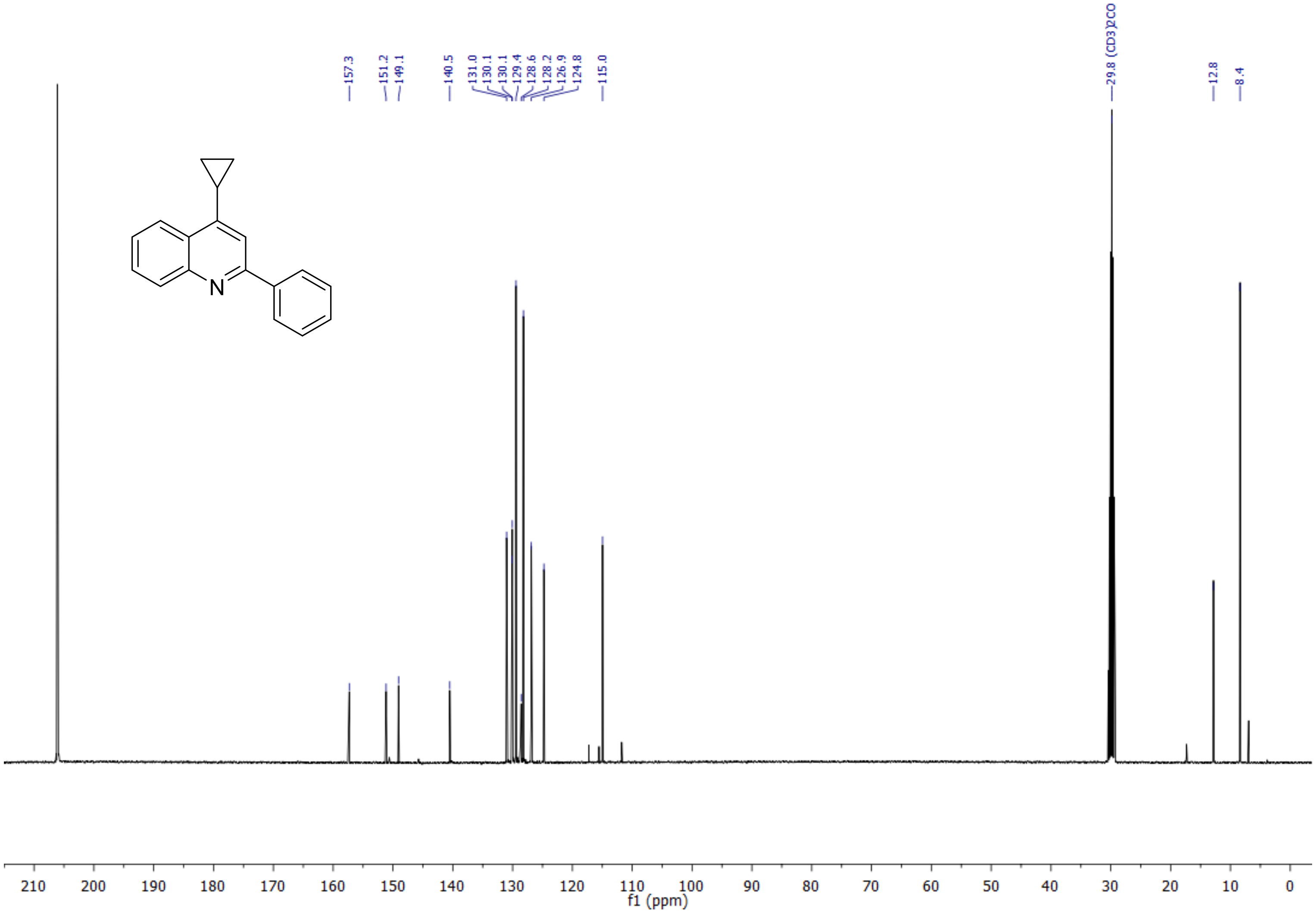

^{1}H NMR (400 MHz, $CDCl_3$) of **2t**

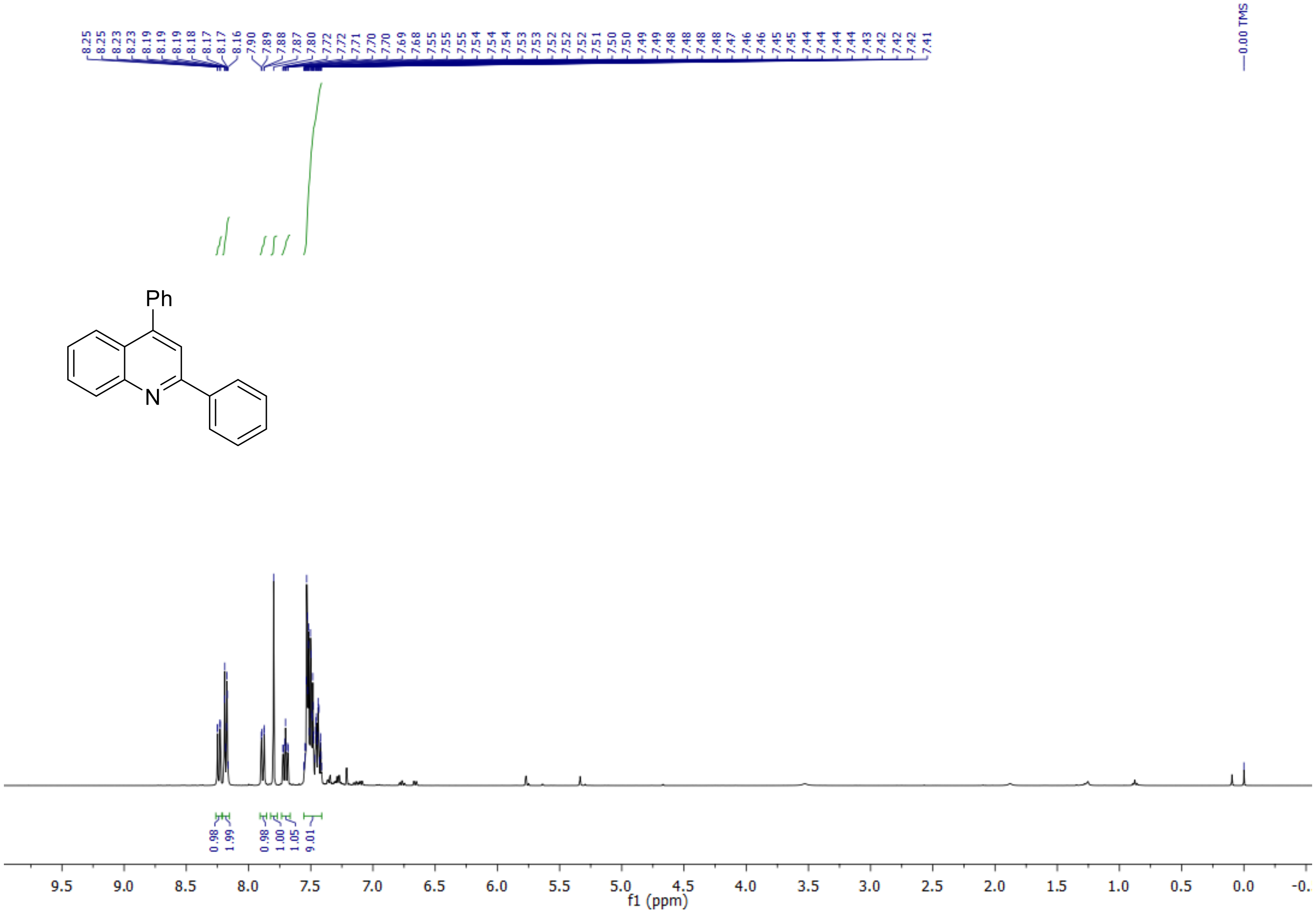

^{13}C NMR (101 MHz, $CDCl_3$) of **2t**

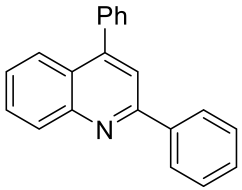

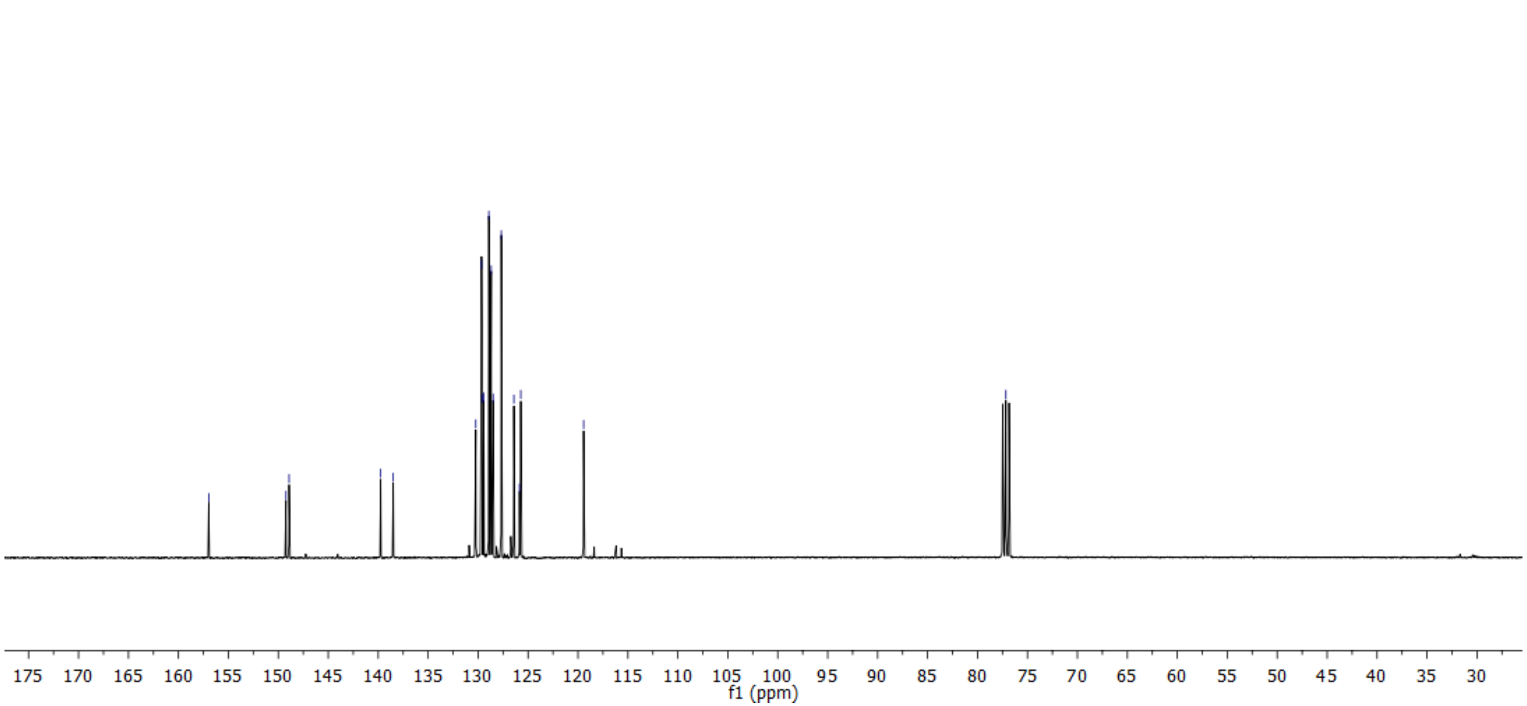

^{1}H NMR (400 MHz, acetone-d_6) of **2u**

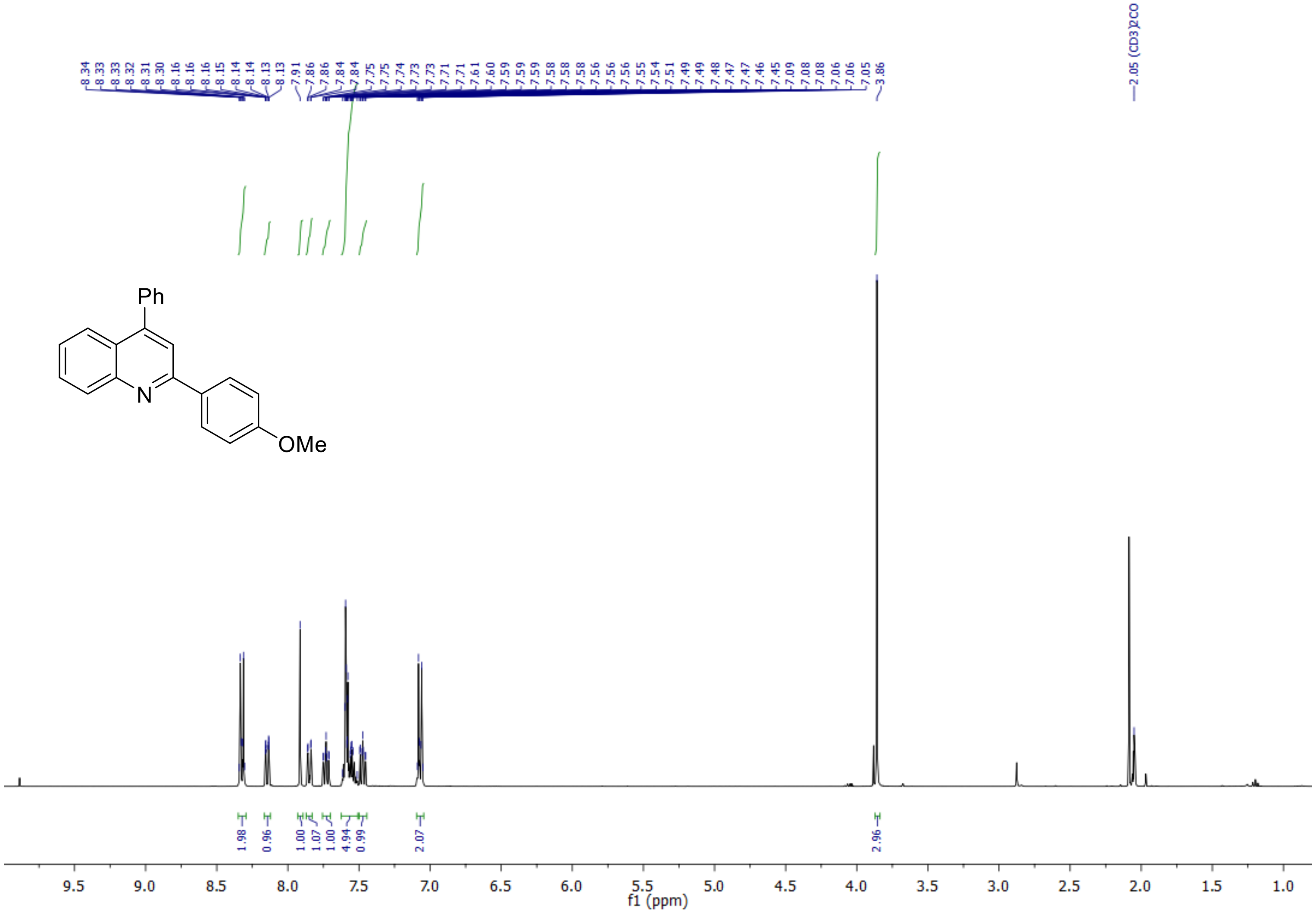

^{13}C NMR (101 MHz, acetone-d_6) of **2u**

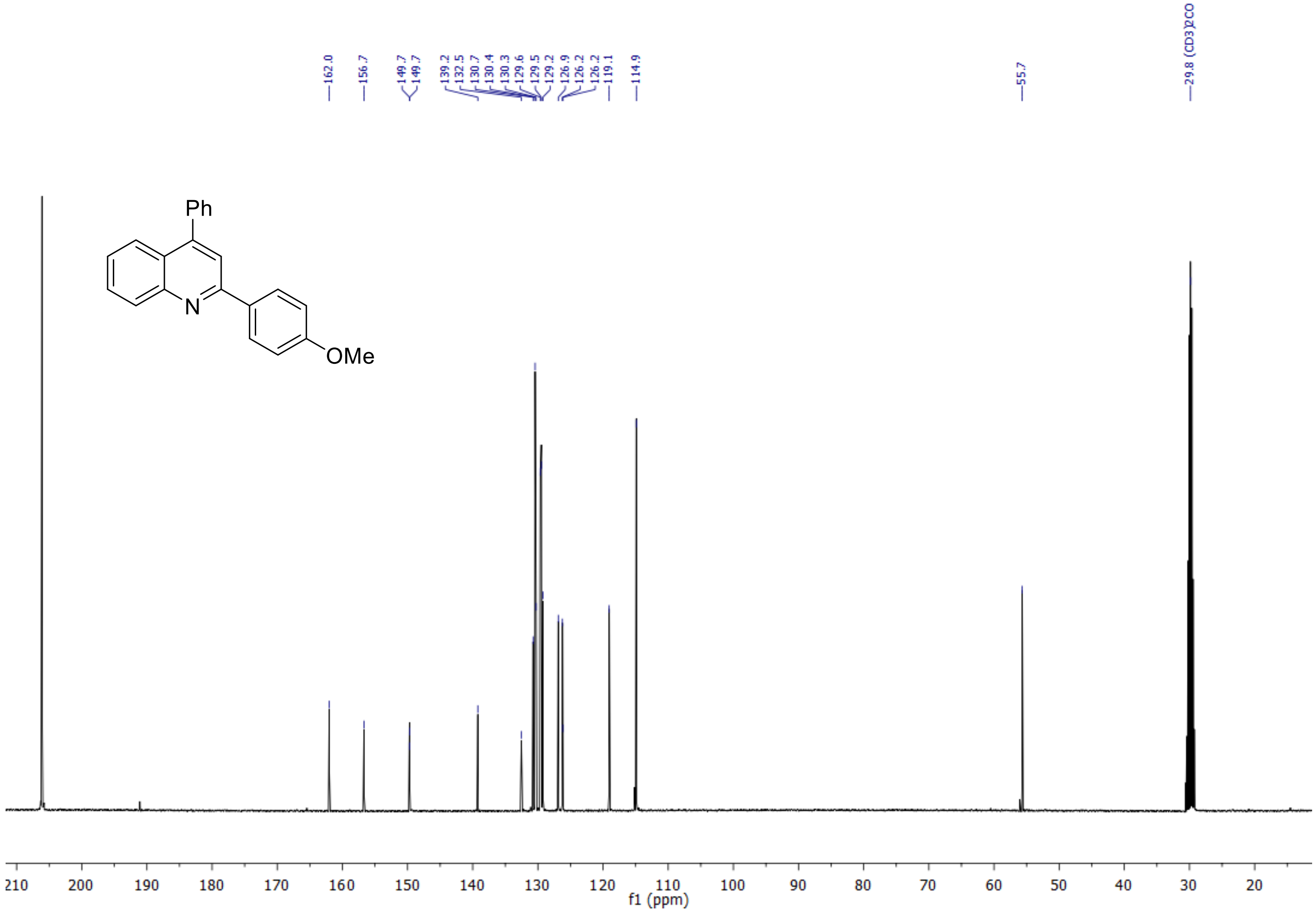

^{1}H NMR (400 MHz, $CDCl_3$) of **2v**

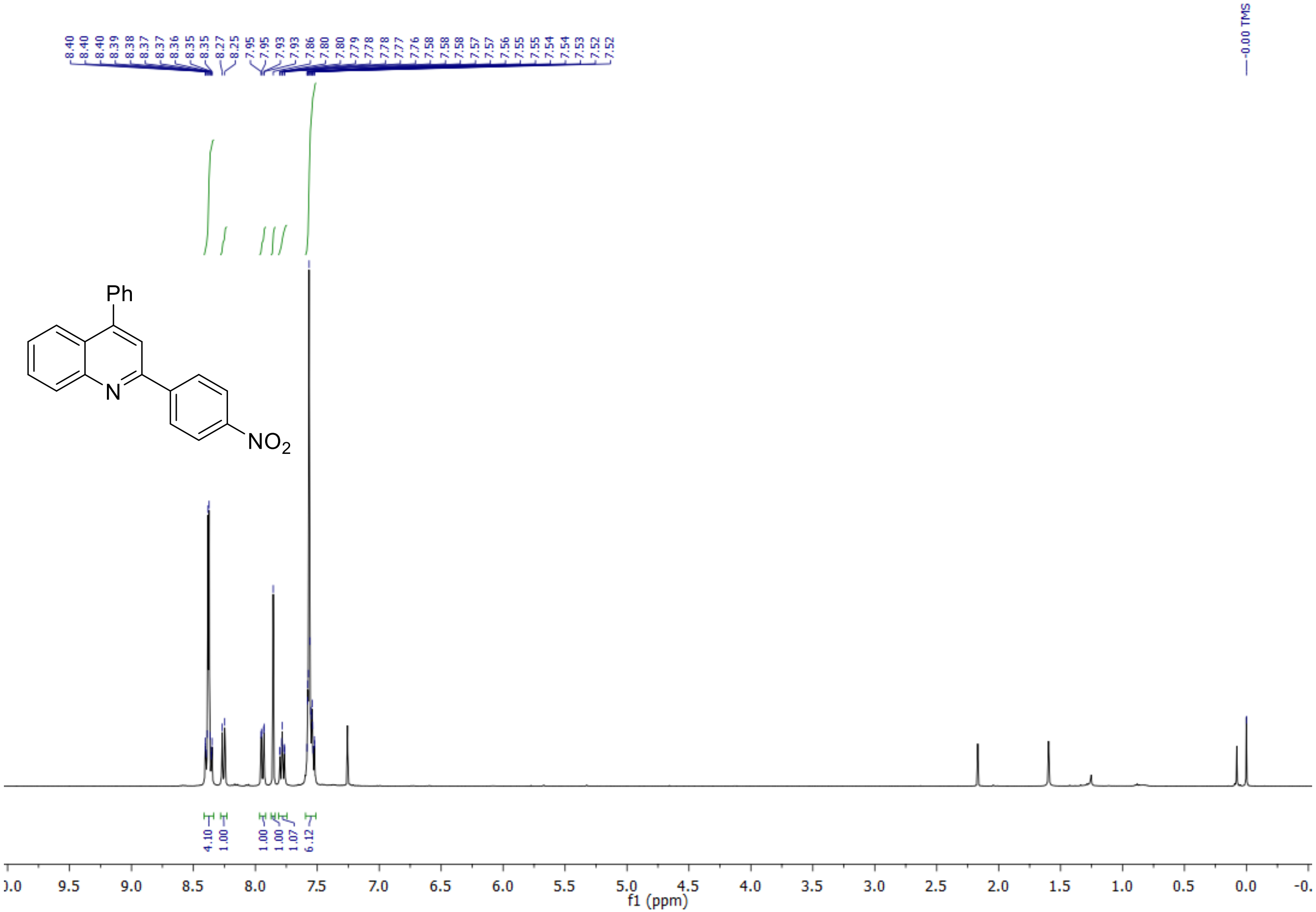

^{13}C NMR (101 MHz, $CDCl_3$) of **2v**

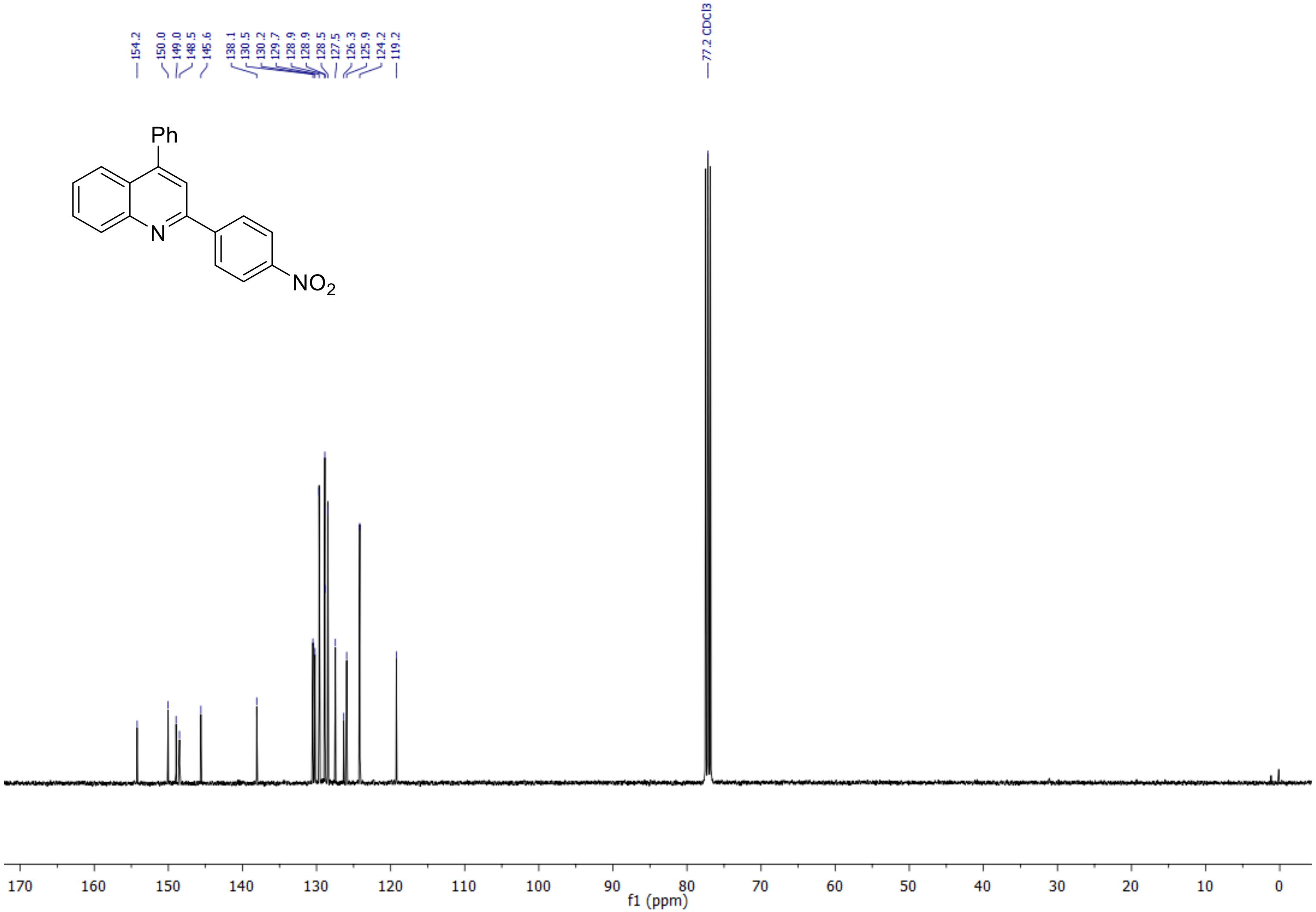

^{1}H NMR (400 MHz, $CDCl_3$) of **2w**

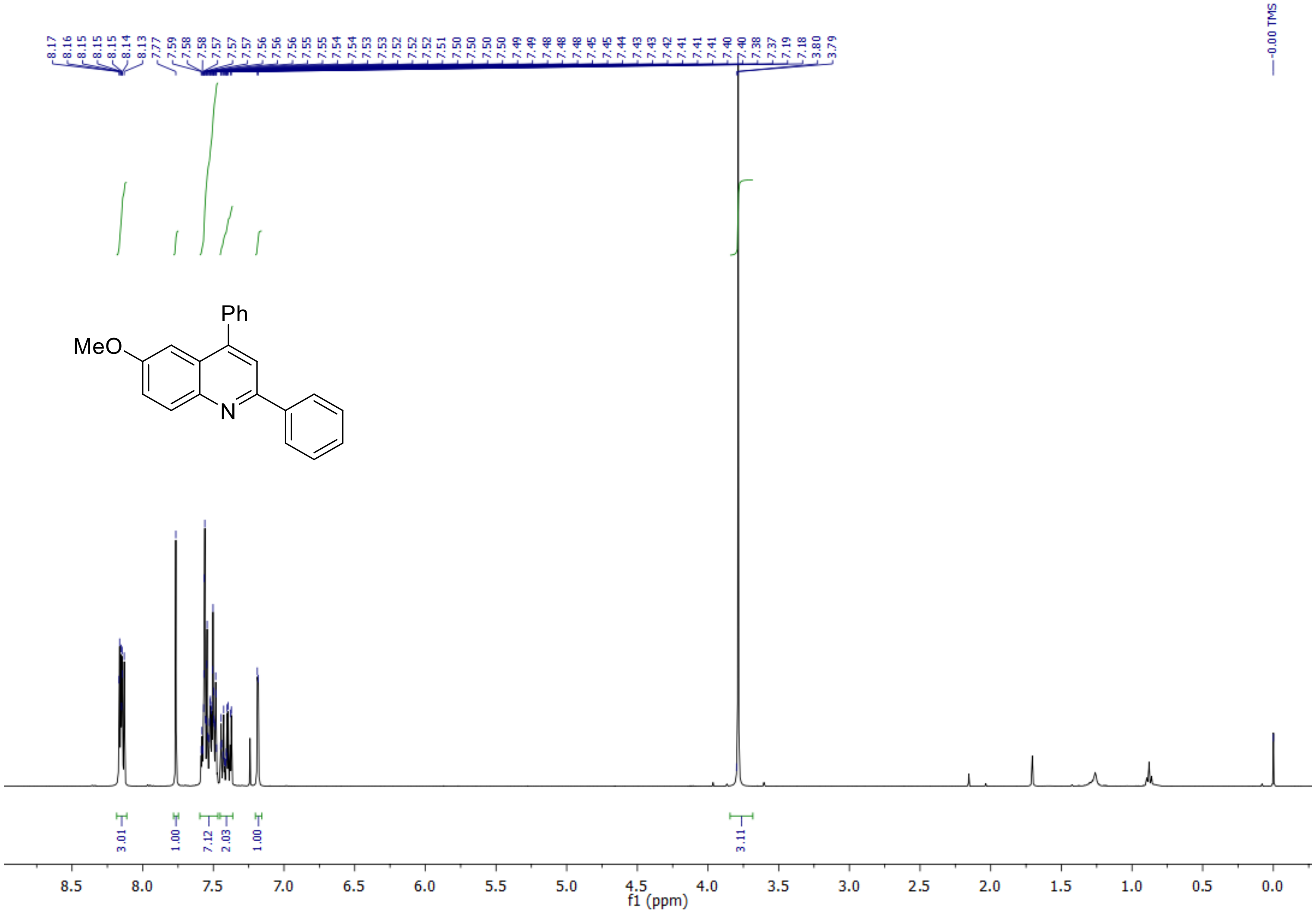

^{13}C NMR (101 MHz, $CDCl_3$) of **2w**

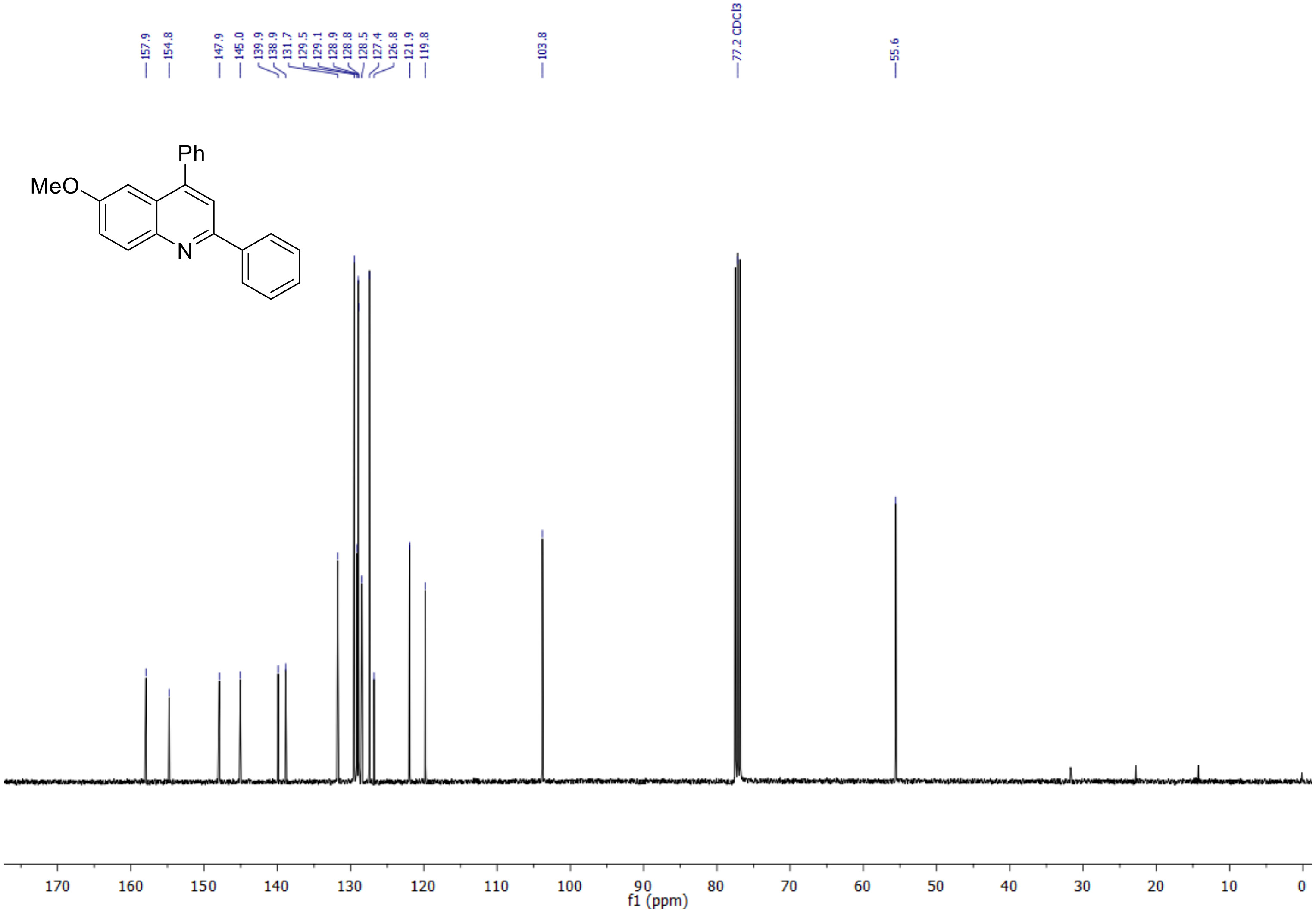

^{1}H NMR (400 MHz, $CDCl_3$) of **2x**

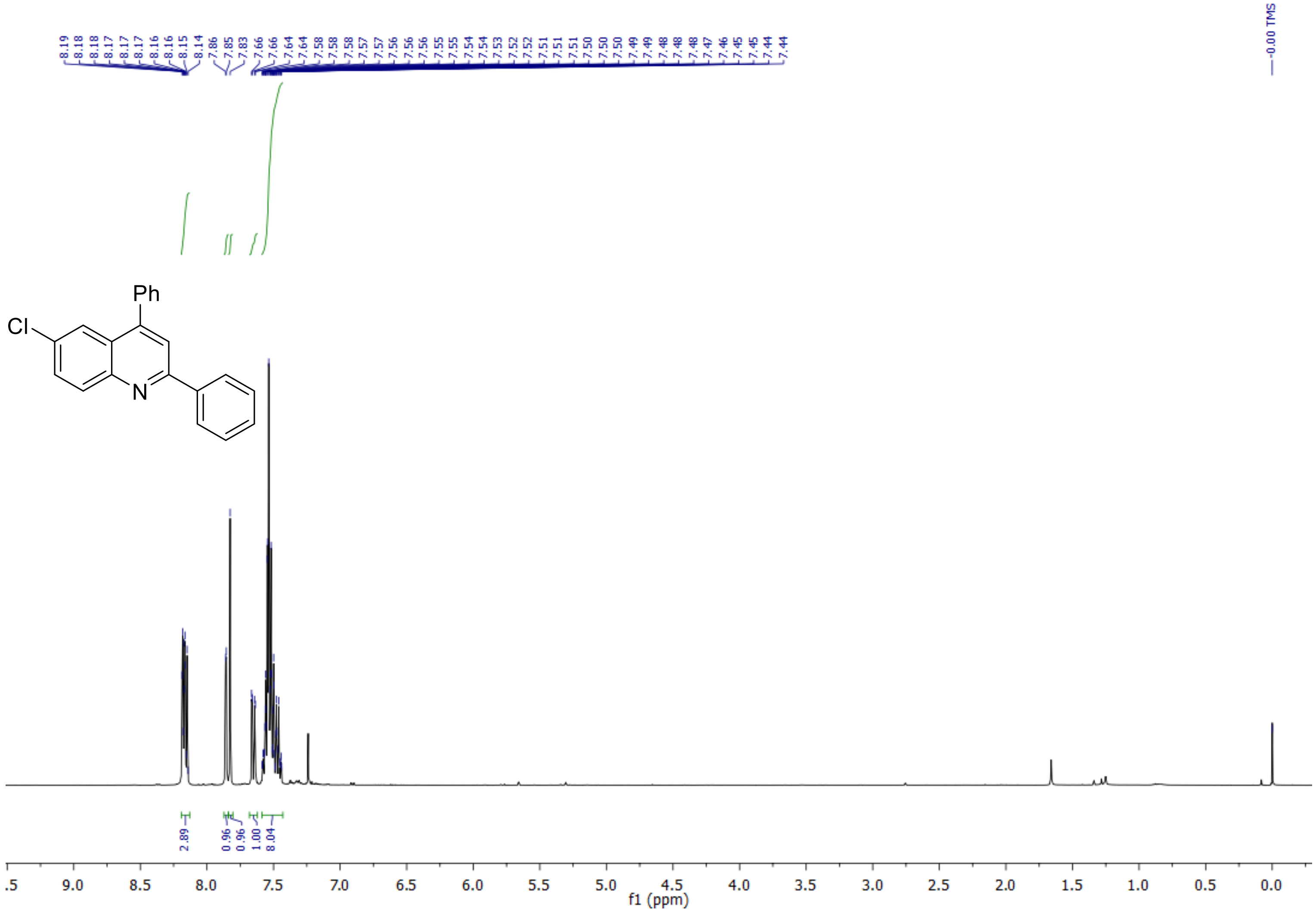

^{13}C NMR (101 MHz, $CDCl_3$) of **2x**

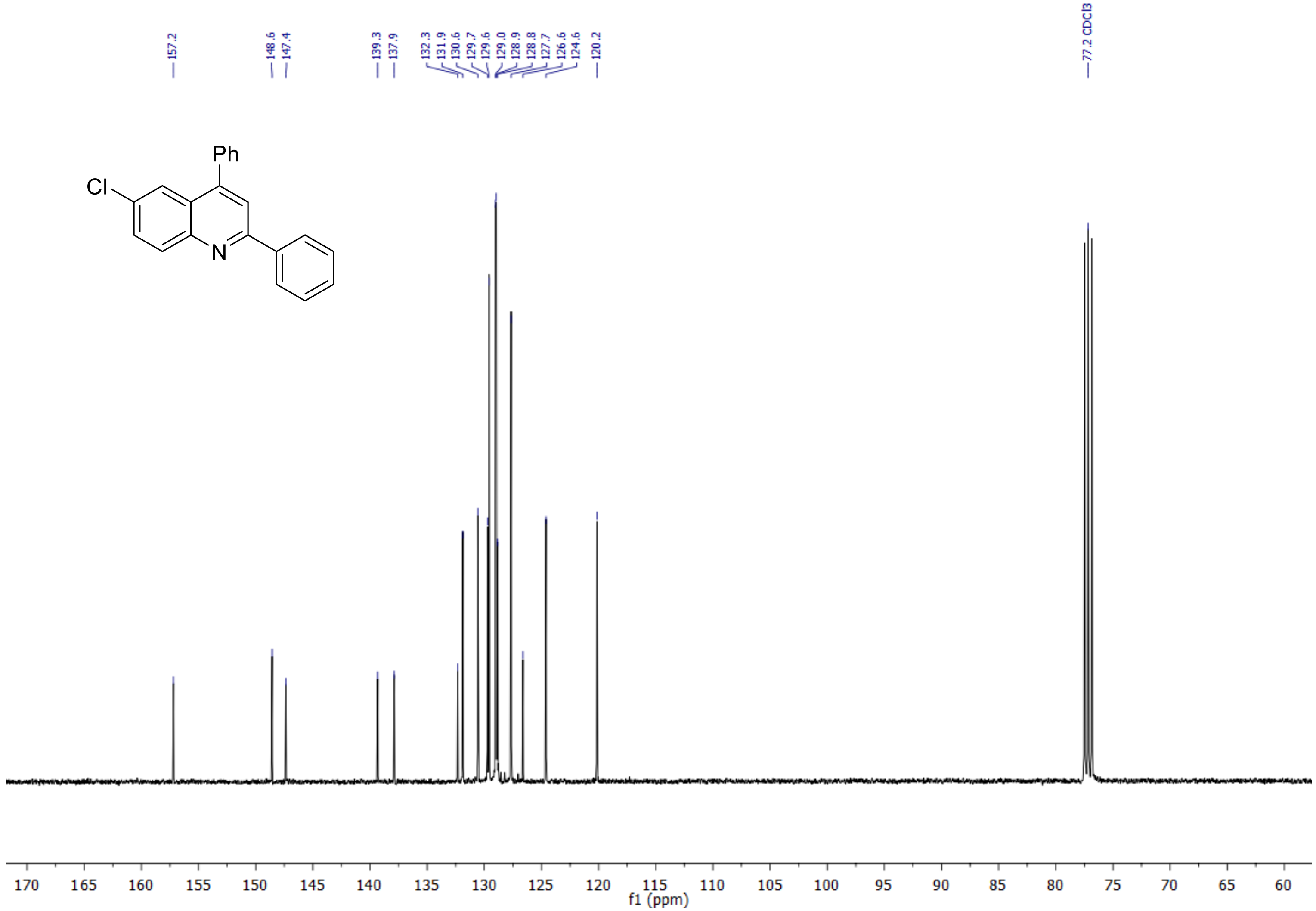

^{1}H NMR (400 MHz, $CDCl_3$) of **2y**

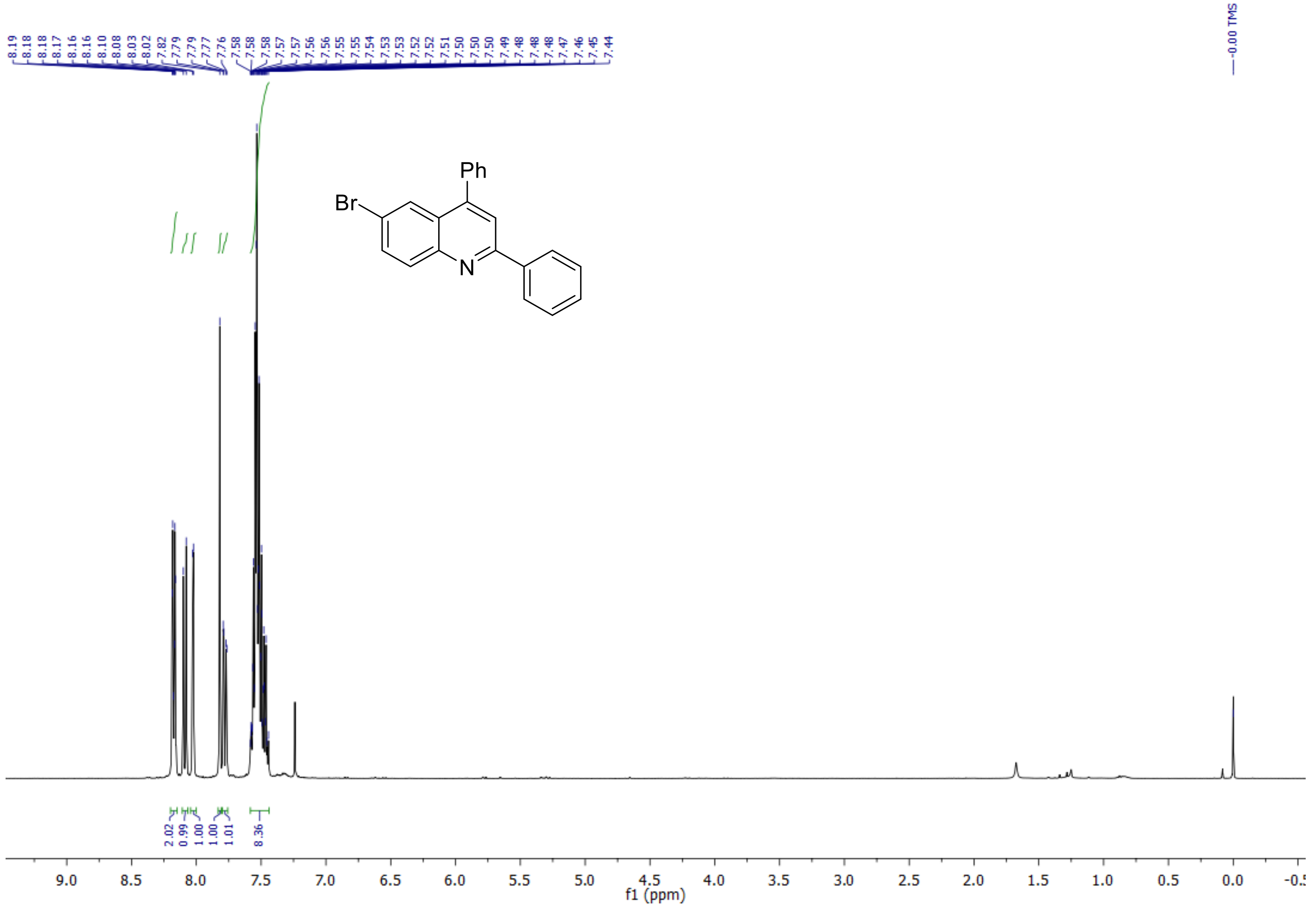

^{13}C NMR (101 MHz, $CDCl_3$) of **2y**

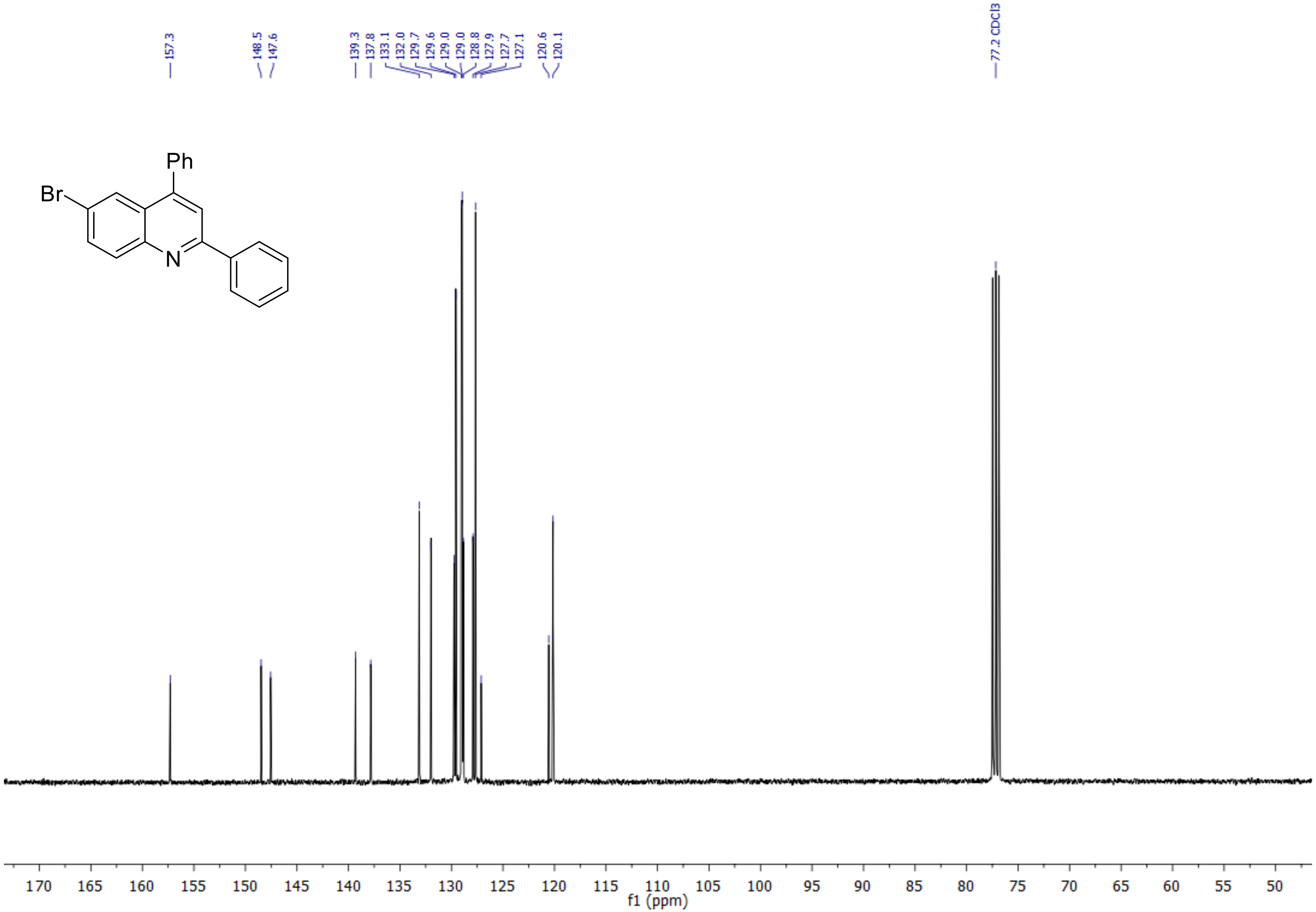

^{1}H NMR (400 MHz, acetone-d_6) of **2z**

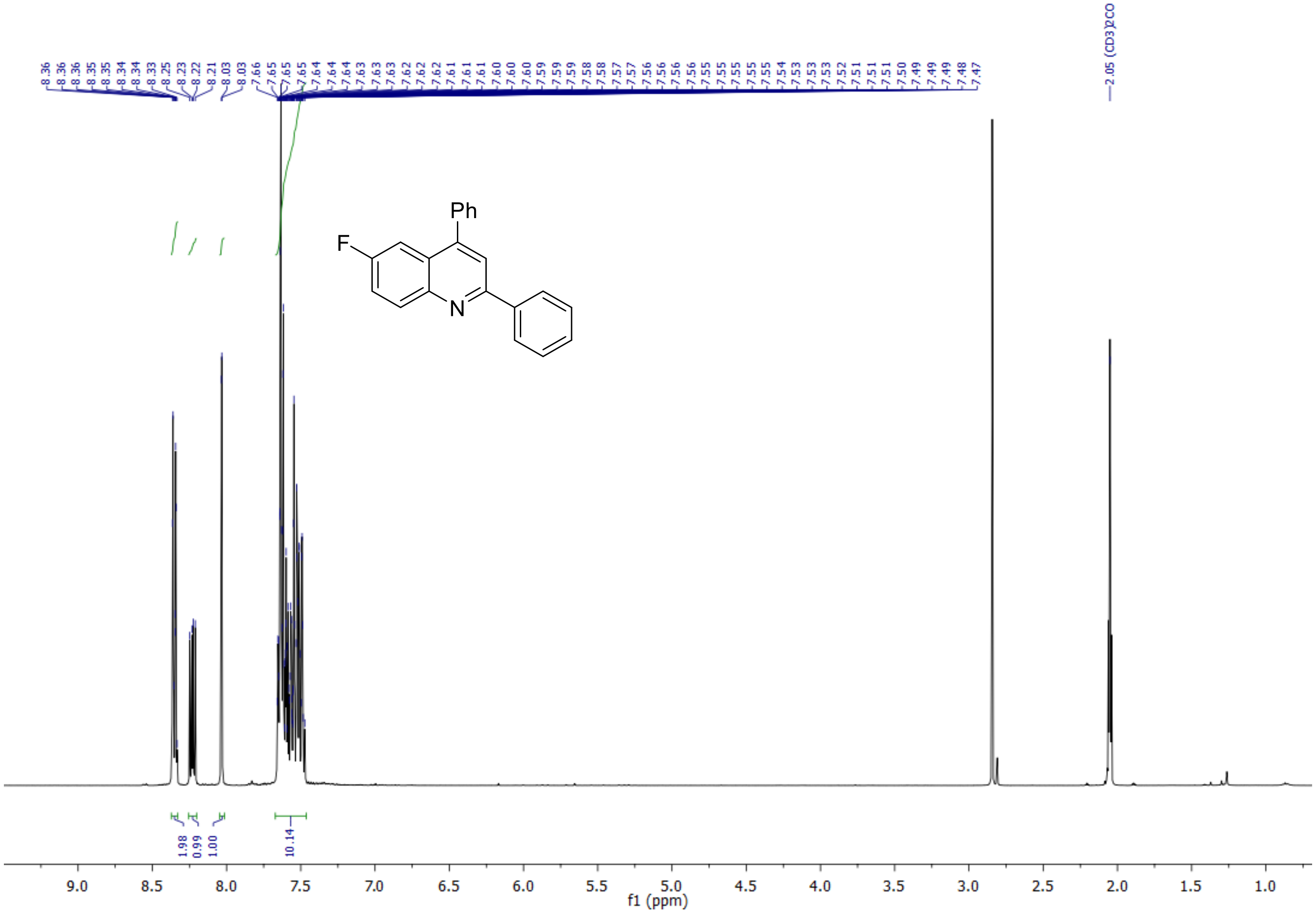

^{13}C NMR (101 MHz, acetone-d_6) of **2z**

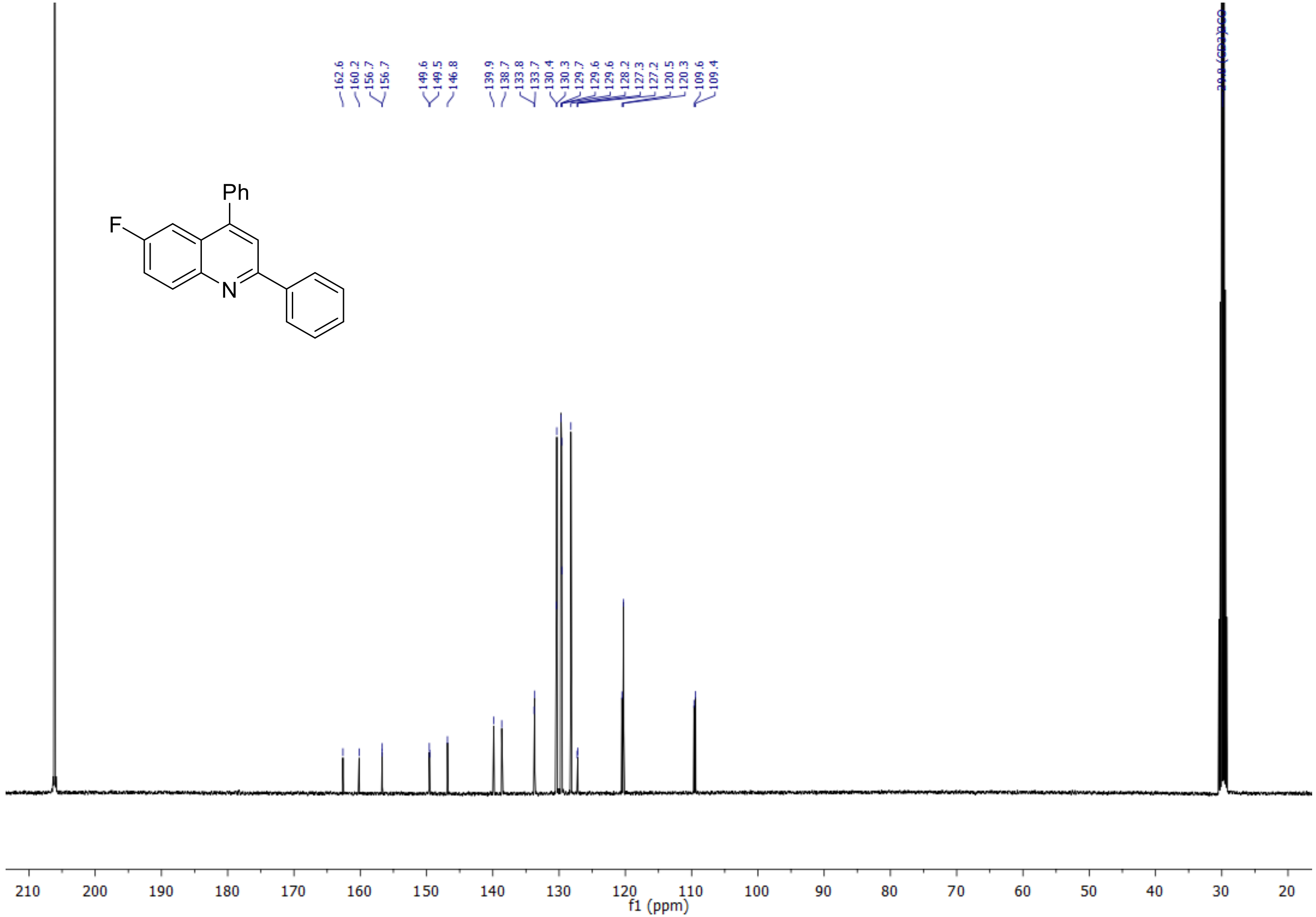

^{19}F NMR (377 MHz, acetone-d_6) of **2z**

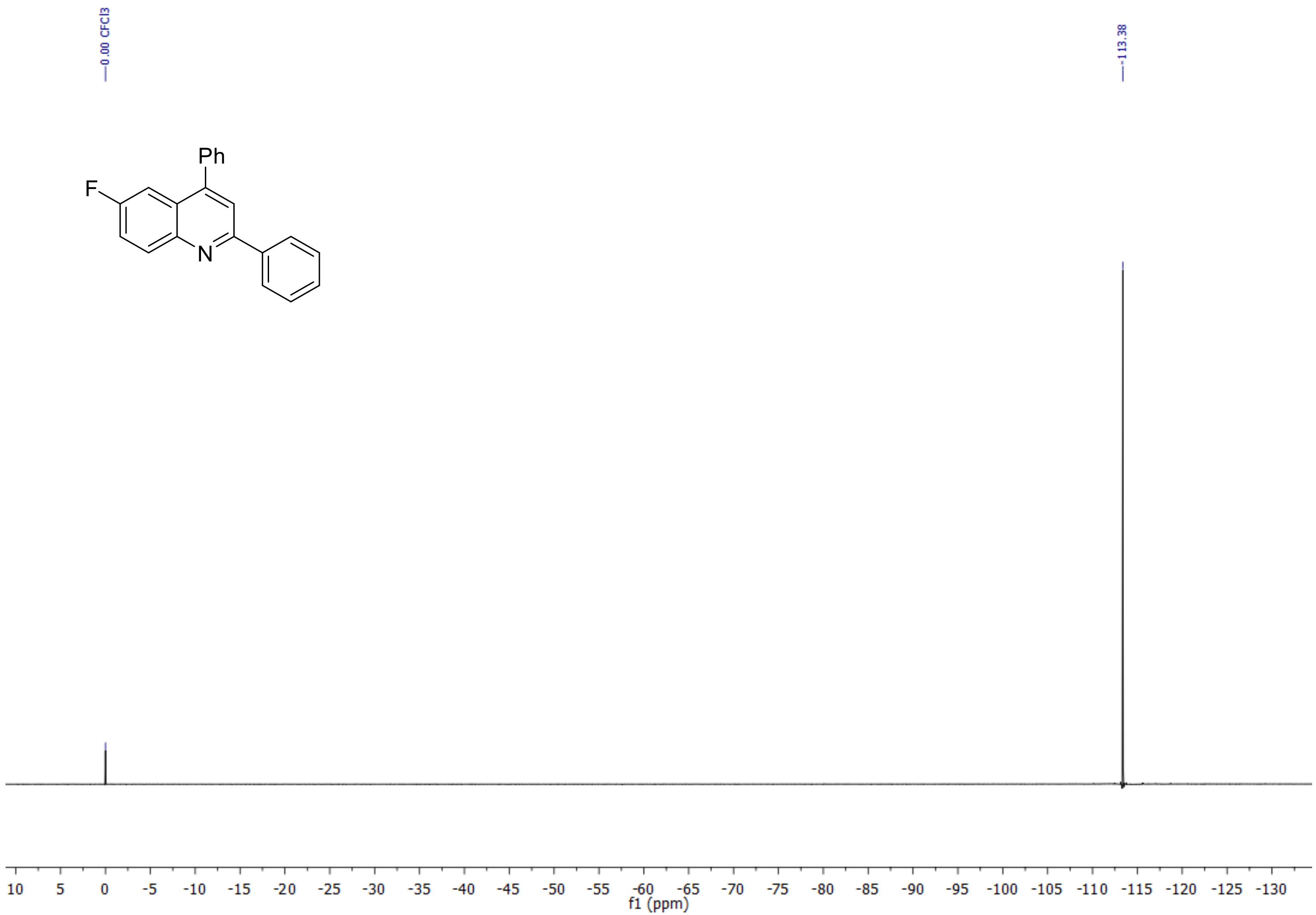

^1H NMR (400 MHz, $CDCl_3$) of **3**

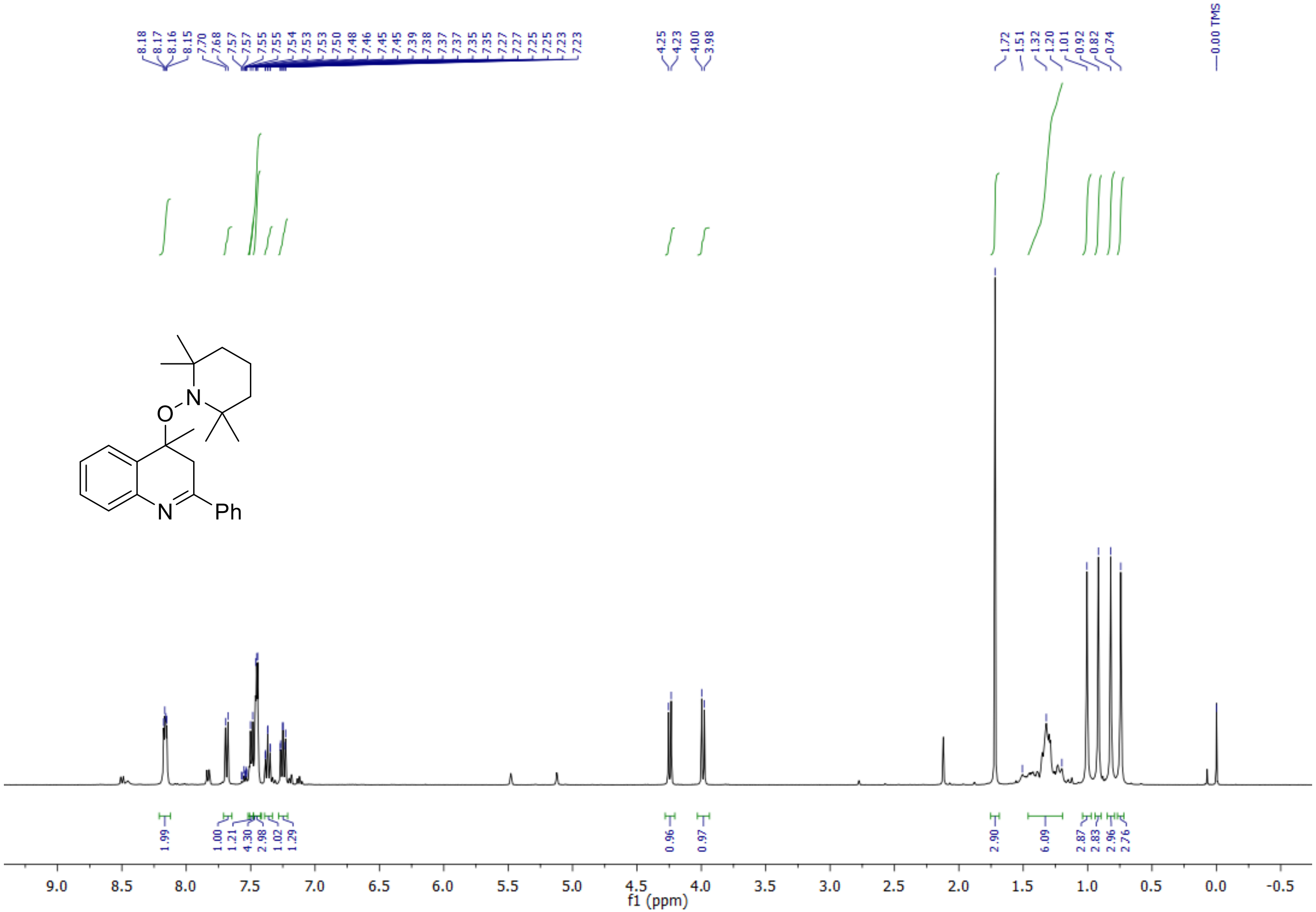

^{13}C NMR (101 MHz, $CDCl_3$) of **3**

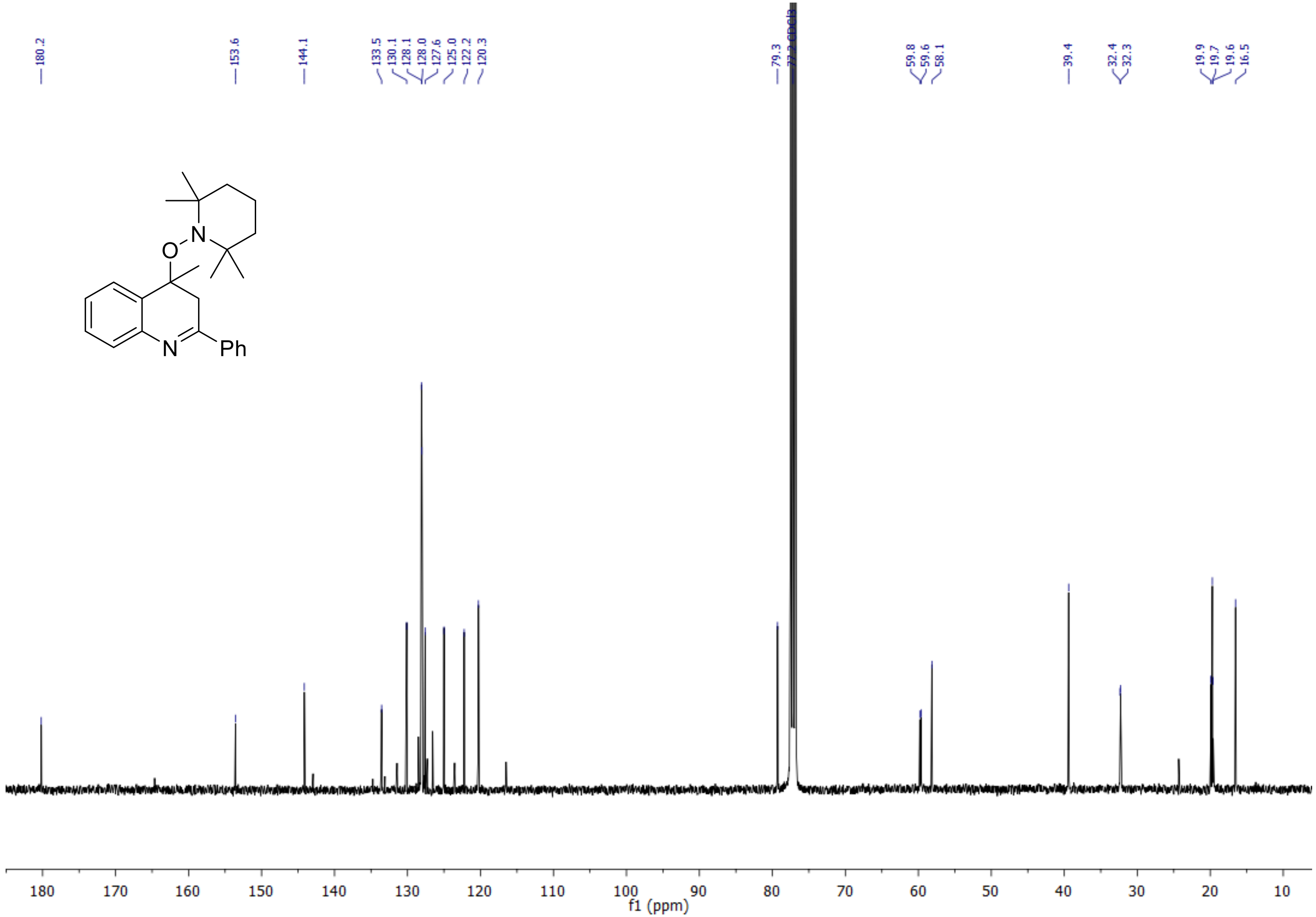

^1H–^{13}C HSQC-DEPT NMR (400 MHz, $CDCl_3$) of **3**

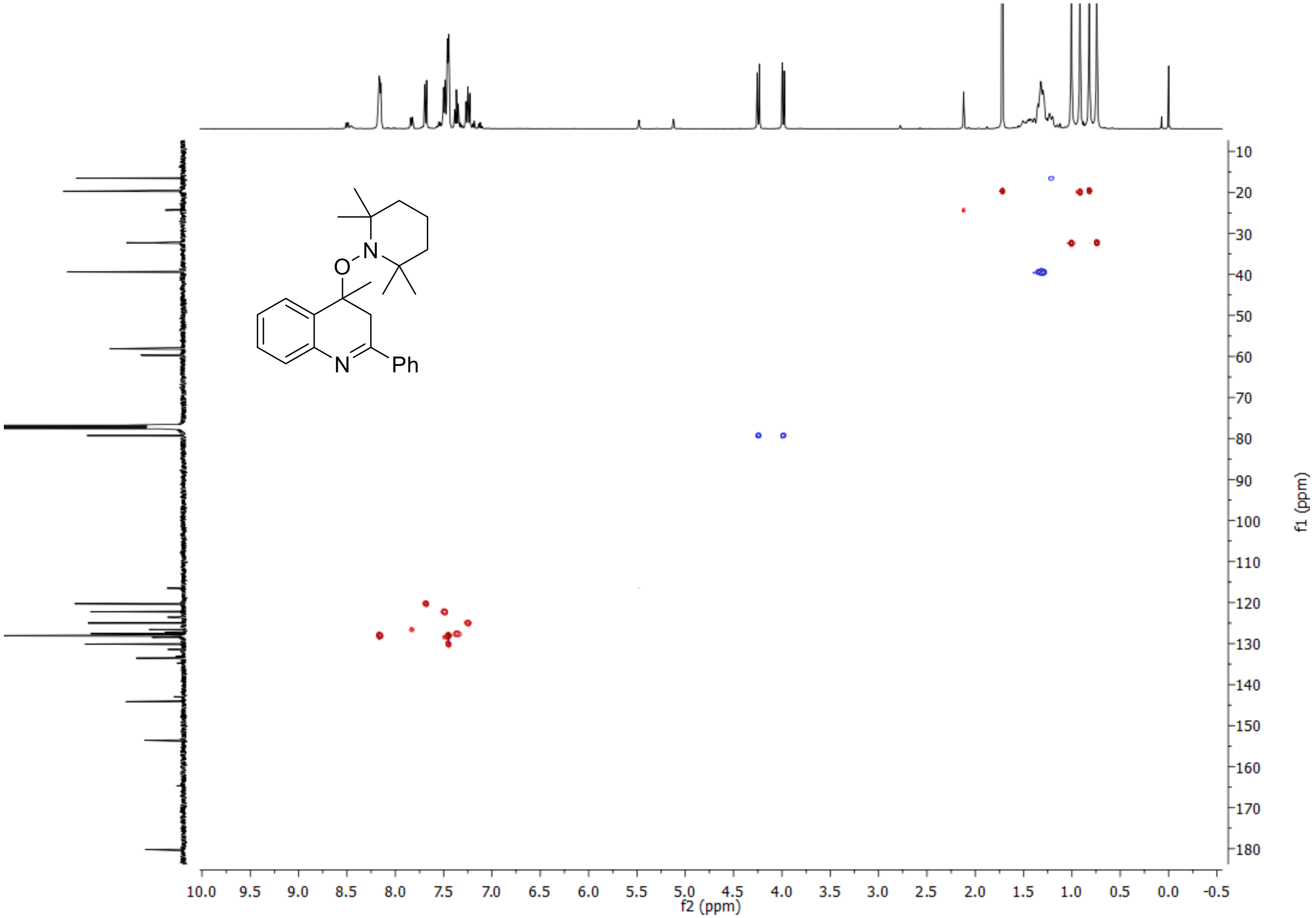

Supplement: Supplementary file 1 — ol1c03934_si_001.pdf [file ol1c03934_si_001.pdf]
